# Analysis of the Nse3/MAGE-Binding Domain of the Nse4/EID Family Proteins

**DOI:** 10.1371/journal.pone.0035813

**Published:** 2012-04-20

**Authors:** Marc Guerineau, Zdenek Kriz, Lucie Kozakova, Katerina Bednarova, Pavel Janos, Jan Palecek

**Affiliations:** 1 Central European Institute of Technology, Masaryk University, Brno, Czech Republic; 2 Functional Genomics and Proteomics, Faculty of Science, Masaryk University, Brno, Czech Republic; 3 National Centre for Biomolecular Research, Faculty of Science, Masaryk University, Brno, Czech Republic; University of South Florida, United States of America

## Abstract

**Background:**

The Nse1, Nse3 and Nse4 proteins form a tight sub-complex of the large SMC5-6 protein complex. hNSE3/MAGEG1, the mammalian ortholog of Nse3, is the founding member of the MAGE (melanoma-associated antigen) protein family and the Nse4 kleisin subunit is related to the EID (E1A-like inhibitor of differentiation) family of proteins. We have recently shown that human MAGE proteins can interact with NSE4/EID proteins through their characteristic conserved hydrophobic pocket.

**Methodology/Principal Findings:**

Using mutagenesis and protein-protein interaction analyses, we have identified a new Nse3/MAGE-binding domain (NMBD) of the Nse4/EID proteins. This short domain is located next to the Nse4 N-terminal kleisin motif and is conserved in all NSE4/EID proteins. The central amino acid residues of the human NSE4b/EID3 domain were essential for its binding to hNSE3/MAGEG1 in yeast two-hybrid assays suggesting they form the core of the binding domain. PEPSCAN ELISA measurements of the MAGEC2 binding affinity to EID2 mutant peptides showed that similar core residues contribute to the EID2-MAGEC2 interaction. In addition, the N-terminal extension of the EID2 binding domain took part in the EID2-MAGEC2 interaction. Finally, docking and molecular dynamic simulations enabled us to generate a structure model for EID2-MAGEC2. Combination of our experimental data and the structure modeling showed how the core helical region of the NSE4/EID domain binds into the conserved pocket characteristic of the MAGE protein family.

**Conclusions/Significance:**

We have identified a new Nse4/EID conserved domain and characterized its binding to Nse3/MAGE proteins. The conservation and binding of the interacting surfaces suggest tight co-evolution of both Nse4/EID and Nse3/MAGE protein families.

## Introduction

The SMC5-6 protein complex is one of the three SMC (Structural maintenance of chromosomes) protein complexes present in all eukaryotes [1], [2]. The SMC5-6 complex is involved in chromatin dynamics such as the response to different types of DNA damage [3], [4]. The core of the complex is formed by the SMC5-SMC6 heterodimer, which is associated with four conserved non-SMC proteins [5], [6], [7]. Yeast Nse1, Nse3 and Nse4 subunits interact with each other and form a tight sub-complex [6], [8], [9]. The Nse4 proteins resemble the kleisin subunits from the other SMC complexes [10], [11]. We have shown that binding of the C-terminal kleisin domain of yeast Nse4 to SMC5 is similar to the binding of the Scc1 kleisin to the SMC1 subunit in the cohesin complex [10], [12]. On the other hand, the N-terminal kleisin motif of *Schizosaccharomyces pombe* Nse4 binds only weakly to SMC6 [10] and the interaction of *Saccharomyces cerevisae* Nse4 with SMC6 was not detected [8]. Our recent data suggest that the Nse1-3-4 subcomplex bridges the SMC5 and SMC6 head domains through both the Nse4 kleisin component and Nse3 subunit [9], [10].

The Nse3 and Nse4 genes are single-member families in all eukaryotic organisms except for placental mammals [9]. In mammals, two protein families have evolved from Nse3 and Nse4, respectively. Nse3 is the founding member of the MAGE (melanoma-associated antigen) protein family [13], [14]. There are tens of MAGE gene (and pseudogene) copies in the human genome. The MAGE proteins share conserved MAGE-homology domains and can be divided into two classes. Genes encoding class I MAGEs (A, B and C sub-families) are expressed only in testis and cancer cells, whereas class II MAGEs are expressed in most tissues. The function of the MAGE proteins is relatively poorly understood, though there is evidence that class I proteins are related to carcinogenesis [15] and several of class II proteins are involved in brain development, apoptosis and differentiation [13].

Only one of the MAGE proteins, hNSE3/MAGEG1, is present in the human SMC5-6 complex and it interacts with both NSE4 kleisin and RING-finger-containing NSE1 subunits [9], [16], [17]. The ability of the other MAGE proteins to bind RING-finger-containing proteins has diverged significantly [9], [17]. However, most MAGE proteins have retained the ability to interact with proteins related to NSE4 (the Nse4/EID family) suggesting a tight evolutionary relationship between Nse3/MAGE and Nse4/EID protein families [9].

The evolution of the Nse4/EID protein family resembles that of the Nse3/MAGE family (there is a single Nse4 gene in most eukaryotes up to non-placental mammals while there are several NSE4/EID copies in placental mammals). In yeasts, the N-terminal part of yeast Nse4 binds to Nse3 [9]. In humans, there are two NSE4 proteins (NSE4a and NSE4b/EID3) containing both N- and C-terminal kleisin domains [10], [16]. Recently, we have shown that both NSE4 proteins interact with most human Nse3/MAGE proteins. The other members of the Nse4/EID protein family (human EID1, EID2 and EID2b), which share sequence homology with the N-terminal part of Nse4 proteins but lack the C-terminal kleisin domain completely [18], were able to interact with some MAGE proteins [9], [19].

In this paper, we first identify a new Nse4 region within its N-terminal part that binds to the previously characterized Nse3 pocket [9]. This short domain is located next to the N-terminal kleisin motif and is conserved in all NSE4/EID proteins. We show that different NSE4/EID proteins interact through this domain with human MAGE proteins. We then focus on the NSE4b-hNSE3/MAGEG1 and EID2-MAGEC2 interactions. Finally we compare our experimental data with *in silico* analysis of this EID2-MAGEC2 heterodimer and present a detailed model of their interaction.

## Results

### Identification of the Nse3-binding domain in the yeast Nse4 protein

**Figure 1:**
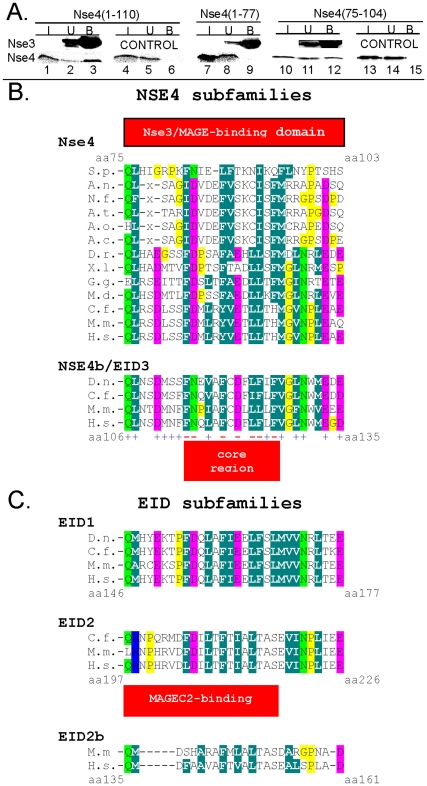
Nse3/MAGE-binding domain within the Nse4/EID family. (**A.**) The His-MBP-tagged fragment of yeast Nse3 (aa 200 - 307) was pre-bound to amylose-beads (lanes 1–3, 7–9 and 10–12) and then incubated with *in vitro* translated fragments spanning aa 1 to 110 (lanes 1–3), aa 1 to 77 (lanes 7–9) and/or aa 75 to 104 (lanes 10–12) of the yeast Nse4 protein. The reaction mixtures were analyzed by 15% SDS–PAGE gel electrophoresis. The amount of the His-MBP-tagged protein was analyzed by immunoblotting with anti-His antibody and the *in vitro* translated proteins were measured by autoradiography. I, input (5% of total); U, unbound (5%); B, bound (40%). Control, no His-MBP-tagged protein present. Alignment of Nse4 (**B.**) and EID (**C.**) subfamilies. The orthologs are from *Schizosaccharomyces pombe* (*S.p.*), *Aspergillus nidulans* (*A.n.*), *Neosartorya fischeri* (*N.f.*), *Aspergillus terreus* (*A.t.*), *Aspergillus clavatus* (*A.c.*), *S. cerevisiae* (*S.c.*), *Danio rerio* (*D.r.*), *Xenopus leavis* (*X.l.*), *Galus galus* (*G.g.*), *Monodelphis domestica* (*M.d.*), *Dasypus novemcinctus* (*D.n.*), *Canis lupus familiaris* (*C.f.*), *Mus musculus* (*M.m.*), *Homo sapiens* (*H.s.*). The EID homologs are present only in some mammals. Red box, Nse3/MAGE-binding domain; blue plus, mutation not affecting interaction; red minus, mutation disrupting the interaction. Amino acid shading represents groups conserved across the family: *dark green*, hydrophobic and aromatic; *light green*, polar; *pink*, acidic; *blue*, basic; all glycine and proline residues are highlighted in *yellow*.

Yeast Nse4 proteins interact with Nse3 and Nse1 forming the Nse1-Nse3-Nse4 subcomplex of the large SMC5-6 complex [6], [8], [20]. In our previous study, we found that the N-terminal half (aa 1-150) of the yeast *S. pombe* Nse4 protein binds to the C-terminal part of its Nse3 partner (aa 200-307; [9]). Figure 1A shows that, His-MBP-tagged Nse3(200-307) protein binds the Nse4(1-110) fragment (lane 3) whereas the first 77 amino acids of Nse4 (encompassing the kleisin motif (aa 13-76)) [10] are not sufficient and may not be necessary for its binding in the *in vitro* pull-down assay (aa 1-77; lane 9). These data suggest that the Nse4 kleisin domain is not sufficient to bind to Nse3 while the amino acids next to the kleisin domain (region 78–110; Fig. 1B) are essential for the Nse4-Nse3 interaction.

Next we expressed the conserved region of Nse4 that was essential for the binding to Nse3 (^75^QLHIGRPKFNIELFTKNIKQFLNYPTSHSN^104^; Fig. 1B) and tested it in our pull-down assay. The His-MBP-tagged Nse3(200-307) protein precipitated the Nse4(75-104) fragment (Fig. 1A, lane 12) suggesting that the yeast Nse4 amino acids from 75 to 104 contain the Nse3-binding domain (Fig. 1B).

### Analysis of the Nse3-binding domain in the human NSE4b protein

**Figure 2:**
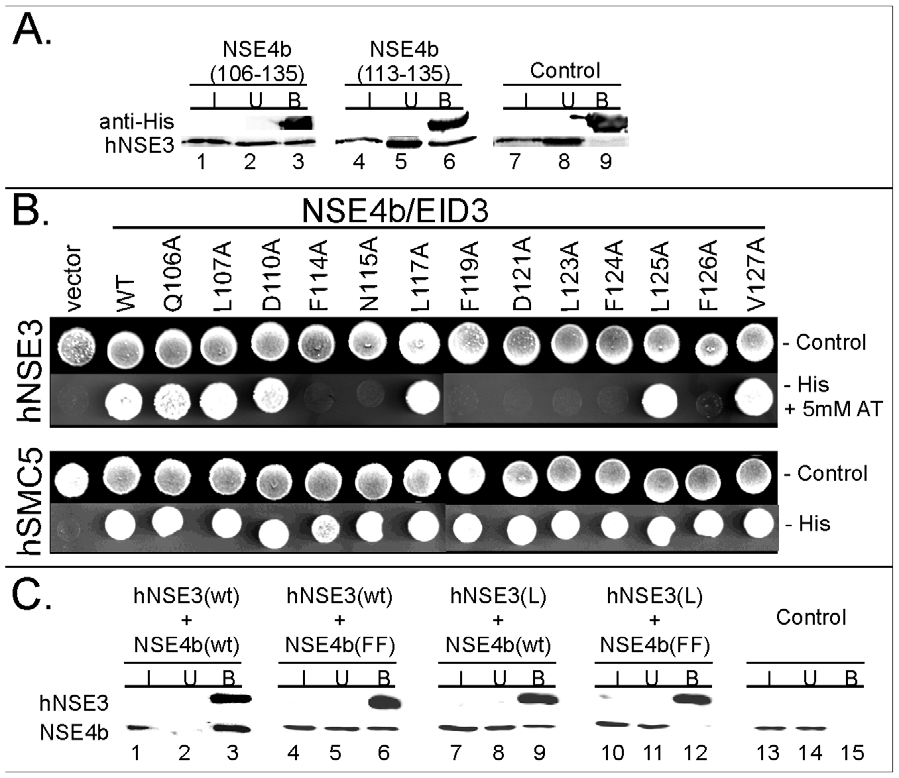
Interactions between human NSE4b and hNse3/MAGEG1. (**A.**) The GST-His-S-NSE4b fragments spanning amino acids 106 to 135 (lanes 1–3) and/or 113 to 135 (lanes 4–6) were pre-incubated with S-protein agarose beads and then incubated with *in vitro* translated full-length hNSE3/MAGEG1 protein (lanes 1–9). The amount of the GST-His-S-tagged protein was analyzed by immunoblotting with anti-His antibody and the *in vitro* translated proteins were measured by autoradiography. Control, GST-His-S-protein present (lanes 7–9). (**B.**) Yeast-2-hybrid analysis of the interaction between the indicated mutants of NSE4b (aa 1 to 333) and hNSE3/MAGEG1 (aa 55 to 292). Interactions result in growth on -Leu, -Trp, -His plates+5 mM AT. Specificity of the NSE4b mutants was checked against the hSMC5 (aa 4 to 1101) construct (interactions result in growth on -Leu, -Trp, -His plates). Control, plate without Leu and Trp. (**C.**) Co-immunoprecipitation from HEK293T cells co-transfected with S-tagged wild-type (wt) and/or mutant (L, L267A) hNSE3/MAGEG1 construct and with FLAG-tagged wild-type (wt) and/or mutant (FF, F114A+F124A) NSE4b/EID3 construct. Lysates were immunoprecipitated with S-protein agarose beads and immunoblotted with either S-HRP (top) or anti-FLAG (bottom). The reaction mixtures were analyzed by 15% SDS–PAGE gel electrophoresis.

The Nse3-binding domain is evolutionarily conserved in Nse4 proteins from yeast to human (Fig. 1B). To determine if the human NSE4b protein binds to hNSE3/MAGEG1 through this conserved region we have tested an NSE4b fragment containing ^106^QLNSDMNFFNQLAFCDFLFLFVGLNWMEGD^135^ in *in vitro* pull-down assays. Figure 2A shows that the GST-His-S-NSE4b(106-135) protein was able to precipitate the *in vitro* expressed hNSE3/MAGEG1 protein (lane 3). The GST-His-S protein alone did not bind hNSE3/MAGEG1 (Fig. 2A, lane 9) suggesting that the NSE4b conserved region interacts specifically with hNSE3/MAGEG1.

To gain further insight into the NSE4b-hNSE3 interaction we have mutated conserved residues within the Nse3-binding domain of NSE4b. Each NSE4b mutant was tested for its ability to interact with hNSE3/MAGEG1 and hSMC5 using the yeast two-hybrid system [9], [10]. 7 out of 20 mutations (F114A, N115A, F119A, D121A, L123A, F124A and F126A) affected the NSE4b binding to hNSE3/MAGEG1 while still interacting with hSMC5, suggesting their specific role in the NSE4b-hNSE3 interaction (Fig. 2B and Fig. S1). Note, the F114A, F119A and F124A mutation reduced the interaction with hNSE3/MAGEG1 most significantly (Fig. S1). Consistent with the hydrophobic character of the hNSE3/MAGEG1 binding pocket [9] most of the hNSE3-specific residues are hydrophobic (Fig. 1B).

To support our yeast two-hybrid data, we have expressed FLAG-NSE4b constructs together with hNSE3/MAGEG1 proteins in HEK293T cells and examined their interactions by immunoprecipitation from cell extracts (Figure 2C). Wild-type hNSE3/MAGEG1 protein co-precipitated most of the wild-type NSE4b (lane 3). The NSE4b(F114A/F124A) mutation reduced its binding to wild-type hNSE3/MAGEG1 (lane 6). Similarly, the hNSE3/MAGEG1(L267A) mutation reduced the binding to wild-type NSE4b (lane 9; [9]). The combination of both NSE4b(F114A/F124A) and hNSE3/MAGEG1(L267A) mutants abolished the NSE4b-hNSE3 binding completely (Fig. 2C, lane 12). These data suggest that the Nse3-binding domain residues and residues in the hNSE3/MAGEG1 pocket are involved in the NSE4b-hNSE3 interaction.

The Q106A, L107A, D110A, M111A, N112A and F113A substitutions had no effect to NSE4b-hNSE3 binding (Figure 2B and. Fig. S1). Consistent with these results a shorter GST-His-S-NSE4b(113-135) fragment (missing residues 106–113) bound the hNSE3/MAGEG1 in pull down assays (Fig. 2A, lane 6). These data suggest that the central conserved residues within the Nse3-binding domain form the core binding motif in the Nse4 proteins from yeast to human (Fig. 1B).

### Human MAGE proteins interact with the Nse3-binding domain of the NSE4b protein

**Figure 3:**
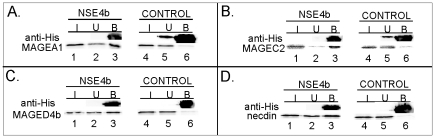
MAGE proteins bind to Nse3-binding domain of the NSE4b protein. The GST-His-S-tagged fragment of human NSE4b(106 - 135) was bound to S-protein agarose beads (lanes 1–3) and then incubated with *in vitro* translated class I (panel **A** and **B**, lanes 1–6) and/or class II (panel **C** and **D**, lanes 1–6) MAGE proteins: MAGEA1 (aa 1-309; panel **A**), MAGEC2 (aa 6-373; panel **B**), MAGED4b (aa 1-741; panel **C**) and necdin (aa 1-321; panel **D**). The reaction mixtures were analyzed by 15% SDS–PAGE gel electrophoresis. The amount of the GST-His-S-tagged protein was analyzed by immunoblotting with anti-His antibody and the *in vitro* translated proteins were measured by autoradiography. Control, GST-His-S-tag protein (lanes 4–6).

The human NSE4b (and NSE4a) protein is able to bind not only the hNSE3/MAGEG1 subunit of the human SMC5-6 complex but other human MAGE proteins, which are not part of this complex, as well [9]. To test if the NSE4b protein interacts with class I and class II MAGE proteins through the Nse3-binding domain we have incubated GST-His-S-NSE4b(106-135) with different MAGE proteins. Figure 3 shows that the NSE4b(106-135) fragment is able to precipitate class I proteins, MAGEA1 and MAGEC2, in the pull-down assays (panel A and B, lane 3). Similarly, the MAGED4b and necdin (representatives of class II) proteins bound to the NSE4b(106-135) fragment (Fig. 3, panel C and D, lane 3). Altogether, our results suggest that the conserved Nse3-binding domain of the NSE4b protein mediates interaction with hNSE3/MAGEG1 as well as with other MAGE proteins [9].

### Interactions between NSE4/EID family members and MAGE proteins

**Figure 4:**
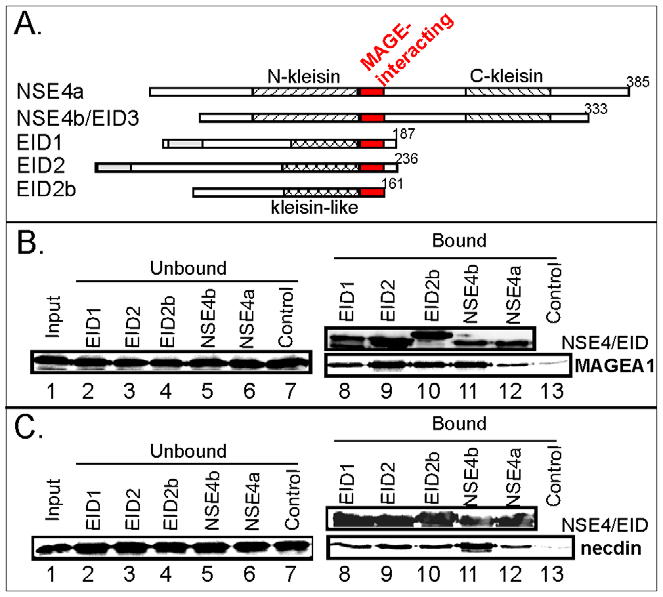
NSE4/EID proteins interact with MAGE proteins through the Nse3/MAGE-binding domain. (**A.**) Alignments of the five members of the human NSE4/EID family: EID1, EID2, EID2b, EID3/NSE4b and NSE4a. The red box indicates Nse3/MAGE-binding domain; hatched and crosshatched boxes indicate kleisin and kleisin-like motifs, respectively (based on Fig. 1B and C; [10]); grey boxes indicate regions of homology between EID1 and 2, respectively. (**B. and C.**) GST-His-S-tagged fragments of each of the NSE4/EID family members were pre-bound to S-protein agarose beads and incubated with *in vitro* translated MAGEA1 (aa 1-309; panel **B**) and necdin (aa 1-321; panel **C**). The indicated NSE4/EID proteins correspond to the MAGE-interacting domain of EID1(aa146-177), EID2(aa197-225), EID2b(aa135-161), NSE4b/EID3(aa106-135) and NSE4a(aa150-179). In lanes 8-12, immunoblotting of bound fragments are shown in the upper panel and bound radioactive MAGE proteins (MAGEA1 and necdin) in the lower panel. Control, GST-His-S protein alone.

The NSE4b/EID3 protein is member of the EID family of proteins [18]. The EID1, 2 and 2b members interact with MAGE proteins [9] and show sequence similarities to the N-terminus of the NSE4b/EID3 (and NSE4a) protein including the Nse3-binding domain (Fig. 1 and 4A). We have generated fragments of each human NSE4/EID family member homologous to the Nse3-binding domain and expressed them as GST-His-S-tagged NSE4/EID proteins in *E. coli*. The bacterial extracts were pre-incubated with S-protein agarose beads and tested for their binding to *in vitro* translated MAGEA1 (class I) and necdin (class II) protein, respectively. All the homologous NSE4/EID fragments interacted with both MAGEA1 and necdin albeit with different affinity (Figure 4B and C, lanes 8 to 12). We conclude that the interaction of the MAGE proteins with the NSE4/EID family members is mediated by the conserved Nse3/MAGE-binding domain homologous to the Nse3-binding domain identified above (Figs. 1, 2, 3).

### Analysis of the conserved residues within the Nse3/MAGE-binding domain of EID2

**Figure 5:**
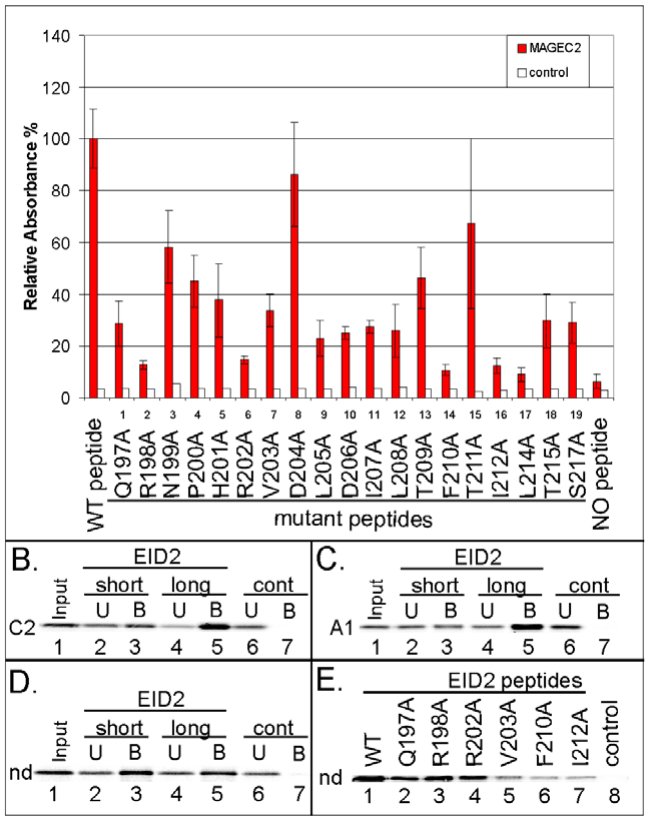
Analysis of EID2 binding to MAGE proteins. (**A.**) Quantification of relative binding of the MAGEC2(129-339) protein (red columns) to the EID2 protein-based synthetic mutant peptides (listed in Table 1) using the PEPSCAN-ELISA method. Results show mean ± SEM of 3 independent measurements. His-hTRF2 protein (white column) was used in the control experiment. (**B. to D.**) The short (biotin-SGSG-^201^HRVDLDILTFTIALTAS^217^) and long (biotin-SGSG-^197^QRNPHRVDLDILTFTIALTAS^217^) EID2 peptides were pre-bound to the streptavidin-agarose beads and then incubated with *in vitro* translated MAGEC2 (aa 6-373; C2 in panel **B.**), MAGEA1 (aa 1-309; A1 in panel **C.**) and/or necdin (aa 1-321; nd in panel **D.**) protein, respectively. (**E.**) Wild type and selected EID2 mutant peptides (as indicated) were pre-bound to the streptavidin-agarose beads and then incubated with *in vitro* translated necdin protein. The reaction mixtures were analyzed by 15% SDS–PAGE gel electrophoresis. The amount of the *in vitro* translated proteins was measured by autoradiography. Control, no peptide.

To further characterize the Nse3/MAGE-binding domain of the EID proteins [9] we used synthetic EID2 peptides carrying specific mutations (Table 1; alanine scan). A library of the mutant EID2 peptides bound to ELISA plates was tested against the purified MAGEC2(129-339) protein. Only with the mutant peptide #8 (D204A substitution) was the binding affinity to MAGEC2 comparable to that of the wild-type peptide (^197^QRNPHRVDLDILTFTIALTAS^217^; Fig. 5A) while the affinity of the other mutant peptides was reduced. Particularly, the affinities of the mutant peptide #2 (R198A), #6 (R202A), #14 (F210A), #16 (I212A) and #17 (L214A) were reduced to background levels (last lane; less then 20% of WT affinity) suggesting that the residues R198, R202, F210, I212 and L214 are essential for the EID2-MAGEC2 interaction (Fig. 5A). In addition, the substitution in mutant peptide #1 (Q197A), #9 (L205A), #10 (D206A), #11 (I207A), #12 (L208A), #18 (T215A) and #19 (S217A) significantly reduced the EID2 binding affinity to MAGEC2 (∼30% of wt) indicating the importance of these residues for the binding to MAGEC2 (Fig. 5A). The binding affinity of the other peptides was reduced modestly suggesting that these residues may either physically interact with MAGEC2 or stabilize the EID2 conformation. Interestingly, the EID2 residues L205, D206, F210, I212, L214, T215 and S217 correspond to the NSE4b/EID3 essential residues (F114, N115, F119, D121, L123, F124 and F126; Fig. 2B) demonstrating that the binding of the core region is conserved from NSE4b to EID2 proteins (Fig. 1B and C).

**Table 1:** List of EID2 peptides used in PEPSCAN ELISA assays.

EID2 peptide	
number	EID2 peptide sequence
WT peptide	QRNPHRVDLDILTFTIALTAS
peptide #1	**A**RNPHRVDLDILTFTIALTAS
peptide #2	Q**A**NPHRVDLDILTFTIALTAS
peptide #3	QR**A**PHRVDLDILTFTIALTAS
peptide #4	QRN**A**HRVDLDILTFTIALTAS
peptide #5	QRNP**A**RVDLDILTFTIALTAS
peptide #6	QRNPH**A**VDLDILTFTIALTAS
peptide #7	QRNPHR**A**DLDILTFTIALTAS
peptide #8	QRNPHRV**A**LDILTFTIALTAS
peptide #9	QRNPHRVD**A**DILTFTIALTAS
peptide #10	QRNPHRVDL**A**ILTFTIALTAS
peptide #11	QRNPHRVDLD**A**LTFTIALTAS
peptide #12	QRNPHRVDLDI**A**TFTIALTAS
peptide #13	QRNPHRVDLDIL**A**FTIALTAS
peptide #14	QRNPHRVDLDILT**A**TIALTAS
peptide #15	QRNPHRVDLDILTF**A**IALTAS
peptide #16	QRNPHRVDLDILTFTA**A**LTAS
peptide #17	QRNPHRVDLDILTFTIA**A**TAS
peptide #18	QRNPHRVDLDILTFTIAL**A**AS
peptide #19	QRNPHRVDLDILTFTIALTA**A**

The substitutions Q197A and R198A within the N-terminus of the EID2 peptide significantly reduced the EID2 binding to the MAGEC2(129-339) protein in the ELISA assays (Fig. 5A; peptides #1 and #2). To verify these findings, we pre-bound the short (^202^RVDLDILTFTIALTAS^217^) and long (^197^QRNPHRVDLDILTFTIALTAS^217^) biotin-tagged peptide to streptavidin-beads and then incubated them with *in vitro* expressed MAGEC2(6-373) and/or MAGEC2(129-339) protein (Fig. 5B and not shown). The binding affinity of the MAGEC2 proteins to short EID2 peptide (missing ^197^QRNPH^201^ region) was significantly lower than the affinity to long peptide suggesting that both Q197 and R198 are involved in the EID2-MAGEC2 interaction. Similar data were obtained for the MAGEA1 protein (Fig. 5C).

In contrast, necdin bound both short and long peptide with similar affinity (Fig. 5D, lanes 3 and 5) and the binding of the mutant peptide #1 (Q197A) and #2 (R198A) to necdin were comparable to the wild-type peptide (Fig. 5E, lanes 1 to 3) demonstrating that the ^197^QRNPH^201^ region is not involved in the EID2-necdin interaction. However, similar to the MAGEC2 data, binding of the mutant peptide #14 (F210A) and #16 (I212A) was significantly reduced (Fig. 5E, lanes 6 and 7) suggesting that the core hydrophobic residues mediate the EID2-necdin interaction. Interestingly, the EID2 ^197^QRNPH^201^ residues correspond to NSE4b/EID3 residues ^106^QLNSD^111^ which were not essential for the binding to hNSE3/MAGEG1 (Fig. 2B). These results suggest that the necdin (class II protein) interacts with EID2 through the core binding motif in the way similar to NSE4b-hNSE3 binding while extra amino acids in front of this core region are necessary for the proper binding to class I (MAGEA1 and MAGEC2) proteins.

### Docking of the EID2 peptide onto MAGEC2 surface

**Figure 6:**
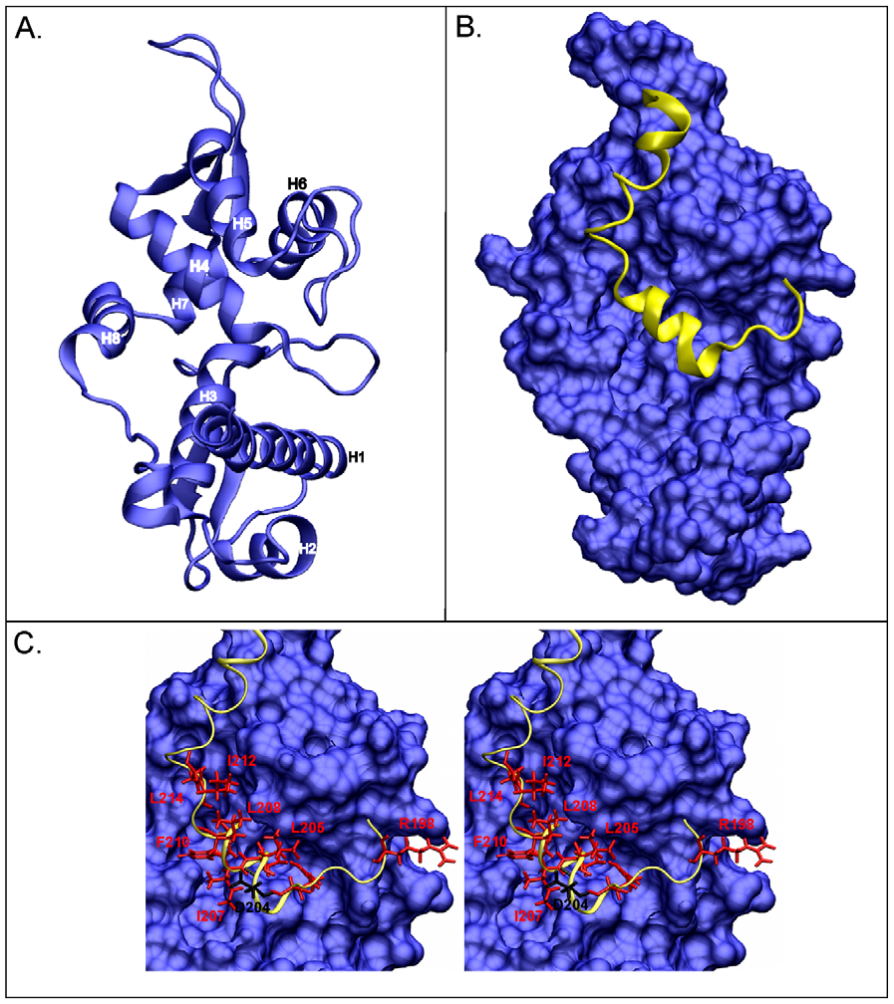
Docking model of the EID2-MAGEC2 heterodimer. Homology modelling was used to generate the predicted MAGEC2 (aa133-336) and EID2 (^197^QRNPHRVDLDILTFTIALTASEVINPLIEE^226^) structure. (**A.**) Ribbon representation of the predicted MAGEC2 3D structure model (blue; helices indicated as in [9]). (**B.**) MAGEC2 surface view (blue) with docked EID2 peptide (yellow; ribbon representation). (**C.**) Stereoscopic detailed view of the MAGEC2 pocket with bound EID2 peptide. The EID2 residues involved in the binding to MAGEC2 are indicated in red (Fig. 5A). The central EID2 amino acid residues (L205, D206, I207, L208, F210, I212 and L214) are in physical contact with the MAGEC2 pocket surface (formed by H4, H5 and H8). The N-terminus of the Nse3/MAGE-binding domain makes contact through the essential residues R198 and R202 (only R198 is labeled) to the MAGEC2 loop region (between H5 and H6). The D204 residue protruding to the solvent is black labeled.

In order to interpret our data we modelled the structure of MAGEC2 on the MAGEA4 (PDB entry 2WA0) and hNSE3/MAGEG1 (PDB entry 3NW0) crystal structures using Chimera software and molecular dynamics (MD) simulations (Fig. 6A; [9]). The structure of the Nse3/MAGE-binding domain of the EID2 protein (^197^QRNPHRVDLDILTFTIALTASEVINPLIEE^226^) was calculated using the I-TASSER server and MD simulations [21]. Then we used HEX software [22] to dock the EID2 peptide into the previously characterized Nse4/EID-binding pocket at the MAGE surface (formed by H4, H5 and H8 helices; Fig.6 A and B; [9]). The highest scoring structures were used for evaluation by MD simulation in an explicit solvent model and their free binding energies were calculated (Table S1 and Fig. S2; Materials and Methods).

The model #18 exhibited the lowest free binding energy and the EID2 core region (^205^LDILTFTIALTAS^217^) fitted best into the hydrophobic pocket of MAGEC2 (Fig. 6B). Consistent with our ELISA data (Fig. 5A), the Q197, R198, R202, L205, D206, I207, L208, F210, I212, L214 and S217 residues interact with MAGEC2 while the D204 residue protrudes into the solvent (Fig. 6C). In this model, all the core region amino acid residues (except the T215 residue) are in physical contact with the MAGEC2 pocket surface while the N-terminus of the Nse3/MAGE-binding domain (^197^QRNPHRVD^204^) makes contact with the MAGEC2 loop region. We conclude that the core region is responsible for the majority of the contacts between the NSE4/EID and Nse3/MAGE proteins. However, the N-terminal part of the Nse3/MAGE-binding domain can stabilize the binding of the Nse4/EID proteins to class I MAGE partners.

## Discussion

In our previous studies we showed that Nse1, Nse3 and Nse4 formed a sub-complex within the highly conserved SMC5-6 protein complex. This subcomplex makes several contacts with the head domains of the SMC5-SMC6 heterodimer [10]. In particular, the conserved Nse4 C-terminal kleisin motif binds the SMC5 head in yeasts [8], [10]. Here we show that human NSE4b binds hSMC5 (Fig. 2B) suggesting the evolutionary conservation of the Nse4-SMC5 interaction from yeast to human. Interestingly, the EID proteins lack the C-terminal kleisin domain (Fig. 4A) and they are not incorporated into the human SMC5-6 complexes [9].

The role of the conserved Nse4 N-terminal part is not known. Recently, we have shown that the yeast Nse4 N-terminal part mediates interactions with both Nse1 and Nse3 [9]. We previously characterized a conserved hydrophobic pocket at the surface of the Nse3/MAGE proteins that interacts with Nse4/EID proteins. Here we have identified an Nse3/MAGE-binding domain within the N-terminal part of both yeast Nse4 and human NSE4b (Figs. 1A and 2A). This domain is immediately C-terminal to the kleisin motif and is well conserved not only in all Nse4 proteins but also in mammalian EID proteins (Fig. 1B and C).

The sequence conservation of Nse3/MAGE pockets and NSE4/EID domain suggests their tight evolutionary connection. Moreover, the NSE4/EID family shows a similar pattern of evolutionary diversification to the MAGE family (Fig. 1B and C; [9], [23]). There is a single Nse3 and Nse4 gene in most eukaryotes up to non-placental mammals while there are multiple copies in placental mammals (up to 5 NSE4/EID copies and tens of NSE3/MAGE copies). We hypothesize that these two protein families have co-evolved in placental mammals.

Both our Y2H (Fig. 2B) and peptide-binding (Fig. 5) data suggest that there is a core region within the NSE4b and EID2 domain that mediates interaction with hNSE3/MAGEG1 and MAGEC2, respectively (similar ELISA results were obtained with MAGEA3(94-314) protein; M.G., unpublished data). Molecular modelling of the EID2-MAGEC2 heterodimer shows that the EID2 core region is helical and fits into the MAGEC2 pocket (Fig. 6). The core region is most conserved in Nse4/EID domains and gives a similar helical appearance when modelled with the I-TASSER server and MD simulations (J.P. and Z.K., unpublished data). We conclude that the core region of most Nse4/EID proteins interact with Nse3/MAGE pockets in a similar way to the EID2-MAGEC2 heterodimer.

Evolutionary variability of the residues within both MAGE pockets and NSE4/EID domain may account for different selective MAGE-EID pairing [9]. While the hNSE3/MAGEG1-NSE4b/EID3 (and NSE4a) sequences were under high selective pressure to keep the SMC5-6 complex functional (and possibly avoid hNSE3/MAGEG1 interactions with EID proteins outside the complex) the relatively recent class I MAGE proteins could have modified their EID-MAGE binding mode [23]. For example, the N-terminal extension of the EID2 core binding region strengthens the interaction with class I MAGE proteins (Fig. 5; M.G. and J.P., unpublished data). According to our EID2-MAGEC2 model, the N-terminal extension region of EID2 binds to the loop region of MAGEC2 (between H5 and H6; Fig. 6) outside the conserved hydrophobic pocket. Interestingly, the EID2b protein is missing most of the N-terminal extension and can not bind to MAGEC2 (Fig. 1C; [9]). Thus the evolutionary diversification of these additional regions may contribute to different EID-MAGE binding affinities and hence determine their specific pairing [9].

Finally, different expression patterns of MAGE and NSE4/EID proteins determine tissue specific heterodimer formation and function [9], [13], [24], [25]. For example, necdin is highly expressed in the nervous system where it antagonizes the repressive effect of EID1 and promotes differentiation of neuronal precursor cells [19]. It was proposed that necdin binding to EID1 can release it from p300 co-activator association and thus enhance p300-dependent transcription of differentiation-specific genes. Consistent with this hypothesis, the necdin/MAGE-binding domain of the EID1 protein (aa 146-177; Figs. 1C and 4C) overlaps with one of the p300-binding sites which are essential for the p300-EID1 interaction [24]. In addition, our preliminary data suggest that the p300(aa1645-1845) protein can bind to EID2(aa163-236) fragment [26] and the MAGEC2(aa129-339) can inhibit this binding in *in vitro* competition experiments (M.G., unpublished data). More detailed studies need to be carried out in future work in order to unravel the nature of these complex interactions and to understand the functions of both Nse3/MAGE and Nse4/EID protein families in their normal cellular contexts.

## Materials and Methods

### Plasmids

All pTriEx4 and pET41 plasmids described in this study were generated by PCR and ligase-independent cloning (Merck). The primers used for PCR amplification are listed in Table S2. For the pET-MBP-Nse3(aa200-307) construct, the NcoI-XhoI fragment of pTriEx4-Nse3(200-307) [9] was inserted into pET-MBP digested with *Nco*I-*Xho*I restriction enzymes (kindly provided from Dr. Lumir Krejci; [27]). The *Bam*HI-*Sal*I fragment of pTriEx4-hSMC5(aa4-1101) was cloned into pGBKT7 yeast-2-hybrid vector. The other constructs were described previously [9].

### Site-directed mutagenesis

The QuikChange Lightning Site-Directed Mutagenesis system (Agilent Technologies) was used to create single point mutations in the pGADT7-NSE4b(aa1-333) plasmid and two mutations in plasmid pCI-neo-FLAG-NSE4b [9]; the primers are listed in Table S3.

### Pull down assays

Proteins carrying (GST-)His-S tag-fusion were expressed using bacterial strain C41. Protein extracts were pre-incubated with S-protein agarose beads (Merck). Similarly, His-MBP-tag-fusion protein extracts were pre-incubated with amylose resin (New England Biolabs). Then *in vitro*-expressed proteins in a total volume of 200 µl of HEPES buffer were added and incubated overnight [10]. Input, unbound, and bound fractions were separated by SDS-PAGE, transferred to nitrocellulose membranes, and analyzed by phosphorimaging and immunoblotting with anti-His antibody (Sigma).

Biotin-tagged EID2 peptides were preincubated overnight with streptavidin-agarose beads (Thermo Scientfic) at 4°C. The beads were washed 2 times with 0.1% Tween 20 in PBS buffer, then *in vitro*-expressed proteins in a total volume of 200 µl PBS buffer were added and incubated for 2 h at 4°C. The beads were washed 4 times with 0.1% Tween 20 in PBS buffer and the bound proteins were eluted in SDS sample buffer and boiled. Input, unbound, and bound fractions of the *in vitro* expressed proteins were separated by SDS-PAGE and analyzed by phosphorimaging (FLA-7000, Fujifilm). The bound fractions were analyzed by dot blotting to ensure equal EID2 peptide levels were used in pull down assays (data not shown).

### Immunoprecipitations

Lysates were made from transfected HEK293T (DSMZ, Germany) cells by scraping in lysis buffer (50 mM Tris-HCl pH 7.7, 0.5% NP40, 150 mM NaCl, 1 mM DTT, 1× protease inhibitor cocktail [Roche]) and sonication. Lysates were incubated for 15 minutes on ice and cleared by centrifugation at 13000 rpm for 15 minutes. Agarose beads conjugated to S protein (Merck) were mixed with lysates for 4 hours at 4°C. Beads were washed 3 times with lysis buffer and resuspended in SDS loading buffer.

### Expression and purification of recombinant MAGEC2 protein in *E. coli*

His-S-MAGEC2(129-339) was expressed in C41 cells on LB media. The cells were grown at 37°C to A_600_ of 0.5 and induced by 1 mM isopropoyl-β-thiogalactopyranoside (IPTG) for 4 h at 22°C. The cells were harvested and lysed by sonication in the buffer containing 50 mM NaH_2_PO_4_/Na_2_HPO_4_ (pH 7.4), 150 mM NaCl and 10 mM imidazole. His-S-MAGEC2 was purified by Fast Protein Liquid Chromatography (FPLC) with a His-TRAP 1 ml column (GE Healthcare) using imidazole step gradient protocol following the manufacturer's instructions.

### PEPSCAN ELISA assays

A peptide library of the alanine mutant peptides (Table 1) of the aa 197-217 region of the human EID2 protein was obtained from Mimotopes (Australia). The library was linked to biotin via an additional peptide spacer of serine–glycine–serine–glycine. Each well on ELISA 96-well plates was coated with 100 µl of 5 µg/ml streptavidin (Sigma), incubated overnight at 37°C and then blocked with 3% bovine serum albumin (BSA) in PBS for 2 h at room temperature [28]. The biotinylated peptides (400 nM, in PBS with 0.1% BSA) were applied into wells to saturate their surface and incubated at room temperature for 1 h. The plates were washed three times with 0.1% Tween 20 in PBS and then His-S-MAGEC2(129-339) protein was added (200–400 nM). Plates were incubated at 4°C overnight before washing (as above) to remove unbound protein. First anti-His antibody (Sigma; diluted 1/1000 in PBS with 1% BSA) was incubated for 2 h, washed three times and secondary peroxidase-conjugated rabbit-antiserum against mouse immunoglobulins (DAKO; diluted 1/1000 in PBS with 1% BSA) was added for 1 h (at 4°C). Peroxidase enzyme activity was measured with tetramethylbenzidine and the results were monitored using an automatic ELISA plate reader at a wavelength 450 nm.

### Yeast two-hybrid assays

The Gal4-based two-hybrid system was used to analyze NSE4b mutants. Each pGADT7-NSE4b(aa1-333) mutant was co-transformed either with the pGBKT7-hNSE3(aa55-292) or pGBKT7-hSMC5(aa1-328) construct [9]. Colonies were inoculated into YPD media and cultivated overnight. 10-fold dilutions were dropped onto the SD-Leu, -Trp (control), SD-Leu, -Trp, -Ade, and SD-Leu, -Trp, -His (with 0, 1, 2, 5, 10, 15, 20, 30, 60 mM 3-aminotriazole) plates. The β-galactosidase activity was assayed using ONPG (o-nitrophenyl β-D-galactopyranoside) according to the manufacturer's protocol (Clontech, TAKARA Bio Company). Each mutant was co-transformed at least three times into *S. cerevisiae* PJ69-4a and at least two independent tests were carried out from each transformation. In addition, the mutant protein expression levels were verified by immunoblotting.

### Molecular modeling

The starting structure of the MAGEC2 protein was modelled using MAGEA4 (PDB entry 2WA0) and hNSE3/MAGEG1 (PDB entry 3NW0) as template with Chimera software. The structure of the protein model was relaxed using 5 ns long molecular dynamics (MD) simulations performed by AMBER software package. The structure of EID2 protein was created using I-TASSER server. The conformations for the docking study were created by using series of simulated annealing procedures with different heating temperatures. Based on backbone conformation these models were classified into eight different groups. Eight models representing each group were used for the docking study using HEX software [22]. First, the possible position of the EID2 peptide fragment on the MAGEC2 protein was studied using Macro Docking option of the HEX software. The rough complex models were used for more precise docking using the HEX software with shape complementarity followed by molecular mechanics refinement. The four highest scoring structures were used for evaluation by molecular dynamics simulation in the explicit solvent model.

The complex structures were solvated using the SOLVATE software with adding of sodium ions to neutralize the charge of the system and with adding of sodium chloride at physiological concentration of 150 mM. The TIP3P explicit water model was used in all simulations [29]. The octahedral box around the solvated complex was added using the *Leap* module of the Amber software package [30].

The system was relaxed by the series of minimizations and low temperature molecular dynamics simulations applied on water molecules and ions followed by MD simulations with restraints applied on backbone atoms of proteins. Finally the system was slowly heated to the simulation temperature 298.15 K followed by 50 ps MD simulation.

The produced phase was performed by the Pmemd module of Amber software package. The simulations were performed under periodic boundary conditions in the [NpT] ensemble at 298.15 K and pressure of 1 atm using 2 fs integration step. The SHAKE algorithm, with tolerance of 10^−5^ Å, was used to fix the positions of all hydrogen atoms, and 9.0 Å cutoff applied to non bonding interactions [31]. The Berendsen thermostat was used [32]. The potential and kinetic energies together with density of the system was monitored during the production phase. The progression of the trajectories was monitored by rms deviation calculated using the *Ptraj* module of the Amber software package. The binding free energies calculated from the MD trajectories using the MM/PBSA and MM/GBSA models were used for evaluation of complexes (Table S1). The *MMPBSA.py* program from the AmberTools version 1.5 software package [30] was used for this analysis. The last 1000 steps of MD simulations were used for the MM/PBSA and MM/GBSA analysis. The atom coordinates for the best model (#18) with the lowest free binding energy are shown in Table S4. Structures were visualized using VMD software [33].

## Supporting Information

Figure S1 **Binding of the hNSE3/MAGEG1 to NSE4b/EID3 protein.** Yeast-2-hybrid analysis of the interaction of the indicated mutants of human NSE4b (aa 1 to 333) with hNse3/MAGEG1 (**A.** and **B.**) and/or hSMC5 (**C.**). Interactions result in growth on -Leu, -Trp, -His plates (with or without aminotriazole, AT) and -Leu, -Trp, -Ade plates. Control, plate without Leu and Trp. (**B.**) Selected NSE4b-hNSE3 pairs were assayed for β-galactosidase activity.
(TIF)

Figure S2 **Comparison of the best four scored structures of the EID2-MAGEC2 docking.** Superposition of starting structure (blue) and structure after 1 ns molecular dynamics simulations (red). The MAGEC2(129-339) protein is visualized by surface and the EID2 peptide is visualized by ribbon model. The model #18 (panel **C**) exhibited the lowest free binding energy (Table S1) and the EID2 core region (central helical part) fitted best into the MAGEC2 pocket.
(TIF)

Table S1 Free binding energies calculated from MD trajectories.
(DOC)

Table S2 Primers used for cloning.
(DOC)

Table S3 Primers used for site-directed mutagenesis of human hNSE4b/EID3.
(DOC)

Table S4 The MAGEC2(133-336)-EID2(197-225) atom coordinates (PDB file).
(DOC)

## References

[1] Murray JM, Carr AM (2007). Smc5/6: a link between DNA repair and unidirectional replication?. Nat Rev Mol Cell Biol.

[2] De Piccoli G, Torres-Rosell J, Aragon L (2009). The unnamed complex: what do we know about Smc5-Smc6?. Chromosome Res.

[3] Lehmann AR (2005). The role of SMC proteins in the responses to DNA damage.. DNA Repair (Amst).

[4] Lehmann AR, Walicka M, Griffiths DJF, Murray JM, Watts FZ (1995). The *rad18* gene of *Schizosaccharomyces pombe* defines a new subgroup of the SMC superfamily involved in DNA repair.. Mol Cell Biol.

[5] Fousteri MI, Lehmann AR (2000). A novel SMC protein complex in *Schizosaccharomyces pombe* contains the Rad18 DNA repair protein.. EMBO J.

[6] Sergeant J, Taylor E, Palecek J, Fousteri M, Andrews E (2005). Composition and architecture of the *Schizosaccharomyces pombe* Rad18 (Smc5-6) complex.. Mol Cell Biol.

[7] Taylor EM, Moghraby JS, Lees JH, Smit B, Moens PB (2001). Characterization of a novel human SMC heterodimer homologous to the *Schizosaccharomyces pombe* Rad18/Spr18 complex.. Mol Biol Cell.

[8] Duan X, Yang Y, Chen YH, Arenz J, Rangi GK (2009). Architecture of the Smc5/6 Complex of *Saccharomyces cerevisiae* Reveals a Unique Interaction between the Nse5-6 Subcomplex and the Hinge Regions of Smc5 and Smc6.. J Biol Chem.

[9] Hudson JJR, Bednarova K, Kozakova L, Liao C, Guerineau M (2011). Interactions between the Nse3 and Nse4 components of the SMC5-6 complex identify evolutionarily conserved interactions between MAGE and EID families.. PLOS One.

[10] Palecek J, Vidot S, Feng M, Doherty AJ, Lehmann AR (2006). The SMC5-6 DNA repair complex: Bridging of the SMC5-6 heads by the Kleisin, NSE4, and non-Kleisin subunits.. J Biol Chem.

[11] Schleiffer A, Kaitna S, Maurer-Stroh S, Glotzer M, Nasmyth K (2003). Kleisins: a superfamily of bacterial and eukaryotic SMC protein partners.. Mol Cell.

[12] Haering CH, Schoffnegger D, Nishino T, Helmhart W, Nasmyth K (2004). Structure and Stability of Cohesin's Smc1-Kleisin Interaction.. Mol Cell.

[13] Barker PA, Salehi A (2002). The MAGE proteins: emerging roles in cell cycle progression, apoptosis, and neurogenetic disease.. J Neurosci Res.

[14] Chomez P, De Backer O, Bertrand M, De Plaen E, Boon T (2001). An overview of the MAGE gene family with the identification of all human members of the family.. Cancer Res.

[15] Caballero OL, Zhao Q, Rimoldi D, Stevenson BJ, Svobodova S (2010). Frequent MAGE mutations in human melanoma.. PLoS One.

[16] Taylor EM, Copsey AC, Hudson JJ, Vidot S, Lehmann AR (2008). Identification of the proteins, including MAGEG1, that make up the human SMC5-6 protein complex.. Mol Cell Biol.

[17] Doyle JM, Gao J, Wang J, Yang M, Potts PR (2010). MAGE-RING protein complexes comprise a family of E3 ubiquitin ligases.. Mol Cell.

[18] Bavner A, Matthews J, Sanyal S, Gustafsson JA, Treuter E (2005). EID3 is a novel EID family member and an inhibitor of CBP-dependent co-activation.. Nucleic Acids Res.

[19] Bush JR, Wevrick R (2008). The Prader-Willi syndrome protein necdin interacts with the E1A-like inhibitor of differentiation EID-1 and promotes myoblast differentiation.. Differentiation.

[20] Pebernard S, Wohlschlegel J, McDonald WH, Yates JR, Boddy MN (2006). The Nse5-Nse6 dimer mediates DNA repair roles of the Smc5-Smc6 complex.. Mol Cell Biol.

[21] Roy A, Kucukural A, Zhang Y (2010). I-TASSER: a unified platform for automated protein structure and function prediction.. Nat Protoc.

[22] Ritchie DW, Kozakov D, Vajda S (2008). Accelerating and focusing protein-protein docking correlations using multi-dimensional rotational FFT generating functions.. Bioinformatics.

[23] Katsura Y, Satta Y (2011). Evolutionary history of the cancer immunity antigen MAGE gene family.. PLoS One.

[24] Miyake S, Sellers WR, Safran M, Li X, Zhao W (2000). Cells degrade a novel inhibitor of differentiation with E1A-like properties upon exiting the cell cycle.. Mol Cell Biol.

[25] Miyake S, Yanagisawa Y, Yuasa Y (2003). A novel EID-1 family member, EID-2, associates with histone deacetylases and inhibits muscle differentiation.. J Biol Chem.

[26] Ji A, Dao D, Chen J, MacLellan WR (2003). EID-2, a novel member of the EID family of p300-binding proteins inhibits transactivation by MyoD.. Gene.

[27] Routzahn KM, Waugh DS (2002). Differential effects of supplementary affinity tags on the solubility of MBP fusion proteins.. J Struct Funct Genomics.

[28] Pospisilova S, Brazda V, Amrichova J, Kamermeierova R, Palecek E (2000). Precise characterisation of monoclonal antibodies to the C-terminal region of p53 protein using the PEPSCAN ELISA technique and a new non-radioactive gel shift assay.. J Immunol Methods.

[29] Jorgensen WL, Chandrasekhar J, Madura JD, Impey RW, Klein ML (1983). Comparison of simple potential functions for simulating liquid water.. Journal of Chemical Physics.

[30] Case DA, Cheatham TE, Darden T, Gohlke H, Luo R (2005). The Amber biomolecular simulation programs.. J Comput Chem.

[31] Ryckaert JP, Ciccotti G, Berendsen HJC (1977). Numerical integration of the cartesian equations of motion of a system with constraints: molecular dynamics of n-alkanes.. Journal of Computational Physics.

[32] Berendsen HJC, Postma JPM, van Gunsteren WF, DiNola A, Haak JR (1984). Molecular dynamics with coupling to an external bath.. The Journal of Chemical Physics.

[33] Humphrey W, Dalke A, Schulten K (1996). VMD: Visual molecular dynamics.. Journal of Molecular Graphics.

