# Supplementary figures and images for: Analysis of the Nse3/MAGE-Binding Domain of the Nse4/EID Family Proteins
Source: PLoS One. 2012 Apr 20;7(4):e35813. doi: 10.1371/journal.pone.0035813 (PMC3335016; doi:10.1371/journal.pone.0035813)

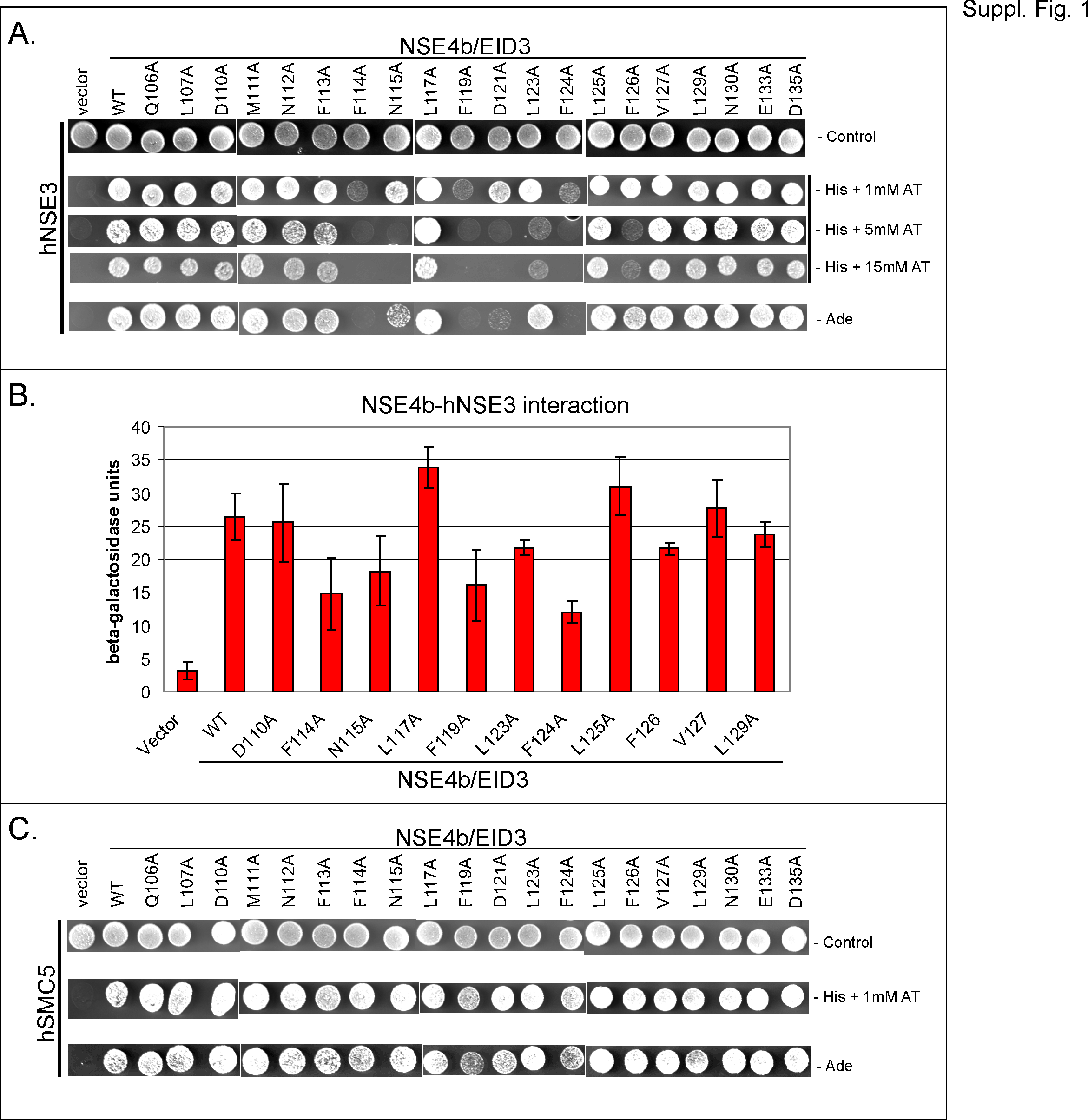

Supplement: Figure S1 — Binding of the hNSE3/MAGEG1 to NSE4b/EID3 protein. Yeast-2-hybrid analysis of the interaction of the indicated mutants of human NSE4b (aa 1 to 333) with hNse3/MAGEG1 (A. and B.) and/or hSMC5 (C.). Interactions result in growth on -Leu, -Trp, -His plates (with or without aminotriazole, AT) and -Leu, -Trp, -Ade plates. Control, plate without Leu and Trp. (B.) Selected NSE4b-hNSE3 pairs were assayed for β-galactosidase activity. (TIF) [file pone.0035813.s001.tif]

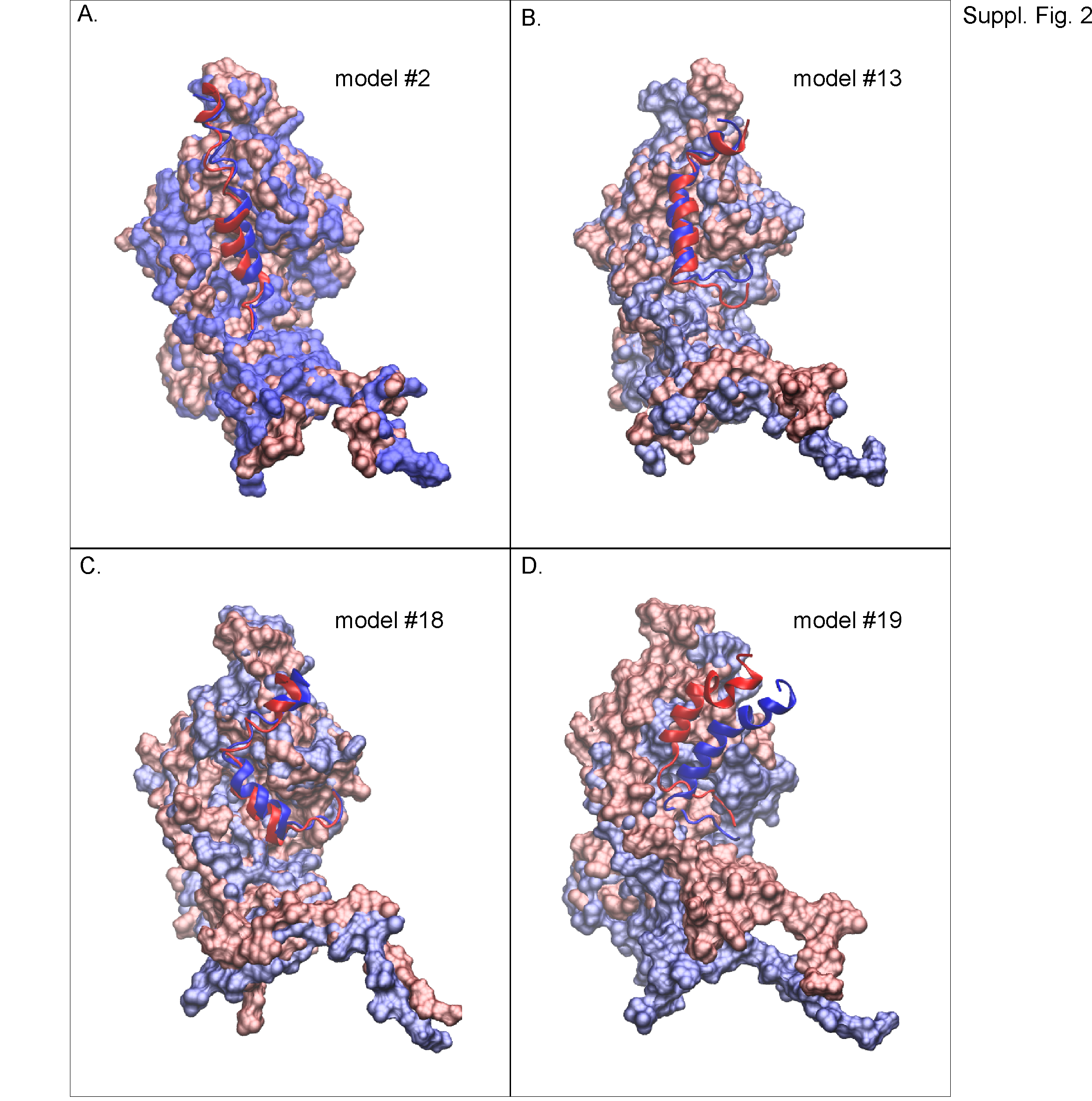

Supplement: Figure S2 — Comparison of the best four scored structures of the EID2-MAGEC2 docking. Superposition of starting structure (blue) and structure after 1 ns molecular dynamics simulations (red). The MAGEC2(129-339) protein is visualized by surface and the EID2 peptide is visualized by ribbon model. The model #18 (panel C) exhibited the lowest free binding energy (Table S1) and the EID2 core region (central helical part) fitted best into the MAGEC2 pocket. (TIF) [file pone.0035813.s002.tif]
