# Supplementary material for: Analysis of the Nse3/MAGE-Binding Domain of the Nse4/EID Family Proteins
Source: PLoS One. 2012 Apr 20;7(4):e35813. doi: 10.1371/journal.pone.0035813 (PMC3335016; doi:10.1371/journal.pone.0035813)
Supplement: Table S1 — Free binding energies calculated from MD trajectories. (DOC) [file pone.0035813.s003.doc]

**Table S1.** Free binding energies calculated from MD trajectories.

| Model number | ΔG [kcal/mol] MM/GBSA | ΔG [kcal/mol] MM/PBSA |
| --- | --- | --- |
| #2 | -6.53 ± 3.89 | -9.03 ± 4.96 |
| #13 | -10.40 ± 2.92 | -16.13 ± 4.91 |
| #18 | -19.99 ± 6.53 | -25.20 ± 6.03 |
| #19 | -12.54 ± 4.06 | -18.34 ± 4.01 |
