# Supplementary material for: Analysis of the Nse3/MAGE-Binding Domain of the Nse4/EID Family Proteins
Source: PLoS One. 2012 Apr 20;7(4):e35813. doi: 10.1371/journal.pone.0035813 (PMC3335016; doi:10.1371/journal.pone.0035813)
Supplement: Table S2 — Primers used for cloning. (DOC) [file pone.0035813.s004.doc]

**Table S2.** Primers used for cloning

| **Construct** | **Primer (5'-/3'-end)** |
| --- | --- |
| pTriEx4-Nse4(aa1-110) | GAC GAC GAC AAG ATG TCC TCC ATT GAT AAA CGG |
|  | GAG GAG AAG CCC GGT TCA TTC TTG GAT TCG AGT AAC AT |
| pTriEx4-Nse4(aa1-77) | GAC GAC GAC AAG ATG CCC ATG GGG ATG TCC TCC ATT GAT AAA CGG |
|  | GAG GAG AAG CCC GGT CTC GAG TCA ATG AAG CTG CCT AGC TTT |
| pET41-Nse4(aa75-104) | GAC GAC GAC AAG ATG CCC ATG GGG CAG CTT CAT ATT GGA AGG |
|  | GAG GAG AAG CCC GGT CTC GAG TCA ATT CGA GTG GGA AGT AGG |
| pET41-NSE4b(aa106-135) | GAC GAC GAC AAG ATG CCC ATG GCA CAG TTA AAC TCA GAT ATG |
|  | GAG GAG AAG CCC GGT CTC GAG CTA ATC GCC TTC CAT CCA ATT C |
| pET41-NSE4b(aa113-135) | GAC GAC GAC AAG ATG CCC ATG GAC TTC TTT AAT CAG TTA GC |
|  | GAG GAG AAG CCC GGT CTC GAG CTA ATC GCC TTC CAT CCA ATT C |
| pET41-NSE4a(aa150-179) | GAC GAC GAC AAG ATG CCC ATG GGG CAG CTG CGC TCA GAC CTG |
|  | GAG GAG AAG CCC GGT CTC GAG TCA TTC AGC TTC TAG CGG ATT |
| pET41-EID1(aa146-177) | GAC GAC GAC AAG ATG CCC ATG GGG TTT CAG ATG CAT TAT GAG |
|  | GAG GAG AAG CCC GGT CTC GAG TCA TTC TTC GGT CAG ACG ATT G |
| pET41-EID2(aa197-225) | GAC GAC GAC AAG ATG GCC ATG GGT CAG CGA AAT CCT CAC AGG |
|  | GAG GAG AAG CCC GGT CTC GAG CTA TTC TAT CAG AGG GTT G |
| pET41-EID2b(aa135-161) | GAC GAC GAC AAG ATG CCC ATG GAT CCC CCG CAG ATG GAC |
|  | GAG GAG AAG CCC GGT CTC GAG TCA GTC GGC CAG AGG ACT |
| pTriEx4-hSMC5(aa4-1101) | GAC GAC GAC AAG ATG GGG ATC CCG AGC AAG AAG ACG TCA AC |
|  | GAG GAG AAG CCC GGT GTC GAC TTA AGA AGG TTG AGT GAA TG |
| pTriEx4-MAGEC2(aa129-339) | GAC GAC GAC AAG ATG CCC ATG GGG CTG CCA GAC AGT GAG TCC TC |
|  | GAG GAG AAG CCC GGT CTC GAG TCA ATC TTT CAA AGC ATC CTT GTA |
