# Supplementary material for: Analysis of the Nse3/MAGE-Binding Domain of the Nse4/EID Family Proteins
Source: PLoS One. 2012 Apr 20;7(4):e35813. doi: 10.1371/journal.pone.0035813 (PMC3335016; doi:10.1371/journal.pone.0035813)
Supplement: Table S3 — Primers used for site-directed mutagenesis of human hNSE4b/EID3. (DOC) [file pone.0035813.s005.doc]

**Table S3.** Primers used for site-directed mutagenesis of human hNSE4b/EID3

| **Mutation** | **Primer (forward / reverse)** |
| --- | --- |
| **Q106A** | GGT AAA GAA AAG GCA AAG gcG TTA AAC TCA GAT ATG AAC |
|  | GTT CAT ATC TGA GTT TAA Cgc CTT TGC CTT TTC TTT ACC |
| **L107A** | AAA GAA AAG GCA AAG CAG gcA AAC TCA GAT ATG AAC TTC |
|  | GAA GTT CAT ATC TGA GTT Tgc CTG CTT TGC CTT TTC TTT |
| **D110A** | GCA AAG CAG TTA AAC TCA GcT ATG AAC TTC TTT AAT CAG |
|  | CTG ATT AAA GAA GTT CAT AgC TGA GTT TAA CTG CTT TGC |
| **M111A** | AAG CAG TTA AAC TCA GAT gcG AAC TTC TTT AAT CAG TTA |
|  | TAA CTG ATT AAA GAA GTT Cgc ATC TGA GTT TAA CTG CTT |
| **N112A** | CAG TTA AAC TCA GAT ATG gcC TTC TTT AAT CAG TTA GCA |
|  | TGC TAA CTG ATT AAA GAA Ggc CAT ATC TGA GTT TAA CTG |
| **F113A** | TTA AAC TCA GAT ATG AAC gcC TTT AAT CAG TTA GCA TTT |
|  | AAA TGC TAA CTG ATT AAA Ggc GTT CAT ATC TGA GTT TAA |
| **F114A** | AAC TCA GAT ATG AAC TTC gcT AAT CAG TTA GCA TTT TGT |
|  | ACA AAA TGC TAA CTG ATT Agc GAA GTT CAT ATC TGA GTT |
| **N115A** | TCA GAT ATG AAC TTC TTT gcT CAG TTA GCA TTT TGT GAC |
|  | GTC ACA AAA TGC TAA CTG Agc AAA GAA GTT CAT ATC TGA |
| **L117A** | ATG AAC TTC TTT AAT CAG gcA GCA TTT TGT GAC TTT CTG |
|  | CAG AAA GTC ACA AAA TGC Tgc CTG ATT AAA GAA GTT CAT |
| **F119A** | TTC TTT AAT CAG TTA GCA gcT TGT GAC TTT CTG TTT CTG |
|  | CAG AAA CAG AAA GTC ACA Agc TGC TAA CTG ATT AAA GAA |
| **D121A** | AAT CAG TTA GCA TTT TGT GcC TTT CTG TTT CTG TTC GTG |
|  | CAC GAA CAG AAA CAG AAA GgC ACA AAA TGC TAA CTG ATT |
| **L123A** | TTA GCA TTT TGT GAC TTT gcG TTT CTG TTC GTG GGT CTG |
|  | CAG ACC CAC GAA CAG AAA Cgc AAA GTC ACA AAA TGC TAA |
| **F124A** | GCA TTT TGT GAC TTT CTG gcT CTG TTC GTG GGT CTG AAT |
|  | ATT CAG ACC CAC GAA CAG Agc CAG AAA GTC ACA AAA TGC |
| **L125A** | TTT TGT GAC TTT CTG TTT gcG TTC GTG GGT CTG AAT TGG |
|  | CCA ATT CAG ACC CAC GAA Cgc AAA CAG AAA GTC ACA AAA |
| **F126A** | TGT GAC TTT CTG TTT CTG gcC GTG GGT CTG AAT TGG ATG |
|  | CAT CCA ATT CAG ACC CAC Ggc CAG AAA CAG AAA GTC ACA |
| **V127A** | GAC TTT CTG TTT CTG TTC GcG GGT CTG AAT TGG ATG GAA |
|  | TTC CAT CCA ATT CAG ACC CcC GAA CAG AAA CAG AAA GTC |
| **L129A** | TTA AAC TCA GAT ATG AAC gcC TTT AAT CAG TTA GCA TTT |
|  | AAA TGC TAA CTG ATT AAA Ggc GTT CAT ATC TGA GTT TAA |
| **N130A** | TTT CTG TTC GTG GGT CTG gcT TGG ATG GAA GGC GAT CCT |
|  | AGG ATC GCC TTC CAT CCA Agc CAG ACC CAC GAA CAG AAA |
| **E133A** | GTG GGT CTG AAT TGG ATG GcA GGC GAT CCT GAC AAG TTG |
|  | CAA CTT GTC AGG ATC GCC TgC CAT CCA ATT CAG ACC CAC |
| **D135A** | CTG AAT TGG ATG GAA GGC GcT CCT GAC AAG TTG AGT GAT |
|  | ATC ACT CAA CTT GTC AGG AgC GCC TTC CAT CCA ATT CAG |
