# Supplementary material for: Analysis of the Nse3/MAGE-Binding Domain of the Nse4/EID Family Proteins
Source: PLoS One. 2012 Apr 20;7(4):e35813. doi: 10.1371/journal.pone.0035813 (PMC3335016; doi:10.1371/journal.pone.0035813)
Supplement: Table S4 — The MAGEC2(133-336)-EID2(197-225) atom coordinates (PDB file). (DOC) [file pone.0035813.s006.doc]

**Table S4.** The MAGEC2(133-336)-EID2(197-225) atom coordinates (PDB file).

REMARK

REMARK MAGEC2 (aa133-336)

REMARK EID2 (aa197-225)

REMARK Docking and MD simulations

REMARK Author: Zdenek Kriz, Dr.

ATOM 174 N SER 14 65.043 59.048 79.353 1.00 0.00 N

ATOM 175 H SER 14 65.405 59.701 80.032 1.00 0.00 H

ATOM 176 CA SER 14 64.352 57.890 79.946 1.00 0.00 C

ATOM 177 HA SER 14 64.749 56.930 79.615 1.00 0.00 H

ATOM 178 CB SER 14 64.471 57.764 81.478 1.00 0.00 C

ATOM 179 HB2 SER 14 64.274 58.757 81.880 1.00 0.00 H

ATOM 180 HB3 SER 14 63.751 57.023 81.826 1.00 0.00 H

ATOM 181 OG SER 14 65.832 57.378 81.844 1.00 0.00 O

ATOM 182 HG SER 14 66.034 57.966 82.575 1.00 0.00 H

ATOM 183 C SER 14 62.844 57.941 79.529 1.00 0.00 C

ATOM 184 O SER 14 62.346 57.018 78.869 1.00 0.00 O

ATOM 185 N GLU 15 62.165 59.122 79.739 1.00 0.00 N

ATOM 186 H GLU 15 62.707 59.941 79.972 1.00 0.00 H

ATOM 187 CA GLU 15 60.770 59.340 79.233 1.00 0.00 C

ATOM 188 HA GLU 15 60.075 58.628 79.680 1.00 0.00 H

ATOM 189 CB GLU 15 60.333 60.806 79.434 1.00 0.00 C

ATOM 190 HB2 GLU 15 61.176 61.445 79.170 1.00 0.00 H

ATOM 191 HB3 GLU 15 59.456 60.915 78.796 1.00 0.00 H

ATOM 192 CG GLU 15 59.946 60.922 80.940 1.00 0.00 C

ATOM 193 HG2 GLU 15 59.180 60.182 81.172 1.00 0.00 H

ATOM 194 HG3 GLU 15 60.813 60.654 81.545 1.00 0.00 H

ATOM 195 CD GLU 15 59.541 62.302 81.379 1.00 0.00 C

ATOM 196 OE1 GLU 15 60.366 63.182 81.497 1.00 0.00 O

ATOM 197 OE2 GLU 15 58.330 62.477 81.771 1.00 0.00 O

ATOM 198 C GLU 15 60.588 59.062 77.730 1.00 0.00 C

ATOM 199 O GLU 15 59.780 58.218 77.359 1.00 0.00 O

ATOM 200 N SER 16 61.487 59.617 76.924 1.00 0.00 N

ATOM 201 H SER 16 62.042 60.348 77.344 1.00 0.00 H

ATOM 202 CA SER 16 61.648 59.454 75.438 1.00 0.00 C

ATOM 203 HA SER 16 60.668 59.716 75.039 1.00 0.00 H

ATOM 204 CB SER 16 62.794 60.268 74.808 1.00 0.00 C

ATOM 205 HB2 SER 16 63.795 59.987 75.137 1.00 0.00 H

ATOM 206 HB3 SER 16 62.732 60.045 73.743 1.00 0.00 H

ATOM 207 OG SER 16 62.482 61.605 75.051 1.00 0.00 O

ATOM 208 HG SER 16 62.651 61.797 75.976 1.00 0.00 H

ATOM 209 C SER 16 61.870 57.965 75.190 1.00 0.00 C

ATOM 210 O SER 16 61.099 57.322 74.466 1.00 0.00 O

ATOM 211 N SER 17 62.787 57.252 75.876 1.00 0.00 N

ATOM 212 H SER 17 63.407 57.714 76.526 1.00 0.00 H

ATOM 213 CA SER 17 62.987 55.843 75.699 1.00 0.00 C

ATOM 214 HA SER 17 63.276 55.664 74.664 1.00 0.00 H

ATOM 215 CB SER 17 64.066 55.270 76.543 1.00 0.00 C

ATOM 216 HB2 SER 17 63.667 55.194 77.555 1.00 0.00 H

ATOM 217 HB3 SER 17 64.339 54.285 76.164 1.00 0.00 H

ATOM 218 OG SER 17 65.198 56.003 76.567 1.00 0.00 O

ATOM 219 HG SER 17 65.037 56.832 77.024 1.00 0.00 H

ATOM 220 C SER 17 61.781 54.967 75.923 1.00 0.00 C

ATOM 221 O SER 17 61.749 53.828 75.461 1.00 0.00 O

ATOM 222 N PHE 18 60.739 55.369 76.600 1.00 0.00 N

ATOM 223 H PHE 18 60.812 56.327 76.911 1.00 0.00 H

ATOM 224 CA PHE 18 59.506 54.578 76.786 1.00 0.00 C

ATOM 225 HA PHE 18 59.695 53.520 76.607 1.00 0.00 H

ATOM 226 CB PHE 18 58.990 54.658 78.233 1.00 0.00 C

ATOM 227 HB2 PHE 18 59.782 54.186 78.814 1.00 0.00 H

ATOM 228 HB3 PHE 18 59.005 55.693 78.575 1.00 0.00 H

ATOM 229 CG PHE 18 57.716 54.001 78.525 1.00 0.00 C

ATOM 230 CD1 PHE 18 57.586 52.689 78.602 1.00 0.00 C

ATOM 231 HD1 PHE 18 58.480 52.121 78.391 1.00 0.00 H

ATOM 232 CE1 PHE 18 56.359 52.048 78.970 1.00 0.00 C

ATOM 233 HE1 PHE 18 56.346 50.989 79.184 1.00 0.00 H

ATOM 234 CZ PHE 18 55.260 52.867 79.303 1.00 0.00 C

ATOM 235 HZ PHE 18 54.325 52.417 79.603 1.00 0.00 H

ATOM 236 CE2 PHE 18 55.433 54.281 79.357 1.00 0.00 C

ATOM 237 HE2 PHE 18 54.670 54.872 79.840 1.00 0.00 H

ATOM 238 CD2 PHE 18 56.619 54.855 78.887 1.00 0.00 C

ATOM 239 HD2 PHE 18 56.742 55.925 78.974 1.00 0.00 H

ATOM 240 C PHE 18 58.471 54.974 75.694 1.00 0.00 C

ATOM 241 O PHE 18 57.642 54.108 75.274 1.00 0.00 O

ATOM 242 N THR 19 58.470 56.239 75.238 1.00 0.00 N

ATOM 243 H THR 19 59.094 56.955 75.581 1.00 0.00 H

ATOM 244 CA THR 19 57.728 56.513 74.017 1.00 0.00 C

ATOM 245 HA THR 19 56.715 56.226 74.298 1.00 0.00 H

ATOM 246 CB THR 19 57.819 57.981 73.604 1.00 0.00 C

ATOM 247 HB THR 19 58.791 58.288 73.217 1.00 0.00 H

ATOM 248 CG2 THR 19 56.660 58.418 72.718 1.00 0.00 C

ATOM 249 HG21 THR 19 56.478 57.599 72.022 1.00 0.00 H

ATOM 250 HG22 THR 19 55.722 58.718 73.185 1.00 0.00 H

ATOM 251 HG23 THR 19 57.035 59.258 72.133 1.00 0.00 H

ATOM 252 OG1 THR 19 57.746 58.794 74.733 1.00 0.00 O

ATOM 253 HG1 THR 19 57.742 59.679 74.361 1.00 0.00 H

ATOM 254 C THR 19 58.266 55.556 72.908 1.00 0.00 C

ATOM 255 O THR 19 57.605 54.743 72.389 1.00 0.00 O

ATOM 256 N TYR 20 59.627 55.489 72.799 1.00 0.00 N

ATOM 257 H TYR 20 60.251 55.903 73.477 1.00 0.00 H

ATOM 258 CA TYR 20 60.382 54.601 71.823 1.00 0.00 C

ATOM 259 HA TYR 20 60.078 54.761 70.789 1.00 0.00 H

ATOM 260 CB TYR 20 61.907 54.898 71.924 1.00 0.00 C

ATOM 261 HB2 TYR 20 61.982 55.976 71.781 1.00 0.00 H

ATOM 262 HB3 TYR 20 62.276 54.614 72.910 1.00 0.00 H

ATOM 263 CG TYR 20 62.748 54.203 70.775 1.00 0.00 C

ATOM 264 CD1 TYR 20 63.512 53.005 71.013 1.00 0.00 C

ATOM 265 HD1 TYR 20 63.406 52.521 71.973 1.00 0.00 H

ATOM 266 CE1 TYR 20 64.392 52.374 70.162 1.00 0.00 C

ATOM 267 HE1 TYR 20 64.863 51.432 70.403 1.00 0.00 H

ATOM 268 CZ TYR 20 64.683 53.069 68.956 1.00 0.00 C

ATOM 269 OH TYR 20 65.473 52.478 67.976 1.00 0.00 O

ATOM 270 HH TYR 20 65.616 51.546 68.156 1.00 0.00 H

ATOM 271 CE2 TYR 20 64.057 54.293 68.749 1.00 0.00 C

ATOM 272 HE2 TYR 20 64.325 54.806 67.837 1.00 0.00 H

ATOM 273 CD2 TYR 20 63.148 54.850 69.636 1.00 0.00 C

ATOM 274 HD2 TYR 20 62.798 55.857 69.468 1.00 0.00 H

ATOM 275 C TYR 20 60.031 53.158 72.060 1.00 0.00 C

ATOM 276 O TYR 20 59.613 52.574 71.129 1.00 0.00 O

ATOM 277 N THR 21 60.164 52.650 73.254 1.00 0.00 N

ATOM 278 H THR 21 60.376 53.196 74.077 1.00 0.00 H

ATOM 279 CA THR 21 60.087 51.140 73.511 1.00 0.00 C

ATOM 280 HA THR 21 60.613 50.657 72.688 1.00 0.00 H

ATOM 281 CB THR 21 60.817 50.670 74.739 1.00 0.00 C

ATOM 282 HB THR 21 60.564 49.616 74.856 1.00 0.00 H

ATOM 283 CG2 THR 21 62.376 50.780 74.451 1.00 0.00 C

ATOM 284 HG21 THR 21 62.733 49.789 74.170 1.00 0.00 H

ATOM 285 HG22 THR 21 62.562 51.528 73.680 1.00 0.00 H

ATOM 286 HG23 THR 21 62.765 51.247 75.356 1.00 0.00 H

ATOM 287 OG1 THR 21 60.551 51.425 75.884 1.00 0.00 O

ATOM 288 HG1 THR 21 61.043 52.238 75.750 1.00 0.00 H

ATOM 289 C THR 21 58.682 50.606 73.473 1.00 0.00 C

ATOM 290 O THR 21 58.550 49.363 73.398 1.00 0.00 O

ATOM 291 N LEU 22 57.655 51.416 73.341 1.00 0.00 N

ATOM 292 H LEU 22 57.713 52.424 73.362 1.00 0.00 H

ATOM 293 CA LEU 22 56.295 51.027 73.015 1.00 0.00 C

ATOM 294 HA LEU 22 56.122 49.978 73.254 1.00 0.00 H

ATOM 295 CB LEU 22 55.216 51.885 73.734 1.00 0.00 C

ATOM 296 HB2 LEU 22 55.535 52.927 73.711 1.00 0.00 H

ATOM 297 HB3 LEU 22 54.238 51.714 73.283 1.00 0.00 H

ATOM 298 CG LEU 22 55.177 51.594 75.220 1.00 0.00 C

ATOM 299 HG LEU 22 56.171 51.600 75.667 1.00 0.00 H

ATOM 300 CD1 LEU 22 54.448 52.653 76.044 1.00 0.00 C

ATOM 301 HD11 LEU 22 54.938 53.616 75.904 1.00 0.00 H

ATOM 302 HD12 LEU 22 53.414 52.650 75.699 1.00 0.00 H

ATOM 303 HD13 LEU 22 54.370 52.436 77.110 1.00 0.00 H

ATOM 304 CD2 LEU 22 54.496 50.259 75.485 1.00 0.00 C

ATOM 305 HD21 LEU 22 54.952 49.435 74.937 1.00 0.00 H

ATOM 306 HD22 LEU 22 54.590 50.065 76.554 1.00 0.00 H

ATOM 307 HD23 LEU 22 53.426 50.265 75.276 1.00 0.00 H

ATOM 308 C LEU 22 56.210 51.001 71.501 1.00 0.00 C

ATOM 309 O LEU 22 55.658 50.072 70.864 1.00 0.00 O

ATOM 310 N ASP 23 56.645 52.107 70.853 1.00 0.00 N

ATOM 311 H ASP 23 57.129 52.759 71.454 1.00 0.00 H

ATOM 312 CA ASP 23 56.459 52.405 69.449 1.00 0.00 C

ATOM 313 HA ASP 23 55.399 52.343 69.200 1.00 0.00 H

ATOM 314 CB ASP 23 56.809 53.851 69.247 1.00 0.00 C

ATOM 315 HB2 ASP 23 56.124 54.379 69.910 1.00 0.00 H

ATOM 316 HB3 ASP 23 57.850 54.014 69.528 1.00 0.00 H

ATOM 317 CG ASP 23 56.530 54.272 67.855 1.00 0.00 C

ATOM 318 OD1 ASP 23 55.374 54.217 67.366 1.00 0.00 O

ATOM 319 OD2 ASP 23 57.518 54.539 67.068 1.00 0.00 O

ATOM 320 C ASP 23 57.249 51.450 68.497 1.00 0.00 C

ATOM 321 O ASP 23 56.844 51.085 67.420 1.00 0.00 O

ATOM 322 N GLU 24 58.366 50.937 68.975 1.00 0.00 N

ATOM 323 H GLU 24 58.740 51.273 69.851 1.00 0.00 H

ATOM 324 CA GLU 24 59.076 49.844 68.348 1.00 0.00 C

ATOM 325 HA GLU 24 59.457 50.204 67.392 1.00 0.00 H

ATOM 326 CB GLU 24 60.339 49.529 69.255 1.00 0.00 C

ATOM 327 HB2 GLU 24 60.040 49.485 70.302 1.00 0.00 H

ATOM 328 HB3 GLU 24 60.795 48.560 69.051 1.00 0.00 H

ATOM 329 CG GLU 24 61.455 50.554 69.252 1.00 0.00 C

ATOM 330 HG2 GLU 24 60.967 51.528 69.216 1.00 0.00 H

ATOM 331 HG3 GLU 24 62.122 50.547 70.115 1.00 0.00 H

ATOM 332 CD GLU 24 62.340 50.300 68.079 1.00 0.00 C

ATOM 333 OE1 GLU 24 61.927 50.678 66.996 1.00 0.00 O

ATOM 334 OE2 GLU 24 63.298 49.501 68.217 1.00 0.00 O

ATOM 335 C GLU 24 58.262 48.554 68.144 1.00 0.00 C

ATOM 336 O GLU 24 58.359 47.783 67.129 1.00 0.00 O

ATOM 337 N LYS 25 57.355 48.351 69.138 1.00 0.00 N

ATOM 338 H LYS 25 57.345 49.038 69.878 1.00 0.00 H

ATOM 339 CA LYS 25 56.440 47.155 69.191 1.00 0.00 C

ATOM 340 HA LYS 25 57.005 46.343 68.731 1.00 0.00 H

ATOM 341 CB LYS 25 56.171 46.654 70.609 1.00 0.00 C

ATOM 342 HB2 LYS 25 55.607 47.432 71.123 1.00 0.00 H

ATOM 343 HB3 LYS 25 55.588 45.733 70.599 1.00 0.00 H

ATOM 344 CG LYS 25 57.491 46.350 71.399 1.00 0.00 C

ATOM 345 HG2 LYS 25 57.823 45.341 71.154 1.00 0.00 H

ATOM 346 HG3 LYS 25 58.325 47.013 71.170 1.00 0.00 H

ATOM 347 CD LYS 25 57.131 46.402 72.875 1.00 0.00 C

ATOM 348 HD2 LYS 25 56.947 47.391 73.294 1.00 0.00 H

ATOM 349 HD3 LYS 25 56.207 45.865 73.088 1.00 0.00 H

ATOM 350 CE LYS 25 58.159 45.744 73.803 1.00 0.00 C

ATOM 351 HE2 LYS 25 57.607 45.739 74.743 1.00 0.00 H

ATOM 352 HE3 LYS 25 58.238 44.717 73.445 1.00 0.00 H

ATOM 353 NZ LYS 25 59.432 46.532 73.906 1.00 0.00 N

ATOM 354 HZ1 LYS 25 60.052 46.261 74.656 1.00 0.00 H

ATOM 355 HZ2 LYS 25 59.912 46.509 73.018 1.00 0.00 H

ATOM 356 HZ3 LYS 25 59.238 47.515 74.034 1.00 0.00 H

ATOM 357 C LYS 25 55.227 47.308 68.301 1.00 0.00 C

ATOM 358 O LYS 25 54.748 46.348 67.740 1.00 0.00 O

ATOM 359 N VAL 26 54.835 48.558 67.972 1.00 0.00 N

ATOM 360 H VAL 26 55.231 49.348 68.462 1.00 0.00 H

ATOM 361 CA VAL 26 53.861 48.878 66.916 1.00 0.00 C

ATOM 362 HA VAL 26 52.994 48.256 67.135 1.00 0.00 H

ATOM 363 CB VAL 26 53.470 50.424 67.010 1.00 0.00 C

ATOM 364 HB VAL 26 54.335 51.076 66.886 1.00 0.00 H

ATOM 365 CG1 VAL 26 52.478 50.580 65.893 1.00 0.00 C

ATOM 366 HG11 VAL 26 51.703 49.826 66.034 1.00 0.00 H

ATOM 367 HG12 VAL 26 52.093 51.599 65.937 1.00 0.00 H

ATOM 368 HG13 VAL 26 52.994 50.446 64.942 1.00 0.00 H

ATOM 369 CG2 VAL 26 52.830 50.923 68.299 1.00 0.00 C

ATOM 370 HG21 VAL 26 52.552 51.962 68.123 1.00 0.00 H

ATOM 371 HG22 VAL 26 52.018 50.225 68.501 1.00 0.00 H

ATOM 372 HG23 VAL 26 53.555 50.736 69.091 1.00 0.00 H

ATOM 373 C VAL 26 54.428 48.483 65.573 1.00 0.00 C

ATOM 374 O VAL 26 53.781 47.840 64.765 1.00 0.00 O

ATOM 375 N ALA 27 55.702 48.861 65.426 1.00 0.00 N

ATOM 376 H ALA 27 56.263 49.316 66.132 1.00 0.00 H

ATOM 377 CA ALA 27 56.313 48.663 64.065 1.00 0.00 C

ATOM 378 HA ALA 27 55.663 49.054 63.283 1.00 0.00 H

ATOM 379 CB ALA 27 57.627 49.526 63.924 1.00 0.00 C

ATOM 380 HB1 ALA 27 58.186 49.231 64.812 1.00 0.00 H

ATOM 381 HB2 ALA 27 58.160 49.175 63.040 1.00 0.00 H

ATOM 382 HB3 ALA 27 57.440 50.600 63.942 1.00 0.00 H

ATOM 383 C ALA 27 56.695 47.178 63.822 1.00 0.00 C

ATOM 384 O ALA 27 56.264 46.619 62.776 1.00 0.00 O

ATOM 385 N GLU 28 57.251 46.472 64.848 1.00 0.00 N

ATOM 386 H GLU 28 57.609 47.043 65.600 1.00 0.00 H

ATOM 387 CA GLU 28 57.384 44.967 64.758 1.00 0.00 C

ATOM 388 HA GLU 28 57.964 44.695 63.876 1.00 0.00 H

ATOM 389 CB GLU 28 58.164 44.497 66.031 1.00 0.00 C

ATOM 390 HB2 GLU 28 59.125 45.010 65.977 1.00 0.00 H

ATOM 391 HB3 GLU 28 57.570 44.772 66.902 1.00 0.00 H

ATOM 392 CG GLU 28 58.467 43.021 66.206 1.00 0.00 C

ATOM 393 HG2 GLU 28 57.468 42.597 66.308 1.00 0.00 H

ATOM 394 HG3 GLU 28 59.030 42.570 65.389 1.00 0.00 H

ATOM 395 CD GLU 28 59.136 42.667 67.572 1.00 0.00 C

ATOM 396 OE1 GLU 28 58.872 43.392 68.580 1.00 0.00 O

ATOM 397 OE2 GLU 28 59.949 41.684 67.582 1.00 0.00 O

ATOM 398 C GLU 28 56.148 44.207 64.625 1.00 0.00 C

ATOM 399 O GLU 28 56.081 43.076 64.145 1.00 0.00 O

ATOM 400 N LEU 29 55.036 44.740 65.089 1.00 0.00 N

ATOM 401 H LEU 29 55.218 45.572 65.632 1.00 0.00 H

ATOM 402 CA LEU 29 53.700 44.105 64.945 1.00 0.00 C

ATOM 403 HA LEU 29 53.849 43.049 65.169 1.00 0.00 H

ATOM 404 CB LEU 29 52.734 44.745 65.944 1.00 0.00 C

ATOM 405 HB2 LEU 29 53.199 44.730 66.930 1.00 0.00 H

ATOM 406 HB3 LEU 29 52.610 45.792 65.667 1.00 0.00 H

ATOM 407 CG LEU 29 51.383 44.142 66.149 1.00 0.00 C

ATOM 408 HG LEU 29 50.798 44.271 65.238 1.00 0.00 H

ATOM 409 CD1 LEU 29 51.264 42.709 66.383 1.00 0.00 C

ATOM 410 HD11 LEU 29 51.622 42.104 65.550 1.00 0.00 H

ATOM 411 HD12 LEU 29 51.776 42.340 67.271 1.00 0.00 H

ATOM 412 HD13 LEU 29 50.200 42.485 66.459 1.00 0.00 H

ATOM 413 CD2 LEU 29 50.574 44.746 67.221 1.00 0.00 C

ATOM 414 HD21 LEU 29 49.540 44.403 67.182 1.00 0.00 H

ATOM 415 HD22 LEU 29 50.901 44.623 68.254 1.00 0.00 H

ATOM 416 HD23 LEU 29 50.640 45.816 67.020 1.00 0.00 H

ATOM 417 C LEU 29 53.186 44.391 63.490 1.00 0.00 C

ATOM 418 O LEU 29 52.628 43.445 62.934 1.00 0.00 O

ATOM 419 N VAL 30 53.346 45.617 62.931 1.00 0.00 N

ATOM 420 H VAL 30 53.835 46.353 63.419 1.00 0.00 H

ATOM 421 CA VAL 30 53.143 45.785 61.483 1.00 0.00 C

ATOM 422 HA VAL 30 52.101 45.646 61.193 1.00 0.00 H

ATOM 423 CB VAL 30 53.358 47.310 61.247 1.00 0.00 C

ATOM 424 HB VAL 30 54.220 47.709 61.781 1.00 0.00 H

ATOM 425 CG1 VAL 30 53.481 47.752 59.756 1.00 0.00 C

ATOM 426 HG11 VAL 30 54.441 47.374 59.404 1.00 0.00 H

ATOM 427 HG12 VAL 30 52.603 47.316 59.277 1.00 0.00 H

ATOM 428 HG13 VAL 30 53.369 48.835 59.796 1.00 0.00 H

ATOM 429 CG2 VAL 30 52.243 48.158 61.774 1.00 0.00 C

ATOM 430 HG21 VAL 30 51.979 47.907 62.801 1.00 0.00 H

ATOM 431 HG22 VAL 30 52.591 49.190 61.782 1.00 0.00 H

ATOM 432 HG23 VAL 30 51.370 48.076 61.126 1.00 0.00 H

ATOM 433 C VAL 30 53.938 44.832 60.655 1.00 0.00 C

ATOM 434 O VAL 30 53.499 44.288 59.650 1.00 0.00 O

ATOM 435 N GLU 31 55.181 44.535 61.068 1.00 0.00 N

ATOM 436 H GLU 31 55.597 45.195 61.708 1.00 0.00 H

ATOM 437 CA GLU 31 56.016 43.495 60.462 1.00 0.00 C

ATOM 438 HA GLU 31 56.210 43.724 59.415 1.00 0.00 H

ATOM 439 CB GLU 31 57.462 43.527 61.090 1.00 0.00 C

ATOM 440 HB2 GLU 31 57.871 44.535 61.020 1.00 0.00 H

ATOM 441 HB3 GLU 31 57.406 43.299 62.155 1.00 0.00 H

ATOM 442 CG GLU 31 58.511 42.604 60.537 1.00 0.00 C

ATOM 443 HG2 GLU 31 58.033 41.694 60.176 1.00 0.00 H

ATOM 444 HG3 GLU 31 58.913 43.069 59.636 1.00 0.00 H

ATOM 445 CD GLU 31 59.750 42.359 61.386 1.00 0.00 C

ATOM 446 OE1 GLU 31 60.809 42.207 60.680 1.00 0.00 O

ATOM 447 OE2 GLU 31 59.664 41.997 62.599 1.00 0.00 O

ATOM 448 C GLU 31 55.456 41.998 60.463 1.00 0.00 C

ATOM 449 O GLU 31 55.743 41.276 59.522 1.00 0.00 O

ATOM 450 N PHE 32 54.663 41.664 61.441 1.00 0.00 N

ATOM 451 H PHE 32 54.478 42.361 62.149 1.00 0.00 H

ATOM 452 CA PHE 32 53.824 40.403 61.552 1.00 0.00 C

ATOM 453 HA PHE 32 54.472 39.624 61.150 1.00 0.00 H

ATOM 454 CB PHE 32 53.625 40.020 63.066 1.00 0.00 C

ATOM 455 HB2 PHE 32 54.562 39.877 63.606 1.00 0.00 H

ATOM 456 HB3 PHE 32 53.128 40.826 63.605 1.00 0.00 H

ATOM 457 CG PHE 32 52.855 38.668 63.313 1.00 0.00 C

ATOM 458 CD1 PHE 32 53.350 37.532 62.660 1.00 0.00 C

ATOM 459 HD1 PHE 32 54.205 37.597 62.004 1.00 0.00 H

ATOM 460 CE1 PHE 32 52.683 36.301 62.826 1.00 0.00 C

ATOM 461 HE1 PHE 32 53.119 35.446 62.331 1.00 0.00 H

ATOM 462 CZ PHE 32 51.452 36.189 63.519 1.00 0.00 C

ATOM 463 HZ PHE 32 50.991 35.216 63.605 1.00 0.00 H

ATOM 464 CE2 PHE 32 50.893 37.386 64.096 1.00 0.00 C

ATOM 465 HE2 PHE 32 49.972 37.312 64.656 1.00 0.00 H

ATOM 466 CD2 PHE 32 51.497 38.654 63.904 1.00 0.00 C

ATOM 467 HD2 PHE 32 51.083 39.535 64.373 1.00 0.00 H

ATOM 468 C PHE 32 52.437 40.488 60.822 1.00 0.00 C

ATOM 469 O PHE 32 52.082 39.509 60.172 1.00 0.00 O

ATOM 470 N LEU 33 51.789 41.687 60.768 1.00 0.00 N

ATOM 471 H LEU 33 52.007 42.367 61.482 1.00 0.00 H

ATOM 472 CA LEU 33 50.688 41.901 59.846 1.00 0.00 C

ATOM 473 HA LEU 33 49.990 41.070 59.948 1.00 0.00 H

ATOM 474 CB LEU 33 49.984 43.294 60.177 1.00 0.00 C

ATOM 475 HB2 LEU 33 50.634 44.155 60.020 1.00 0.00 H

ATOM 476 HB3 LEU 33 49.192 43.450 59.445 1.00 0.00 H

ATOM 477 CG LEU 33 49.388 43.440 61.594 1.00 0.00 C

ATOM 478 HG LEU 33 50.057 43.082 62.376 1.00 0.00 H

ATOM 479 CD1 LEU 33 49.042 44.980 61.865 1.00 0.00 C

ATOM 480 HD11 LEU 33 48.783 44.934 62.923 1.00 0.00 H

ATOM 481 HD12 LEU 33 49.905 45.620 61.686 1.00 0.00 H

ATOM 482 HD13 LEU 33 48.194 45.381 61.310 1.00 0.00 H

ATOM 483 CD2 LEU 33 48.158 42.603 61.864 1.00 0.00 C

ATOM 484 HD21 LEU 33 48.314 41.538 61.691 1.00 0.00 H

ATOM 485 HD22 LEU 33 47.872 42.709 62.910 1.00 0.00 H

ATOM 486 HD23 LEU 33 47.411 42.957 61.153 1.00 0.00 H

ATOM 487 C LEU 33 51.052 41.805 58.334 1.00 0.00 C

ATOM 488 O LEU 33 50.406 41.044 57.600 1.00 0.00 O

ATOM 489 N LEU 34 52.206 42.344 57.937 1.00 0.00 N

ATOM 490 H LEU 34 52.732 42.942 58.558 1.00 0.00 H

ATOM 491 CA LEU 34 52.764 42.179 56.570 1.00 0.00 C

ATOM 492 HA LEU 34 51.968 42.441 55.874 1.00 0.00 H

ATOM 493 CB LEU 34 53.946 43.137 56.330 1.00 0.00 C

ATOM 494 HB2 LEU 34 54.663 42.978 57.135 1.00 0.00 H

ATOM 495 HB3 LEU 34 54.384 42.904 55.360 1.00 0.00 H

ATOM 496 CG LEU 34 53.427 44.545 56.140 1.00 0.00 C

ATOM 497 HG LEU 34 52.773 44.823 56.967 1.00 0.00 H

ATOM 498 CD1 LEU 34 54.617 45.482 56.276 1.00 0.00 C

ATOM 499 HD11 LEU 34 55.351 45.207 55.518 1.00 0.00 H

ATOM 500 HD12 LEU 34 54.413 46.544 56.413 1.00 0.00 H

ATOM 501 HD13 LEU 34 55.013 45.233 57.260 1.00 0.00 H

ATOM 502 CD2 LEU 34 52.788 44.729 54.755 1.00 0.00 C

ATOM 503 HD21 LEU 34 52.411 45.750 54.701 1.00 0.00 H

ATOM 504 HD22 LEU 34 53.378 44.424 53.891 1.00 0.00 H

ATOM 505 HD23 LEU 34 51.782 44.309 54.741 1.00 0.00 H

ATOM 506 C LEU 34 53.191 40.688 56.284 1.00 0.00 C

ATOM 507 O LEU 34 52.952 40.241 55.186 1.00 0.00 O

ATOM 508 N LEU 35 53.672 39.956 57.315 1.00 0.00 N

ATOM 509 H LEU 35 53.732 40.459 58.189 1.00 0.00 H

ATOM 510 CA LEU 35 54.161 38.594 57.215 1.00 0.00 C

ATOM 511 HA LEU 35 54.780 38.517 56.321 1.00 0.00 H

ATOM 512 CB LEU 35 55.029 38.164 58.402 1.00 0.00 C

ATOM 513 HB2 LEU 35 55.962 38.715 58.519 1.00 0.00 H

ATOM 514 HB3 LEU 35 54.378 38.425 59.238 1.00 0.00 H

ATOM 515 CG LEU 35 55.271 36.683 58.549 1.00 0.00 C

ATOM 516 HG LEU 35 55.405 36.186 57.587 1.00 0.00 H

ATOM 517 CD1 LEU 35 56.577 36.446 59.365 1.00 0.00 C

ATOM 518 HD11 LEU 35 56.813 35.382 59.365 1.00 0.00 H

ATOM 519 HD12 LEU 35 57.338 36.957 58.775 1.00 0.00 H

ATOM 520 HD13 LEU 35 56.558 36.937 60.338 1.00 0.00 H

ATOM 521 CD2 LEU 35 54.239 35.930 59.440 1.00 0.00 C

ATOM 522 HD21 LEU 35 54.557 34.903 59.617 1.00 0.00 H

ATOM 523 HD22 LEU 35 54.009 36.437 60.378 1.00 0.00 H

ATOM 524 HD23 LEU 35 53.357 35.720 58.835 1.00 0.00 H

ATOM 525 C LEU 35 52.982 37.610 56.989 1.00 0.00 C

ATOM 526 O LEU 35 53.006 36.776 56.092 1.00 0.00 O

ATOM 527 N LYS 36 51.925 37.757 57.832 1.00 0.00 N

ATOM 528 H LYS 36 52.163 38.382 58.590 1.00 0.00 H

ATOM 529 CA LYS 36 50.647 37.117 57.704 1.00 0.00 C

ATOM 530 HA LYS 36 50.841 36.050 57.591 1.00 0.00 H

ATOM 531 CB LYS 36 49.678 37.582 58.827 1.00 0.00 C

ATOM 532 HB2 LYS 36 49.958 38.620 59.007 1.00 0.00 H

ATOM 533 HB3 LYS 36 48.652 37.490 58.472 1.00 0.00 H

ATOM 534 CG LYS 36 49.957 36.755 60.047 1.00 0.00 C

ATOM 535 HG2 LYS 36 51.045 36.696 60.068 1.00 0.00 H

ATOM 536 HG3 LYS 36 49.600 37.266 60.940 1.00 0.00 H

ATOM 537 CD LYS 36 49.248 35.389 59.933 1.00 0.00 C

ATOM 538 HD2 LYS 36 48.172 35.500 59.795 1.00 0.00 H

ATOM 539 HD3 LYS 36 49.717 34.914 59.072 1.00 0.00 H

ATOM 540 CE LYS 36 49.439 34.641 61.262 1.00 0.00 C

ATOM 541 HE2 LYS 36 50.490 34.353 61.251 1.00 0.00 H

ATOM 542 HE3 LYS 36 49.213 35.437 61.972 1.00 0.00 H

ATOM 543 NZ LYS 36 48.705 33.386 61.371 1.00 0.00 N

ATOM 544 HZ1 LYS 36 48.946 32.692 60.679 1.00 0.00 H

ATOM 545 HZ2 LYS 36 48.737 33.009 62.308 1.00 0.00 H

ATOM 546 HZ3 LYS 36 47.724 33.603 61.273 1.00 0.00 H

ATOM 547 C LYS 36 49.954 37.568 56.355 1.00 0.00 C

ATOM 548 O LYS 36 49.263 36.763 55.753 1.00 0.00 O

ATOM 549 N TYR 37 50.126 38.800 55.909 1.00 0.00 N

ATOM 550 H TYR 37 50.584 39.519 56.450 1.00 0.00 H

ATOM 551 CA TYR 37 49.504 39.237 54.644 1.00 0.00 C

ATOM 552 HA TYR 37 48.479 38.872 54.582 1.00 0.00 H

ATOM 553 CB TYR 37 49.336 40.793 54.637 1.00 0.00 C

ATOM 554 HB2 TYR 37 48.809 41.226 55.488 1.00 0.00 H

ATOM 555 HB3 TYR 37 50.345 41.191 54.526 1.00 0.00 H

ATOM 556 CG TYR 37 48.452 41.105 53.477 1.00 0.00 C

ATOM 557 CD1 TYR 37 47.166 40.642 53.402 1.00 0.00 C

ATOM 558 HD1 TYR 37 46.720 40.117 54.233 1.00 0.00 H

ATOM 559 CE1 TYR 37 46.335 40.684 52.264 1.00 0.00 C

ATOM 560 HE1 TYR 37 45.368 40.208 52.188 1.00 0.00 H

ATOM 561 CZ TYR 37 46.929 41.414 51.171 1.00 0.00 C

ATOM 562 OH TYR 37 46.197 41.529 49.986 1.00 0.00 O

ATOM 563 HH TYR 37 45.351 41.110 50.157 1.00 0.00 H

ATOM 564 CE2 TYR 37 48.251 42.029 51.230 1.00 0.00 C

ATOM 565 HE2 TYR 37 48.554 42.638 50.391 1.00 0.00 H

ATOM 566 CD2 TYR 37 48.985 41.915 52.401 1.00 0.00 C

ATOM 567 HD2 TYR 37 49.972 42.343 52.496 1.00 0.00 H

ATOM 568 C TYR 37 50.187 38.671 53.440 1.00 0.00 C

ATOM 569 O TYR 37 49.597 38.369 52.401 1.00 0.00 O

ATOM 570 N GLU 38 51.489 38.435 53.613 1.00 0.00 N

ATOM 571 H GLU 38 51.886 38.669 54.513 1.00 0.00 H

ATOM 572 CA GLU 38 52.238 37.530 52.639 1.00 0.00 C

ATOM 573 HA GLU 38 52.082 37.840 51.605 1.00 0.00 H

ATOM 574 CB GLU 38 53.779 37.704 52.776 1.00 0.00 C

ATOM 575 HB2 GLU 38 53.994 38.716 53.117 1.00 0.00 H

ATOM 576 HB3 GLU 38 54.157 36.978 53.496 1.00 0.00 H

ATOM 577 CG GLU 38 54.435 37.502 51.375 1.00 0.00 C

ATOM 578 HG2 GLU 38 53.949 36.689 50.836 1.00 0.00 H

ATOM 579 HG3 GLU 38 54.335 38.428 50.808 1.00 0.00 H

ATOM 580 CD GLU 38 55.952 37.071 51.509 1.00 0.00 C

ATOM 581 OE1 GLU 38 56.321 36.102 50.745 1.00 0.00 O

ATOM 582 OE2 GLU 38 56.703 37.727 52.189 1.00 0.00 O

ATOM 583 C GLU 38 51.866 36.057 52.661 1.00 0.00 C

ATOM 584 O GLU 38 51.836 35.411 51.644 1.00 0.00 O

ATOM 585 N ALA 39 51.541 35.455 53.813 1.00 0.00 N

ATOM 586 H ALA 39 51.506 36.027 54.645 1.00 0.00 H

ATOM 587 CA ALA 39 50.703 34.231 53.882 1.00 0.00 C

ATOM 588 HA ALA 39 51.239 33.452 53.340 1.00 0.00 H

ATOM 589 CB ALA 39 50.771 33.771 55.230 1.00 0.00 C

ATOM 590 HB1 ALA 39 51.803 33.633 55.555 1.00 0.00 H

ATOM 591 HB2 ALA 39 50.202 34.399 55.916 1.00 0.00 H

ATOM 592 HB3 ALA 39 50.276 32.819 55.420 1.00 0.00 H

ATOM 593 C ALA 39 49.203 34.226 53.363 1.00 0.00 C

ATOM 594 O ALA 39 48.649 33.183 53.058 1.00 0.00 O

ATOM 595 N GLU 40 48.633 35.424 53.216 1.00 0.00 N

ATOM 596 H GLU 40 49.328 36.156 53.254 1.00 0.00 H

ATOM 597 CA GLU 40 47.230 35.867 53.231 1.00 0.00 C

ATOM 598 HA GLU 40 47.253 36.911 53.545 1.00 0.00 H

ATOM 599 CB GLU 40 46.681 35.735 51.795 1.00 0.00 C

ATOM 600 HB2 GLU 40 47.456 35.853 51.037 1.00 0.00 H

ATOM 601 HB3 GLU 40 46.327 34.705 51.743 1.00 0.00 H

ATOM 602 CG GLU 40 45.616 36.787 51.461 1.00 0.00 C

ATOM 603 HG2 GLU 40 44.785 36.848 52.164 1.00 0.00 H

ATOM 604 HG3 GLU 40 46.052 37.786 51.463 1.00 0.00 H

ATOM 605 CD GLU 40 45.066 36.652 50.034 1.00 0.00 C

ATOM 606 OE1 GLU 40 45.142 37.681 49.353 1.00 0.00 O

ATOM 607 OE2 GLU 40 44.668 35.577 49.597 1.00 0.00 O

ATOM 608 C GLU 40 46.301 35.194 54.333 1.00 0.00 C

ATOM 609 O GLU 40 45.108 35.222 54.134 1.00 0.00 O

ATOM 610 N GLU 41 46.960 34.706 55.378 1.00 0.00 N

ATOM 611 H GLU 41 47.900 35.069 55.430 1.00 0.00 H

ATOM 612 CA GLU 41 46.446 33.956 56.568 1.00 0.00 C

ATOM 613 HA GLU 41 45.627 33.394 56.119 1.00 0.00 H

ATOM 614 CB GLU 41 47.586 33.093 57.106 1.00 0.00 C

ATOM 615 HB2 GLU 41 47.810 32.457 56.249 1.00 0.00 H

ATOM 616 HB3 GLU 41 48.423 33.702 57.449 1.00 0.00 H

ATOM 617 CG GLU 41 47.147 32.048 58.190 1.00 0.00 C

ATOM 618 HG2 GLU 41 46.866 32.586 59.095 1.00 0.00 H

ATOM 619 HG3 GLU 41 46.264 31.560 57.778 1.00 0.00 H

ATOM 620 CD GLU 41 48.294 31.045 58.395 1.00 0.00 C

ATOM 621 OE1 GLU 41 48.452 30.081 57.553 1.00 0.00 O

ATOM 622 OE2 GLU 41 49.024 31.186 59.443 1.00 0.00 O

ATOM 623 C GLU 41 45.835 34.819 57.628 1.00 0.00 C

ATOM 624 O GLU 41 46.396 35.922 57.937 1.00 0.00 O

ATOM 625 N PRO 42 44.688 34.396 58.242 1.00 0.00 N

ATOM 626 CD PRO 42 43.879 33.105 58.050 1.00 0.00 C

ATOM 627 HD2 PRO 42 44.127 32.374 58.819 1.00 0.00 H

ATOM 628 HD3 PRO 42 43.988 32.634 57.073 1.00 0.00 H

ATOM 629 CG PRO 42 42.413 33.435 58.191 1.00 0.00 C

ATOM 630 HG2 PRO 42 41.849 32.567 58.535 1.00 0.00 H

ATOM 631 HG3 PRO 42 42.045 33.974 57.318 1.00 0.00 H

ATOM 632 CB PRO 42 42.592 34.338 59.406 1.00 0.00 C

ATOM 633 HB2 PRO 42 42.774 33.826 60.351 1.00 0.00 H

ATOM 634 HB3 PRO 42 41.680 34.929 59.488 1.00 0.00 H

ATOM 635 CA PRO 42 43.895 35.150 59.211 1.00 0.00 C

ATOM 636 HA PRO 42 43.639 36.001 58.579 1.00 0.00 H

ATOM 637 C PRO 42 44.638 35.491 60.524 1.00 0.00 C

ATOM 638 O PRO 42 45.677 34.893 60.963 1.00 0.00 O

ATOM 639 N VAL 43 44.051 36.368 61.275 1.00 0.00 N

ATOM 640 H VAL 43 43.199 36.804 60.951 1.00 0.00 H

ATOM 641 CA VAL 43 44.684 37.049 62.401 1.00 0.00 C

ATOM 642 HA VAL 43 45.421 36.353 62.800 1.00 0.00 H

ATOM 643 CB VAL 43 45.375 38.355 62.054 1.00 0.00 C

ATOM 644 HB VAL 43 45.623 38.813 63.012 1.00 0.00 H

ATOM 645 CG1 VAL 43 46.776 38.279 61.330 1.00 0.00 C

ATOM 646 HG11 VAL 43 47.150 39.259 61.032 1.00 0.00 H

ATOM 647 HG12 VAL 43 47.563 37.879 61.969 1.00 0.00 H

ATOM 648 HG13 VAL 43 46.733 37.774 60.365 1.00 0.00 H

ATOM 649 CG2 VAL 43 44.410 39.282 61.317 1.00 0.00 C

ATOM 650 HG21 VAL 43 44.736 40.321 61.262 1.00 0.00 H

ATOM 651 HG22 VAL 43 44.218 39.075 60.264 1.00 0.00 H

ATOM 652 HG23 VAL 43 43.434 39.321 61.801 1.00 0.00 H

ATOM 653 C VAL 43 43.728 37.217 63.577 1.00 0.00 C

ATOM 654 O VAL 43 42.527 37.611 63.448 1.00 0.00 O

ATOM 655 N THR 44 44.310 36.996 64.742 1.00 0.00 N

ATOM 656 H THR 44 45.268 36.686 64.657 1.00 0.00 H

ATOM 657 CA THR 44 43.671 37.054 66.071 1.00 0.00 C

ATOM 658 HA THR 44 42.948 37.867 66.004 1.00 0.00 H

ATOM 659 CB THR 44 42.939 35.676 66.285 1.00 0.00 C

ATOM 660 HB THR 44 42.320 35.438 65.420 1.00 0.00 H

ATOM 661 CG2 THR 44 43.998 34.556 66.622 1.00 0.00 C

ATOM 662 HG21 THR 44 43.536 33.584 66.792 1.00 0.00 H

ATOM 663 HG22 THR 44 44.747 34.494 65.833 1.00 0.00 H

ATOM 664 HG23 THR 44 44.481 34.691 67.589 1.00 0.00 H

ATOM 665 OG1 THR 44 42.055 35.779 67.361 1.00 0.00 O

ATOM 666 HG1 THR 44 41.594 34.947 67.492 1.00 0.00 H

ATOM 667 C THR 44 44.651 37.383 67.261 1.00 0.00 C

ATOM 668 O THR 44 45.854 37.129 67.171 1.00 0.00 O

ATOM 669 N GLU 45 44.097 37.988 68.344 1.00 0.00 N

ATOM 670 H GLU 45 43.110 38.172 68.454 1.00 0.00 H

ATOM 671 CA GLU 45 44.775 38.571 69.486 1.00 0.00 C

ATOM 672 HA GLU 45 45.477 39.285 69.055 1.00 0.00 H

ATOM 673 CB GLU 45 43.830 39.364 70.332 1.00 0.00 C

ATOM 674 HB2 GLU 45 42.937 38.770 70.527 1.00 0.00 H

ATOM 675 HB3 GLU 45 44.316 39.553 71.289 1.00 0.00 H

ATOM 676 CG GLU 45 43.487 40.769 69.633 1.00 0.00 C

ATOM 677 HG2 GLU 45 44.469 41.193 69.422 1.00 0.00 H

ATOM 678 HG3 GLU 45 42.920 40.552 68.728 1.00 0.00 H

ATOM 679 CD GLU 45 42.537 41.531 70.556 1.00 0.00 C

ATOM 680 OE1 GLU 45 43.085 42.110 71.486 1.00 0.00 O

ATOM 681 OE2 GLU 45 41.303 41.626 70.337 1.00 0.00 O

ATOM 682 C GLU 45 45.579 37.636 70.381 1.00 0.00 C

ATOM 683 O GLU 45 46.706 37.974 70.762 1.00 0.00 O

ATOM 684 N ALA 46 45.181 36.372 70.594 1.00 0.00 N

ATOM 685 H ALA 46 44.253 36.116 70.289 1.00 0.00 H

ATOM 686 CA ALA 46 45.932 35.381 71.339 1.00 0.00 C

ATOM 687 HA ALA 46 46.258 35.783 72.298 1.00 0.00 H

ATOM 688 CB ALA 46 45.066 34.128 71.439 1.00 0.00 C

ATOM 689 HB1 ALA 46 44.106 34.419 71.866 1.00 0.00 H

ATOM 690 HB2 ALA 46 44.910 33.735 70.435 1.00 0.00 H

ATOM 691 HB3 ALA 46 45.638 33.492 72.115 1.00 0.00 H

ATOM 692 C ALA 46 47.330 34.991 70.590 1.00 0.00 C

ATOM 693 O ALA 46 48.383 34.777 71.251 1.00 0.00 O

ATOM 694 N GLU 47 47.369 34.903 69.262 1.00 0.00 N

ATOM 695 H GLU 47 46.489 34.986 68.773 1.00 0.00 H

ATOM 696 CA GLU 47 48.592 34.535 68.514 1.00 0.00 C

ATOM 697 HA GLU 47 49.012 33.621 68.934 1.00 0.00 H

ATOM 698 CB GLU 47 48.221 34.240 67.031 1.00 0.00 C

ATOM 699 HB2 GLU 47 47.431 33.491 66.970 1.00 0.00 H

ATOM 700 HB3 GLU 47 47.783 35.152 66.625 1.00 0.00 H

ATOM 701 CG GLU 47 49.395 34.020 66.134 1.00 0.00 C

ATOM 702 HG2 GLU 47 49.954 34.950 66.230 1.00 0.00 H

ATOM 703 HG3 GLU 47 50.059 33.193 66.386 1.00 0.00 H

ATOM 704 CD GLU 47 48.983 33.828 64.650 1.00 0.00 C

ATOM 705 OE1 GLU 47 49.509 32.914 63.995 1.00 0.00 O

ATOM 706 OE2 GLU 47 48.191 34.680 64.124 1.00 0.00 O

ATOM 707 C GLU 47 49.520 35.638 68.614 1.00 0.00 C

ATOM 708 O GLU 47 50.711 35.334 68.609 1.00 0.00 O

ATOM 709 N MET 48 49.045 36.891 68.580 1.00 0.00 N

ATOM 710 H MET 48 48.044 37.024 68.584 1.00 0.00 H

ATOM 711 CA MET 48 49.962 38.061 68.618 1.00 0.00 C

ATOM 712 HA MET 48 50.763 37.958 67.886 1.00 0.00 H

ATOM 713 CB MET 48 49.252 39.317 68.255 1.00 0.00 C

ATOM 714 HB2 MET 48 48.603 39.663 69.059 1.00 0.00 H

ATOM 715 HB3 MET 48 50.056 40.052 68.234 1.00 0.00 H

ATOM 716 CG MET 48 48.305 39.450 67.016 1.00 0.00 C

ATOM 717 HG2 MET 48 48.855 39.139 66.128 1.00 0.00 H

ATOM 718 HG3 MET 48 47.473 38.758 67.144 1.00 0.00 H

ATOM 719 SD MET 48 47.718 41.098 66.620 1.00 0.00 S

ATOM 720 CE MET 48 46.341 40.883 65.395 1.00 0.00 C

ATOM 721 HE1 MET 48 45.738 40.026 65.693 1.00 0.00 H

ATOM 722 HE2 MET 48 45.712 41.767 65.502 1.00 0.00 H

ATOM 723 HE3 MET 48 46.545 40.780 64.329 1.00 0.00 H

ATOM 724 C MET 48 50.721 38.157 69.943 1.00 0.00 C

ATOM 725 O MET 48 51.821 38.691 69.919 1.00 0.00 O

ATOM 726 N LEU 49 50.131 37.673 71.031 1.00 0.00 N

ATOM 727 H LEU 49 49.204 37.335 70.818 1.00 0.00 H

ATOM 728 CA LEU 49 50.724 37.382 72.311 1.00 0.00 C

ATOM 729 HA LEU 49 51.251 38.301 72.568 1.00 0.00 H

ATOM 730 CB LEU 49 49.707 37.228 73.426 1.00 0.00 C

ATOM 731 HB2 LEU 49 49.011 36.432 73.160 1.00 0.00 H

ATOM 732 HB3 LEU 49 50.267 37.089 74.351 1.00 0.00 H

ATOM 733 CG LEU 49 48.891 38.526 73.746 1.00 0.00 C

ATOM 734 HG LEU 49 48.714 39.111 72.843 1.00 0.00 H

ATOM 735 CD1 LEU 49 47.666 38.143 74.513 1.00 0.00 C

ATOM 736 HD11 LEU 49 47.962 37.625 75.425 1.00 0.00 H

ATOM 737 HD12 LEU 49 47.063 39.017 74.762 1.00 0.00 H

ATOM 738 HD13 LEU 49 47.051 37.413 73.987 1.00 0.00 H

ATOM 739 CD2 LEU 49 49.638 39.528 74.607 1.00 0.00 C

ATOM 740 HD21 LEU 49 49.035 40.421 74.774 1.00 0.00 H

ATOM 741 HD22 LEU 49 49.781 39.068 75.585 1.00 0.00 H

ATOM 742 HD23 LEU 49 50.635 39.700 74.201 1.00 0.00 H

ATOM 743 C LEU 49 51.712 36.110 72.260 1.00 0.00 C

ATOM 744 O LEU 49 52.783 36.083 72.906 1.00 0.00 O

ATOM 745 N MET 50 51.319 35.039 71.477 1.00 0.00 N

ATOM 746 H MET 50 50.449 35.140 70.973 1.00 0.00 H

ATOM 747 CA MET 50 52.164 33.855 71.349 1.00 0.00 C

ATOM 748 HA MET 50 52.368 33.478 72.351 1.00 0.00 H

ATOM 749 CB MET 50 51.344 32.634 70.801 1.00 0.00 C

ATOM 750 HB2 MET 50 50.388 32.645 71.325 1.00 0.00 H

ATOM 751 HB3 MET 50 51.084 32.792 69.755 1.00 0.00 H

ATOM 752 CG MET 50 52.044 31.293 71.054 1.00 0.00 C

ATOM 753 HG2 MET 50 52.816 31.276 70.284 1.00 0.00 H

ATOM 754 HG3 MET 50 52.558 31.281 72.015 1.00 0.00 H

ATOM 755 SD MET 50 50.933 29.811 70.851 1.00 0.00 S

ATOM 756 CE MET 50 49.312 30.088 71.782 1.00 0.00 C

ATOM 757 HE1 MET 50 48.733 29.164 71.792 1.00 0.00 H

ATOM 758 HE2 MET 50 49.547 30.104 72.847 1.00 0.00 H

ATOM 759 HE3 MET 50 48.762 30.935 71.375 1.00 0.00 H

ATOM 760 C MET 50 53.506 34.098 70.498 1.00 0.00 C

ATOM 761 O MET 50 54.554 33.512 70.703 1.00 0.00 O

ATOM 762 N ILE 51 53.396 34.980 69.509 1.00 0.00 N

ATOM 763 H ILE 51 52.484 35.396 69.383 1.00 0.00 H

ATOM 764 CA ILE 51 54.411 35.204 68.385 1.00 0.00 C

ATOM 765 HA ILE 51 55.216 34.513 68.634 1.00 0.00 H

ATOM 766 CB ILE 51 53.757 34.890 67.004 1.00 0.00 C

ATOM 767 HB ILE 51 52.835 35.459 66.882 1.00 0.00 H

ATOM 768 CG2 ILE 51 54.725 35.273 65.866 1.00 0.00 C

ATOM 769 HG21 ILE 51 55.049 36.312 65.929 1.00 0.00 H

ATOM 770 HG22 ILE 51 55.658 34.720 65.981 1.00 0.00 H

ATOM 771 HG23 ILE 51 54.243 35.137 64.898 1.00 0.00 H

ATOM 772 CG1 ILE 51 53.401 33.377 67.076 1.00 0.00 C

ATOM 773 HG12 ILE 51 54.205 32.767 67.489 1.00 0.00 H

ATOM 774 HG13 ILE 51 52.578 33.155 67.755 1.00 0.00 H

ATOM 775 CD1 ILE 51 52.990 32.890 65.719 1.00 0.00 C

ATOM 776 HD11 ILE 51 52.180 33.574 65.466 1.00 0.00 H

ATOM 777 HD12 ILE 51 53.816 32.813 65.012 1.00 0.00 H

ATOM 778 HD13 ILE 51 52.654 31.885 65.976 1.00 0.00 H

ATOM 779 C ILE 51 55.113 36.557 68.501 1.00 0.00 C

ATOM 780 O ILE 51 56.306 36.548 68.281 1.00 0.00 O

ATOM 781 N VAL 52 54.458 37.681 68.774 1.00 0.00 N

ATOM 782 H VAL 52 53.475 37.658 69.003 1.00 0.00 H

ATOM 783 CA VAL 52 55.143 38.996 68.544 1.00 0.00 C

ATOM 784 HA VAL 52 56.186 38.801 68.294 1.00 0.00 H

ATOM 785 CB VAL 52 54.471 39.606 67.260 1.00 0.00 C

ATOM 786 HB VAL 52 54.830 39.049 66.395 1.00 0.00 H

ATOM 787 CG1 VAL 52 52.924 39.674 67.229 1.00 0.00 C

ATOM 788 HG11 VAL 52 52.513 38.692 67.460 1.00 0.00 H

ATOM 789 HG12 VAL 52 52.688 40.305 68.086 1.00 0.00 H

ATOM 790 HG13 VAL 52 52.473 40.026 66.301 1.00 0.00 H

ATOM 791 CG2 VAL 52 54.987 40.967 66.964 1.00 0.00 C

ATOM 792 HG21 VAL 52 54.770 41.701 67.740 1.00 0.00 H

ATOM 793 HG22 VAL 52 56.069 40.982 67.093 1.00 0.00 H

ATOM 794 HG23 VAL 52 54.743 41.184 65.924 1.00 0.00 H

ATOM 795 C VAL 52 55.203 39.916 69.764 1.00 0.00 C

ATOM 796 O VAL 52 56.132 40.629 69.923 1.00 0.00 O

ATOM 797 N ILE 53 54.194 39.873 70.623 1.00 0.00 N

ATOM 798 H ILE 53 53.382 39.363 70.305 1.00 0.00 H

ATOM 799 CA ILE 53 54.140 40.536 71.958 1.00 0.00 C

ATOM 800 HA ILE 53 55.012 41.189 71.972 1.00 0.00 H

ATOM 801 CB ILE 53 52.905 41.401 72.001 1.00 0.00 C

ATOM 802 HB ILE 53 52.075 40.696 72.044 1.00 0.00 H

ATOM 803 CG2 ILE 53 52.964 42.368 73.265 1.00 0.00 C

ATOM 804 HG21 ILE 53 52.692 41.856 74.189 1.00 0.00 H

ATOM 805 HG22 ILE 53 53.944 42.838 73.339 1.00 0.00 H

ATOM 806 HG23 ILE 53 52.308 43.229 73.138 1.00 0.00 H

ATOM 807 CG1 ILE 53 52.590 42.330 70.758 1.00 0.00 C

ATOM 808 HG12 ILE 53 52.528 41.624 69.930 1.00 0.00 H

ATOM 809 HG13 ILE 53 51.627 42.802 70.958 1.00 0.00 H

ATOM 810 CD1 ILE 53 53.620 43.451 70.423 1.00 0.00 C

ATOM 811 HD11 ILE 53 54.580 42.973 70.230 1.00 0.00 H

ATOM 812 HD12 ILE 53 53.315 44.073 69.580 1.00 0.00 H

ATOM 813 HD13 ILE 53 53.743 44.156 71.245 1.00 0.00 H

ATOM 814 C ILE 53 54.345 39.569 73.141 1.00 0.00 C

ATOM 815 O ILE 53 53.484 39.429 74.018 1.00 0.00 O

ATOM 816 N LYS 54 55.486 38.859 73.118 1.00 0.00 N

ATOM 817 H LYS 54 56.132 39.153 72.399 1.00 0.00 H

ATOM 818 CA LYS 54 55.878 37.722 74.027 1.00 0.00 C

ATOM 819 HA LYS 54 55.043 37.021 74.031 1.00 0.00 H

ATOM 820 CB LYS 54 57.104 36.945 73.504 1.00 0.00 C

ATOM 821 HB2 LYS 54 57.963 37.611 73.575 1.00 0.00 H

ATOM 822 HB3 LYS 54 57.341 36.259 74.317 1.00 0.00 H

ATOM 823 CG LYS 54 57.023 36.336 72.116 1.00 0.00 C

ATOM 824 HG2 LYS 54 56.137 35.709 72.016 1.00 0.00 H

ATOM 825 HG3 LYS 54 57.016 37.152 71.394 1.00 0.00 H

ATOM 826 CD LYS 54 58.196 35.419 71.763 1.00 0.00 C

ATOM 827 HD2 LYS 54 58.222 34.516 72.373 1.00 0.00 H

ATOM 828 HD3 LYS 54 58.139 35.198 70.698 1.00 0.00 H

ATOM 829 CE LYS 54 59.416 36.275 72.052 1.00 0.00 C

ATOM 830 HE2 LYS 54 59.306 37.316 71.749 1.00 0.00 H

ATOM 831 HE3 LYS 54 59.600 36.357 73.123 1.00 0.00 H

ATOM 832 NZ LYS 54 60.580 35.658 71.348 1.00 0.00 N

ATOM 833 HZ1 LYS 54 60.459 34.683 71.582 1.00 0.00 H

ATOM 834 HZ2 LYS 54 60.482 35.764 70.349 1.00 0.00 H

ATOM 835 HZ3 LYS 54 61.489 35.804 71.763 1.00 0.00 H

ATOM 836 C LYS 54 56.043 38.148 75.561 1.00 0.00 C

ATOM 837 O LYS 54 55.900 37.245 76.404 1.00 0.00 O

ATOM 838 N TYR 55 56.219 39.430 75.888 1.00 0.00 N

ATOM 839 H TYR 55 56.509 40.044 75.140 1.00 0.00 H

ATOM 840 CA TYR 55 56.429 40.054 77.201 1.00 0.00 C

ATOM 841 HA TYR 55 56.947 39.340 77.841 1.00 0.00 H

ATOM 842 CB TYR 55 57.327 41.253 77.070 1.00 0.00 C

ATOM 843 HB2 TYR 55 58.039 41.204 77.893 1.00 0.00 H

ATOM 844 HB3 TYR 55 58.000 41.175 76.216 1.00 0.00 H

ATOM 845 CG TYR 55 56.736 42.634 76.931 1.00 0.00 C

ATOM 846 CD1 TYR 55 57.294 43.700 77.643 1.00 0.00 C

ATOM 847 HD1 TYR 55 58.064 43.445 78.356 1.00 0.00 H

ATOM 848 CE1 TYR 55 56.906 45.048 77.453 1.00 0.00 C

ATOM 849 HE1 TYR 55 57.426 45.859 77.942 1.00 0.00 H

ATOM 850 CZ TYR 55 55.935 45.326 76.463 1.00 0.00 C

ATOM 851 OH TYR 55 55.636 46.577 76.096 1.00 0.00 O

ATOM 852 HH TYR 55 56.034 47.256 76.646 1.00 0.00 H

ATOM 853 CE2 TYR 55 55.385 44.235 75.716 1.00 0.00 C

ATOM 854 HE2 TYR 55 54.581 44.375 75.008 1.00 0.00 H

ATOM 855 CD2 TYR 55 55.766 42.943 75.947 1.00 0.00 C

ATOM 856 HD2 TYR 55 55.371 42.120 75.371 1.00 0.00 H

ATOM 857 C TYR 55 55.111 40.459 77.814 1.00 0.00 C

ATOM 858 O TYR 55 54.975 40.584 79.005 1.00 0.00 O

ATOM 859 N LYS 56 54.106 40.683 76.987 1.00 0.00 N

ATOM 860 H LYS 56 54.253 40.517 76.001 1.00 0.00 H

ATOM 861 CA LYS 56 52.692 40.853 77.296 1.00 0.00 C

ATOM 862 HA LYS 56 52.226 41.185 76.368 1.00 0.00 H

ATOM 863 CB LYS 56 52.116 39.481 77.762 1.00 0.00 C

ATOM 864 HB2 LYS 56 52.424 39.365 78.801 1.00 0.00 H

ATOM 865 HB3 LYS 56 51.041 39.621 77.651 1.00 0.00 H

ATOM 866 CG LYS 56 52.493 38.246 76.893 1.00 0.00 C

ATOM 867 HG2 LYS 56 52.149 38.362 75.865 1.00 0.00 H

ATOM 868 HG3 LYS 56 53.577 38.139 76.899 1.00 0.00 H

ATOM 869 CD LYS 56 51.712 37.082 77.412 1.00 0.00 C

ATOM 870 HD2 LYS 56 52.055 36.859 78.423 1.00 0.00 H

ATOM 871 HD3 LYS 56 50.684 37.436 77.339 1.00 0.00 H

ATOM 872 CE LYS 56 51.988 35.849 76.522 1.00 0.00 C

ATOM 873 HE2 LYS 56 51.186 35.114 76.466 1.00 0.00 H

ATOM 874 HE3 LYS 56 52.219 36.218 75.523 1.00 0.00 H

ATOM 875 NZ LYS 56 53.260 35.062 76.866 1.00 0.00 N

ATOM 876 HZ1 LYS 56 54.143 35.384 76.497 1.00 0.00 H

ATOM 877 HZ2 LYS 56 53.386 35.052 77.868 1.00 0.00 H

ATOM 878 HZ3 LYS 56 53.172 34.070 76.699 1.00 0.00 H

ATOM 879 C LYS 56 52.347 42.044 78.214 1.00 0.00 C

ATOM 880 O LYS 56 51.158 42.381 78.459 1.00 0.00 O

ATOM 881 N ASP 57 53.278 42.781 78.769 1.00 0.00 N

ATOM 882 H ASP 57 54.169 42.307 78.788 1.00 0.00 H

ATOM 883 CA ASP 57 53.081 43.914 79.773 1.00 0.00 C

ATOM 884 HA ASP 57 52.476 43.565 80.610 1.00 0.00 H

ATOM 885 CB ASP 57 54.406 44.383 80.294 1.00 0.00 C

ATOM 886 HB2 ASP 57 55.062 43.558 80.571 1.00 0.00 H

ATOM 887 HB3 ASP 57 54.901 44.949 79.504 1.00 0.00 H

ATOM 888 CG ASP 57 54.340 45.159 81.587 1.00 0.00 C

ATOM 889 OD1 ASP 57 55.360 45.874 81.846 1.00 0.00 O

ATOM 890 OD2 ASP 57 53.375 45.176 82.348 1.00 0.00 O

ATOM 891 C ASP 57 52.267 45.100 79.189 1.00 0.00 C

ATOM 892 O ASP 57 51.412 45.643 79.880 1.00 0.00 O

ATOM 893 N TYR 58 52.480 45.428 77.941 1.00 0.00 N

ATOM 894 H TYR 58 53.230 45.043 77.387 1.00 0.00 H

ATOM 895 CA TYR 58 51.643 46.304 77.260 1.00 0.00 C

ATOM 896 HA TYR 58 50.689 46.475 77.760 1.00 0.00 H

ATOM 897 CB TYR 58 52.271 47.714 77.215 1.00 0.00 C

ATOM 898 HB2 TYR 58 53.239 47.689 76.714 1.00 0.00 H

ATOM 899 HB3 TYR 58 51.670 48.322 76.539 1.00 0.00 H

ATOM 900 CG TYR 58 52.450 48.497 78.492 1.00 0.00 C

ATOM 901 CD1 TYR 58 51.299 49.116 79.026 1.00 0.00 C

ATOM 902 HD1 TYR 58 50.338 49.112 78.533 1.00 0.00 H

ATOM 903 CE1 TYR 58 51.484 49.844 80.234 1.00 0.00 C

ATOM 904 HE1 TYR 58 50.629 50.292 80.719 1.00 0.00 H

ATOM 905 CZ TYR 58 52.692 50.026 80.908 1.00 0.00 C

ATOM 906 OH TYR 58 52.908 50.790 81.992 1.00 0.00 O

ATOM 907 HH TYR 58 52.181 51.399 82.140 1.00 0.00 H

ATOM 908 CE2 TYR 58 53.824 49.434 80.240 1.00 0.00 C

ATOM 909 HE2 TYR 58 54.816 49.663 80.602 1.00 0.00 H

ATOM 910 CD2 TYR 58 53.695 48.703 79.039 1.00 0.00 C

ATOM 911 HD2 TYR 58 54.534 48.285 78.502 1.00 0.00 H

ATOM 912 C TYR 58 51.299 45.670 75.899 1.00 0.00 C

ATOM 913 O TYR 58 52.073 44.899 75.372 1.00 0.00 O

ATOM 914 N PHE 59 50.150 46.092 75.288 1.00 0.00 N

ATOM 915 H PHE 59 49.683 46.832 75.792 1.00 0.00 H

ATOM 916 CA PHE 59 49.509 45.534 74.026 1.00 0.00 C

ATOM 917 HA PHE 59 50.306 45.572 73.283 1.00 0.00 H

ATOM 918 CB PHE 59 49.169 44.036 74.185 1.00 0.00 C

ATOM 919 HB2 PHE 59 50.094 43.500 74.395 1.00 0.00 H

ATOM 920 HB3 PHE 59 48.403 43.917 74.951 1.00 0.00 H

ATOM 921 CG PHE 59 48.586 43.263 73.036 1.00 0.00 C

ATOM 922 CD1 PHE 59 49.029 43.495 71.727 1.00 0.00 C

ATOM 923 HD1 PHE 59 49.869 44.120 71.464 1.00 0.00 H

ATOM 924 CE1 PHE 59 48.461 42.743 70.676 1.00 0.00 C

ATOM 925 HE1 PHE 59 48.883 42.863 69.689 1.00 0.00 H

ATOM 926 CZ PHE 59 47.348 41.840 70.950 1.00 0.00 C

ATOM 927 HZ PHE 59 46.812 41.312 70.175 1.00 0.00 H

ATOM 928 CE2 PHE 59 46.852 41.715 72.274 1.00 0.00 C

ATOM 929 HE2 PHE 59 46.020 41.039 72.400 1.00 0.00 H

ATOM 930 CD2 PHE 59 47.496 42.411 73.322 1.00 0.00 C

ATOM 931 HD2 PHE 59 47.215 42.249 74.352 1.00 0.00 H

ATOM 932 C PHE 59 48.352 46.318 73.402 1.00 0.00 C

ATOM 933 O PHE 59 48.412 46.640 72.211 1.00 0.00 O

ATOM 934 N PRO 60 47.298 46.711 74.099 1.00 0.00 N

ATOM 935 CD PRO 60 46.979 46.235 75.440 1.00 0.00 C

ATOM 936 HD2 PRO 60 47.515 46.817 76.190 1.00 0.00 H

ATOM 937 HD3 PRO 60 47.265 45.201 75.635 1.00 0.00 H

ATOM 938 CG PRO 60 45.491 46.472 75.617 1.00 0.00 C

ATOM 939 HG2 PRO 60 45.175 46.646 76.645 1.00 0.00 H

ATOM 940 HG3 PRO 60 44.979 45.564 75.299 1.00 0.00 H

ATOM 941 CB PRO 60 45.147 47.564 74.644 1.00 0.00 C

ATOM 942 HB2 PRO 60 45.259 48.539 75.118 1.00 0.00 H

ATOM 943 HB3 PRO 60 44.130 47.453 74.270 1.00 0.00 H

ATOM 944 CA PRO 60 46.108 47.318 73.510 1.00 0.00 C

ATOM 945 HA PRO 60 45.800 46.552 72.799 1.00 0.00 H

ATOM 946 C PRO 60 46.235 48.514 72.564 1.00 0.00 C

ATOM 947 O PRO 60 45.787 48.606 71.412 1.00 0.00 O

ATOM 948 N VAL 61 47.019 49.477 73.071 1.00 0.00 N

ATOM 949 H VAL 61 47.337 49.343 74.020 1.00 0.00 H

ATOM 950 CA VAL 61 47.447 50.698 72.227 1.00 0.00 C

ATOM 951 HA VAL 61 46.592 51.151 71.725 1.00 0.00 H

ATOM 952 CB VAL 61 47.990 51.893 73.103 1.00 0.00 C

ATOM 953 HB VAL 61 48.847 51.553 73.683 1.00 0.00 H

ATOM 954 CG1 VAL 61 48.394 53.109 72.287 1.00 0.00 C

ATOM 955 HG11 VAL 61 48.668 53.963 72.907 1.00 0.00 H

ATOM 956 HG12 VAL 61 49.167 52.912 71.544 1.00 0.00 H

ATOM 957 HG13 VAL 61 47.565 53.455 71.670 1.00 0.00 H

ATOM 958 CG2 VAL 61 46.919 52.442 74.123 1.00 0.00 C

ATOM 959 HG21 VAL 61 47.378 53.317 74.582 1.00 0.00 H

ATOM 960 HG22 VAL 61 46.023 52.778 73.602 1.00 0.00 H

ATOM 961 HG23 VAL 61 46.717 51.747 74.938 1.00 0.00 H

ATOM 962 C VAL 61 48.507 50.291 71.216 1.00 0.00 C

ATOM 963 O VAL 61 48.584 50.903 70.143 1.00 0.00 O

ATOM 964 N ILE 62 49.282 49.219 71.477 1.00 0.00 N

ATOM 965 H ILE 62 49.100 48.599 72.252 1.00 0.00 H

ATOM 966 CA ILE 62 50.345 48.924 70.524 1.00 0.00 C

ATOM 967 HA ILE 62 50.901 49.816 70.237 1.00 0.00 H

ATOM 968 CB ILE 62 51.379 47.948 71.178 1.00 0.00 C

ATOM 969 HB ILE 62 50.921 46.972 71.338 1.00 0.00 H

ATOM 970 CG2 ILE 62 52.616 47.745 70.253 1.00 0.00 C

ATOM 971 HG21 ILE 62 52.453 47.741 69.176 1.00 0.00 H

ATOM 972 HG22 ILE 62 53.442 48.435 70.426 1.00 0.00 H

ATOM 973 HG23 ILE 62 52.935 46.712 70.392 1.00 0.00 H

ATOM 974 CG1 ILE 62 51.878 48.400 72.560 1.00 0.00 C

ATOM 975 HG12 ILE 62 52.318 49.372 72.338 1.00 0.00 H

ATOM 976 HG13 ILE 62 50.995 48.509 73.189 1.00 0.00 H

ATOM 977 CD1 ILE 62 52.929 47.515 73.187 1.00 0.00 C

ATOM 978 HD11 ILE 62 53.884 47.599 72.669 1.00 0.00 H

ATOM 979 HD12 ILE 62 52.985 47.716 74.256 1.00 0.00 H

ATOM 980 HD13 ILE 62 52.688 46.452 73.156 1.00 0.00 H

ATOM 981 C ILE 62 49.656 48.293 69.258 1.00 0.00 C

ATOM 982 O ILE 62 50.097 48.663 68.174 1.00 0.00 O

ATOM 983 N LEU 63 48.705 47.333 69.305 1.00 0.00 N

ATOM 984 H LEU 63 48.487 46.995 70.232 1.00 0.00 H

ATOM 985 CA LEU 63 47.800 47.011 68.218 1.00 0.00 C

ATOM 986 HA LEU 63 48.358 46.573 67.391 1.00 0.00 H

ATOM 987 CB LEU 63 46.920 45.828 68.708 1.00 0.00 C

ATOM 988 HB2 LEU 63 47.544 44.984 69.002 1.00 0.00 H

ATOM 989 HB3 LEU 63 46.316 46.213 69.530 1.00 0.00 H

ATOM 990 CG LEU 63 45.873 45.321 67.731 1.00 0.00 C

ATOM 991 HG LEU 63 45.102 46.003 67.372 1.00 0.00 H

ATOM 992 CD1 LEU 63 46.541 44.648 66.561 1.00 0.00 C

ATOM 993 HD11 LEU 63 45.797 44.304 65.843 1.00 0.00 H

ATOM 994 HD12 LEU 63 47.125 45.435 66.084 1.00 0.00 H

ATOM 995 HD13 LEU 63 47.245 43.846 66.780 1.00 0.00 H

ATOM 996 CD2 LEU 63 45.089 44.188 68.384 1.00 0.00 C

ATOM 997 HD21 LEU 63 44.457 44.704 69.107 1.00 0.00 H

ATOM 998 HD22 LEU 63 44.559 43.642 67.604 1.00 0.00 H

ATOM 999 HD23 LEU 63 45.739 43.485 68.905 1.00 0.00 H

ATOM 1000 C LEU 63 46.893 48.108 67.660 1.00 0.00 C

ATOM 1001 O LEU 63 46.692 48.128 66.429 1.00 0.00 O

ATOM 1002 N LYS 64 46.310 49.030 68.459 1.00 0.00 N

ATOM 1003 H LYS 64 46.550 49.117 69.436 1.00 0.00 H

ATOM 1004 CA LYS 64 45.398 50.050 67.872 1.00 0.00 C

ATOM 1005 HA LYS 64 44.564 49.598 67.334 1.00 0.00 H

ATOM 1006 CB LYS 64 44.842 50.984 68.967 1.00 0.00 C

ATOM 1007 HB2 LYS 64 45.614 51.183 69.710 1.00 0.00 H

ATOM 1008 HB3 LYS 64 44.517 51.934 68.542 1.00 0.00 H

ATOM 1009 CG LYS 64 43.640 50.411 69.636 1.00 0.00 C

ATOM 1010 HG2 LYS 64 42.867 50.233 68.889 1.00 0.00 H

ATOM 1011 HG3 LYS 64 43.776 49.396 70.009 1.00 0.00 H

ATOM 1012 CD LYS 64 43.082 51.316 70.769 1.00 0.00 C

ATOM 1013 HD2 LYS 64 43.939 51.583 71.387 1.00 0.00 H

ATOM 1014 HD3 LYS 64 42.863 52.327 70.424 1.00 0.00 H

ATOM 1015 CE LYS 64 41.994 50.774 71.706 1.00 0.00 C

ATOM 1016 HE2 LYS 64 41.892 51.440 72.563 1.00 0.00 H

ATOM 1017 HE3 LYS 64 41.086 50.683 71.110 1.00 0.00 H

ATOM 1018 NZ LYS 64 42.546 49.547 72.217 1.00 0.00 N

ATOM 1019 HZ1 LYS 64 43.230 49.772 72.926 1.00 0.00 H

ATOM 1020 HZ2 LYS 64 41.793 49.035 72.654 1.00 0.00 H

ATOM 1021 HZ3 LYS 64 43.092 48.993 71.572 1.00 0.00 H

ATOM 1022 C LYS 64 46.114 50.938 66.907 1.00 0.00 C

ATOM 1023 O LYS 64 45.610 51.286 65.849 1.00 0.00 O

ATOM 1024 N ARG 65 47.303 51.441 67.351 1.00 0.00 N

ATOM 1025 H ARG 65 47.742 51.253 68.240 1.00 0.00 H

ATOM 1026 CA ARG 65 48.049 52.276 66.479 1.00 0.00 C

ATOM 1027 HA ARG 65 47.434 53.114 66.151 1.00 0.00 H

ATOM 1028 CB ARG 65 49.206 52.858 67.413 1.00 0.00 C

ATOM 1029 HB2 ARG 65 49.737 52.030 67.882 1.00 0.00 H

ATOM 1030 HB3 ARG 65 49.909 53.300 66.708 1.00 0.00 H

ATOM 1031 CG ARG 65 48.689 53.812 68.491 1.00 0.00 C

ATOM 1032 HG2 ARG 65 47.868 54.320 67.986 1.00 0.00 H

ATOM 1033 HG3 ARG 65 48.174 53.193 69.226 1.00 0.00 H

ATOM 1034 CD ARG 65 49.761 54.648 69.169 1.00 0.00 C

ATOM 1035 HD2 ARG 65 49.303 55.089 70.054 1.00 0.00 H

ATOM 1036 HD3 ARG 65 50.589 54.057 69.561 1.00 0.00 H

ATOM 1037 NE ARG 65 50.325 55.770 68.410 1.00 0.00 N

ATOM 1038 HE ARG 65 51.326 55.778 68.276 1.00 0.00 H

ATOM 1039 CZ ARG 65 49.798 56.869 67.951 1.00 0.00 C

ATOM 1040 NH1 ARG 65 48.543 57.047 68.137 1.00 0.00 N

ATOM 1041 HH11 ARG 65 48.021 56.488 68.797 1.00 0.00 H

ATOM 1042 HH12 ARG 65 48.103 57.850 67.711 1.00 0.00 H

ATOM 1043 NH2 ARG 65 50.506 57.701 67.322 1.00 0.00 N

ATOM 1044 HH21 ARG 65 51.516 57.700 67.319 1.00 0.00 H

ATOM 1045 HH22 ARG 65 50.105 58.561 66.977 1.00 0.00 H

ATOM 1046 C ARG 65 48.668 51.424 65.289 1.00 0.00 C

ATOM 1047 O ARG 65 48.823 51.939 64.213 1.00 0.00 O

ATOM 1048 N ALA 66 48.999 50.100 65.434 1.00 0.00 N

ATOM 1049 H ALA 66 48.835 49.651 66.324 1.00 0.00 H

ATOM 1050 CA ALA 66 49.399 49.196 64.325 1.00 0.00 C

ATOM 1051 HA ALA 66 50.204 49.692 63.783 1.00 0.00 H

ATOM 1052 CB ALA 66 49.764 47.788 64.820 1.00 0.00 C

ATOM 1053 HB1 ALA 66 49.046 47.498 65.588 1.00 0.00 H

ATOM 1054 HB2 ALA 66 49.866 47.158 63.936 1.00 0.00 H

ATOM 1055 HB3 ALA 66 50.704 47.781 65.371 1.00 0.00 H

ATOM 1056 C ALA 66 48.255 48.961 63.210 1.00 0.00 C

ATOM 1057 O ALA 66 48.580 48.960 62.012 1.00 0.00 O

ATOM 1058 N ARG 67 46.986 48.928 63.605 1.00 0.00 N

ATOM 1059 H ARG 67 46.774 48.993 64.590 1.00 0.00 H

ATOM 1060 CA ARG 67 45.866 48.785 62.627 1.00 0.00 C

ATOM 1061 HA ARG 67 46.138 48.007 61.914 1.00 0.00 H

ATOM 1062 CB ARG 67 44.482 48.458 63.282 1.00 0.00 C

ATOM 1063 HB2 ARG 67 44.409 49.144 64.125 1.00 0.00 H

ATOM 1064 HB3 ARG 67 43.671 48.677 62.587 1.00 0.00 H

ATOM 1065 CG ARG 67 44.593 46.936 63.698 1.00 0.00 C

ATOM 1066 HG2 ARG 67 45.091 46.393 62.895 1.00 0.00 H

ATOM 1067 HG3 ARG 67 45.235 46.857 64.576 1.00 0.00 H

ATOM 1068 CD ARG 67 43.167 46.397 64.067 1.00 0.00 C

ATOM 1069 HD2 ARG 67 42.567 46.441 63.157 1.00 0.00 H

ATOM 1070 HD3 ARG 67 43.150 45.333 64.301 1.00 0.00 H

ATOM 1071 NE ARG 67 42.495 47.143 65.112 1.00 0.00 N

ATOM 1072 HE ARG 67 42.066 48.047 64.976 1.00 0.00 H

ATOM 1073 CZ ARG 67 42.161 46.752 66.317 1.00 0.00 C

ATOM 1074 NH1 ARG 67 42.250 45.510 66.701 1.00 0.00 N

ATOM 1075 HH11 ARG 67 42.215 44.675 66.133 1.00 0.00 H

ATOM 1076 HH12 ARG 67 41.886 45.351 67.630 1.00 0.00 H

ATOM 1077 NH2 ARG 67 41.831 47.668 67.179 1.00 0.00 N

ATOM 1078 HH21 ARG 67 41.724 48.600 66.807 1.00 0.00 H

ATOM 1079 HH22 ARG 67 41.618 47.345 68.112 1.00 0.00 H

ATOM 1080 C ARG 67 45.717 50.110 61.893 1.00 0.00 C

ATOM 1081 O ARG 67 45.461 50.112 60.679 1.00 0.00 O

ATOM 1082 N GLU 68 45.861 51.259 62.538 1.00 0.00 N

ATOM 1083 H GLU 68 45.916 51.216 63.546 1.00 0.00 H

ATOM 1084 CA GLU 68 45.606 52.586 61.961 1.00 0.00 C

ATOM 1085 HA GLU 68 44.689 52.609 61.372 1.00 0.00 H

ATOM 1086 CB GLU 68 45.259 53.564 63.177 1.00 0.00 C

ATOM 1087 HB2 GLU 68 46.109 53.608 63.858 1.00 0.00 H

ATOM 1088 HB3 GLU 68 45.208 54.598 62.836 1.00 0.00 H

ATOM 1089 CG GLU 68 43.956 53.236 63.952 1.00 0.00 C

ATOM 1090 HG2 GLU 68 43.889 52.155 64.077 1.00 0.00 H

ATOM 1091 HG3 GLU 68 43.827 53.731 64.915 1.00 0.00 H

ATOM 1092 CD GLU 68 42.831 53.730 63.076 1.00 0.00 C

ATOM 1093 OE1 GLU 68 42.257 52.973 62.295 1.00 0.00 O

ATOM 1094 OE2 GLU 68 42.540 54.952 63.014 1.00 0.00 O

ATOM 1095 C GLU 68 46.837 52.976 61.064 1.00 0.00 C

ATOM 1096 O GLU 68 46.705 53.608 60.046 1.00 0.00 O

ATOM 1097 N PHE 69 48.055 52.455 61.351 1.00 0.00 N

ATOM 1098 H PHE 69 48.193 52.201 62.319 1.00 0.00 H

ATOM 1099 CA PHE 69 49.242 52.462 60.501 1.00 0.00 C

ATOM 1100 HA PHE 69 49.261 53.455 60.052 1.00 0.00 H

ATOM 1101 CB PHE 69 50.546 52.346 61.297 1.00 0.00 C

ATOM 1102 HB2 PHE 69 50.351 51.445 61.878 1.00 0.00 H

ATOM 1103 HB3 PHE 69 51.354 52.187 60.583 1.00 0.00 H

ATOM 1104 CG PHE 69 51.018 53.524 62.082 1.00 0.00 C

ATOM 1105 CD1 PHE 69 51.047 54.828 61.477 1.00 0.00 C

ATOM 1106 HD1 PHE 69 50.764 54.949 60.442 1.00 0.00 H

ATOM 1107 CE1 PHE 69 51.554 55.987 62.094 1.00 0.00 C

ATOM 1108 HE1 PHE 69 51.563 56.949 61.601 1.00 0.00 H

ATOM 1109 CZ PHE 69 51.922 55.911 63.453 1.00 0.00 C

ATOM 1110 HZ PHE 69 52.365 56.761 63.952 1.00 0.00 H

ATOM 1111 CE2 PHE 69 51.736 54.671 64.129 1.00 0.00 C

ATOM 1112 HE2 PHE 69 51.861 54.572 65.198 1.00 0.00 H

ATOM 1113 CD2 PHE 69 51.319 53.534 63.472 1.00 0.00 C

ATOM 1114 HD2 PHE 69 51.256 52.624 64.050 1.00 0.00 H

ATOM 1115 C PHE 69 49.116 51.538 59.326 1.00 0.00 C

ATOM 1116 O PHE 69 49.342 52.056 58.171 1.00 0.00 O

ATOM 1117 N MET 70 48.683 50.290 59.439 1.00 0.00 N

ATOM 1118 H MET 70 48.543 49.899 60.359 1.00 0.00 H

ATOM 1119 CA MET 70 48.486 49.393 58.342 1.00 0.00 C

ATOM 1120 HA MET 70 49.495 49.209 57.971 1.00 0.00 H

ATOM 1121 CB MET 70 47.927 48.105 59.051 1.00 0.00 C

ATOM 1122 HB2 MET 70 48.535 47.695 59.857 1.00 0.00 H

ATOM 1123 HB3 MET 70 46.866 48.204 59.278 1.00 0.00 H

ATOM 1124 CG MET 70 47.956 47.036 57.966 1.00 0.00 C

ATOM 1125 HG2 MET 70 47.272 47.221 57.138 1.00 0.00 H

ATOM 1126 HG3 MET 70 48.965 47.022 57.553 1.00 0.00 H

ATOM 1127 SD MET 70 47.479 45.381 58.488 1.00 0.00 S

ATOM 1128 CE MET 70 45.681 45.400 58.602 1.00 0.00 C

ATOM 1129 HE1 MET 70 45.436 46.032 59.456 1.00 0.00 H

ATOM 1130 HE2 MET 70 45.221 45.707 57.663 1.00 0.00 H

ATOM 1131 HE3 MET 70 45.282 44.427 58.889 1.00 0.00 H

ATOM 1132 C MET 70 47.554 49.929 57.263 1.00 0.00 C

ATOM 1133 O MET 70 47.701 49.778 56.090 1.00 0.00 O

ATOM 1134 N GLU 71 46.611 50.694 57.807 1.00 0.00 N

ATOM 1135 H GLU 71 46.676 50.768 58.813 1.00 0.00 H

ATOM 1136 CA GLU 71 45.670 51.510 57.061 1.00 0.00 C

ATOM 1137 HA GLU 71 45.266 50.843 56.300 1.00 0.00 H

ATOM 1138 CB GLU 71 44.536 51.903 57.979 1.00 0.00 C

ATOM 1139 HB2 GLU 71 44.228 50.968 58.447 1.00 0.00 H

ATOM 1140 HB3 GLU 71 44.836 52.662 58.702 1.00 0.00 H

ATOM 1141 CG GLU 71 43.294 52.353 57.124 1.00 0.00 C

ATOM 1142 HG2 GLU 71 43.628 52.905 56.245 1.00 0.00 H

ATOM 1143 HG3 GLU 71 42.813 51.499 56.647 1.00 0.00 H

ATOM 1144 CD GLU 71 42.252 53.216 57.918 1.00 0.00 C

ATOM 1145 OE1 GLU 71 41.189 52.601 58.137 1.00 0.00 O

ATOM 1146 OE2 GLU 71 42.476 54.342 58.436 1.00 0.00 O

ATOM 1147 C GLU 71 46.126 52.812 56.339 1.00 0.00 C

ATOM 1148 O GLU 71 45.861 53.029 55.191 1.00 0.00 O

ATOM 1149 N LEU 72 47.008 53.619 57.007 1.00 0.00 N

ATOM 1150 H LEU 72 47.183 53.424 57.982 1.00 0.00 H

ATOM 1151 CA LEU 72 47.740 54.780 56.389 1.00 0.00 C

ATOM 1152 HA LEU 72 47.027 55.357 55.801 1.00 0.00 H

ATOM 1153 CB LEU 72 48.179 55.696 57.554 1.00 0.00 C

ATOM 1154 HB2 LEU 72 47.301 55.974 58.137 1.00 0.00 H

ATOM 1155 HB3 LEU 72 48.796 55.023 58.149 1.00 0.00 H

ATOM 1156 CG LEU 72 48.872 57.003 57.041 1.00 0.00 C

ATOM 1157 HG LEU 72 49.658 56.742 56.333 1.00 0.00 H

ATOM 1158 CD1 LEU 72 48.019 57.905 56.200 1.00 0.00 C

ATOM 1159 HD11 LEU 72 48.707 58.645 55.790 1.00 0.00 H

ATOM 1160 HD12 LEU 72 47.531 57.382 55.378 1.00 0.00 H

ATOM 1161 HD13 LEU 72 47.171 58.308 56.753 1.00 0.00 H

ATOM 1162 CD2 LEU 72 49.417 57.720 58.301 1.00 0.00 C

ATOM 1163 HD21 LEU 72 50.327 57.254 58.679 1.00 0.00 H

ATOM 1164 HD22 LEU 72 49.663 58.681 57.850 1.00 0.00 H

ATOM 1165 HD23 LEU 72 48.644 57.797 59.066 1.00 0.00 H

ATOM 1166 C LEU 72 48.880 54.333 55.441 1.00 0.00 C

ATOM 1167 O LEU 72 48.958 54.881 54.331 1.00 0.00 O

ATOM 1168 N LEU 73 49.771 53.357 55.780 1.00 0.00 N

ATOM 1169 H LEU 73 49.609 52.776 56.590 1.00 0.00 H

ATOM 1170 CA LEU 73 51.039 53.289 55.103 1.00 0.00 C

ATOM 1171 HA LEU 73 51.401 54.247 54.728 1.00 0.00 H

ATOM 1172 CB LEU 73 52.041 52.864 56.171 1.00 0.00 C

ATOM 1173 HB2 LEU 73 51.769 51.904 56.611 1.00 0.00 H

ATOM 1174 HB3 LEU 73 53.008 52.605 55.740 1.00 0.00 H

ATOM 1175 CG LEU 73 52.130 53.810 57.418 1.00 0.00 C

ATOM 1176 HG LEU 73 51.144 53.780 57.882 1.00 0.00 H

ATOM 1177 CD1 LEU 73 53.192 53.346 58.432 1.00 0.00 C

ATOM 1178 HD11 LEU 73 53.112 54.113 59.201 1.00 0.00 H

ATOM 1179 HD12 LEU 73 53.017 52.352 58.845 1.00 0.00 H

ATOM 1180 HD13 LEU 73 54.163 53.448 57.946 1.00 0.00 H

ATOM 1181 CD2 LEU 73 52.372 55.270 57.001 1.00 0.00 C

ATOM 1182 HD21 LEU 73 52.722 55.961 57.768 1.00 0.00 H

ATOM 1183 HD22 LEU 73 53.069 55.214 56.164 1.00 0.00 H

ATOM 1184 HD23 LEU 73 51.439 55.661 56.597 1.00 0.00 H

ATOM 1185 C LEU 73 51.132 52.275 53.882 1.00 0.00 C

ATOM 1186 O LEU 73 52.100 52.313 53.167 1.00 0.00 O

ATOM 1187 N PHE 74 50.105 51.408 53.668 1.00 0.00 N

ATOM 1188 H PHE 74 49.323 51.491 54.302 1.00 0.00 H

ATOM 1189 CA PHE 74 49.951 50.361 52.721 1.00 0.00 C

ATOM 1190 HA PHE 74 50.664 50.514 51.911 1.00 0.00 H

ATOM 1191 CB PHE 74 50.210 48.953 53.290 1.00 0.00 C

ATOM 1192 HB2 PHE 74 49.387 48.626 53.925 1.00 0.00 H

ATOM 1193 HB3 PHE 74 50.247 48.145 52.559 1.00 0.00 H

ATOM 1194 CG PHE 74 51.395 48.908 54.144 1.00 0.00 C

ATOM 1195 CD1 PHE 74 52.672 49.276 53.679 1.00 0.00 C

ATOM 1196 HD1 PHE 74 52.833 49.428 52.622 1.00 0.00 H

ATOM 1197 CE1 PHE 74 53.793 49.356 54.561 1.00 0.00 C

ATOM 1198 HE1 PHE 74 54.752 49.416 54.069 1.00 0.00 H

ATOM 1199 CZ PHE 74 53.654 49.245 55.957 1.00 0.00 C

ATOM 1200 HZ PHE 74 54.515 49.449 56.576 1.00 0.00 H

ATOM 1201 CE2 PHE 74 52.368 48.898 56.408 1.00 0.00 C

ATOM 1202 HE2 PHE 74 52.238 48.726 57.466 1.00 0.00 H

ATOM 1203 CD2 PHE 74 51.307 48.636 55.525 1.00 0.00 C

ATOM 1204 HD2 PHE 74 50.354 48.347 55.942 1.00 0.00 H

ATOM 1205 C PHE 74 48.596 50.412 52.060 1.00 0.00 C

ATOM 1206 O PHE 74 48.475 50.436 50.866 1.00 0.00 O

ATOM 1207 N GLY 75 47.611 50.469 52.941 1.00 0.00 N

ATOM 1208 H GLY 75 47.737 50.497 53.942 1.00 0.00 H

ATOM 1209 CA GLY 75 46.231 50.451 52.629 1.00 0.00 C

ATOM 1210 HA2 GLY 75 45.900 51.290 53.241 1.00 0.00 H

ATOM 1211 HA3 GLY 75 46.144 50.862 51.623 1.00 0.00 H

ATOM 1212 C GLY 75 45.403 49.194 52.850 1.00 0.00 C

ATOM 1213 O GLY 75 44.960 48.501 51.925 1.00 0.00 O

ATOM 1214 N LEU 76 45.356 48.778 54.160 1.00 0.00 N

ATOM 1215 H LEU 76 45.819 49.362 54.840 1.00 0.00 H

ATOM 1216 CA LEU 76 44.843 47.495 54.554 1.00 0.00 C

ATOM 1217 HA LEU 76 44.128 47.141 53.812 1.00 0.00 H

ATOM 1218 CB LEU 76 45.871 46.393 54.812 1.00 0.00 C

ATOM 1219 HB2 LEU 76 46.823 46.850 55.082 1.00 0.00 H

ATOM 1220 HB3 LEU 76 45.545 45.748 55.628 1.00 0.00 H

ATOM 1221 CG LEU 76 46.356 45.562 53.603 1.00 0.00 C

ATOM 1222 HG LEU 76 46.649 46.307 52.863 1.00 0.00 H

ATOM 1223 CD1 LEU 76 47.654 44.732 53.905 1.00 0.00 C

ATOM 1224 HD11 LEU 76 47.970 44.196 53.010 1.00 0.00 H

ATOM 1225 HD12 LEU 76 48.398 45.501 54.114 1.00 0.00 H

ATOM 1226 HD13 LEU 76 47.593 44.082 54.778 1.00 0.00 H

ATOM 1227 CD2 LEU 76 45.191 44.653 53.169 1.00 0.00 C

ATOM 1228 HD21 LEU 76 45.481 44.224 52.210 1.00 0.00 H

ATOM 1229 HD22 LEU 76 44.755 44.037 53.955 1.00 0.00 H

ATOM 1230 HD23 LEU 76 44.420 45.370 52.888 1.00 0.00 H

ATOM 1231 C LEU 76 43.995 47.725 55.813 1.00 0.00 C

ATOM 1232 O LEU 76 44.205 48.637 56.616 1.00 0.00 O

ATOM 1233 N ALA 77 42.881 46.968 55.855 1.00 0.00 N

ATOM 1234 H ALA 77 42.735 46.336 55.081 1.00 0.00 H

ATOM 1235 CA ALA 77 41.923 46.956 56.963 1.00 0.00 C

ATOM 1236 HA ALA 77 42.212 47.619 57.779 1.00 0.00 H

ATOM 1237 CB ALA 77 40.560 47.611 56.438 1.00 0.00 C

ATOM 1238 HB1 ALA 77 39.766 47.384 57.149 1.00 0.00 H

ATOM 1239 HB2 ALA 77 40.656 48.694 56.360 1.00 0.00 H

ATOM 1240 HB3 ALA 77 40.218 47.198 55.489 1.00 0.00 H

ATOM 1241 C ALA 77 41.786 45.503 57.398 1.00 0.00 C

ATOM 1242 O ALA 77 42.439 44.626 56.802 1.00 0.00 O

ATOM 1243 N LEU 78 40.969 45.243 58.405 1.00 0.00 N

ATOM 1244 H LEU 78 40.277 45.932 58.662 1.00 0.00 H

ATOM 1245 CA LEU 78 40.606 43.925 58.785 1.00 0.00 C

ATOM 1246 HA LEU 78 41.004 43.256 58.022 1.00 0.00 H

ATOM 1247 CB LEU 78 41.253 43.542 60.174 1.00 0.00 C

ATOM 1248 HB2 LEU 78 40.968 44.233 60.967 1.00 0.00 H

ATOM 1249 HB3 LEU 78 40.792 42.570 60.354 1.00 0.00 H

ATOM 1250 CG LEU 78 42.816 43.330 60.191 1.00 0.00 C

ATOM 1251 HG LEU 78 43.296 44.299 60.049 1.00 0.00 H

ATOM 1252 CD1 LEU 78 43.105 42.792 61.641 1.00 0.00 C

ATOM 1253 HD11 LEU 78 42.446 41.949 61.851 1.00 0.00 H

ATOM 1254 HD12 LEU 78 44.131 42.428 61.692 1.00 0.00 H

ATOM 1255 HD13 LEU 78 43.076 43.605 62.366 1.00 0.00 H

ATOM 1256 CD2 LEU 78 43.464 42.276 59.213 1.00 0.00 C

ATOM 1257 HD21 LEU 78 44.541 42.348 59.366 1.00 0.00 H

ATOM 1258 HD22 LEU 78 43.124 41.262 59.425 1.00 0.00 H

ATOM 1259 HD23 LEU 78 43.168 42.633 58.226 1.00 0.00 H

ATOM 1260 C LEU 78 39.085 43.732 58.834 1.00 0.00 C

ATOM 1261 O LEU 78 38.410 44.694 59.061 1.00 0.00 O

ATOM 1262 N ILE 79 38.479 42.535 58.707 1.00 0.00 N

ATOM 1263 H ILE 79 38.987 41.698 58.459 1.00 0.00 H

ATOM 1264 CA ILE 79 37.047 42.342 58.549 1.00 0.00 C

ATOM 1265 HA ILE 79 36.509 43.125 59.083 1.00 0.00 H

ATOM 1266 CB ILE 79 36.475 42.419 57.122 1.00 0.00 C

ATOM 1267 HB ILE 79 35.457 42.034 57.059 1.00 0.00 H

ATOM 1268 CG2 ILE 79 36.375 43.884 56.741 1.00 0.00 C

ATOM 1269 HG21 ILE 79 37.397 44.148 56.468 1.00 0.00 H

ATOM 1270 HG22 ILE 79 35.730 43.890 55.862 1.00 0.00 H

ATOM 1271 HG23 ILE 79 35.944 44.505 57.525 1.00 0.00 H

ATOM 1272 CG1 ILE 79 37.281 41.611 56.048 1.00 0.00 C

ATOM 1273 HG12 ILE 79 38.269 42.053 55.916 1.00 0.00 H

ATOM 1274 HG13 ILE 79 37.530 40.646 56.490 1.00 0.00 H

ATOM 1275 CD1 ILE 79 36.562 41.361 54.798 1.00 0.00 C

ATOM 1276 HD11 ILE 79 36.354 42.351 54.392 1.00 0.00 H

ATOM 1277 HD12 ILE 79 37.135 40.851 54.024 1.00 0.00 H

ATOM 1278 HD13 ILE 79 35.593 40.884 54.942 1.00 0.00 H

ATOM 1279 C ILE 79 36.744 41.012 59.210 1.00 0.00 C

ATOM 1280 O ILE 79 37.397 39.964 59.098 1.00 0.00 O

ATOM 1281 N GLU 80 35.711 41.054 59.997 1.00 0.00 N

ATOM 1282 H GLU 80 35.432 41.998 60.223 1.00 0.00 H

ATOM 1283 CA GLU 80 35.107 39.893 60.754 1.00 0.00 C

ATOM 1284 HA GLU 80 35.822 39.527 61.491 1.00 0.00 H

ATOM 1285 CB GLU 80 33.865 40.359 61.612 1.00 0.00 C

ATOM 1286 HB2 GLU 80 33.100 40.667 60.899 1.00 0.00 H

ATOM 1287 HB3 GLU 80 33.564 39.520 62.239 1.00 0.00 H

ATOM 1288 CG GLU 80 34.295 41.455 62.624 1.00 0.00 C

ATOM 1289 HG2 GLU 80 35.249 41.162 63.061 1.00 0.00 H

ATOM 1290 HG3 GLU 80 34.382 42.432 62.149 1.00 0.00 H

ATOM 1291 CD GLU 80 33.226 41.598 63.730 1.00 0.00 C

ATOM 1292 OE1 GLU 80 32.189 42.285 63.507 1.00 0.00 O

ATOM 1293 OE2 GLU 80 33.420 41.065 64.810 1.00 0.00 O

ATOM 1294 C GLU 80 34.520 38.794 59.895 1.00 0.00 C

ATOM 1295 O GLU 80 34.060 38.917 58.767 1.00 0.00 O

ATOM 1296 N VAL 81 34.588 37.583 60.439 1.00 0.00 N

ATOM 1297 H VAL 81 34.909 37.548 61.396 1.00 0.00 H

ATOM 1298 CA VAL 81 34.169 36.358 59.805 1.00 0.00 C

ATOM 1299 HA VAL 81 33.501 36.518 58.959 1.00 0.00 H

ATOM 1300 CB VAL 81 35.470 35.615 59.221 1.00 0.00 C

ATOM 1301 HB VAL 81 35.223 34.632 58.820 1.00 0.00 H

ATOM 1302 CG1 VAL 81 36.049 36.448 58.043 1.00 0.00 C

ATOM 1303 HG11 VAL 81 36.342 37.437 58.397 1.00 0.00 H

ATOM 1304 HG12 VAL 81 36.865 35.948 57.521 1.00 0.00 H

ATOM 1305 HG13 VAL 81 35.265 36.468 57.286 1.00 0.00 H

ATOM 1306 CG2 VAL 81 36.551 35.374 60.271 1.00 0.00 C

ATOM 1307 HG21 VAL 81 37.246 34.642 59.858 1.00 0.00 H

ATOM 1308 HG22 VAL 81 37.082 36.316 60.405 1.00 0.00 H

ATOM 1309 HG23 VAL 81 36.157 35.070 61.241 1.00 0.00 H

ATOM 1310 C VAL 81 33.394 35.430 60.723 1.00 0.00 C

ATOM 1311 O VAL 81 33.543 35.437 61.923 1.00 0.00 O

ATOM 1312 N GLY 82 32.702 34.433 60.093 1.00 0.00 N

ATOM 1313 H GLY 82 32.870 34.403 59.098 1.00 0.00 H

ATOM 1314 CA GLY 82 31.894 33.395 60.704 1.00 0.00 C

ATOM 1315 HA2 GLY 82 30.978 33.767 61.162 1.00 0.00 H

ATOM 1316 HA3 GLY 82 31.755 32.760 59.828 1.00 0.00 H

ATOM 1317 C GLY 82 32.540 32.662 61.846 1.00 0.00 C

ATOM 1318 O GLY 82 31.893 32.618 62.883 1.00 0.00 O

ATOM 1319 N PRO 83 33.844 32.251 61.755 1.00 0.00 N

ATOM 1320 CD PRO 83 34.610 32.092 60.533 1.00 0.00 C

ATOM 1321 HD2 PRO 83 35.282 32.940 60.399 1.00 0.00 H

ATOM 1322 HD3 PRO 83 33.985 32.021 59.643 1.00 0.00 H

ATOM 1323 CG PRO 83 35.404 30.794 60.813 1.00 0.00 C

ATOM 1324 HG2 PRO 83 36.194 30.705 60.067 1.00 0.00 H

ATOM 1325 HG3 PRO 83 34.684 30.002 60.608 1.00 0.00 H

ATOM 1326 CB PRO 83 35.828 31.022 62.309 1.00 0.00 C

ATOM 1327 HB2 PRO 83 36.690 31.671 62.465 1.00 0.00 H

ATOM 1328 HB3 PRO 83 35.991 30.050 62.775 1.00 0.00 H

ATOM 1329 CA PRO 83 34.499 31.625 62.860 1.00 0.00 C

ATOM 1330 HA PRO 83 33.933 30.735 63.134 1.00 0.00 H

ATOM 1331 C PRO 83 34.668 32.540 64.104 1.00 0.00 C

ATOM 1332 O PRO 83 34.879 33.750 64.026 1.00 0.00 O

ATOM 1333 N ASP 84 34.710 31.942 65.261 1.00 0.00 N

ATOM 1334 H ASP 84 34.651 30.935 65.299 1.00 0.00 H

ATOM 1335 CA ASP 84 34.839 32.603 66.552 1.00 0.00 C

ATOM 1336 HA ASP 84 34.098 33.396 66.649 1.00 0.00 H

ATOM 1337 CB ASP 84 34.755 31.749 67.830 1.00 0.00 C

ATOM 1338 HB2 ASP 84 34.892 32.444 68.658 1.00 0.00 H

ATOM 1339 HB3 ASP 84 33.781 31.276 67.952 1.00 0.00 H

ATOM 1340 CG ASP 84 35.729 30.565 67.876 1.00 0.00 C

ATOM 1341 OD1 ASP 84 35.626 29.609 67.073 1.00 0.00 O

ATOM 1342 OD2 ASP 84 36.572 30.584 68.801 1.00 0.00 O

ATOM 1343 C ASP 84 36.111 33.451 66.556 1.00 0.00 C

ATOM 1344 O ASP 84 37.244 33.031 66.372 1.00 0.00 O

ATOM 1345 N HIE 85 35.859 34.690 66.947 1.00 0.00 N

ATOM 1346 H HIE 85 34.869 34.805 67.109 1.00 0.00 H

ATOM 1347 CA HIE 85 36.818 35.702 67.312 1.00 0.00 C

ATOM 1348 HA HIE 85 36.316 36.666 67.403 1.00 0.00 H

ATOM 1349 CB HIE 85 37.422 35.476 68.748 1.00 0.00 C

ATOM 1350 HB2 HIE 85 37.929 34.512 68.783 1.00 0.00 H

ATOM 1351 HB3 HIE 85 38.111 36.263 69.054 1.00 0.00 H

ATOM 1352 CG HIE 85 36.334 35.604 69.745 1.00 0.00 C

ATOM 1353 ND1 HIE 85 35.670 36.788 69.988 1.00 0.00 N

ATOM 1354 CE1 HIE 85 34.811 36.550 70.994 1.00 0.00 C

ATOM 1355 HE1 HIE 85 34.036 37.234 71.306 1.00 0.00 H

ATOM 1356 NE2 HIE 85 34.911 35.260 71.378 1.00 0.00 N

ATOM 1357 HE2 HIE 85 34.513 34.830 72.201 1.00 0.00 H

ATOM 1358 CD2 HIE 85 35.969 34.656 70.612 1.00 0.00 C

ATOM 1359 HD2 HIE 85 36.344 33.644 70.586 1.00 0.00 H

ATOM 1360 C HIE 85 37.801 36.114 66.209 1.00 0.00 C

ATOM 1361 O HIE 85 38.708 36.917 66.499 1.00 0.00 O

ATOM 1362 N PHE 86 37.745 35.631 64.963 1.00 0.00 N

ATOM 1363 H PHE 86 37.152 34.817 64.886 1.00 0.00 H

ATOM 1364 CA PHE 86 38.813 35.927 63.983 1.00 0.00 C

ATOM 1365 HA PHE 86 39.764 36.204 64.437 1.00 0.00 H

ATOM 1366 CB PHE 86 38.973 34.729 63.025 1.00 0.00 C

ATOM 1367 HB2 PHE 86 37.991 34.296 62.834 1.00 0.00 H

ATOM 1368 HB3 PHE 86 39.404 35.083 62.088 1.00 0.00 H

ATOM 1369 CG PHE 86 39.984 33.763 63.575 1.00 0.00 C

ATOM 1370 CD1 PHE 86 39.574 32.623 64.215 1.00 0.00 C

ATOM 1371 HD1 PHE 86 38.525 32.458 64.411 1.00 0.00 H

ATOM 1372 CE1 PHE 86 40.521 31.714 64.697 1.00 0.00 C

ATOM 1373 HE1 PHE 86 40.130 30.868 65.243 1.00 0.00 H

ATOM 1374 CZ PHE 86 41.886 31.989 64.527 1.00 0.00 C

ATOM 1375 HZ PHE 86 42.572 31.278 64.963 1.00 0.00 H

ATOM 1376 CE2 PHE 86 42.329 33.128 63.864 1.00 0.00 C

ATOM 1377 HE2 PHE 86 43.396 33.287 63.806 1.00 0.00 H

ATOM 1378 CD2 PHE 86 41.369 34.054 63.347 1.00 0.00 C

ATOM 1379 HD2 PHE 86 41.625 34.996 62.883 1.00 0.00 H

ATOM 1380 C PHE 86 38.465 37.107 63.114 1.00 0.00 C

ATOM 1381 O PHE 86 37.337 37.471 62.719 1.00 0.00 O

ATOM 1382 N CYS 87 39.534 37.614 62.500 1.00 0.00 N

ATOM 1383 H CYS 87 40.426 37.295 62.852 1.00 0.00 H

ATOM 1384 CA CYS 87 39.527 38.630 61.474 1.00 0.00 C

ATOM 1385 HA CYS 87 38.545 38.649 61.001 1.00 0.00 H

ATOM 1386 CB CYS 87 39.740 40.056 62.115 1.00 0.00 C

ATOM 1387 HB2 CYS 87 40.518 40.105 62.877 1.00 0.00 H

ATOM 1388 HB3 CYS 87 40.276 40.708 61.425 1.00 0.00 H

ATOM 1389 SG CYS 87 38.188 40.716 62.817 1.00 0.00 S

ATOM 1390 HG CYS 87 37.521 39.786 62.128 1.00 0.00 H

ATOM 1391 C CYS 87 40.521 38.376 60.344 1.00 0.00 C

ATOM 1392 O CYS 87 41.599 37.817 60.541 1.00 0.00 O

ATOM 1393 N VAL 88 40.093 38.664 59.088 1.00 0.00 N

ATOM 1394 H VAL 88 39.200 39.135 59.084 1.00 0.00 H

ATOM 1395 CA VAL 88 40.832 38.585 57.783 1.00 0.00 C

ATOM 1396 HA VAL 88 41.846 38.211 57.927 1.00 0.00 H

ATOM 1397 CB VAL 88 40.006 37.677 56.772 1.00 0.00 C

ATOM 1398 HB VAL 88 40.560 37.526 55.845 1.00 0.00 H

ATOM 1399 CG1 VAL 88 39.679 36.234 57.280 1.00 0.00 C

ATOM 1400 HG11 VAL 88 39.000 35.750 56.578 1.00 0.00 H

ATOM 1401 HG12 VAL 88 40.559 35.591 57.286 1.00 0.00 H

ATOM 1402 HG13 VAL 88 39.228 36.281 58.271 1.00 0.00 H

ATOM 1403 CG2 VAL 88 38.640 38.262 56.350 1.00 0.00 C

ATOM 1404 HG21 VAL 88 38.857 39.210 55.858 1.00 0.00 H

ATOM 1405 HG22 VAL 88 38.056 37.675 55.641 1.00 0.00 H

ATOM 1406 HG23 VAL 88 38.095 38.423 57.280 1.00 0.00 H

ATOM 1407 C VAL 88 41.128 39.937 57.100 1.00 0.00 C

ATOM 1408 O VAL 88 40.513 40.976 57.485 1.00 0.00 O

ATOM 1409 N PHE 89 42.008 39.968 56.105 1.00 0.00 N

ATOM 1410 H PHE 89 42.529 39.114 55.964 1.00 0.00 H

ATOM 1411 CA PHE 89 42.460 41.206 55.553 1.00 0.00 C

ATOM 1412 HA PHE 89 42.450 42.012 56.286 1.00 0.00 H

ATOM 1413 CB PHE 89 43.872 40.966 54.989 1.00 0.00 C

ATOM 1414 HB2 PHE 89 43.887 40.054 54.392 1.00 0.00 H

ATOM 1415 HB3 PHE 89 44.169 41.770 54.315 1.00 0.00 H

ATOM 1416 CG PHE 89 44.980 40.744 56.049 1.00 0.00 C

ATOM 1417 CD1 PHE 89 45.704 41.810 56.546 1.00 0.00 C

ATOM 1418 HD1 PHE 89 45.675 42.815 56.153 1.00 0.00 H

ATOM 1419 CE1 PHE 89 46.646 41.664 57.592 1.00 0.00 C

ATOM 1420 HE1 PHE 89 47.325 42.441 57.910 1.00 0.00 H

ATOM 1421 CZ PHE 89 46.807 40.382 58.176 1.00 0.00 C

ATOM 1422 HZ PHE 89 47.482 40.116 58.975 1.00 0.00 H

ATOM 1423 CE2 PHE 89 46.044 39.312 57.731 1.00 0.00 C

ATOM 1424 HE2 PHE 89 46.292 38.307 58.038 1.00 0.00 H

ATOM 1425 CD2 PHE 89 45.170 39.490 56.642 1.00 0.00 C

ATOM 1426 HD2 PHE 89 44.624 38.650 56.235 1.00 0.00 H

ATOM 1427 C PHE 89 41.608 41.615 54.351 1.00 0.00 C

ATOM 1428 O PHE 89 40.967 40.786 53.694 1.00 0.00 O

ATOM 1429 N ALA 90 41.569 42.942 54.151 1.00 0.00 N

ATOM 1430 H ALA 90 42.077 43.589 54.737 1.00 0.00 H

ATOM 1431 CA ALA 90 40.707 43.623 53.192 1.00 0.00 C

ATOM 1432 HA ALA 90 40.875 43.011 52.305 1.00 0.00 H

ATOM 1433 CB ALA 90 39.238 43.702 53.593 1.00 0.00 C

ATOM 1434 HB1 ALA 90 39.213 44.215 54.555 1.00 0.00 H

ATOM 1435 HB2 ALA 90 38.699 44.303 52.861 1.00 0.00 H

ATOM 1436 HB3 ALA 90 38.769 42.722 53.678 1.00 0.00 H

ATOM 1437 C ALA 90 41.366 45.011 52.796 1.00 0.00 C

ATOM 1438 O ALA 90 41.738 45.771 53.678 1.00 0.00 O

ATOM 1439 N ASN 91 41.558 45.258 51.533 1.00 0.00 N

ATOM 1440 H ASN 91 41.300 44.574 50.836 1.00 0.00 H

ATOM 1441 CA ASN 91 42.138 46.520 51.106 1.00 0.00 C

ATOM 1442 HA ASN 91 43.031 46.585 51.726 1.00 0.00 H

ATOM 1443 CB ASN 91 42.562 46.448 49.629 1.00 0.00 C

ATOM 1444 HB2 ASN 91 42.969 47.427 49.378 1.00 0.00 H

ATOM 1445 HB3 ASN 91 43.315 45.683 49.437 1.00 0.00 H

ATOM 1446 CG ASN 91 41.521 46.460 48.562 1.00 0.00 C

ATOM 1447 OD1 ASN 91 40.545 45.766 48.688 1.00 0.00 O

ATOM 1448 ND2 ASN 91 41.799 47.132 47.443 1.00 0.00 N

ATOM 1449 HD21 ASN 91 41.219 46.939 46.639 1.00 0.00 H

ATOM 1450 HD22 ASN 91 42.761 47.367 47.240 1.00 0.00 H

ATOM 1451 C ASN 91 41.171 47.697 51.361 1.00 0.00 C

ATOM 1452 O ASN 91 39.909 47.590 51.473 1.00 0.00 O

ATOM 1453 N THR 92 41.788 48.863 51.503 1.00 0.00 N

ATOM 1454 H THR 92 42.790 48.765 51.587 1.00 0.00 H

ATOM 1455 CA THR 92 41.176 50.160 51.924 1.00 0.00 C

ATOM 1456 HA THR 92 40.403 49.973 52.670 1.00 0.00 H

ATOM 1457 CB THR 92 42.152 51.239 52.459 1.00 0.00 C

ATOM 1458 HB THR 92 41.790 52.266 52.394 1.00 0.00 H

ATOM 1459 CG2 THR 92 42.482 50.902 53.898 1.00 0.00 C

ATOM 1460 HG21 THR 92 43.389 51.382 54.266 1.00 0.00 H

ATOM 1461 HG22 THR 92 41.624 51.190 54.505 1.00 0.00 H

ATOM 1462 HG23 THR 92 42.627 49.824 53.965 1.00 0.00 H

ATOM 1463 OG1 THR 92 43.368 51.216 51.764 1.00 0.00 O

ATOM 1464 HG1 THR 92 43.800 52.029 52.037 1.00 0.00 H

ATOM 1465 C THR 92 40.295 50.735 50.808 1.00 0.00 C

ATOM 1466 O THR 92 40.615 50.656 49.658 1.00 0.00 O

ATOM 1467 N VAL 93 39.199 51.335 51.266 1.00 0.00 N

ATOM 1468 H VAL 93 38.995 51.271 52.253 1.00 0.00 H

ATOM 1469 CA VAL 93 37.994 51.736 50.371 1.00 0.00 C

ATOM 1470 HA VAL 93 37.232 51.888 51.135 1.00 0.00 H

ATOM 1471 CB VAL 93 38.316 53.066 49.673 1.00 0.00 C

ATOM 1472 HB VAL 93 39.056 52.910 48.888 1.00 0.00 H

ATOM 1473 CG1 VAL 93 37.166 53.798 49.039 1.00 0.00 C

ATOM 1474 HG11 VAL 93 36.663 53.183 48.292 1.00 0.00 H

ATOM 1475 HG12 VAL 93 36.448 54.159 49.775 1.00 0.00 H

ATOM 1476 HG13 VAL 93 37.499 54.702 48.530 1.00 0.00 H

ATOM 1477 CG2 VAL 93 38.820 54.149 50.555 1.00 0.00 C

ATOM 1478 HG21 VAL 93 38.195 54.317 51.433 1.00 0.00 H

ATOM 1479 HG22 VAL 93 39.794 53.906 50.980 1.00 0.00 H

ATOM 1480 HG23 VAL 93 38.885 55.139 50.103 1.00 0.00 H

ATOM 1481 C VAL 93 37.363 50.748 49.474 1.00 0.00 C

ATOM 1482 O VAL 93 36.254 50.965 48.931 1.00 0.00 O

ATOM 1483 N GLY 94 37.923 49.548 49.367 1.00 0.00 N

ATOM 1484 H GLY 94 38.796 49.329 49.826 1.00 0.00 H

ATOM 1485 CA GLY 94 37.269 48.447 48.646 1.00 0.00 C

ATOM 1486 HA2 GLY 94 37.513 47.524 49.171 1.00 0.00 H

ATOM 1487 HA3 GLY 94 36.186 48.400 48.525 1.00 0.00 H

ATOM 1488 C GLY 94 37.729 48.194 47.171 1.00 0.00 C

ATOM 1489 O GLY 94 36.952 47.642 46.437 1.00 0.00 O

ATOM 1490 N LEU 95 38.949 48.565 46.802 1.00 0.00 N

ATOM 1491 H LEU 95 39.596 48.855 47.521 1.00 0.00 H

ATOM 1492 CA LEU 95 39.404 48.576 45.442 1.00 0.00 C

ATOM 1493 HA LEU 95 38.540 48.998 44.930 1.00 0.00 H

ATOM 1494 CB LEU 95 40.474 49.604 45.236 1.00 0.00 C

ATOM 1495 HB2 LEU 95 41.380 49.493 45.832 1.00 0.00 H

ATOM 1496 HB3 LEU 95 40.742 49.450 44.190 1.00 0.00 H

ATOM 1497 CG LEU 95 40.029 51.127 45.458 1.00 0.00 C

ATOM 1498 HG LEU 95 40.115 51.324 46.527 1.00 0.00 H

ATOM 1499 CD1 LEU 95 41.229 51.949 44.832 1.00 0.00 C

ATOM 1500 HD11 LEU 95 41.378 51.650 43.794 1.00 0.00 H

ATOM 1501 HD12 LEU 95 40.991 53.012 44.828 1.00 0.00 H

ATOM 1502 HD13 LEU 95 42.128 51.835 45.437 1.00 0.00 H

ATOM 1503 CD2 LEU 95 38.722 51.695 44.828 1.00 0.00 C

ATOM 1504 HD21 LEU 95 38.426 51.202 43.902 1.00 0.00 H

ATOM 1505 HD22 LEU 95 37.834 51.495 45.427 1.00 0.00 H

ATOM 1506 HD23 LEU 95 38.840 52.764 44.651 1.00 0.00 H

ATOM 1507 C LEU 95 39.634 47.189 44.920 1.00 0.00 C

ATOM 1508 O LEU 95 39.989 46.274 45.656 1.00 0.00 O

ATOM 1509 N THR 96 39.385 46.899 43.651 1.00 0.00 N

ATOM 1510 H THR 96 39.078 47.645 43.042 1.00 0.00 H

ATOM 1511 CA THR 96 39.484 45.473 43.242 1.00 0.00 C

ATOM 1512 HA THR 96 39.137 44.820 44.043 1.00 0.00 H

ATOM 1513 CB THR 96 38.655 45.123 41.957 1.00 0.00 C

ATOM 1514 HB THR 96 38.770 44.041 41.885 1.00 0.00 H

ATOM 1515 CG2 THR 96 37.142 45.349 41.962 1.00 0.00 C

ATOM 1516 HG21 THR 96 36.863 46.308 42.397 1.00 0.00 H

ATOM 1517 HG22 THR 96 36.877 45.328 40.905 1.00 0.00 H

ATOM 1518 HG23 THR 96 36.694 44.525 42.518 1.00 0.00 H

ATOM 1519 OG1 THR 96 39.107 45.652 40.719 1.00 0.00 O

ATOM 1520 HG1 THR 96 38.509 46.366 40.487 1.00 0.00 H

ATOM 1521 C THR 96 40.936 44.972 43.104 1.00 0.00 C

ATOM 1522 O THR 96 41.150 43.749 43.142 1.00 0.00 O

ATOM 1523 N ASP 97 41.869 45.925 42.984 1.00 0.00 N

ATOM 1524 H ASP 97 41.617 46.898 42.880 1.00 0.00 H

ATOM 1525 CA ASP 97 43.298 45.778 43.145 1.00 0.00 C

ATOM 1526 HA ASP 97 43.488 44.724 43.348 1.00 0.00 H

ATOM 1527 CB ASP 97 43.975 46.043 41.786 1.00 0.00 C

ATOM 1528 HB2 ASP 97 43.700 45.332 41.007 1.00 0.00 H

ATOM 1529 HB3 ASP 97 43.548 47.004 41.501 1.00 0.00 H

ATOM 1530 CG ASP 97 45.499 45.995 41.904 1.00 0.00 C

ATOM 1531 OD1 ASP 97 46.157 46.826 41.249 1.00 0.00 O

ATOM 1532 OD2 ASP 97 46.001 45.098 42.623 1.00 0.00 O

ATOM 1533 C ASP 97 43.832 46.621 44.276 1.00 0.00 C

ATOM 1534 O ASP 97 43.540 47.781 44.369 1.00 0.00 O

ATOM 1535 N GLU 98 44.600 46.022 45.139 1.00 0.00 N

ATOM 1536 H GLU 98 44.750 45.051 44.906 1.00 0.00 H

ATOM 1537 CA GLU 98 45.296 46.711 46.237 1.00 0.00 C

ATOM 1538 HA GLU 98 44.932 47.738 46.211 1.00 0.00 H

ATOM 1539 CB GLU 98 45.085 46.022 47.529 1.00 0.00 C

ATOM 1540 HB2 GLU 98 45.219 46.706 48.367 1.00 0.00 H

ATOM 1541 HB3 GLU 98 44.026 45.762 47.551 1.00 0.00 H

ATOM 1542 CG GLU 98 45.883 44.800 47.820 1.00 0.00 C

ATOM 1543 HG2 GLU 98 46.925 44.973 47.554 1.00 0.00 H

ATOM 1544 HG3 GLU 98 45.941 44.562 48.882 1.00 0.00 H

ATOM 1545 CD GLU 98 45.374 43.543 47.112 1.00 0.00 C

ATOM 1546 OE1 GLU 98 46.119 42.908 46.316 1.00 0.00 O

ATOM 1547 OE2 GLU 98 44.185 43.201 47.325 1.00 0.00 O

ATOM 1548 C GLU 98 46.742 46.975 45.929 1.00 0.00 C

ATOM 1549 O GLU 98 47.340 47.720 46.687 1.00 0.00 O

ATOM 1550 N GLY 99 47.266 46.441 44.880 1.00 0.00 N

ATOM 1551 H GLY 99 46.699 45.736 44.431 1.00 0.00 H

ATOM 1552 CA GLY 99 48.645 46.544 44.416 1.00 0.00 C

ATOM 1553 HA2 GLY 99 48.711 46.208 43.382 1.00 0.00 H

ATOM 1554 HA3 GLY 99 49.139 47.514 44.481 1.00 0.00 H

ATOM 1555 C GLY 99 49.661 45.654 45.144 1.00 0.00 C

ATOM 1556 O GLY 99 49.322 44.883 46.043 1.00 0.00 O

ATOM 1557 N SER 100 50.932 45.813 44.804 1.00 0.00 N

ATOM 1558 H SER 100 51.065 46.515 44.090 1.00 0.00 H

ATOM 1559 CA SER 100 52.111 45.342 45.515 1.00 0.00 C

ATOM 1560 HA SER 100 52.023 45.698 46.541 1.00 0.00 H

ATOM 1561 CB SER 100 52.130 43.784 45.404 1.00 0.00 C

ATOM 1562 HB2 SER 100 53.107 43.449 45.754 1.00 0.00 H

ATOM 1563 HB3 SER 100 51.331 43.382 46.027 1.00 0.00 H

ATOM 1564 OG SER 100 52.091 43.351 44.053 1.00 0.00 O

ATOM 1565 HG SER 100 51.187 43.489 43.761 1.00 0.00 H

ATOM 1566 C SER 100 53.407 45.932 44.980 1.00 0.00 C

ATOM 1567 O SER 100 53.439 46.306 43.833 1.00 0.00 O

ATOM 1568 N ASP 101 54.435 45.828 45.778 1.00 0.00 N

ATOM 1569 H ASP 101 54.227 45.401 46.669 1.00 0.00 H

ATOM 1570 CA ASP 101 55.848 46.073 45.408 1.00 0.00 C

ATOM 1571 HA ASP 101 55.894 45.875 44.337 1.00 0.00 H

ATOM 1572 CB ASP 101 56.313 47.553 45.613 1.00 0.00 C

ATOM 1573 HB2 ASP 101 55.727 48.202 44.962 1.00 0.00 H

ATOM 1574 HB3 ASP 101 56.068 47.894 46.619 1.00 0.00 H

ATOM 1575 CG ASP 101 57.787 47.725 45.355 1.00 0.00 C

ATOM 1576 OD1 ASP 101 58.138 47.367 44.223 1.00 0.00 O

ATOM 1577 OD2 ASP 101 58.480 48.317 46.190 1.00 0.00 O

ATOM 1578 C ASP 101 56.736 44.993 46.213 1.00 0.00 C

ATOM 1579 O ASP 101 56.242 44.465 47.189 1.00 0.00 O

ATOM 1580 N ASP 102 57.963 44.719 45.817 1.00 0.00 N

ATOM 1581 H ASP 102 58.236 45.295 45.033 1.00 0.00 H

ATOM 1582 CA ASP 102 59.021 43.741 46.372 1.00 0.00 C

ATOM 1583 HA ASP 102 59.738 43.604 45.562 1.00 0.00 H

ATOM 1584 CB ASP 102 59.724 44.593 47.466 1.00 0.00 C

ATOM 1585 HB2 ASP 102 60.026 45.581 47.118 1.00 0.00 H

ATOM 1586 HB3 ASP 102 59.056 44.757 48.311 1.00 0.00 H

ATOM 1587 CG ASP 102 61.032 43.896 47.891 1.00 0.00 C

ATOM 1588 OD1 ASP 102 61.786 43.590 46.943 1.00 0.00 O

ATOM 1589 OD2 ASP 102 61.294 43.754 49.089 1.00 0.00 O

ATOM 1590 C ASP 102 58.460 42.305 46.771 1.00 0.00 C

ATOM 1591 O ASP 102 59.150 41.682 47.556 1.00 0.00 O

ATOM 1592 N GLU 103 57.272 41.972 46.303 1.00 0.00 N

ATOM 1593 H GLU 103 57.009 42.691 45.644 1.00 0.00 H

ATOM 1594 CA GLU 103 56.421 40.789 46.542 1.00 0.00 C

ATOM 1595 HA GLU 103 55.526 40.984 45.952 1.00 0.00 H

ATOM 1596 CB GLU 103 57.008 39.487 45.896 1.00 0.00 C

ATOM 1597 HB2 GLU 103 57.887 39.116 46.423 1.00 0.00 H

ATOM 1598 HB3 GLU 103 56.278 38.689 46.029 1.00 0.00 H

ATOM 1599 CG GLU 103 57.346 39.684 44.453 1.00 0.00 C

ATOM 1600 HG2 GLU 103 56.462 40.170 44.041 1.00 0.00 H

ATOM 1601 HG3 GLU 103 58.241 40.286 44.294 1.00 0.00 H

ATOM 1602 CD GLU 103 57.591 38.374 43.807 1.00 0.00 C

ATOM 1603 OE1 GLU 103 58.297 37.532 44.411 1.00 0.00 O

ATOM 1604 OE2 GLU 103 57.082 38.150 42.699 1.00 0.00 O

ATOM 1605 C GLU 103 55.850 40.609 47.989 1.00 0.00 C

ATOM 1606 O GLU 103 55.640 39.495 48.464 1.00 0.00 O

ATOM 1607 N GLY 104 55.652 41.730 48.696 1.00 0.00 N

ATOM 1608 H GLY 104 55.906 42.631 48.317 1.00 0.00 H

ATOM 1609 CA GLY 104 54.951 41.714 50.013 1.00 0.00 C

ATOM 1610 HA2 GLY 104 54.090 41.055 49.902 1.00 0.00 H

ATOM 1611 HA3 GLY 104 55.581 41.221 50.754 1.00 0.00 H

ATOM 1612 C GLY 104 54.433 43.044 50.662 1.00 0.00 C

ATOM 1613 O GLY 104 54.109 42.902 51.812 1.00 0.00 O

ATOM 1614 N MET 105 54.498 44.221 50.063 1.00 0.00 N

ATOM 1615 H MET 105 55.017 44.322 49.202 1.00 0.00 H

ATOM 1616 CA MET 105 53.892 45.421 50.630 1.00 0.00 C

ATOM 1617 HA MET 105 53.389 45.162 51.561 1.00 0.00 H

ATOM 1618 CB MET 105 54.923 46.422 50.990 1.00 0.00 C

ATOM 1619 HB2 MET 105 55.664 46.517 50.196 1.00 0.00 H

ATOM 1620 HB3 MET 105 54.498 47.424 51.039 1.00 0.00 H

ATOM 1621 CG MET 105 55.656 46.307 52.341 1.00 0.00 C

ATOM 1622 HG2 MET 105 54.895 46.463 53.105 1.00 0.00 H

ATOM 1623 HG3 MET 105 56.108 45.325 52.479 1.00 0.00 H

ATOM 1624 SD MET 105 56.925 47.410 52.733 1.00 0.00 S

ATOM 1625 CE MET 105 57.781 46.624 54.137 1.00 0.00 C

ATOM 1626 HE1 MET 105 57.375 46.841 55.125 1.00 0.00 H

ATOM 1627 HE2 MET 105 57.930 45.562 53.939 1.00 0.00 H

ATOM 1628 HE3 MET 105 58.747 47.121 54.049 1.00 0.00 H

ATOM 1629 C MET 105 52.961 45.995 49.575 1.00 0.00 C

ATOM 1630 O MET 105 53.370 46.251 48.451 1.00 0.00 O

ATOM 1631 N PRO 106 51.657 46.295 49.928 1.00 0.00 N

ATOM 1632 CD PRO 106 50.974 45.764 51.100 1.00 0.00 C

ATOM 1633 HD2 PRO 106 51.218 46.356 51.982 1.00 0.00 H

ATOM 1634 HD3 PRO 106 51.338 44.762 51.324 1.00 0.00 H

ATOM 1635 CG PRO 106 49.499 45.670 50.754 1.00 0.00 C

ATOM 1636 HG2 PRO 106 48.932 45.795 51.677 1.00 0.00 H

ATOM 1637 HG3 PRO 106 49.292 44.689 50.326 1.00 0.00 H

ATOM 1638 CB PRO 106 49.221 46.775 49.767 1.00 0.00 C

ATOM 1639 HB2 PRO 106 48.930 47.656 50.338 1.00 0.00 H

ATOM 1640 HB3 PRO 106 48.471 46.382 49.081 1.00 0.00 H

ATOM 1641 CA PRO 106 50.592 46.843 49.055 1.00 0.00 C

ATOM 1642 HA PRO 106 50.603 46.173 48.195 1.00 0.00 H

ATOM 1643 C PRO 106 50.826 48.328 48.775 1.00 0.00 C

ATOM 1644 O PRO 106 51.438 49.001 49.573 1.00 0.00 O

ATOM 1645 N GLU 107 50.187 48.870 47.711 1.00 0.00 N

ATOM 1646 H GLU 107 49.636 48.192 47.205 1.00 0.00 H

ATOM 1647 CA GLU 107 50.317 50.193 47.139 1.00 0.00 C

ATOM 1648 HA GLU 107 51.102 50.769 47.629 1.00 0.00 H

ATOM 1649 CB GLU 107 50.575 49.946 45.719 1.00 0.00 C

ATOM 1650 HB2 GLU 107 49.820 49.375 45.179 1.00 0.00 H

ATOM 1651 HB3 GLU 107 50.584 50.908 45.206 1.00 0.00 H

ATOM 1652 CG GLU 107 51.968 49.368 45.421 1.00 0.00 C

ATOM 1653 HG2 GLU 107 52.788 50.009 45.745 1.00 0.00 H

ATOM 1654 HG3 GLU 107 52.158 48.410 45.906 1.00 0.00 H

ATOM 1655 CD GLU 107 52.089 49.165 43.888 1.00 0.00 C

ATOM 1656 OE1 GLU 107 51.395 48.348 43.226 1.00 0.00 O

ATOM 1657 OE2 GLU 107 52.950 49.895 43.257 1.00 0.00 O

ATOM 1658 C GLU 107 48.969 51.078 47.216 1.00 0.00 C

ATOM 1659 O GLU 107 48.790 52.199 46.663 1.00 0.00 O

ATOM 1660 N ASN 108 47.968 50.554 47.875 1.00 0.00 N

ATOM 1661 H ASN 108 48.046 49.593 48.175 1.00 0.00 H

ATOM 1662 CA ASN 108 46.597 51.129 47.850 1.00 0.00 C

ATOM 1663 HA ASN 108 46.315 51.193 46.799 1.00 0.00 H

ATOM 1664 CB ASN 108 45.640 50.058 48.463 1.00 0.00 C

ATOM 1665 HB2 ASN 108 45.749 49.105 47.945 1.00 0.00 H

ATOM 1666 HB3 ASN 108 45.845 49.934 49.527 1.00 0.00 H

ATOM 1667 CG ASN 108 44.195 50.290 48.101 1.00 0.00 C

ATOM 1668 OD1 ASN 108 43.867 50.394 46.882 1.00 0.00 O

ATOM 1669 ND2 ASN 108 43.305 50.285 49.029 1.00 0.00 N

ATOM 1670 HD21 ASN 108 42.326 50.306 48.779 1.00 0.00 H

ATOM 1671 HD22 ASN 108 43.504 50.472 50.001 1.00 0.00 H

ATOM 1672 C ASN 108 46.427 52.497 48.398 1.00 0.00 C

ATOM 1673 O ASN 108 45.725 53.347 47.904 1.00 0.00 O

ATOM 1674 N SER 109 47.089 52.780 49.571 1.00 0.00 N

ATOM 1675 H SER 109 47.741 52.127 49.982 1.00 0.00 H

ATOM 1676 CA SER 109 46.804 54.076 50.306 1.00 0.00 C

ATOM 1677 HA SER 109 45.724 54.109 50.451 1.00 0.00 H

ATOM 1678 CB SER 109 47.486 54.210 51.627 1.00 0.00 C

ATOM 1679 HB2 SER 109 47.665 55.267 51.825 1.00 0.00 H

ATOM 1680 HB3 SER 109 46.890 53.667 52.360 1.00 0.00 H

ATOM 1681 OG SER 109 48.787 53.597 51.734 1.00 0.00 O

ATOM 1682 HG SER 109 49.080 53.958 52.574 1.00 0.00 H

ATOM 1683 C SER 109 47.132 55.256 49.493 1.00 0.00 C

ATOM 1684 O SER 109 46.255 56.153 49.579 1.00 0.00 O

ATOM 1685 N LEU 110 48.231 55.316 48.714 1.00 0.00 N

ATOM 1686 H LEU 110 48.870 54.534 48.725 1.00 0.00 H

ATOM 1687 CA LEU 110 48.658 56.537 48.094 1.00 0.00 C

ATOM 1688 HA LEU 110 48.479 57.382 48.760 1.00 0.00 H

ATOM 1689 CB LEU 110 50.227 56.455 47.936 1.00 0.00 C

ATOM 1690 HB2 LEU 110 50.742 56.512 48.895 1.00 0.00 H

ATOM 1691 HB3 LEU 110 50.525 55.483 47.543 1.00 0.00 H

ATOM 1692 CG LEU 110 50.821 57.516 47.066 1.00 0.00 C

ATOM 1693 HG LEU 110 50.285 57.439 46.120 1.00 0.00 H

ATOM 1694 CD1 LEU 110 50.580 58.888 47.679 1.00 0.00 C

ATOM 1695 HD11 LEU 110 49.579 59.316 47.631 1.00 0.00 H

ATOM 1696 HD12 LEU 110 51.060 58.874 48.658 1.00 0.00 H

ATOM 1697 HD13 LEU 110 51.136 59.576 47.043 1.00 0.00 H

ATOM 1698 CD2 LEU 110 52.329 57.218 46.841 1.00 0.00 C

ATOM 1699 HD21 LEU 110 52.741 57.849 46.053 1.00 0.00 H

ATOM 1700 HD22 LEU 110 52.960 57.333 47.722 1.00 0.00 H

ATOM 1701 HD23 LEU 110 52.642 56.236 46.486 1.00 0.00 H

ATOM 1702 C LEU 110 47.864 56.805 46.821 1.00 0.00 C

ATOM 1703 O LEU 110 47.735 57.935 46.423 1.00 0.00 O

ATOM 1704 N LEU 111 47.228 55.758 46.211 1.00 0.00 N

ATOM 1705 H LEU 111 47.325 54.801 46.520 1.00 0.00 H

ATOM 1706 CA LEU 111 46.202 55.999 45.147 1.00 0.00 C

ATOM 1707 HA LEU 111 46.654 56.647 44.395 1.00 0.00 H

ATOM 1708 CB LEU 111 45.784 54.589 44.536 1.00 0.00 C

ATOM 1709 HB2 LEU 111 46.695 54.063 44.252 1.00 0.00 H

ATOM 1710 HB3 LEU 111 45.227 54.069 45.316 1.00 0.00 H

ATOM 1711 CG LEU 111 44.836 54.552 43.347 1.00 0.00 C

ATOM 1712 HG LEU 111 43.936 55.022 43.744 1.00 0.00 H

ATOM 1713 CD1 LEU 111 45.238 55.371 42.133 1.00 0.00 C

ATOM 1714 HD11 LEU 111 45.632 56.336 42.453 1.00 0.00 H

ATOM 1715 HD12 LEU 111 46.099 54.878 41.683 1.00 0.00 H

ATOM 1716 HD13 LEU 111 44.396 55.582 41.472 1.00 0.00 H

ATOM 1717 CD2 LEU 111 44.549 53.110 42.955 1.00 0.00 C

ATOM 1718 HD21 LEU 111 45.247 52.803 42.176 1.00 0.00 H

ATOM 1719 HD22 LEU 111 44.581 52.426 43.803 1.00 0.00 H

ATOM 1720 HD23 LEU 111 43.520 53.072 42.600 1.00 0.00 H

ATOM 1721 C LEU 111 44.947 56.626 45.686 1.00 0.00 C

ATOM 1722 O LEU 111 44.613 57.629 45.170 1.00 0.00 O

ATOM 1723 N ILE 112 44.351 56.097 46.765 1.00 0.00 N

ATOM 1724 H ILE 112 44.741 55.218 47.074 1.00 0.00 H

ATOM 1725 CA ILE 112 43.207 56.793 47.495 1.00 0.00 C

ATOM 1726 HA ILE 112 42.376 56.937 46.806 1.00 0.00 H

ATOM 1727 CB ILE 112 42.741 55.898 48.642 1.00 0.00 C

ATOM 1728 HB ILE 112 43.598 55.754 49.301 1.00 0.00 H

ATOM 1729 CG2 ILE 112 41.504 56.409 49.440 1.00 0.00 C

ATOM 1730 HG21 ILE 112 40.611 56.256 48.833 1.00 0.00 H

ATOM 1731 HG22 ILE 112 41.441 55.965 50.433 1.00 0.00 H

ATOM 1732 HG23 ILE 112 41.493 57.484 49.621 1.00 0.00 H

ATOM 1733 CG1 ILE 112 42.277 54.498 48.130 1.00 0.00 C

ATOM 1734 HG12 ILE 112 41.288 54.508 47.673 1.00 0.00 H

ATOM 1735 HG13 ILE 112 43.052 54.210 47.420 1.00 0.00 H

ATOM 1736 CD1 ILE 112 42.460 53.425 49.278 1.00 0.00 C

ATOM 1737 HD11 ILE 112 43.536 53.293 49.166 1.00 0.00 H

ATOM 1738 HD12 ILE 112 42.225 53.788 50.279 1.00 0.00 H

ATOM 1739 HD13 ILE 112 41.848 52.573 48.982 1.00 0.00 H

ATOM 1740 C ILE 112 43.593 58.229 48.018 1.00 0.00 C

ATOM 1741 O ILE 112 42.838 59.195 47.907 1.00 0.00 O

ATOM 1742 N ILE 113 44.908 58.412 48.352 1.00 0.00 N

ATOM 1743 H ILE 113 45.545 57.645 48.191 1.00 0.00 H

ATOM 1744 CA ILE 113 45.415 59.842 48.561 1.00 0.00 C

ATOM 1745 HA ILE 113 44.757 60.212 49.347 1.00 0.00 H

ATOM 1746 CB ILE 113 46.874 59.910 49.176 1.00 0.00 C

ATOM 1747 HB ILE 113 47.506 59.113 48.783 1.00 0.00 H

ATOM 1748 CG2 ILE 113 47.654 61.231 48.848 1.00 0.00 C

ATOM 1749 HG21 ILE 113 47.043 62.109 49.057 1.00 0.00 H

ATOM 1750 HG22 ILE 113 48.600 61.195 49.387 1.00 0.00 H

ATOM 1751 HG23 ILE 113 47.948 61.248 47.798 1.00 0.00 H

ATOM 1752 CG1 ILE 113 46.656 59.680 50.673 1.00 0.00 C

ATOM 1753 HG12 ILE 113 46.328 60.610 51.138 1.00 0.00 H

ATOM 1754 HG13 ILE 113 45.811 59.014 50.848 1.00 0.00 H

ATOM 1755 CD1 ILE 113 47.928 59.075 51.287 1.00 0.00 C

ATOM 1756 HD11 ILE 113 48.735 59.787 51.118 1.00 0.00 H

ATOM 1757 HD12 ILE 113 47.815 58.997 52.368 1.00 0.00 H

ATOM 1758 HD13 ILE 113 48.188 58.113 50.844 1.00 0.00 H

ATOM 1759 C ILE 113 45.167 60.741 47.250 1.00 0.00 C

ATOM 1760 O ILE 113 44.622 61.806 47.341 1.00 0.00 O

ATOM 1761 N ILE 114 45.759 60.297 46.174 1.00 0.00 N

ATOM 1762 H ILE 114 46.235 59.406 46.199 1.00 0.00 H

ATOM 1763 CA ILE 114 45.775 61.160 44.944 1.00 0.00 C

ATOM 1764 HA ILE 114 46.057 62.150 45.303 1.00 0.00 H

ATOM 1765 CB ILE 114 46.875 60.799 43.900 1.00 0.00 C

ATOM 1766 HB ILE 114 46.804 61.512 43.078 1.00 0.00 H

ATOM 1767 CG2 ILE 114 48.296 60.919 44.534 1.00 0.00 C

ATOM 1768 HG21 ILE 114 49.020 60.855 43.722 1.00 0.00 H

ATOM 1769 HG22 ILE 114 48.423 61.903 44.986 1.00 0.00 H

ATOM 1770 HG23 ILE 114 48.435 60.077 45.212 1.00 0.00 H

ATOM 1771 CG1 ILE 114 46.739 59.337 43.311 1.00 0.00 C

ATOM 1772 HG12 ILE 114 46.671 58.546 44.058 1.00 0.00 H

ATOM 1773 HG13 ILE 114 45.783 59.205 42.803 1.00 0.00 H

ATOM 1774 CD1 ILE 114 47.764 59.031 42.216 1.00 0.00 C

ATOM 1775 HD11 ILE 114 47.946 59.935 41.634 1.00 0.00 H

ATOM 1776 HD12 ILE 114 48.734 58.739 42.620 1.00 0.00 H

ATOM 1777 HD13 ILE 114 47.382 58.227 41.588 1.00 0.00 H

ATOM 1778 C ILE 114 44.364 61.309 44.259 1.00 0.00 C

ATOM 1779 O ILE 114 44.022 62.336 43.756 1.00 0.00 O

ATOM 1780 N LEU 115 43.581 60.259 44.387 1.00 0.00 N

ATOM 1781 H LEU 115 44.032 59.449 44.787 1.00 0.00 H

ATOM 1782 CA LEU 115 42.178 60.201 43.912 1.00 0.00 C

ATOM 1783 HA LEU 115 42.118 60.632 42.912 1.00 0.00 H

ATOM 1784 CB LEU 115 41.604 58.760 43.865 1.00 0.00 C

ATOM 1785 HB2 LEU 115 41.827 58.290 44.823 1.00 0.00 H

ATOM 1786 HB3 LEU 115 40.522 58.854 43.779 1.00 0.00 H

ATOM 1787 CG LEU 115 42.116 57.903 42.631 1.00 0.00 C

ATOM 1788 HG LEU 115 43.202 57.898 42.723 1.00 0.00 H

ATOM 1789 CD1 LEU 115 41.566 56.475 42.658 1.00 0.00 C

ATOM 1790 HD11 LEU 115 40.485 56.598 42.726 1.00 0.00 H

ATOM 1791 HD12 LEU 115 41.814 55.943 41.739 1.00 0.00 H

ATOM 1792 HD13 LEU 115 41.871 55.883 43.520 1.00 0.00 H

ATOM 1793 CD2 LEU 115 41.930 58.457 41.245 1.00 0.00 C

ATOM 1794 HD21 LEU 115 42.584 57.916 40.561 1.00 0.00 H

ATOM 1795 HD22 LEU 115 40.879 58.351 40.974 1.00 0.00 H

ATOM 1796 HD23 LEU 115 42.164 59.521 41.219 1.00 0.00 H

ATOM 1797 C LEU 115 41.348 61.127 44.734 1.00 0.00 C

ATOM 1798 O LEU 115 40.448 61.807 44.225 1.00 0.00 O

ATOM 1799 N SER 116 41.642 61.359 45.988 1.00 0.00 N

ATOM 1800 H SER 116 42.292 60.695 46.385 1.00 0.00 H

ATOM 1801 CA SER 116 40.955 62.371 46.832 1.00 0.00 C

ATOM 1802 HA SER 116 39.878 62.288 46.686 1.00 0.00 H

ATOM 1803 CB SER 116 41.220 62.116 48.341 1.00 0.00 C

ATOM 1804 HB2 SER 116 42.282 62.185 48.575 1.00 0.00 H

ATOM 1805 HB3 SER 116 40.687 62.868 48.923 1.00 0.00 H

ATOM 1806 OG SER 116 40.756 60.834 48.838 1.00 0.00 O

ATOM 1807 HG SER 116 41.331 60.143 48.503 1.00 0.00 H

ATOM 1808 C SER 116 41.401 63.767 46.449 1.00 0.00 C

ATOM 1809 O SER 116 40.528 64.675 46.276 1.00 0.00 O

ATOM 1810 N VAL 117 42.692 63.932 46.092 1.00 0.00 N

ATOM 1811 H VAL 117 43.330 63.161 46.230 1.00 0.00 H

ATOM 1812 CA VAL 117 43.223 65.229 45.589 1.00 0.00 C

ATOM 1813 HA VAL 117 42.957 65.917 46.391 1.00 0.00 H

ATOM 1814 CB VAL 117 44.754 65.389 45.599 1.00 0.00 C

ATOM 1815 HB VAL 117 45.250 64.633 44.989 1.00 0.00 H

ATOM 1816 CG1 VAL 117 45.220 66.709 44.944 1.00 0.00 C

ATOM 1817 HG11 VAL 117 46.298 66.850 45.024 1.00 0.00 H

ATOM 1818 HG12 VAL 117 45.048 66.695 43.868 1.00 0.00 H

ATOM 1819 HG13 VAL 117 44.764 67.563 45.444 1.00 0.00 H

ATOM 1820 CG2 VAL 117 45.310 65.285 46.970 1.00 0.00 C

ATOM 1821 HG21 VAL 117 46.384 65.425 47.090 1.00 0.00 H

ATOM 1822 HG22 VAL 117 44.855 66.095 47.540 1.00 0.00 H

ATOM 1823 HG23 VAL 117 45.067 64.341 47.457 1.00 0.00 H

ATOM 1824 C VAL 117 42.587 65.667 44.291 1.00 0.00 C

ATOM 1825 O VAL 117 42.160 66.778 44.197 1.00 0.00 O

ATOM 1826 N ILE 118 42.347 64.756 43.407 1.00 0.00 N

ATOM 1827 H ILE 118 42.637 63.817 43.640 1.00 0.00 H

ATOM 1828 CA ILE 118 41.682 64.983 42.149 1.00 0.00 C

ATOM 1829 HA ILE 118 42.228 65.664 41.497 1.00 0.00 H

ATOM 1830 CB ILE 118 41.871 63.598 41.336 1.00 0.00 C

ATOM 1831 HB ILE 118 41.669 62.749 41.988 1.00 0.00 H

ATOM 1832 CG2 ILE 118 40.929 63.578 40.114 1.00 0.00 C

ATOM 1833 HG21 ILE 118 41.021 62.597 39.646 1.00 0.00 H

ATOM 1834 HG22 ILE 118 39.924 63.717 40.512 1.00 0.00 H

ATOM 1835 HG23 ILE 118 41.181 64.364 39.402 1.00 0.00 H

ATOM 1836 CG1 ILE 118 43.343 63.514 40.760 1.00 0.00 C

ATOM 1837 HG12 ILE 118 43.632 64.207 39.969 1.00 0.00 H

ATOM 1838 HG13 ILE 118 43.976 63.805 41.598 1.00 0.00 H

ATOM 1839 CD1 ILE 118 43.885 62.123 40.441 1.00 0.00 C

ATOM 1840 HD11 ILE 118 43.270 61.568 39.732 1.00 0.00 H

ATOM 1841 HD12 ILE 118 44.879 62.157 39.996 1.00 0.00 H

ATOM 1842 HD13 ILE 118 44.109 61.579 41.359 1.00 0.00 H

ATOM 1843 C ILE 118 40.304 65.493 42.244 1.00 0.00 C

ATOM 1844 O ILE 118 39.996 66.539 41.669 1.00 0.00 O

ATOM 1845 N PHE 119 39.508 64.816 43.026 1.00 0.00 N

ATOM 1846 H PHE 119 39.993 64.055 43.481 1.00 0.00 H

ATOM 1847 CA PHE 119 38.118 65.149 43.253 1.00 0.00 C

ATOM 1848 HA PHE 119 37.658 65.430 42.305 1.00 0.00 H

ATOM 1849 CB PHE 119 37.470 63.901 43.831 1.00 0.00 C

ATOM 1850 HB2 PHE 119 36.964 63.351 43.038 1.00 0.00 H

ATOM 1851 HB3 PHE 119 38.222 63.173 44.135 1.00 0.00 H

ATOM 1852 CG PHE 119 36.377 64.108 44.901 1.00 0.00 C

ATOM 1853 CD1 PHE 119 35.060 64.610 44.515 1.00 0.00 C

ATOM 1854 HD1 PHE 119 34.742 64.857 43.513 1.00 0.00 H

ATOM 1855 CE1 PHE 119 34.136 64.713 45.598 1.00 0.00 C

ATOM 1856 HE1 PHE 119 33.105 64.929 45.358 1.00 0.00 H

ATOM 1857 CZ PHE 119 34.527 64.460 46.938 1.00 0.00 C

ATOM 1858 HZ PHE 119 33.790 64.527 47.724 1.00 0.00 H

ATOM 1859 CE2 PHE 119 35.774 64.060 47.234 1.00 0.00 C

ATOM 1860 HE2 PHE 119 36.105 63.805 48.230 1.00 0.00 H

ATOM 1861 CD2 PHE 119 36.727 63.854 46.208 1.00 0.00 C

ATOM 1862 HD2 PHE 119 37.773 63.714 46.438 1.00 0.00 H

ATOM 1863 C PHE 119 37.973 66.326 44.195 1.00 0.00 C

ATOM 1864 O PHE 119 36.978 66.996 44.063 1.00 0.00 O

ATOM 1865 N ILE 120 38.972 66.654 45.022 1.00 0.00 N

ATOM 1866 H ILE 120 39.713 65.993 45.205 1.00 0.00 H

ATOM 1867 CA ILE 120 38.881 67.859 45.838 1.00 0.00 C

ATOM 1868 HA ILE 120 37.837 67.865 46.149 1.00 0.00 H

ATOM 1869 CB ILE 120 39.776 67.704 47.085 1.00 0.00 C

ATOM 1870 HB ILE 120 40.688 67.142 46.883 1.00 0.00 H

ATOM 1871 CG2 ILE 120 40.267 69.019 47.656 1.00 0.00 C

ATOM 1872 HG21 ILE 120 40.921 68.781 48.495 1.00 0.00 H

ATOM 1873 HG22 ILE 120 40.919 69.594 46.999 1.00 0.00 H

ATOM 1874 HG23 ILE 120 39.469 69.708 47.935 1.00 0.00 H

ATOM 1875 CG1 ILE 120 39.108 66.736 48.087 1.00 0.00 C

ATOM 1876 HG12 ILE 120 38.919 65.794 47.572 1.00 0.00 H

ATOM 1877 HG13 ILE 120 39.889 66.590 48.834 1.00 0.00 H

ATOM 1878 CD1 ILE 120 37.777 67.148 48.738 1.00 0.00 C

ATOM 1879 HD11 ILE 120 36.850 67.069 48.170 1.00 0.00 H

ATOM 1880 HD12 ILE 120 37.755 66.489 49.606 1.00 0.00 H

ATOM 1881 HD13 ILE 120 37.799 68.162 49.138 1.00 0.00 H

ATOM 1882 C ILE 120 39.198 69.172 45.061 1.00 0.00 C

ATOM 1883 O ILE 120 38.514 70.166 45.140 1.00 0.00 O

ATOM 1884 N LYS 121 40.221 69.056 44.217 1.00 0.00 N

ATOM 1885 H LYS 121 40.688 68.163 44.275 1.00 0.00 H

ATOM 1886 CA LYS 121 40.547 70.067 43.179 1.00 0.00 C

ATOM 1887 HA LYS 121 40.670 70.989 43.748 1.00 0.00 H

ATOM 1888 CB LYS 121 41.908 69.754 42.575 1.00 0.00 C

ATOM 1889 HB2 LYS 121 41.766 68.777 42.115 1.00 0.00 H

ATOM 1890 HB3 LYS 121 42.048 70.328 41.659 1.00 0.00 H

ATOM 1891 CG LYS 121 43.221 69.859 43.445 1.00 0.00 C

ATOM 1892 HG2 LYS 121 43.128 69.303 44.378 1.00 0.00 H

ATOM 1893 HG3 LYS 121 44.052 69.395 42.911 1.00 0.00 H

ATOM 1894 CD LYS 121 43.555 71.272 43.929 1.00 0.00 C

ATOM 1895 HD2 LYS 121 42.831 71.503 44.711 1.00 0.00 H

ATOM 1896 HD3 LYS 121 44.492 71.421 44.466 1.00 0.00 H

ATOM 1897 CE LYS 121 43.407 72.375 42.881 1.00 0.00 C

ATOM 1898 HE2 LYS 121 44.170 72.236 42.115 1.00 0.00 H

ATOM 1899 HE3 LYS 121 42.479 72.165 42.349 1.00 0.00 H

ATOM 1900 NZ LYS 121 43.326 73.787 43.415 1.00 0.00 N

ATOM 1901 HZ1 LYS 121 43.372 74.463 42.666 1.00 0.00 H

ATOM 1902 HZ2 LYS 121 42.506 73.916 43.990 1.00 0.00 H

ATOM 1903 HZ3 LYS 121 44.089 73.973 44.050 1.00 0.00 H

ATOM 1904 C LYS 121 39.428 70.304 42.172 1.00 0.00 C

ATOM 1905 O LYS 121 39.084 71.423 41.998 1.00 0.00 O

ATOM 1906 N GLY 122 38.811 69.194 41.783 1.00 0.00 N

ATOM 1907 H GLY 122 39.251 68.305 41.974 1.00 0.00 H

ATOM 1908 CA GLY 122 37.489 69.193 41.111 1.00 0.00 C

ATOM 1909 HA2 GLY 122 36.700 69.629 41.723 1.00 0.00 H

ATOM 1910 HA3 GLY 122 37.590 69.865 40.258 1.00 0.00 H

ATOM 1911 C GLY 122 37.170 67.849 40.529 1.00 0.00 C

ATOM 1912 O GLY 122 36.536 67.001 41.167 1.00 0.00 O

ATOM 1913 N ASN 123 37.745 67.651 39.348 1.00 0.00 N

ATOM 1914 H ASN 123 38.375 68.387 39.063 1.00 0.00 H

ATOM 1915 CA ASN 123 37.609 66.502 38.432 1.00 0.00 C

ATOM 1916 HA ASN 123 37.044 65.700 38.906 1.00 0.00 H

ATOM 1917 CB ASN 123 36.823 66.900 37.166 1.00 0.00 C

ATOM 1918 HB2 ASN 123 36.709 67.977 37.051 1.00 0.00 H

ATOM 1919 HB3 ASN 123 37.212 66.469 36.243 1.00 0.00 H

ATOM 1920 CG ASN 123 35.373 66.340 37.310 1.00 0.00 C

ATOM 1921 OD1 ASN 123 34.720 66.636 38.301 1.00 0.00 O

ATOM 1922 ND2 ASN 123 34.940 65.537 36.380 1.00 0.00 N

ATOM 1923 HD21 ASN 123 33.967 65.272 36.441 1.00 0.00 H

ATOM 1924 HD22 ASN 123 35.360 65.500 35.462 1.00 0.00 H

ATOM 1925 C ASN 123 39.025 66.009 38.143 1.00 0.00 C

ATOM 1926 O ASN 123 39.202 64.817 37.747 1.00 0.00 O

ATOM 1927 N CYS 124 40.037 66.873 38.201 1.00 0.00 N

ATOM 1928 H CYS 124 39.682 67.799 38.395 1.00 0.00 H

ATOM 1929 CA CYS 124 41.458 66.809 37.840 1.00 0.00 C

ATOM 1930 HA CYS 124 41.653 65.740 37.755 1.00 0.00 H

ATOM 1931 CB CYS 124 41.803 67.392 36.489 1.00 0.00 C

ATOM 1932 HB2 CYS 124 41.816 68.477 36.592 1.00 0.00 H

ATOM 1933 HB3 CYS 124 42.735 66.944 36.145 1.00 0.00 H

ATOM 1934 SG CYS 124 40.558 66.948 35.220 1.00 0.00 S

ATOM 1935 HG CYS 124 39.499 67.570 35.745 1.00 0.00 H

ATOM 1936 C CYS 124 42.506 67.367 38.903 1.00 0.00 C

ATOM 1937 O CYS 124 42.184 68.246 39.640 1.00 0.00 O

ATOM 1938 N ALA 125 43.791 66.845 38.837 1.00 0.00 N

ATOM 1939 H ALA 125 43.894 66.257 38.022 1.00 0.00 H

ATOM 1940 CA ALA 125 45.041 67.356 39.515 1.00 0.00 C

ATOM 1941 HA ALA 125 44.840 68.279 40.059 1.00 0.00 H

ATOM 1942 CB ALA 125 45.483 66.391 40.611 1.00 0.00 C

ATOM 1943 HB1 ALA 125 45.702 65.453 40.101 1.00 0.00 H

ATOM 1944 HB2 ALA 125 46.409 66.768 41.045 1.00 0.00 H

ATOM 1945 HB3 ALA 125 44.672 66.268 41.329 1.00 0.00 H

ATOM 1946 C ALA 125 46.218 67.520 38.545 1.00 0.00 C

ATOM 1947 O ALA 125 46.222 66.787 37.580 1.00 0.00 O

ATOM 1948 N SER 126 47.212 68.418 38.920 1.00 0.00 N

ATOM 1949 H SER 126 47.123 68.888 39.809 1.00 0.00 H

ATOM 1950 CA SER 126 48.217 68.858 37.926 1.00 0.00 C

ATOM 1951 HA SER 126 48.060 68.239 37.043 1.00 0.00 H

ATOM 1952 CB SER 126 47.831 70.251 37.431 1.00 0.00 C

ATOM 1953 HB2 SER 126 48.357 70.389 36.486 1.00 0.00 H

ATOM 1954 HB3 SER 126 46.766 70.297 37.205 1.00 0.00 H

ATOM 1955 OG SER 126 48.216 71.169 38.360 1.00 0.00 O

ATOM 1956 HG SER 126 48.610 71.929 37.925 1.00 0.00 H

ATOM 1957 C SER 126 49.602 68.897 38.439 1.00 0.00 C

ATOM 1958 O SER 126 49.850 68.672 39.616 1.00 0.00 O

ATOM 1959 N GLU 127 50.606 69.251 37.601 1.00 0.00 N

ATOM 1960 H GLU 127 50.383 69.427 36.632 1.00 0.00 H

ATOM 1961 CA GLU 127 52.039 69.311 37.974 1.00 0.00 C

ATOM 1962 HA GLU 127 52.177 68.324 38.415 1.00 0.00 H

ATOM 1963 CB GLU 127 52.958 69.450 36.827 1.00 0.00 C

ATOM 1964 HB2 GLU 127 52.719 70.444 36.449 1.00 0.00 H

ATOM 1965 HB3 GLU 127 54.027 69.469 37.037 1.00 0.00 H

ATOM 1966 CG GLU 127 52.827 68.444 35.691 1.00 0.00 C

ATOM 1967 HG2 GLU 127 51.817 68.158 35.399 1.00 0.00 H

ATOM 1968 HG3 GLU 127 53.283 68.906 34.815 1.00 0.00 H

ATOM 1969 CD GLU 127 53.634 67.172 36.014 1.00 0.00 C

ATOM 1970 OE1 GLU 127 53.049 66.190 36.484 1.00 0.00 O

ATOM 1971 OE2 GLU 127 54.881 67.247 35.871 1.00 0.00 O

ATOM 1972 C GLU 127 52.246 70.336 39.179 1.00 0.00 C

ATOM 1973 O GLU 127 53.156 70.161 40.015 1.00 0.00 O

ATOM 1974 N GLU 128 51.333 71.354 39.400 1.00 0.00 N

ATOM 1975 H GLU 128 50.600 71.279 38.710 1.00 0.00 H

ATOM 1976 CA GLU 128 51.394 72.420 40.341 1.00 0.00 C

ATOM 1977 HA GLU 128 52.418 72.788 40.285 1.00 0.00 H

ATOM 1978 CB GLU 128 50.445 73.527 39.971 1.00 0.00 C

ATOM 1979 HB2 GLU 128 49.568 72.923 39.737 1.00 0.00 H

ATOM 1980 HB3 GLU 128 50.146 74.143 40.819 1.00 0.00 H

ATOM 1981 CG GLU 128 50.826 74.315 38.762 1.00 0.00 C

ATOM 1982 HG2 GLU 128 50.065 75.086 38.642 1.00 0.00 H

ATOM 1983 HG3 GLU 128 51.814 74.765 38.856 1.00 0.00 H

ATOM 1984 CD GLU 128 50.887 73.548 37.431 1.00 0.00 C

ATOM 1985 OE1 GLU 128 49.948 72.784 37.127 1.00 0.00 O

ATOM 1986 OE2 GLU 128 51.864 73.719 36.663 1.00 0.00 O

ATOM 1987 C GLU 128 51.036 71.881 41.742 1.00 0.00 C

ATOM 1988 O GLU 128 51.684 72.158 42.730 1.00 0.00 O

ATOM 1989 N VAL 129 50.062 70.958 41.912 1.00 0.00 N

ATOM 1990 H VAL 129 49.659 70.601 41.057 1.00 0.00 H

ATOM 1991 CA VAL 129 49.816 70.145 43.151 1.00 0.00 C

ATOM 1992 HA VAL 129 50.086 70.703 44.048 1.00 0.00 H

ATOM 1993 CB VAL 129 48.260 69.894 43.240 1.00 0.00 C

ATOM 1994 HB VAL 129 47.671 70.778 42.998 1.00 0.00 H

ATOM 1995 CG1 VAL 129 47.714 68.760 42.449 1.00 0.00 C

ATOM 1996 HG11 VAL 129 48.000 67.799 42.877 1.00 0.00 H

ATOM 1997 HG12 VAL 129 46.651 68.756 42.692 1.00 0.00 H

ATOM 1998 HG13 VAL 129 47.886 68.977 41.395 1.00 0.00 H

ATOM 1999 CG2 VAL 129 47.844 69.752 44.647 1.00 0.00 C

ATOM 2000 HG21 VAL 129 48.317 68.921 45.169 1.00 0.00 H

ATOM 2001 HG22 VAL 129 48.118 70.619 45.248 1.00 0.00 H

ATOM 2002 HG23 VAL 129 46.769 69.568 44.649 1.00 0.00 H

ATOM 2003 C VAL 129 50.551 68.778 43.311 1.00 0.00 C

ATOM 2004 O VAL 129 50.791 68.408 44.440 1.00 0.00 O

ATOM 2005 N ILE 130 50.775 68.043 42.243 1.00 0.00 N

ATOM 2006 H ILE 130 50.361 68.364 41.379 1.00 0.00 H

ATOM 2007 CA ILE 130 51.017 66.547 42.350 1.00 0.00 C

ATOM 2008 HA ILE 130 50.194 66.158 42.948 1.00 0.00 H

ATOM 2009 CB ILE 130 50.914 65.928 40.970 1.00 0.00 C

ATOM 2010 HB ILE 130 51.311 66.576 40.189 1.00 0.00 H

ATOM 2011 CG2 ILE 130 51.713 64.577 40.805 1.00 0.00 C

ATOM 2012 HG21 ILE 130 51.571 64.030 39.872 1.00 0.00 H

ATOM 2013 HG22 ILE 130 52.791 64.690 40.924 1.00 0.00 H

ATOM 2014 HG23 ILE 130 51.435 63.947 41.650 1.00 0.00 H

ATOM 2015 CG1 ILE 130 49.424 65.678 40.600 1.00 0.00 C

ATOM 2016 HG12 ILE 130 48.762 66.509 40.845 1.00 0.00 H

ATOM 2017 HG13 ILE 130 49.403 65.435 39.538 1.00 0.00 H

ATOM 2018 CD1 ILE 130 48.792 64.479 41.406 1.00 0.00 C

ATOM 2019 HD11 ILE 130 47.869 64.234 40.880 1.00 0.00 H

ATOM 2020 HD12 ILE 130 49.386 63.572 41.294 1.00 0.00 H

ATOM 2021 HD13 ILE 130 48.607 64.766 42.441 1.00 0.00 H

ATOM 2022 C ILE 130 52.384 66.163 42.987 1.00 0.00 C

ATOM 2023 O ILE 130 52.431 65.147 43.668 1.00 0.00 O

ATOM 2024 N TRP 131 53.420 66.999 42.684 1.00 0.00 N

ATOM 2025 H TRP 131 53.190 67.822 42.146 1.00 0.00 H

ATOM 2026 CA TRP 131 54.753 66.770 43.179 1.00 0.00 C

ATOM 2027 HA TRP 131 54.845 65.695 43.338 1.00 0.00 H

ATOM 2028 CB TRP 131 55.679 67.275 42.064 1.00 0.00 C

ATOM 2029 HB2 TRP 131 55.607 68.361 42.009 1.00 0.00 H

ATOM 2030 HB3 TRP 131 56.678 66.930 42.332 1.00 0.00 H

ATOM 2031 CG TRP 131 55.460 66.683 40.741 1.00 0.00 C

ATOM 2032 CD1 TRP 131 54.566 66.989 39.789 1.00 0.00 C

ATOM 2033 HD1 TRP 131 53.982 67.898 39.779 1.00 0.00 H

ATOM 2034 NE1 TRP 131 54.360 65.976 38.895 1.00 0.00 N

ATOM 2035 HE1 TRP 131 53.710 66.002 38.123 1.00 0.00 H

ATOM 2036 CE2 TRP 131 55.228 64.928 39.226 1.00 0.00 C

ATOM 2037 CZ2 TRP 131 55.231 63.603 38.802 1.00 0.00 C

ATOM 2038 HZ2 TRP 131 54.452 63.251 38.143 1.00 0.00 H

ATOM 2039 CH2 TRP 131 56.199 62.762 39.384 1.00 0.00 C

ATOM 2040 HH2 TRP 131 56.257 61.709 39.151 1.00 0.00 H

ATOM 2041 CZ3 TRP 131 57.149 63.233 40.278 1.00 0.00 C

ATOM 2042 HZ3 TRP 131 57.891 62.555 40.674 1.00 0.00 H

ATOM 2043 CE3 TRP 131 56.997 64.494 40.792 1.00 0.00 C

ATOM 2044 HE3 TRP 131 57.734 64.864 41.490 1.00 0.00 H

ATOM 2045 CD2 TRP 131 55.992 65.370 40.322 1.00 0.00 C

ATOM 2046 C TRP 131 55.028 67.355 44.531 1.00 0.00 C

ATOM 2047 O TRP 131 55.927 66.864 45.217 1.00 0.00 O

ATOM 2048 N GLU 132 54.139 68.199 45.013 1.00 0.00 N

ATOM 2049 H GLU 132 53.433 68.574 44.395 1.00 0.00 H

ATOM 2050 CA GLU 132 54.071 68.573 46.440 1.00 0.00 C

ATOM 2051 HA GLU 132 55.102 68.769 46.735 1.00 0.00 H

ATOM 2052 CB GLU 132 53.429 69.993 46.461 1.00 0.00 C

ATOM 2053 HB2 GLU 132 53.762 70.525 45.570 1.00 0.00 H

ATOM 2054 HB3 GLU 132 52.343 69.911 46.490 1.00 0.00 H

ATOM 2055 CG GLU 132 53.941 70.834 47.679 1.00 0.00 C

ATOM 2056 HG2 GLU 132 53.517 71.834 47.579 1.00 0.00 H

ATOM 2057 HG3 GLU 132 53.443 70.497 48.588 1.00 0.00 H

ATOM 2058 CD GLU 132 55.445 71.018 47.799 1.00 0.00 C

ATOM 2059 OE1 GLU 132 56.028 70.886 48.897 1.00 0.00 O

ATOM 2060 OE2 GLU 132 56.047 71.150 46.744 1.00 0.00 O

ATOM 2061 C GLU 132 53.386 67.535 47.316 1.00 0.00 C

ATOM 2062 O GLU 132 53.850 67.254 48.392 1.00 0.00 O

ATOM 2063 N VAL 133 52.366 66.781 46.858 1.00 0.00 N

ATOM 2064 H VAL 133 51.975 67.086 45.979 1.00 0.00 H

ATOM 2065 CA VAL 133 51.803 65.560 47.502 1.00 0.00 C

ATOM 2066 HA VAL 133 51.520 65.914 48.493 1.00 0.00 H

ATOM 2067 CB VAL 133 50.583 64.996 46.754 1.00 0.00 C

ATOM 2068 HB VAL 133 50.861 64.738 45.732 1.00 0.00 H

ATOM 2069 CG1 VAL 133 50.115 63.652 47.457 1.00 0.00 C

ATOM 2070 HG11 VAL 133 50.825 62.861 47.213 1.00 0.00 H

ATOM 2071 HG12 VAL 133 50.081 63.682 48.546 1.00 0.00 H

ATOM 2072 HG13 VAL 133 49.180 63.318 47.008 1.00 0.00 H

ATOM 2073 CG2 VAL 133 49.372 65.993 46.693 1.00 0.00 C

ATOM 2074 HG21 VAL 133 49.050 66.294 47.690 1.00 0.00 H

ATOM 2075 HG22 VAL 133 49.647 66.907 46.169 1.00 0.00 H

ATOM 2076 HG23 VAL 133 48.528 65.518 46.192 1.00 0.00 H

ATOM 2077 C VAL 133 52.947 64.498 47.637 1.00 0.00 C

ATOM 2078 O VAL 133 53.268 63.992 48.711 1.00 0.00 O

ATOM 2079 N LEU 134 53.764 64.301 46.579 1.00 0.00 N

ATOM 2080 H LEU 134 53.331 64.605 45.719 1.00 0.00 H

ATOM 2081 CA LEU 134 54.906 63.343 46.555 1.00 0.00 C

ATOM 2082 HA LEU 134 54.590 62.366 46.921 1.00 0.00 H

ATOM 2083 CB LEU 134 55.411 62.985 45.096 1.00 0.00 C

ATOM 2084 HB2 LEU 134 55.673 63.933 44.626 1.00 0.00 H

ATOM 2085 HB3 LEU 134 56.281 62.328 45.091 1.00 0.00 H

ATOM 2086 CG LEU 134 54.321 62.382 44.185 1.00 0.00 C

ATOM 2087 HG LEU 134 53.413 62.977 44.284 1.00 0.00 H

ATOM 2088 CD1 LEU 134 54.536 62.525 42.688 1.00 0.00 C

ATOM 2089 HD11 LEU 134 55.558 62.191 42.507 1.00 0.00 H

ATOM 2090 HD12 LEU 134 53.789 61.991 42.101 1.00 0.00 H

ATOM 2091 HD13 LEU 134 54.478 63.573 42.394 1.00 0.00 H

ATOM 2092 CD2 LEU 134 53.946 60.900 44.507 1.00 0.00 C

ATOM 2093 HD21 LEU 134 52.888 60.725 44.313 1.00 0.00 H

ATOM 2094 HD22 LEU 134 54.451 60.170 43.874 1.00 0.00 H

ATOM 2095 HD23 LEU 134 54.229 60.770 45.552 1.00 0.00 H

ATOM 2096 C LEU 134 56.051 63.800 47.469 1.00 0.00 C

ATOM 2097 O LEU 134 56.754 62.960 47.984 1.00 0.00 O

ATOM 2098 N ASN 135 56.237 65.108 47.543 1.00 0.00 N

ATOM 2099 H ASN 135 55.636 65.758 47.057 1.00 0.00 H

ATOM 2100 CA ASN 135 57.320 65.701 48.280 1.00 0.00 C

ATOM 2101 HA ASN 135 58.302 65.345 47.969 1.00 0.00 H

ATOM 2102 CB ASN 135 57.341 67.221 48.103 1.00 0.00 C

ATOM 2103 HB2 ASN 135 57.263 67.547 47.065 1.00 0.00 H

ATOM 2104 HB3 ASN 135 56.446 67.689 48.512 1.00 0.00 H

ATOM 2105 CG ASN 135 58.495 68.052 48.671 1.00 0.00 C

ATOM 2106 OD1 ASN 135 59.573 67.510 48.885 1.00 0.00 O

ATOM 2107 ND2 ASN 135 58.357 69.339 49.009 1.00 0.00 N

ATOM 2108 HD21 ASN 135 59.146 69.850 49.377 1.00 0.00 H

ATOM 2109 HD22 ASN 135 57.503 69.876 48.946 1.00 0.00 H

ATOM 2110 C ASN 135 57.124 65.441 49.866 1.00 0.00 C

ATOM 2111 O ASN 135 58.087 65.483 50.626 1.00 0.00 O

ATOM 2112 N ALA 136 55.880 65.246 50.321 1.00 0.00 N

ATOM 2113 H ALA 136 55.143 65.097 49.647 1.00 0.00 H

ATOM 2114 CA ALA 136 55.545 64.881 51.731 1.00 0.00 C

ATOM 2115 HA ALA 136 56.336 65.285 52.364 1.00 0.00 H

ATOM 2116 CB ALA 136 54.136 65.417 52.188 1.00 0.00 C

ATOM 2117 HB1 ALA 136 53.962 64.840 53.096 1.00 0.00 H

ATOM 2118 HB2 ALA 136 54.138 66.468 52.479 1.00 0.00 H

ATOM 2119 HB3 ALA 136 53.392 65.222 51.416 1.00 0.00 H

ATOM 2120 C ALA 136 55.703 63.347 51.929 1.00 0.00 C

ATOM 2121 O ALA 136 56.289 62.950 52.958 1.00 0.00 O

ATOM 2122 N VAL 137 55.397 62.507 50.960 1.00 0.00 N

ATOM 2123 H VAL 137 54.791 62.839 50.224 1.00 0.00 H

ATOM 2124 CA VAL 137 55.513 61.006 51.078 1.00 0.00 C

ATOM 2125 HA VAL 137 55.454 60.847 52.155 1.00 0.00 H

ATOM 2126 CB VAL 137 54.366 60.215 50.358 1.00 0.00 C

ATOM 2127 HB VAL 137 53.491 60.703 50.788 1.00 0.00 H

ATOM 2128 CG1 VAL 137 54.299 60.366 48.823 1.00 0.00 C

ATOM 2129 HG11 VAL 137 53.855 61.332 48.582 1.00 0.00 H

ATOM 2130 HG12 VAL 137 55.310 60.212 48.446 1.00 0.00 H

ATOM 2131 HG13 VAL 137 53.633 59.619 48.391 1.00 0.00 H

ATOM 2132 CG2 VAL 137 54.313 58.757 50.743 1.00 0.00 C

ATOM 2133 HG21 VAL 137 55.084 58.107 50.330 1.00 0.00 H

ATOM 2134 HG22 VAL 137 54.434 58.708 51.825 1.00 0.00 H

ATOM 2135 HG23 VAL 137 53.333 58.309 50.575 1.00 0.00 H

ATOM 2136 C VAL 137 56.901 60.492 50.717 1.00 0.00 C

ATOM 2137 O VAL 137 57.202 59.369 51.020 1.00 0.00 O

ATOM 2138 N GLY 138 57.835 61.322 50.378 1.00 0.00 N

ATOM 2139 H GLY 138 57.490 62.173 49.958 1.00 0.00 H

ATOM 2140 CA GLY 138 59.260 61.049 50.175 1.00 0.00 C

ATOM 2141 HA2 GLY 138 59.845 61.963 50.275 1.00 0.00 H

ATOM 2142 HA3 GLY 138 59.606 60.401 50.981 1.00 0.00 H

ATOM 2143 C GLY 138 59.621 60.518 48.728 1.00 0.00 C

ATOM 2144 O GLY 138 60.630 59.783 48.508 1.00 0.00 O

ATOM 2145 N VAL 139 58.708 60.613 47.764 1.00 0.00 N

ATOM 2146 H VAL 139 57.818 61.064 47.924 1.00 0.00 H

ATOM 2147 CA VAL 139 58.686 60.128 46.406 1.00 0.00 C

ATOM 2148 HA VAL 139 59.490 59.403 46.281 1.00 0.00 H

ATOM 2149 CB VAL 139 57.324 59.446 46.085 1.00 0.00 C

ATOM 2150 HB VAL 139 56.536 60.197 46.139 1.00 0.00 H

ATOM 2151 CG1 VAL 139 57.350 58.704 44.680 1.00 0.00 C

ATOM 2152 HG11 VAL 139 56.326 58.386 44.487 1.00 0.00 H

ATOM 2153 HG12 VAL 139 57.774 59.341 43.904 1.00 0.00 H

ATOM 2154 HG13 VAL 139 58.006 57.857 44.883 1.00 0.00 H

ATOM 2155 CG2 VAL 139 57.000 58.307 47.134 1.00 0.00 C

ATOM 2156 HG21 VAL 139 56.734 58.768 48.085 1.00 0.00 H

ATOM 2157 HG22 VAL 139 56.104 57.777 46.812 1.00 0.00 H

ATOM 2158 HG23 VAL 139 57.865 57.655 47.260 1.00 0.00 H

ATOM 2159 C VAL 139 59.010 61.226 45.394 1.00 0.00 C

ATOM 2160 O VAL 139 58.509 62.364 45.525 1.00 0.00 O

ATOM 2161 N TYR 140 59.947 60.974 44.466 1.00 0.00 N

ATOM 2162 H TYR 140 60.333 60.041 44.492 1.00 0.00 H

ATOM 2163 CA TYR 140 60.473 61.911 43.515 1.00 0.00 C

ATOM 2164 HA TYR 140 59.656 62.592 43.278 1.00 0.00 H

ATOM 2165 CB TYR 140 61.741 62.551 44.125 1.00 0.00 C

ATOM 2166 HB2 TYR 140 62.541 61.817 44.229 1.00 0.00 H

ATOM 2167 HB3 TYR 140 62.070 63.279 43.384 1.00 0.00 H

ATOM 2168 CG TYR 140 61.619 63.184 45.547 1.00 0.00 C

ATOM 2169 CD1 TYR 140 61.079 64.485 45.606 1.00 0.00 C

ATOM 2170 HD1 TYR 140 60.696 65.063 44.778 1.00 0.00 H

ATOM 2171 CE1 TYR 140 60.968 65.169 46.831 1.00 0.00 C

ATOM 2172 HE1 TYR 140 60.724 66.221 46.813 1.00 0.00 H

ATOM 2173 CZ TYR 140 61.240 64.533 48.050 1.00 0.00 C

ATOM 2174 OH TYR 140 60.963 65.156 49.208 1.00 0.00 O

ATOM 2175 HH TYR 140 60.371 65.904 49.097 1.00 0.00 H

ATOM 2176 CE2 TYR 140 61.874 63.256 48.003 1.00 0.00 C

ATOM 2177 HE2 TYR 140 62.135 62.814 48.954 1.00 0.00 H

ATOM 2178 CD2 TYR 140 62.034 62.562 46.764 1.00 0.00 C

ATOM 2179 HD2 TYR 140 62.428 61.564 46.645 1.00 0.00 H

ATOM 2180 C TYR 140 60.860 61.192 42.204 1.00 0.00 C

ATOM 2181 O TYR 140 61.153 59.988 42.196 1.00 0.00 O

ATOM 2182 N ALA 141 60.921 61.910 41.102 1.00 0.00 N

ATOM 2183 H ALA 141 60.535 62.840 41.179 1.00 0.00 H

ATOM 2184 CA ALA 141 61.230 61.463 39.771 1.00 0.00 C

ATOM 2185 HA ALA 141 60.419 60.838 39.398 1.00 0.00 H

ATOM 2186 CB ALA 141 61.103 62.581 38.786 1.00 0.00 C

ATOM 2187 HB1 ALA 141 60.038 62.705 38.593 1.00 0.00 H

ATOM 2188 HB2 ALA 141 61.590 63.478 39.169 1.00 0.00 H

ATOM 2189 HB3 ALA 141 61.585 62.284 37.854 1.00 0.00 H

ATOM 2190 C ALA 141 62.589 60.789 39.731 1.00 0.00 C

ATOM 2191 O ALA 141 63.621 61.360 39.973 1.00 0.00 O

ATOM 2192 N GLY 142 62.516 59.520 39.377 1.00 0.00 N

ATOM 2193 H GLY 142 61.632 59.116 39.104 1.00 0.00 H

ATOM 2194 CA GLY 142 63.798 58.789 39.100 1.00 0.00 C

ATOM 2195 HA2 GLY 142 63.517 57.835 38.653 1.00 0.00 H

ATOM 2196 HA3 GLY 142 64.470 59.341 38.442 1.00 0.00 H

ATOM 2197 C GLY 142 64.556 58.562 40.358 1.00 0.00 C

ATOM 2198 O GLY 142 65.784 58.336 40.272 1.00 0.00 O

ATOM 2199 N ARG 143 63.854 58.491 41.565 1.00 0.00 N

ATOM 2200 H ARG 143 62.863 58.682 41.522 1.00 0.00 H

ATOM 2201 CA ARG 143 64.432 58.078 42.831 1.00 0.00 C

ATOM 2202 HA ARG 143 65.506 57.925 42.729 1.00 0.00 H

ATOM 2203 CB ARG 143 64.303 59.155 43.927 1.00 0.00 C

ATOM 2204 HB2 ARG 143 63.264 59.471 44.016 1.00 0.00 H

ATOM 2205 HB3 ARG 143 64.600 58.746 44.892 1.00 0.00 H

ATOM 2206 CG ARG 143 65.270 60.360 43.748 1.00 0.00 C

ATOM 2207 HG2 ARG 143 65.195 60.973 44.646 1.00 0.00 H

ATOM 2208 HG3 ARG 143 66.270 60.006 43.495 1.00 0.00 H

ATOM 2209 CD ARG 143 64.863 61.283 42.564 1.00 0.00 C

ATOM 2210 HD2 ARG 143 65.208 60.851 41.625 1.00 0.00 H

ATOM 2211 HD3 ARG 143 63.801 61.525 42.524 1.00 0.00 H

ATOM 2212 NE ARG 143 65.456 62.624 42.718 1.00 0.00 N

ATOM 2213 HE ARG 143 65.812 62.852 43.635 1.00 0.00 H

ATOM 2214 CZ ARG 143 65.450 63.642 41.870 1.00 0.00 C

ATOM 2215 NH1 ARG 143 65.021 63.646 40.633 1.00 0.00 N

ATOM 2216 HH11 ARG 143 64.650 62.762 40.316 1.00 0.00 H

ATOM 2217 HH12 ARG 143 65.098 64.434 40.007 1.00 0.00 H

ATOM 2218 NH2 ARG 143 65.887 64.778 42.236 1.00 0.00 N

ATOM 2219 HH21 ARG 143 66.351 64.808 43.133 1.00 0.00 H

ATOM 2220 HH22 ARG 143 65.863 65.526 41.558 1.00 0.00 H

ATOM 2221 C ARG 143 63.769 56.817 43.316 1.00 0.00 C

ATOM 2222 O ARG 143 62.693 56.420 42.873 1.00 0.00 O

ATOM 2223 N GLU 144 64.432 56.045 44.239 1.00 0.00 N

ATOM 2224 H GLU 144 65.316 56.436 44.532 1.00 0.00 H

ATOM 2225 CA GLU 144 63.804 54.980 45.061 1.00 0.00 C

ATOM 2226 HA GLU 144 63.187 54.267 44.515 1.00 0.00 H

ATOM 2227 CB GLU 144 64.976 54.133 45.593 1.00 0.00 C

ATOM 2228 HB2 GLU 144 65.670 54.861 46.014 1.00 0.00 H

ATOM 2229 HB3 GLU 144 64.519 53.547 46.391 1.00 0.00 H

ATOM 2230 CG GLU 144 65.663 53.219 44.449 1.00 0.00 C

ATOM 2231 HG2 GLU 144 64.987 52.544 43.925 1.00 0.00 H

ATOM 2232 HG3 GLU 144 66.079 53.891 43.699 1.00 0.00 H

ATOM 2233 CD GLU 144 66.850 52.455 45.001 1.00 0.00 C

ATOM 2234 OE1 GLU 144 67.847 53.076 45.427 1.00 0.00 O

ATOM 2235 OE2 GLU 144 66.795 51.182 45.222 1.00 0.00 O

ATOM 2236 C GLU 144 62.874 55.486 46.193 1.00 0.00 C

ATOM 2237 O GLU 144 63.112 56.586 46.726 1.00 0.00 O

ATOM 2238 N HIE 145 61.891 54.658 46.686 1.00 0.00 N

ATOM 2239 H HIE 145 61.819 53.747 46.256 1.00 0.00 H

ATOM 2240 CA HIE 145 61.078 54.874 47.897 1.00 0.00 C

ATOM 2241 HA HIE 145 61.690 55.451 48.590 1.00 0.00 H

ATOM 2242 CB HIE 145 59.773 55.532 47.558 1.00 0.00 C

ATOM 2243 HB2 HIE 145 60.180 56.340 46.949 1.00 0.00 H

ATOM 2244 HB3 HIE 145 59.204 54.851 46.927 1.00 0.00 H

ATOM 2245 CG HIE 145 58.942 55.912 48.758 1.00 0.00 C

ATOM 2246 ND1 HIE 145 57.835 55.190 49.161 1.00 0.00 N

ATOM 2247 CE1 HIE 145 57.360 55.734 50.318 1.00 0.00 C

ATOM 2248 HE1 HIE 145 56.449 55.544 50.867 1.00 0.00 H

ATOM 2249 NE2 HIE 145 58.225 56.681 50.683 1.00 0.00 N

ATOM 2250 HE2 HIE 145 57.998 57.421 51.332 1.00 0.00 H

ATOM 2251 CD2 HIE 145 59.144 56.912 49.676 1.00 0.00 C

ATOM 2252 HD2 HIE 145 59.980 57.595 49.679 1.00 0.00 H

ATOM 2253 C HIE 145 60.737 53.503 48.555 1.00 0.00 C

ATOM 2254 O HIE 145 60.635 52.478 47.967 1.00 0.00 O

ATOM 2255 N PHE 146 60.498 53.523 49.884 1.00 0.00 N

ATOM 2256 H PHE 146 60.612 54.461 50.238 1.00 0.00 H

ATOM 2257 CA PHE 146 60.107 52.353 50.679 1.00 0.00 C

ATOM 2258 HA PHE 146 60.943 51.659 50.768 1.00 0.00 H

ATOM 2259 CB PHE 146 59.609 52.737 52.046 1.00 0.00 C

ATOM 2260 HB2 PHE 146 60.123 53.687 52.191 1.00 0.00 H

ATOM 2261 HB3 PHE 146 58.545 52.970 52.058 1.00 0.00 H

ATOM 2262 CG PHE 146 60.011 51.756 53.172 1.00 0.00 C

ATOM 2263 CD1 PHE 146 61.180 51.983 53.922 1.00 0.00 C

ATOM 2264 HD1 PHE 146 61.943 52.670 53.587 1.00 0.00 H

ATOM 2265 CE1 PHE 146 61.450 51.291 55.137 1.00 0.00 C

ATOM 2266 HE1 PHE 146 62.281 51.563 55.771 1.00 0.00 H

ATOM 2267 CZ PHE 146 60.565 50.267 55.498 1.00 0.00 C

ATOM 2268 HZ PHE 146 60.945 49.733 56.356 1.00 0.00 H

ATOM 2269 CE2 PHE 146 59.450 49.859 54.709 1.00 0.00 C

ATOM 2270 HE2 PHE 146 58.863 49.008 55.020 1.00 0.00 H

ATOM 2271 CD2 PHE 146 59.178 50.675 53.553 1.00 0.00 C

ATOM 2272 HD2 PHE 146 58.177 50.561 53.166 1.00 0.00 H

ATOM 2273 C PHE 146 58.995 51.453 50.021 1.00 0.00 C

ATOM 2274 O PHE 146 59.004 50.234 50.231 1.00 0.00 O

ATOM 2275 N VAL 147 58.023 51.978 49.190 1.00 0.00 N

ATOM 2276 H VAL 147 58.098 52.939 48.889 1.00 0.00 H

ATOM 2277 CA VAL 147 57.077 51.103 48.507 1.00 0.00 C

ATOM 2278 HA VAL 147 57.612 50.169 48.338 1.00 0.00 H

ATOM 2279 CB VAL 147 55.746 50.986 49.308 1.00 0.00 C

ATOM 2280 HB VAL 147 55.228 51.938 49.195 1.00 0.00 H

ATOM 2281 CG1 VAL 147 54.895 49.898 48.821 1.00 0.00 C

ATOM 2282 HG11 VAL 147 53.937 49.933 49.340 1.00 0.00 H

ATOM 2283 HG12 VAL 147 54.784 49.956 47.738 1.00 0.00 H

ATOM 2284 HG13 VAL 147 55.311 48.913 49.033 1.00 0.00 H

ATOM 2285 CG2 VAL 147 55.869 50.848 50.845 1.00 0.00 C

ATOM 2286 HG21 VAL 147 56.371 49.921 51.123 1.00 0.00 H

ATOM 2287 HG22 VAL 147 56.400 51.722 51.222 1.00 0.00 H

ATOM 2288 HG23 VAL 147 54.851 50.868 51.234 1.00 0.00 H

ATOM 2289 C VAL 147 56.725 51.592 47.083 1.00 0.00 C

ATOM 2290 O VAL 147 56.502 50.722 46.219 1.00 0.00 O

ATOM 2291 N TYR 148 56.763 52.895 46.834 1.00 0.00 N

ATOM 2292 H TYR 148 56.790 53.581 47.574 1.00 0.00 H

ATOM 2293 CA TYR 148 56.127 53.482 45.649 1.00 0.00 C

ATOM 2294 HA TYR 148 55.383 52.745 45.347 1.00 0.00 H

ATOM 2295 CB TYR 148 55.260 54.680 46.048 1.00 0.00 C

ATOM 2296 HB2 TYR 148 55.807 55.616 46.160 1.00 0.00 H

ATOM 2297 HB3 TYR 148 54.452 54.682 45.317 1.00 0.00 H

ATOM 2298 CG TYR 148 54.525 54.392 47.303 1.00 0.00 C

ATOM 2299 CD1 TYR 148 53.681 53.240 47.380 1.00 0.00 C

ATOM 2300 HD1 TYR 148 53.544 52.589 46.530 1.00 0.00 H

ATOM 2301 CE1 TYR 148 52.877 53.091 48.551 1.00 0.00 C

ATOM 2302 HE1 TYR 148 52.210 52.247 48.646 1.00 0.00 H

ATOM 2303 CZ TYR 148 52.912 54.109 49.534 1.00 0.00 C

ATOM 2304 OH TYR 148 52.242 53.955 50.719 1.00 0.00 O

ATOM 2305 HH TYR 148 51.860 53.085 50.857 1.00 0.00 H

ATOM 2306 CE2 TYR 148 53.841 55.138 49.531 1.00 0.00 C

ATOM 2307 HE2 TYR 148 53.805 55.771 50.405 1.00 0.00 H

ATOM 2308 CD2 TYR 148 54.653 55.274 48.390 1.00 0.00 C

ATOM 2309 HD2 TYR 148 55.260 56.166 48.335 1.00 0.00 H

ATOM 2310 C TYR 148 57.060 53.635 44.496 1.00 0.00 C

ATOM 2311 O TYR 148 56.736 54.414 43.589 1.00 0.00 O

ATOM 2312 N GLY 149 58.221 52.955 44.457 1.00 0.00 N

ATOM 2313 H GLY 149 58.217 52.205 45.134 1.00 0.00 H

ATOM 2314 CA GLY 149 59.217 52.958 43.310 1.00 0.00 C

ATOM 2315 HA2 GLY 149 60.162 52.589 43.710 1.00 0.00 H

ATOM 2316 HA3 GLY 149 58.913 52.204 42.584 1.00 0.00 H

ATOM 2317 C GLY 149 59.455 54.344 42.630 1.00 0.00 C

ATOM 2318 O GLY 149 59.690 55.356 43.323 1.00 0.00 O

ATOM 2319 N GLU 150 59.251 54.469 41.314 1.00 0.00 N

ATOM 2320 H GLU 150 58.939 53.640 40.830 1.00 0.00 H

ATOM 2321 CA GLU 150 59.598 55.646 40.468 1.00 0.00 C

ATOM 2322 HA GLU 150 60.065 56.374 41.132 1.00 0.00 H

ATOM 2323 CB GLU 150 60.557 55.227 39.323 1.00 0.00 C

ATOM 2324 HB2 GLU 150 61.392 54.618 39.670 1.00 0.00 H

ATOM 2325 HB3 GLU 150 59.984 54.483 38.771 1.00 0.00 H

ATOM 2326 CG GLU 150 60.997 56.365 38.429 1.00 0.00 C

ATOM 2327 HG2 GLU 150 61.799 56.899 38.939 1.00 0.00 H

ATOM 2328 HG3 GLU 150 61.519 55.887 37.600 1.00 0.00 H

ATOM 2329 CD GLU 150 59.961 57.257 37.834 1.00 0.00 C

ATOM 2330 OE1 GLU 150 59.256 56.839 36.923 1.00 0.00 O

ATOM 2331 OE2 GLU 150 60.043 58.444 38.281 1.00 0.00 O

ATOM 2332 C GLU 150 58.201 56.207 40.103 1.00 0.00 C

ATOM 2333 O GLU 150 57.398 55.573 39.423 1.00 0.00 O

ATOM 2334 N PRO 151 57.826 57.392 40.558 1.00 0.00 N

ATOM 2335 CD PRO 151 58.552 58.406 41.379 1.00 0.00 C

ATOM 2336 HD2 PRO 151 59.599 58.514 41.096 1.00 0.00 H

ATOM 2337 HD3 PRO 151 58.640 58.063 42.410 1.00 0.00 H

ATOM 2338 CG PRO 151 57.880 59.808 41.258 1.00 0.00 C

ATOM 2339 HG2 PRO 151 58.269 60.237 40.334 1.00 0.00 H

ATOM 2340 HG3 PRO 151 58.111 60.500 42.067 1.00 0.00 H

ATOM 2341 CB PRO 151 56.459 59.305 41.178 1.00 0.00 C

ATOM 2342 HB2 PRO 151 55.932 60.082 40.624 1.00 0.00 H

ATOM 2343 HB3 PRO 151 56.072 59.217 42.194 1.00 0.00 H

ATOM 2344 CA PRO 151 56.452 57.946 40.410 1.00 0.00 C

ATOM 2345 HA PRO 151 55.801 57.212 40.884 1.00 0.00 H

ATOM 2346 C PRO 151 55.981 58.247 38.996 1.00 0.00 C

ATOM 2347 O PRO 151 54.793 58.166 38.737 1.00 0.00 O

ATOM 2348 N ARG 152 56.873 58.735 38.138 1.00 0.00 N

ATOM 2349 H ARG 152 57.855 58.572 38.311 1.00 0.00 H

ATOM 2350 CA ARG 152 56.455 59.192 36.822 1.00 0.00 C

ATOM 2351 HA ARG 152 55.661 59.906 37.039 1.00 0.00 H

ATOM 2352 CB ARG 152 57.526 60.107 36.162 1.00 0.00 C

ATOM 2353 HB2 ARG 152 57.780 60.918 36.844 1.00 0.00 H

ATOM 2354 HB3 ARG 152 58.447 59.527 36.127 1.00 0.00 H

ATOM 2355 CG ARG 152 57.193 60.561 34.682 1.00 0.00 C

ATOM 2356 HG2 ARG 152 58.013 61.011 34.122 1.00 0.00 H

ATOM 2357 HG3 ARG 152 56.791 59.726 34.110 1.00 0.00 H

ATOM 2358 CD ARG 152 56.018 61.606 34.608 1.00 0.00 C

ATOM 2359 HD2 ARG 152 55.825 61.747 33.545 1.00 0.00 H

ATOM 2360 HD3 ARG 152 55.060 61.364 35.068 1.00 0.00 H

ATOM 2361 NE ARG 152 56.458 62.874 35.254 1.00 0.00 N

ATOM 2362 HE ARG 152 57.427 63.101 35.423 1.00 0.00 H

ATOM 2363 CZ ARG 152 55.699 63.868 35.643 1.00 0.00 C

ATOM 2364 NH1 ARG 152 54.402 63.943 35.504 1.00 0.00 N

ATOM 2365 HH11 ARG 152 54.024 63.237 34.888 1.00 0.00 H

ATOM 2366 HH12 ARG 152 53.833 64.732 35.774 1.00 0.00 H

ATOM 2367 NH2 ARG 152 56.207 64.942 36.145 1.00 0.00 N

ATOM 2368 HH21 ARG 152 57.196 65.026 36.330 1.00 0.00 H

ATOM 2369 HH22 ARG 152 55.604 65.747 36.245 1.00 0.00 H

ATOM 2370 C ARG 152 55.831 58.066 35.902 1.00 0.00 C

ATOM 2371 O ARG 152 54.861 58.367 35.197 1.00 0.00 O

ATOM 2372 N GLU 153 56.417 56.875 35.932 1.00 0.00 N

ATOM 2373 H GLU 153 57.356 56.800 36.297 1.00 0.00 H

ATOM 2374 CA GLU 153 55.852 55.643 35.424 1.00 0.00 C

ATOM 2375 HA GLU 153 55.561 55.805 34.386 1.00 0.00 H

ATOM 2376 CB GLU 153 56.979 54.587 35.214 1.00 0.00 C

ATOM 2377 HB2 GLU 153 57.786 55.086 34.678 1.00 0.00 H

ATOM 2378 HB3 GLU 153 57.278 54.164 36.173 1.00 0.00 H

ATOM 2379 CG GLU 153 56.543 53.356 34.411 1.00 0.00 C

ATOM 2380 HG2 GLU 153 55.858 52.736 34.990 1.00 0.00 H

ATOM 2381 HG3 GLU 153 55.944 53.527 33.517 1.00 0.00 H

ATOM 2382 CD GLU 153 57.810 52.558 33.982 1.00 0.00 C

ATOM 2383 OE1 GLU 153 58.198 52.517 32.792 1.00 0.00 O

ATOM 2384 OE2 GLU 153 58.391 51.881 34.792 1.00 0.00 O

ATOM 2385 C GLU 153 54.690 55.158 36.220 1.00 0.00 C

ATOM 2386 O GLU 153 53.686 54.881 35.503 1.00 0.00 O

ATOM 2387 N LEU 154 54.742 55.067 37.506 1.00 0.00 N

ATOM 2388 H LEU 154 55.596 55.405 37.926 1.00 0.00 H

ATOM 2389 CA LEU 154 53.572 54.692 38.339 1.00 0.00 C

ATOM 2390 HA LEU 154 53.325 53.631 38.284 1.00 0.00 H

ATOM 2391 CB LEU 154 53.934 54.964 39.770 1.00 0.00 C

ATOM 2392 HB2 LEU 154 54.884 54.519 40.066 1.00 0.00 H

ATOM 2393 HB3 LEU 154 54.083 56.036 39.904 1.00 0.00 H

ATOM 2394 CG LEU 154 53.074 54.441 40.956 1.00 0.00 C

ATOM 2395 HG LEU 154 52.039 54.777 40.894 1.00 0.00 H

ATOM 2396 CD1 LEU 154 52.985 52.877 41.033 1.00 0.00 C

ATOM 2397 HD11 LEU 154 53.988 52.456 41.090 1.00 0.00 H

ATOM 2398 HD12 LEU 154 52.515 52.567 41.967 1.00 0.00 H

ATOM 2399 HD13 LEU 154 52.307 52.436 40.302 1.00 0.00 H

ATOM 2400 CD2 LEU 154 53.601 54.927 42.305 1.00 0.00 C

ATOM 2401 HD21 LEU 154 52.843 54.622 43.027 1.00 0.00 H

ATOM 2402 HD22 LEU 154 54.540 54.549 42.709 1.00 0.00 H

ATOM 2403 HD23 LEU 154 53.654 56.016 42.311 1.00 0.00 H

ATOM 2404 C LEU 154 52.337 55.556 38.050 1.00 0.00 C

ATOM 2405 O LEU 154 51.175 55.016 37.903 1.00 0.00 O

ATOM 2406 N LEU 155 52.538 56.868 37.887 1.00 0.00 N

ATOM 2407 H LEU 155 53.433 57.263 38.138 1.00 0.00 H

ATOM 2408 CA LEU 155 51.480 57.796 37.472 1.00 0.00 C

ATOM 2409 HA LEU 155 50.615 57.552 38.089 1.00 0.00 H

ATOM 2410 CB LEU 155 51.934 59.280 37.676 1.00 0.00 C

ATOM 2411 HB2 LEU 155 52.938 59.301 37.253 1.00 0.00 H

ATOM 2412 HB3 LEU 155 51.324 59.909 37.027 1.00 0.00 H

ATOM 2413 CG LEU 155 52.044 59.707 39.192 1.00 0.00 C

ATOM 2414 HG LEU 155 52.583 58.946 39.755 1.00 0.00 H

ATOM 2415 CD1 LEU 155 52.816 60.988 39.313 1.00 0.00 C

ATOM 2416 HD11 LEU 155 52.769 61.356 40.339 1.00 0.00 H

ATOM 2417 HD12 LEU 155 53.827 60.870 38.924 1.00 0.00 H

ATOM 2418 HD13 LEU 155 52.390 61.837 38.778 1.00 0.00 H

ATOM 2419 CD2 LEU 155 50.659 59.943 39.819 1.00 0.00 C

ATOM 2420 HD21 LEU 155 50.128 58.991 39.853 1.00 0.00 H

ATOM 2421 HD22 LEU 155 50.659 60.361 40.826 1.00 0.00 H

ATOM 2422 HD23 LEU 155 50.177 60.653 39.147 1.00 0.00 H

ATOM 2423 C LEU 155 51.011 57.558 36.039 1.00 0.00 C

ATOM 2424 O LEU 155 49.821 57.235 35.794 1.00 0.00 O

ATOM 2425 N THR 156 51.881 57.745 35.060 1.00 0.00 N

ATOM 2426 H THR 156 52.880 57.733 35.211 1.00 0.00 H

ATOM 2427 CA THR 156 51.486 57.897 33.644 1.00 0.00 C

ATOM 2428 HA THR 156 50.555 58.458 33.553 1.00 0.00 H

ATOM 2429 CB THR 156 52.450 58.750 32.808 1.00 0.00 C

ATOM 2430 HB THR 156 53.279 58.212 32.348 1.00 0.00 H

ATOM 2431 CG2 THR 156 51.652 59.474 31.706 1.00 0.00 C

ATOM 2432 HG21 THR 156 51.268 58.791 30.948 1.00 0.00 H

ATOM 2433 HG22 THR 156 51.014 60.104 32.326 1.00 0.00 H

ATOM 2434 HG23 THR 156 52.244 60.122 31.059 1.00 0.00 H

ATOM 2435 OG1 THR 156 53.063 59.722 33.491 1.00 0.00 O

ATOM 2436 HG1 THR 156 53.566 59.247 34.157 1.00 0.00 H

ATOM 2437 C THR 156 51.436 56.556 32.894 1.00 0.00 C

ATOM 2438 O THR 156 51.024 56.559 31.745 1.00 0.00 O

ATOM 2439 N LYS 157 51.859 55.422 33.473 1.00 0.00 N

ATOM 2440 H LYS 157 52.309 55.576 34.364 1.00 0.00 H

ATOM 2441 CA LYS 157 51.728 54.050 32.891 1.00 0.00 C

ATOM 2442 HA LYS 157 51.082 54.003 32.014 1.00 0.00 H

ATOM 2443 CB LYS 157 53.117 53.462 32.472 1.00 0.00 C

ATOM 2444 HB2 LYS 157 53.580 54.087 31.708 1.00 0.00 H

ATOM 2445 HB3 LYS 157 53.783 53.330 33.324 1.00 0.00 H

ATOM 2446 CG LYS 157 52.977 52.044 31.830 1.00 0.00 C

ATOM 2447 HG2 LYS 157 52.665 51.314 32.577 1.00 0.00 H

ATOM 2448 HG3 LYS 157 52.142 52.094 31.131 1.00 0.00 H

ATOM 2449 CD LYS 157 54.266 51.620 31.069 1.00 0.00 C

ATOM 2450 HD2 LYS 157 54.656 52.393 30.407 1.00 0.00 H

ATOM 2451 HD3 LYS 157 55.082 51.321 31.727 1.00 0.00 H

ATOM 2452 CE LYS 157 54.034 50.451 30.178 1.00 0.00 C

ATOM 2453 HE2 LYS 157 53.361 49.879 30.816 1.00 0.00 H

ATOM 2454 HE3 LYS 157 53.540 50.761 29.257 1.00 0.00 H

ATOM 2455 NZ LYS 157 55.327 49.838 29.872 1.00 0.00 N

ATOM 2456 HZ1 LYS 157 55.230 49.067 29.227 1.00 0.00 H

ATOM 2457 HZ2 LYS 157 55.966 50.538 29.526 1.00 0.00 H

ATOM 2458 HZ3 LYS 157 55.687 49.507 30.756 1.00 0.00 H

ATOM 2459 C LYS 157 51.034 53.068 33.791 1.00 0.00 C

ATOM 2460 O LYS 157 49.980 52.695 33.377 1.00 0.00 O

ATOM 2461 N VAL 158 51.550 52.808 34.949 1.00 0.00 N

ATOM 2462 H VAL 158 52.375 53.307 35.250 1.00 0.00 H

ATOM 2463 CA VAL 158 51.000 51.777 35.875 1.00 0.00 C

ATOM 2464 HA VAL 158 50.893 50.920 35.210 1.00 0.00 H

ATOM 2465 CB VAL 158 51.977 51.555 37.051 1.00 0.00 C

ATOM 2466 HB VAL 158 51.828 52.385 37.741 1.00 0.00 H

ATOM 2467 CG1 VAL 158 51.582 50.254 37.714 1.00 0.00 C

ATOM 2468 HG11 VAL 158 52.284 50.086 38.531 1.00 0.00 H

ATOM 2469 HG12 VAL 158 50.634 50.255 38.252 1.00 0.00 H

ATOM 2470 HG13 VAL 158 51.693 49.461 36.975 1.00 0.00 H

ATOM 2471 CG2 VAL 158 53.422 51.354 36.613 1.00 0.00 C

ATOM 2472 HG21 VAL 158 53.776 52.318 36.249 1.00 0.00 H

ATOM 2473 HG22 VAL 158 54.081 51.006 37.409 1.00 0.00 H

ATOM 2474 HG23 VAL 158 53.569 50.660 35.785 1.00 0.00 H

ATOM 2475 C VAL 158 49.580 51.981 36.434 1.00 0.00 C

ATOM 2476 O VAL 158 48.816 51.032 36.426 1.00 0.00 O

ATOM 2477 N TRP 159 49.173 53.191 36.897 1.00 0.00 N

ATOM 2478 H TRP 159 49.824 53.963 36.923 1.00 0.00 H

ATOM 2479 CA TRP 159 47.785 53.537 37.328 1.00 0.00 C

ATOM 2480 HA TRP 159 47.354 52.581 37.627 1.00 0.00 H

ATOM 2481 CB TRP 159 47.860 54.616 38.449 1.00 0.00 C

ATOM 2482 HB2 TRP 159 48.639 55.343 38.222 1.00 0.00 H

ATOM 2483 HB3 TRP 159 46.894 55.119 38.417 1.00 0.00 H

ATOM 2484 CG TRP 159 48.040 54.231 39.856 1.00 0.00 C

ATOM 2485 CD1 TRP 159 47.561 53.109 40.454 1.00 0.00 C

ATOM 2486 HD1 TRP 159 46.932 52.392 39.948 1.00 0.00 H

ATOM 2487 NE1 TRP 159 48.113 53.010 41.738 1.00 0.00 N

ATOM 2488 HE1 TRP 159 47.897 52.277 42.398 1.00 0.00 H

ATOM 2489 CE2 TRP 159 48.730 54.199 42.078 1.00 0.00 C

ATOM 2490 CZ2 TRP 159 49.311 54.639 43.281 1.00 0.00 C

ATOM 2491 HZ2 TRP 159 49.226 53.944 44.103 1.00 0.00 H

ATOM 2492 CH2 TRP 159 50.034 55.825 43.354 1.00 0.00 C

ATOM 2493 HH2 TRP 159 50.547 56.061 44.275 1.00 0.00 H

ATOM 2494 CZ3 TRP 159 50.200 56.575 42.179 1.00 0.00 C

ATOM 2495 HZ3 TRP 159 50.783 57.484 42.202 1.00 0.00 H

ATOM 2496 CE3 TRP 159 49.576 56.201 40.977 1.00 0.00 C

ATOM 2497 HE3 TRP 159 49.597 56.845 40.110 1.00 0.00 H

ATOM 2498 CD2 TRP 159 48.851 54.959 40.853 1.00 0.00 C

ATOM 2499 C TRP 159 46.867 53.953 36.114 1.00 0.00 C

ATOM 2500 O TRP 159 45.638 53.754 36.216 1.00 0.00 O

ATOM 2501 N VAL 160 47.462 54.315 34.915 1.00 0.00 N

ATOM 2502 H VAL 160 48.472 54.285 34.943 1.00 0.00 H

ATOM 2503 CA VAL 160 46.769 54.457 33.631 1.00 0.00 C

ATOM 2504 HA VAL 160 45.834 54.972 33.855 1.00 0.00 H

ATOM 2505 CB VAL 160 47.486 55.310 32.614 1.00 0.00 C

ATOM 2506 HB VAL 160 48.470 54.885 32.419 1.00 0.00 H

ATOM 2507 CG1 VAL 160 46.846 55.304 31.224 1.00 0.00 C

ATOM 2508 HG11 VAL 160 45.770 55.477 31.260 1.00 0.00 H

ATOM 2509 HG12 VAL 160 47.395 56.084 30.697 1.00 0.00 H

ATOM 2510 HG13 VAL 160 46.987 54.395 30.639 1.00 0.00 H

ATOM 2511 CG2 VAL 160 47.619 56.796 33.058 1.00 0.00 C

ATOM 2512 HG21 VAL 160 46.614 57.217 33.038 1.00 0.00 H

ATOM 2513 HG22 VAL 160 48.028 56.924 34.061 1.00 0.00 H

ATOM 2514 HG23 VAL 160 48.208 57.371 32.344 1.00 0.00 H

ATOM 2515 C VAL 160 46.376 53.112 33.067 1.00 0.00 C

ATOM 2516 O VAL 160 45.161 52.833 32.931 1.00 0.00 O

ATOM 2517 N GLN 161 47.346 52.176 32.781 1.00 0.00 N

ATOM 2518 H GLN 161 48.265 52.490 33.060 1.00 0.00 H

ATOM 2519 CA GLN 161 47.204 50.717 32.459 1.00 0.00 C

ATOM 2520 HA GLN 161 46.665 50.681 31.512 1.00 0.00 H

ATOM 2521 CB GLN 161 48.557 50.145 32.202 1.00 0.00 C

ATOM 2522 HB2 GLN 161 49.207 50.344 33.055 1.00 0.00 H

ATOM 2523 HB3 GLN 161 48.530 49.057 32.137 1.00 0.00 H

ATOM 2524 CG GLN 161 49.168 50.788 30.923 1.00 0.00 C

ATOM 2525 HG2 GLN 161 48.414 50.749 30.137 1.00 0.00 H

ATOM 2526 HG3 GLN 161 49.283 51.872 30.902 1.00 0.00 H

ATOM 2527 CD GLN 161 50.289 49.879 30.434 1.00 0.00 C

ATOM 2528 OE1 GLN 161 51.164 49.406 31.189 1.00 0.00 O

ATOM 2529 NE2 GLN 161 50.271 49.590 29.189 1.00 0.00 N

ATOM 2530 HE21 GLN 161 51.134 49.164 28.883 1.00 0.00 H

ATOM 2531 HE22 GLN 161 49.787 50.173 28.521 1.00 0.00 H

ATOM 2532 C GLN 161 46.428 49.902 33.496 1.00 0.00 C

ATOM 2533 O GLN 161 45.832 48.850 33.229 1.00 0.00 O

ATOM 2534 N GLY 162 46.420 50.445 34.731 1.00 0.00 N

ATOM 2535 H GLY 162 47.064 51.195 34.940 1.00 0.00 H

ATOM 2536 CA GLY 162 45.657 50.074 35.952 1.00 0.00 C

ATOM 2537 HA2 GLY 162 45.657 48.984 35.937 1.00 0.00 H

ATOM 2538 HA3 GLY 162 46.148 50.370 36.879 1.00 0.00 H

ATOM 2539 C GLY 162 44.218 50.657 36.008 1.00 0.00 C

ATOM 2540 O GLY 162 43.524 50.287 36.992 1.00 0.00 O

ATOM 2541 N HIE 163 43.759 51.475 35.055 1.00 0.00 N

ATOM 2542 H HIE 163 44.405 51.757 34.331 1.00 0.00 H

ATOM 2543 CA HIE 163 42.415 52.026 34.911 1.00 0.00 C

ATOM 2544 HA HIE 163 42.394 52.902 34.262 1.00 0.00 H

ATOM 2545 CB HIE 163 41.539 50.876 34.409 1.00 0.00 C

ATOM 2546 HB2 HIE 163 41.380 50.151 35.207 1.00 0.00 H

ATOM 2547 HB3 HIE 163 40.559 51.297 34.187 1.00 0.00 H

ATOM 2548 CG HIE 163 42.137 50.041 33.259 1.00 0.00 C

ATOM 2549 ND1 HIE 163 41.930 48.688 33.065 1.00 0.00 N

ATOM 2550 CE1 HIE 163 42.606 48.312 31.972 1.00 0.00 C

ATOM 2551 HE1 HIE 163 42.637 47.273 31.680 1.00 0.00 H

ATOM 2552 NE2 HIE 163 43.232 49.398 31.413 1.00 0.00 N

ATOM 2553 HE2 HIE 163 43.791 49.372 30.572 1.00 0.00 H

ATOM 2554 CD2 HIE 163 43.007 50.472 32.284 1.00 0.00 C

ATOM 2555 HD2 HIE 163 43.434 51.463 32.224 1.00 0.00 H

ATOM 2556 C HIE 163 41.862 52.757 36.161 1.00 0.00 C

ATOM 2557 O HIE 163 40.758 53.298 36.136 1.00 0.00 O

ATOM 2558 N TYR 164 42.711 52.973 37.131 1.00 0.00 N

ATOM 2559 H TYR 164 43.567 52.444 37.222 1.00 0.00 H

ATOM 2560 CA TYR 164 42.468 53.993 38.182 1.00 0.00 C

ATOM 2561 HA TYR 164 41.411 53.848 38.404 1.00 0.00 H

ATOM 2562 CB TYR 164 43.123 53.536 39.530 1.00 0.00 C

ATOM 2563 HB2 TYR 164 44.191 53.397 39.363 1.00 0.00 H

ATOM 2564 HB3 TYR 164 42.947 54.349 40.235 1.00 0.00 H

ATOM 2565 CG TYR 164 42.575 52.293 40.166 1.00 0.00 C

ATOM 2566 CD1 TYR 164 41.265 52.311 40.662 1.00 0.00 C

ATOM 2567 HD1 TYR 164 40.619 53.174 40.591 1.00 0.00 H

ATOM 2568 CE1 TYR 164 40.662 51.143 41.103 1.00 0.00 C

ATOM 2569 HE1 TYR 164 39.612 51.239 41.338 1.00 0.00 H

ATOM 2570 CZ TYR 164 41.494 49.940 41.220 1.00 0.00 C

ATOM 2571 OH TYR 164 40.964 48.743 41.515 1.00 0.00 O

ATOM 2572 HH TYR 164 40.022 48.765 41.698 1.00 0.00 H

ATOM 2573 CE2 TYR 164 42.830 49.977 40.789 1.00 0.00 C

ATOM 2574 HE2 TYR 164 43.368 49.040 40.770 1.00 0.00 H

ATOM 2575 CD2 TYR 164 43.418 51.178 40.382 1.00 0.00 C

ATOM 2576 HD2 TYR 164 44.440 51.310 40.059 1.00 0.00 H

ATOM 2577 C TYR 164 42.711 55.432 37.863 1.00 0.00 C

ATOM 2578 O TYR 164 42.121 56.368 38.443 1.00 0.00 O

ATOM 2579 N LEU 165 43.524 55.676 36.821 1.00 0.00 N

ATOM 2580 H LEU 165 43.916 54.872 36.353 1.00 0.00 H

ATOM 2581 CA LEU 165 43.640 57.021 36.220 1.00 0.00 C

ATOM 2582 HA LEU 165 42.922 57.712 36.662 1.00 0.00 H

ATOM 2583 CB LEU 165 45.044 57.631 36.548 1.00 0.00 C

ATOM 2584 HB2 LEU 165 45.844 57.018 36.134 1.00 0.00 H

ATOM 2585 HB3 LEU 165 45.204 58.582 36.039 1.00 0.00 H

ATOM 2586 CG LEU 165 45.441 57.957 38.001 1.00 0.00 C

ATOM 2587 HG LEU 165 45.348 57.068 38.624 1.00 0.00 H

ATOM 2588 CD1 LEU 165 46.917 58.557 37.950 1.00 0.00 C

ATOM 2589 HD11 LEU 165 47.384 58.332 38.910 1.00 0.00 H

ATOM 2590 HD12 LEU 165 47.432 57.989 37.175 1.00 0.00 H

ATOM 2591 HD13 LEU 165 47.024 59.611 37.696 1.00 0.00 H

ATOM 2592 CD2 LEU 165 44.527 58.999 38.695 1.00 0.00 C

ATOM 2593 HD21 LEU 165 44.288 59.767 37.960 1.00 0.00 H

ATOM 2594 HD22 LEU 165 43.532 58.609 38.909 1.00 0.00 H

ATOM 2595 HD23 LEU 165 44.978 59.335 39.629 1.00 0.00 H

ATOM 2596 C LEU 165 43.508 57.032 34.721 1.00 0.00 C

ATOM 2597 O LEU 165 43.804 56.016 34.074 1.00 0.00 O

ATOM 2598 N GLU 166 43.185 58.185 34.189 1.00 0.00 N

ATOM 2599 H GLU 166 42.856 58.831 34.893 1.00 0.00 H

ATOM 2600 CA GLU 166 43.537 58.650 32.820 1.00 0.00 C

ATOM 2601 HA GLU 166 44.298 57.981 32.419 1.00 0.00 H

ATOM 2602 CB GLU 166 42.347 58.489 31.878 1.00 0.00 C

ATOM 2603 HB2 GLU 166 42.669 58.606 30.843 1.00 0.00 H

ATOM 2604 HB3 GLU 166 41.940 57.479 31.930 1.00 0.00 H

ATOM 2605 CG GLU 166 41.127 59.442 32.174 1.00 0.00 C

ATOM 2606 HG2 GLU 166 40.790 59.427 33.211 1.00 0.00 H

ATOM 2607 HG3 GLU 166 41.373 60.485 31.979 1.00 0.00 H

ATOM 2608 CD GLU 166 39.926 59.147 31.266 1.00 0.00 C

ATOM 2609 OE1 GLU 166 38.813 59.655 31.617 1.00 0.00 O

ATOM 2610 OE2 GLU 166 40.083 58.596 30.150 1.00 0.00 O

ATOM 2611 C GLU 166 44.200 60.065 32.848 1.00 0.00 C

ATOM 2612 O GLU 166 44.033 60.778 33.834 1.00 0.00 O

ATOM 2613 N TYR 167 44.796 60.414 31.660 1.00 0.00 N

ATOM 2614 H TYR 167 44.886 59.669 30.984 1.00 0.00 H

ATOM 2615 CA TYR 167 45.628 61.610 31.540 1.00 0.00 C

ATOM 2616 HA TYR 167 45.668 62.172 32.474 1.00 0.00 H

ATOM 2617 CB TYR 167 47.121 61.052 31.372 1.00 0.00 C

ATOM 2618 HB2 TYR 167 47.319 60.590 32.339 1.00 0.00 H

ATOM 2619 HB3 TYR 167 46.981 60.343 30.557 1.00 0.00 H

ATOM 2620 CG TYR 167 48.218 62.181 31.081 1.00 0.00 C

ATOM 2621 CD1 TYR 167 48.921 62.629 32.229 1.00 0.00 C

ATOM 2622 HD1 TYR 167 48.638 62.404 33.247 1.00 0.00 H

ATOM 2623 CE1 TYR 167 50.088 63.454 32.021 1.00 0.00 C

ATOM 2624 HE1 TYR 167 50.694 63.800 32.845 1.00 0.00 H

ATOM 2625 CZ TYR 167 50.380 63.879 30.711 1.00 0.00 C

ATOM 2626 OH TYR 167 51.598 64.517 30.480 1.00 0.00 O

ATOM 2627 HH TYR 167 52.073 64.591 31.311 1.00 0.00 H

ATOM 2628 CE2 TYR 167 49.618 63.463 29.620 1.00 0.00 C

ATOM 2629 HE2 TYR 167 49.794 63.894 28.646 1.00 0.00 H

ATOM 2630 CD2 TYR 167 48.650 62.495 29.799 1.00 0.00 C

ATOM 2631 HD2 TYR 167 48.027 62.088 29.017 1.00 0.00 H

ATOM 2632 C TYR 167 45.263 62.704 30.440 1.00 0.00 C

ATOM 2633 O TYR 167 44.598 62.389 29.460 1.00 0.00 O

ATOM 2634 N ARG 168 45.551 63.980 30.551 1.00 0.00 N

ATOM 2635 H ARG 168 46.163 64.138 31.339 1.00 0.00 H

ATOM 2636 CA ARG 168 45.308 65.066 29.578 1.00 0.00 C

ATOM 2637 HA ARG 168 45.160 64.636 28.587 1.00 0.00 H

ATOM 2638 CB ARG 168 44.002 65.724 29.989 1.00 0.00 C

ATOM 2639 HB2 ARG 168 43.450 65.233 30.791 1.00 0.00 H

ATOM 2640 HB3 ARG 168 44.290 66.712 30.347 1.00 0.00 H

ATOM 2641 CG ARG 168 42.995 65.792 28.866 1.00 0.00 C

ATOM 2642 HG2 ARG 168 43.487 66.032 27.923 1.00 0.00 H

ATOM 2643 HG3 ARG 168 42.518 64.813 28.818 1.00 0.00 H

ATOM 2644 CD ARG 168 41.939 66.904 29.117 1.00 0.00 C

ATOM 2645 HD2 ARG 168 41.555 66.901 30.137 1.00 0.00 H

ATOM 2646 HD3 ARG 168 42.465 67.842 28.938 1.00 0.00 H

ATOM 2647 NE ARG 168 40.780 66.828 28.177 1.00 0.00 N

ATOM 2648 HE ARG 168 40.417 65.924 27.910 1.00 0.00 H

ATOM 2649 CZ ARG 168 40.065 67.801 27.708 1.00 0.00 C

ATOM 2650 NH1 ARG 168 40.348 69.110 27.904 1.00 0.00 N

ATOM 2651 HH11 ARG 168 41.196 69.403 28.367 1.00 0.00 H

ATOM 2652 HH12 ARG 168 39.871 69.833 27.384 1.00 0.00 H

ATOM 2653 NH2 ARG 168 39.092 67.560 26.847 1.00 0.00 N

ATOM 2654 HH21 ARG 168 39.035 66.643 26.427 1.00 0.00 H

ATOM 2655 HH22 ARG 168 38.542 68.332 26.498 1.00 0.00 H

ATOM 2656 C ARG 168 46.509 66.018 29.571 1.00 0.00 C

ATOM 2657 O ARG 168 47.177 66.189 30.562 1.00 0.00 O

ATOM 2658 N GLU 169 46.863 66.508 28.440 1.00 0.00 N

ATOM 2659 H GLU 169 46.439 66.013 27.668 1.00 0.00 H

ATOM 2660 CA GLU 169 47.837 67.642 28.191 1.00 0.00 C

ATOM 2661 HA GLU 169 48.082 68.122 29.139 1.00 0.00 H

ATOM 2662 CB GLU 169 49.182 67.001 27.738 1.00 0.00 C

ATOM 2663 HB2 GLU 169 49.856 67.846 27.600 1.00 0.00 H

ATOM 2664 HB3 GLU 169 49.634 66.419 28.541 1.00 0.00 H

ATOM 2665 CG GLU 169 49.232 66.170 26.449 1.00 0.00 C

ATOM 2666 HG2 GLU 169 48.599 65.291 26.575 1.00 0.00 H

ATOM 2667 HG3 GLU 169 48.940 66.866 25.663 1.00 0.00 H

ATOM 2668 CD GLU 169 50.581 65.636 26.185 1.00 0.00 C

ATOM 2669 OE1 GLU 169 51.534 66.357 25.855 1.00 0.00 O

ATOM 2670 OE2 GLU 169 50.780 64.412 26.257 1.00 0.00 O

ATOM 2671 C GLU 169 47.246 68.679 27.327 1.00 0.00 C

ATOM 2672 O GLU 169 47.785 69.722 27.051 1.00 0.00 O

ATOM 2673 N VAL 170 46.018 68.474 26.801 1.00 0.00 N

ATOM 2674 H VAL 170 45.439 67.680 27.037 1.00 0.00 H

ATOM 2675 CA VAL 170 45.545 69.359 25.680 1.00 0.00 C

ATOM 2676 HA VAL 170 46.468 69.506 25.118 1.00 0.00 H

ATOM 2677 CB VAL 170 44.496 68.669 24.739 1.00 0.00 C

ATOM 2678 HB VAL 170 44.812 67.694 24.368 1.00 0.00 H

ATOM 2679 CG1 VAL 170 43.188 68.288 25.454 1.00 0.00 C

ATOM 2680 HG11 VAL 170 42.686 67.672 24.708 1.00 0.00 H

ATOM 2681 HG12 VAL 170 43.440 67.648 26.300 1.00 0.00 H

ATOM 2682 HG13 VAL 170 42.627 69.118 25.883 1.00 0.00 H

ATOM 2683 CG2 VAL 170 44.147 69.514 23.477 1.00 0.00 C

ATOM 2684 HG21 VAL 170 45.021 69.578 22.829 1.00 0.00 H

ATOM 2685 HG22 VAL 170 43.454 69.012 22.802 1.00 0.00 H

ATOM 2686 HG23 VAL 170 43.835 70.511 23.789 1.00 0.00 H

ATOM 2687 C VAL 170 45.167 70.714 26.260 1.00 0.00 C

ATOM 2688 O VAL 170 44.609 70.849 27.302 1.00 0.00 O

ATOM 2689 N PRO 171 45.504 71.793 25.572 1.00 0.00 N

ATOM 2690 CD PRO 171 46.600 71.890 24.604 1.00 0.00 C

ATOM 2691 HD2 PRO 171 46.393 71.358 23.675 1.00 0.00 H

ATOM 2692 HD3 PRO 171 47.504 71.561 25.117 1.00 0.00 H

ATOM 2693 CG PRO 171 46.814 73.385 24.316 1.00 0.00 C

ATOM 2694 HG2 PRO 171 46.342 73.710 23.389 1.00 0.00 H

ATOM 2695 HG3 PRO 171 47.881 73.608 24.310 1.00 0.00 H

ATOM 2696 CB PRO 171 46.201 74.104 25.504 1.00 0.00 C

ATOM 2697 HB2 PRO 171 45.789 75.080 25.248 1.00 0.00 H

ATOM 2698 HB3 PRO 171 46.819 74.116 26.402 1.00 0.00 H

ATOM 2699 CA PRO 171 45.059 73.132 25.836 1.00 0.00 C

ATOM 2700 HA PRO 171 44.862 73.360 26.884 1.00 0.00 H

ATOM 2701 C PRO 171 43.768 73.464 25.116 1.00 0.00 C

ATOM 2702 O PRO 171 43.470 72.925 24.059 1.00 0.00 O

ATOM 2703 N HIE 172 42.931 74.284 25.718 1.00 0.00 N

ATOM 2704 H HIE 172 43.052 74.541 26.688 1.00 0.00 H

ATOM 2705 CA HIE 172 41.673 74.796 25.104 1.00 0.00 C

ATOM 2706 HA HIE 172 41.872 74.860 24.034 1.00 0.00 H

ATOM 2707 CB HIE 172 40.503 73.894 25.433 1.00 0.00 C

ATOM 2708 HB2 HIE 172 39.691 74.316 24.840 1.00 0.00 H

ATOM 2709 HB3 HIE 172 40.638 72.849 25.154 1.00 0.00 H

ATOM 2710 CG HIE 172 40.096 74.053 26.878 1.00 0.00 C

ATOM 2711 ND1 HIE 172 39.018 74.919 27.348 1.00 0.00 N

ATOM 2712 CE1 HIE 172 38.957 74.580 28.669 1.00 0.00 C

ATOM 2713 HE1 HIE 172 38.410 75.069 29.461 1.00 0.00 H

ATOM 2714 NE2 HIE 172 39.901 73.604 29.020 1.00 0.00 N

ATOM 2715 HE2 HIE 172 39.964 73.174 29.932 1.00 0.00 H

ATOM 2716 CD2 HIE 172 40.585 73.255 27.913 1.00 0.00 C

ATOM 2717 HD2 HIE 172 41.388 72.541 27.808 1.00 0.00 H

ATOM 2718 C HIE 172 41.449 76.306 25.504 1.00 0.00 C

ATOM 2719 O HIE 172 40.749 76.964 24.743 1.00 0.00 O

ATOM 2720 N SER 173 41.932 76.749 26.715 1.00 0.00 N

ATOM 2721 H SER 173 42.524 76.087 27.197 1.00 0.00 H

ATOM 2722 CA SER 173 41.668 78.137 27.327 1.00 0.00 C

ATOM 2723 HA SER 173 41.286 78.858 26.604 1.00 0.00 H

ATOM 2724 CB SER 173 40.720 78.041 28.451 1.00 0.00 C

ATOM 2725 HB2 SER 173 41.065 77.340 29.211 1.00 0.00 H

ATOM 2726 HB3 SER 173 40.625 79.006 28.949 1.00 0.00 H

ATOM 2727 OG SER 173 39.463 77.602 28.061 1.00 0.00 O

ATOM 2728 HG SER 173 39.403 76.648 28.149 1.00 0.00 H

ATOM 2729 C SER 173 42.958 78.772 27.943 1.00 0.00 C

ATOM 2730 O SER 173 43.230 79.947 27.557 1.00 0.00 O

ATOM 2731 N SER 174 43.701 78.007 28.800 1.00 0.00 N

ATOM 2732 H SER 174 43.329 77.111 29.082 1.00 0.00 H

ATOM 2733 CA SER 174 45.003 78.476 29.291 1.00 0.00 C

ATOM 2734 HA SER 174 45.044 79.565 29.249 1.00 0.00 H

ATOM 2735 CB SER 174 45.209 77.957 30.780 1.00 0.00 C

ATOM 2736 HB2 SER 174 45.055 76.879 30.819 1.00 0.00 H

ATOM 2737 HB3 SER 174 46.211 78.221 31.121 1.00 0.00 H

ATOM 2738 OG SER 174 44.327 78.566 31.623 1.00 0.00 O

ATOM 2739 HG SER 174 44.458 78.209 32.505 1.00 0.00 H

ATOM 2740 C SER 174 46.175 77.997 28.408 1.00 0.00 C

ATOM 2741 O SER 174 46.000 77.200 27.498 1.00 0.00 O

ATOM 2742 N PRO 175 47.360 78.516 28.689 1.00 0.00 N

ATOM 2743 CD PRO 175 47.718 79.717 29.449 1.00 0.00 C

ATOM 2744 HD2 PRO 175 47.920 79.485 30.495 1.00 0.00 H

ATOM 2745 HD3 PRO 175 47.053 80.576 29.359 1.00 0.00 H

ATOM 2746 CG PRO 175 49.085 80.113 28.904 1.00 0.00 C

ATOM 2747 HG2 PRO 175 49.711 80.714 29.563 1.00 0.00 H

ATOM 2748 HG3 PRO 175 48.912 80.624 27.957 1.00 0.00 H

ATOM 2749 CB PRO 175 49.695 78.726 28.675 1.00 0.00 C

ATOM 2750 HB2 PRO 175 50.204 78.363 29.568 1.00 0.00 H

ATOM 2751 HB3 PRO 175 50.344 78.747 27.800 1.00 0.00 H

ATOM 2752 CA PRO 175 48.556 77.841 28.299 1.00 0.00 C

ATOM 2753 HA PRO 175 48.581 77.747 27.213 1.00 0.00 H

ATOM 2754 C PRO 175 48.632 76.416 28.860 1.00 0.00 C

ATOM 2755 O PRO 175 47.850 76.176 29.798 1.00 0.00 O

ATOM 2756 N PRO 176 49.450 75.538 28.382 1.00 0.00 N

ATOM 2757 CD PRO 176 50.230 75.693 27.146 1.00 0.00 C

ATOM 2758 HD2 PRO 176 51.135 76.272 27.329 1.00 0.00 H

ATOM 2759 HD3 PRO 176 49.762 76.371 26.432 1.00 0.00 H

ATOM 2760 CG PRO 176 50.522 74.268 26.650 1.00 0.00 C

ATOM 2761 HG2 PRO 176 51.510 74.088 26.226 1.00 0.00 H

ATOM 2762 HG3 PRO 176 49.740 73.915 25.977 1.00 0.00 H

ATOM 2763 CB PRO 176 50.490 73.450 27.973 1.00 0.00 C

ATOM 2764 HB2 PRO 176 51.392 73.620 28.559 1.00 0.00 H

ATOM 2765 HB3 PRO 176 50.306 72.388 27.808 1.00 0.00 H

ATOM 2766 CA PRO 176 49.353 74.111 28.760 1.00 0.00 C

ATOM 2767 HA PRO 176 48.335 73.893 28.437 1.00 0.00 H

ATOM 2768 C PRO 176 49.483 73.867 30.194 1.00 0.00 C

ATOM 2769 O PRO 176 50.301 74.384 30.866 1.00 0.00 O

ATOM 2770 N TYR 177 48.684 72.973 30.706 1.00 0.00 N

ATOM 2771 H TYR 177 47.894 72.701 30.139 1.00 0.00 H

ATOM 2772 CA TYR 177 48.795 72.188 32.001 1.00 0.00 C

ATOM 2773 HA TYR 177 49.817 72.280 32.368 1.00 0.00 H

ATOM 2774 CB TYR 177 47.745 72.760 32.961 1.00 0.00 C

ATOM 2775 HB2 TYR 177 46.749 72.837 32.524 1.00 0.00 H

ATOM 2776 HB3 TYR 177 47.566 72.174 33.863 1.00 0.00 H

ATOM 2777 CG TYR 177 48.035 74.157 33.483 1.00 0.00 C

ATOM 2778 CD1 TYR 177 49.221 74.585 34.030 1.00 0.00 C

ATOM 2779 HD1 TYR 177 50.101 73.959 34.011 1.00 0.00 H

ATOM 2780 CE1 TYR 177 49.312 75.818 34.629 1.00 0.00 C

ATOM 2781 HE1 TYR 177 50.271 76.022 35.081 1.00 0.00 H

ATOM 2782 CZ TYR 177 48.230 76.706 34.665 1.00 0.00 C

ATOM 2783 OH TYR 177 48.440 77.934 35.217 1.00 0.00 O

ATOM 2784 HH TYR 177 49.314 77.958 35.612 1.00 0.00 H

ATOM 2785 CE2 TYR 177 47.021 76.301 33.970 1.00 0.00 C

ATOM 2786 HE2 TYR 177 46.226 77.021 33.852 1.00 0.00 H

ATOM 2787 CD2 TYR 177 46.954 75.040 33.353 1.00 0.00 C

ATOM 2788 HD2 TYR 177 46.020 74.898 32.829 1.00 0.00 H

ATOM 2789 C TYR 177 48.599 70.696 31.728 1.00 0.00 C

ATOM 2790 O TYR 177 48.022 70.352 30.670 1.00 0.00 O

ATOM 2791 N TYR 178 49.065 69.831 32.599 1.00 0.00 N

ATOM 2792 H TYR 178 49.537 70.232 33.397 1.00 0.00 H

ATOM 2793 CA TYR 178 49.221 68.344 32.474 1.00 0.00 C

ATOM 2794 HA TYR 178 48.662 68.150 31.558 1.00 0.00 H

ATOM 2795 CB TYR 178 50.709 68.013 32.165 1.00 0.00 C

ATOM 2796 HB2 TYR 178 51.148 67.936 33.160 1.00 0.00 H

ATOM 2797 HB3 TYR 178 50.758 67.038 31.680 1.00 0.00 H

ATOM 2798 CG TYR 178 51.426 68.956 31.287 1.00 0.00 C

ATOM 2799 CD1 TYR 178 51.484 68.881 29.849 1.00 0.00 C

ATOM 2800 HD1 TYR 178 50.945 68.066 29.389 1.00 0.00 H

ATOM 2801 CE1 TYR 178 52.286 69.801 29.115 1.00 0.00 C

ATOM 2802 HE1 TYR 178 52.277 69.825 28.036 1.00 0.00 H

ATOM 2803 CZ TYR 178 53.062 70.802 29.761 1.00 0.00 C

ATOM 2804 OH TYR 178 53.810 71.706 29.104 1.00 0.00 O

ATOM 2805 HH TYR 178 53.726 71.768 28.150 1.00 0.00 H

ATOM 2806 CE2 TYR 178 52.887 70.977 31.125 1.00 0.00 C

ATOM 2807 HE2 TYR 178 53.226 71.866 31.636 1.00 0.00 H

ATOM 2808 CD2 TYR 178 52.085 70.028 31.902 1.00 0.00 C

ATOM 2809 HD2 TYR 178 51.860 70.200 32.945 1.00 0.00 H

ATOM 2810 C TYR 178 48.535 67.646 33.618 1.00 0.00 C

ATOM 2811 O TYR 178 48.849 67.874 34.773 1.00 0.00 O

ATOM 2812 N GLU 179 47.552 66.835 33.278 1.00 0.00 N

ATOM 2813 H GLU 179 47.432 66.611 32.300 1.00 0.00 H

ATOM 2814 CA GLU 179 46.436 66.510 34.192 1.00 0.00 C

ATOM 2815 HA GLU 179 46.728 66.907 35.164 1.00 0.00 H

ATOM 2816 CB GLU 179 45.145 67.275 33.762 1.00 0.00 C

ATOM 2817 HB2 GLU 179 44.971 67.025 32.716 1.00 0.00 H

ATOM 2818 HB3 GLU 179 44.310 66.943 34.379 1.00 0.00 H

ATOM 2819 CG GLU 179 45.328 68.830 33.865 1.00 0.00 C

ATOM 2820 HG2 GLU 179 45.630 69.160 34.859 1.00 0.00 H

ATOM 2821 HG3 GLU 179 46.008 69.164 33.082 1.00 0.00 H

ATOM 2822 CD GLU 179 44.049 69.554 33.563 1.00 0.00 C

ATOM 2823 OE1 GLU 179 43.188 69.688 34.460 1.00 0.00 O

ATOM 2824 OE2 GLU 179 43.790 69.767 32.411 1.00 0.00 O

ATOM 2825 C GLU 179 46.177 64.999 34.327 1.00 0.00 C

ATOM 2826 O GLU 179 46.217 64.250 33.312 1.00 0.00 O

ATOM 2827 N PHE 180 45.836 64.516 35.570 1.00 0.00 N

ATOM 2828 H PHE 180 45.895 65.177 36.331 1.00 0.00 H

ATOM 2829 CA PHE 180 45.355 63.263 36.050 1.00 0.00 C

ATOM 2830 HA PHE 180 45.358 62.585 35.196 1.00 0.00 H

ATOM 2831 CB PHE 180 46.310 62.621 37.059 1.00 0.00 C

ATOM 2832 HB2 PHE 180 46.286 63.201 37.982 1.00 0.00 H

ATOM 2833 HB3 PHE 180 45.905 61.617 37.184 1.00 0.00 H

ATOM 2834 CG PHE 180 47.798 62.506 36.715 1.00 0.00 C

ATOM 2835 CD1 PHE 180 48.629 63.523 37.150 1.00 0.00 C

ATOM 2836 HD1 PHE 180 48.230 64.368 37.691 1.00 0.00 H

ATOM 2837 CE1 PHE 180 50.011 63.447 36.914 1.00 0.00 C

ATOM 2838 HE1 PHE 180 50.765 64.187 37.137 1.00 0.00 H

ATOM 2839 CZ PHE 180 50.508 62.359 36.160 1.00 0.00 C

ATOM 2840 HZ PHE 180 51.577 62.396 36.012 1.00 0.00 H

ATOM 2841 CE2 PHE 180 49.662 61.305 35.670 1.00 0.00 C

ATOM 2842 HE2 PHE 180 50.057 60.458 35.128 1.00 0.00 H

ATOM 2843 CD2 PHE 180 48.293 61.463 35.922 1.00 0.00 C

ATOM 2844 HD2 PHE 180 47.646 60.612 35.769 1.00 0.00 H

ATOM 2845 C PHE 180 43.902 63.450 36.586 1.00 0.00 C

ATOM 2846 O PHE 180 43.619 64.314 37.398 1.00 0.00 O

ATOM 2847 N LEU 181 43.105 62.462 36.266 1.00 0.00 N

ATOM 2848 H LEU 181 43.424 61.828 35.547 1.00 0.00 H

ATOM 2849 CA LEU 181 41.663 62.346 36.710 1.00 0.00 C

ATOM 2850 HA LEU 181 41.433 62.903 37.618 1.00 0.00 H

ATOM 2851 CB LEU 181 40.700 62.721 35.635 1.00 0.00 C

ATOM 2852 HB2 LEU 181 39.730 62.276 35.859 1.00 0.00 H

ATOM 2853 HB3 LEU 181 40.596 63.788 35.832 1.00 0.00 H

ATOM 2854 CG LEU 181 41.165 62.513 34.180 1.00 0.00 C

ATOM 2855 HG LEU 181 41.642 61.541 34.059 1.00 0.00 H

ATOM 2856 CD1 LEU 181 39.929 62.342 33.254 1.00 0.00 C

ATOM 2857 HD11 LEU 181 39.593 61.318 33.418 1.00 0.00 H

ATOM 2858 HD12 LEU 181 39.111 62.958 33.628 1.00 0.00 H

ATOM 2859 HD13 LEU 181 40.067 62.347 32.173 1.00 0.00 H

ATOM 2860 CD2 LEU 181 41.986 63.676 33.496 1.00 0.00 C

ATOM 2861 HD21 LEU 181 41.908 63.688 32.409 1.00 0.00 H

ATOM 2862 HD22 LEU 181 41.535 64.558 33.951 1.00 0.00 H

ATOM 2863 HD23 LEU 181 42.998 63.846 33.863 1.00 0.00 H

ATOM 2864 C LEU 181 41.390 60.843 37.023 1.00 0.00 C

ATOM 2865 O LEU 181 42.181 59.951 36.566 1.00 0.00 O

ATOM 2866 N TRP 182 40.288 60.500 37.673 1.00 0.00 N

ATOM 2867 H TRP 182 39.688 61.249 37.988 1.00 0.00 H

ATOM 2868 CA TRP 182 39.822 59.230 38.047 1.00 0.00 C

ATOM 2869 HA TRP 182 40.541 58.689 38.662 1.00 0.00 H

ATOM 2870 CB TRP 182 38.442 59.338 38.771 1.00 0.00 C

ATOM 2871 HB2 TRP 182 37.804 60.002 38.187 1.00 0.00 H

ATOM 2872 HB3 TRP 182 37.896 58.419 38.561 1.00 0.00 H

ATOM 2873 CG TRP 182 38.412 59.759 40.205 1.00 0.00 C

ATOM 2874 CD1 TRP 182 39.416 60.476 40.854 1.00 0.00 C

ATOM 2875 HD1 TRP 182 40.341 60.792 40.395 1.00 0.00 H

ATOM 2876 NE1 TRP 182 39.013 60.777 42.146 1.00 0.00 N

ATOM 2877 HE1 TRP 182 39.578 61.340 42.766 1.00 0.00 H

ATOM 2878 CE2 TRP 182 37.858 60.160 42.478 1.00 0.00 C

ATOM 2879 CZ2 TRP 182 37.159 60.049 43.649 1.00 0.00 C

ATOM 2880 HZ2 TRP 182 37.609 60.483 44.530 1.00 0.00 H

ATOM 2881 CH2 TRP 182 35.909 59.468 43.662 1.00 0.00 C

ATOM 2882 HH2 TRP 182 35.376 59.361 44.595 1.00 0.00 H

ATOM 2883 CZ3 TRP 182 35.399 58.937 42.453 1.00 0.00 C

ATOM 2884 HZ3 TRP 182 34.422 58.493 42.332 1.00 0.00 H

ATOM 2885 CE3 TRP 182 36.069 59.046 41.246 1.00 0.00 C

ATOM 2886 HE3 TRP 182 35.641 58.583 40.370 1.00 0.00 H

ATOM 2887 CD2 TRP 182 37.321 59.619 41.246 1.00 0.00 C

ATOM 2888 C TRP 182 39.630 58.382 36.732 1.00 0.00 C

ATOM 2889 O TRP 182 38.857 58.830 35.874 1.00 0.00 O

ATOM 2890 N GLY 183 40.271 57.223 36.563 1.00 0.00 N

ATOM 2891 H GLY 183 40.811 56.916 37.359 1.00 0.00 H

ATOM 2892 CA GLY 183 40.006 56.439 35.389 1.00 0.00 C

ATOM 2893 HA2 GLY 183 39.966 56.983 34.446 1.00 0.00 H

ATOM 2894 HA3 GLY 183 40.870 55.776 35.335 1.00 0.00 H

ATOM 2895 C GLY 183 38.680 55.623 35.551 1.00 0.00 C

ATOM 2896 O GLY 183 38.104 55.564 36.621 1.00 0.00 O

ATOM 2897 N PRO 184 38.188 55.041 34.400 1.00 0.00 N

ATOM 2898 CD PRO 184 38.729 55.313 33.092 1.00 0.00 C

ATOM 2899 HD2 PRO 184 39.577 54.674 32.844 1.00 0.00 H

ATOM 2900 HD3 PRO 184 39.043 56.345 32.931 1.00 0.00 H

ATOM 2901 CG PRO 184 37.610 54.957 32.086 1.00 0.00 C

ATOM 2902 HG2 PRO 184 38.087 54.599 31.173 1.00 0.00 H

ATOM 2903 HG3 PRO 184 36.887 55.753 31.907 1.00 0.00 H

ATOM 2904 CB PRO 184 36.846 53.858 32.900 1.00 0.00 C

ATOM 2905 HB2 PRO 184 37.440 52.950 32.792 1.00 0.00 H

ATOM 2906 HB3 PRO 184 35.829 53.596 32.608 1.00 0.00 H

ATOM 2907 CA PRO 184 36.880 54.454 34.281 1.00 0.00 C

ATOM 2908 HA PRO 184 36.198 55.303 34.280 1.00 0.00 H

ATOM 2909 C PRO 184 36.604 53.328 35.303 1.00 0.00 C

ATOM 2910 O PRO 184 35.452 53.228 35.662 1.00 0.00 O

ATOM 2911 N ARG 185 37.627 52.702 35.849 1.00 0.00 N

ATOM 2912 H ARG 185 38.498 53.043 35.470 1.00 0.00 H

ATOM 2913 CA ARG 185 37.612 51.738 36.961 1.00 0.00 C

ATOM 2914 HA ARG 185 36.960 50.895 36.728 1.00 0.00 H

ATOM 2915 CB ARG 185 38.917 51.066 36.957 1.00 0.00 C

ATOM 2916 HB2 ARG 185 39.163 51.045 35.895 1.00 0.00 H

ATOM 2917 HB3 ARG 185 39.591 51.741 37.485 1.00 0.00 H

ATOM 2918 CG ARG 185 38.911 49.701 37.589 1.00 0.00 C

ATOM 2919 HG2 ARG 185 38.760 49.883 38.653 1.00 0.00 H

ATOM 2920 HG3 ARG 185 38.156 49.100 37.082 1.00 0.00 H

ATOM 2921 CD ARG 185 40.236 49.037 37.449 1.00 0.00 C

ATOM 2922 HD2 ARG 185 40.364 48.755 36.404 1.00 0.00 H

ATOM 2923 HD3 ARG 185 40.906 49.846 37.741 1.00 0.00 H

ATOM 2924 NE ARG 185 40.363 47.898 38.388 1.00 0.00 N

ATOM 2925 HE ARG 185 39.529 47.663 38.906 1.00 0.00 H

ATOM 2926 CZ ARG 185 41.572 47.329 38.656 1.00 0.00 C

ATOM 2927 NH1 ARG 185 42.596 47.677 37.986 1.00 0.00 N

ATOM 2928 HH11 ARG 185 42.414 48.531 37.479 1.00 0.00 H

ATOM 2929 HH12 ARG 185 43.531 47.332 38.149 1.00 0.00 H

ATOM 2930 NH2 ARG 185 41.699 46.474 39.577 1.00 0.00 N

ATOM 2931 HH21 ARG 185 40.898 46.012 39.984 1.00 0.00 H

ATOM 2932 HH22 ARG 185 42.642 46.116 39.633 1.00 0.00 H

ATOM 2933 C ARG 185 37.367 52.303 38.297 1.00 0.00 C

ATOM 2934 O ARG 185 36.686 51.681 39.134 1.00 0.00 O

ATOM 2935 N ALA 186 38.004 53.432 38.622 1.00 0.00 N

ATOM 2936 H ALA 186 38.257 54.014 37.837 1.00 0.00 H

ATOM 2937 CA ALA 186 37.832 54.076 39.910 1.00 0.00 C

ATOM 2938 HA ALA 186 38.150 53.412 40.713 1.00 0.00 H

ATOM 2939 CB ALA 186 38.694 55.385 39.959 1.00 0.00 C

ATOM 2940 HB1 ALA 186 39.717 55.168 39.649 1.00 0.00 H

ATOM 2941 HB2 ALA 186 38.219 56.080 39.267 1.00 0.00 H

ATOM 2942 HB3 ALA 186 38.693 55.669 41.011 1.00 0.00 H

ATOM 2943 C ALA 186 36.321 54.510 40.205 1.00 0.00 C

ATOM 2944 O ALA 186 35.807 54.461 41.366 1.00 0.00 O

ATOM 2945 N HIE 187 35.621 54.821 39.066 1.00 0.00 N

ATOM 2946 H HIE 187 36.079 54.916 38.171 1.00 0.00 H

ATOM 2947 CA HIE 187 34.135 55.150 39.079 1.00 0.00 C

ATOM 2948 HA HIE 187 33.981 55.770 39.962 1.00 0.00 H

ATOM 2949 CB HIE 187 33.640 55.992 37.898 1.00 0.00 C

ATOM 2950 HB2 HIE 187 33.592 55.387 36.993 1.00 0.00 H

ATOM 2951 HB3 HIE 187 32.654 56.323 38.223 1.00 0.00 H

ATOM 2952 CG HIE 187 34.406 57.235 37.564 1.00 0.00 C

ATOM 2953 ND1 HIE 187 34.241 58.422 38.307 1.00 0.00 N

ATOM 2954 CE1 HIE 187 34.901 59.333 37.607 1.00 0.00 C

ATOM 2955 HE1 HIE 187 34.849 60.341 37.993 1.00 0.00 H

ATOM 2956 NE2 HIE 187 35.429 58.810 36.529 1.00 0.00 N

ATOM 2957 HE2 HIE 187 35.923 59.340 35.825 1.00 0.00 H

ATOM 2958 CD2 HIE 187 35.120 57.455 36.472 1.00 0.00 C

ATOM 2959 HD2 HIE 187 35.248 56.765 35.651 1.00 0.00 H

ATOM 2960 C HIE 187 33.205 53.942 39.155 1.00 0.00 C

ATOM 2961 O HIE 187 32.063 54.101 39.712 1.00 0.00 O

ATOM 2962 N SER 188 33.629 52.796 38.661 1.00 0.00 N

ATOM 2963 H SER 188 34.550 52.782 38.247 1.00 0.00 H

ATOM 2964 CA SER 188 32.937 51.548 39.004 1.00 0.00 C

ATOM 2965 HA SER 188 31.864 51.602 38.825 1.00 0.00 H

ATOM 2966 CB SER 188 33.576 50.460 38.252 1.00 0.00 C

ATOM 2967 HB2 SER 188 34.655 50.412 38.397 1.00 0.00 H

ATOM 2968 HB3 SER 188 33.100 49.535 38.578 1.00 0.00 H

ATOM 2969 OG SER 188 33.208 50.456 36.854 1.00 0.00 O

ATOM 2970 HG SER 188 32.264 50.286 36.874 1.00 0.00 H

ATOM 2971 C SER 188 33.017 51.163 40.518 1.00 0.00 C

ATOM 2972 O SER 188 32.036 50.612 41.056 1.00 0.00 O

ATOM 2973 N GLU 189 34.172 51.370 41.125 1.00 0.00 N

ATOM 2974 H GLU 189 34.894 51.694 40.497 1.00 0.00 H

ATOM 2975 CA GLU 189 34.576 50.719 42.335 1.00 0.00 C

ATOM 2976 HA GLU 189 33.943 49.874 42.607 1.00 0.00 H

ATOM 2977 CB GLU 189 36.047 50.125 42.250 1.00 0.00 C

ATOM 2978 HB2 GLU 189 36.664 50.957 41.912 1.00 0.00 H

ATOM 2979 HB3 GLU 189 36.358 49.692 43.202 1.00 0.00 H

ATOM 2980 CG GLU 189 36.113 49.050 41.180 1.00 0.00 C

ATOM 2981 HG2 GLU 189 35.486 48.255 41.583 1.00 0.00 H

ATOM 2982 HG3 GLU 189 35.598 49.408 40.289 1.00 0.00 H

ATOM 2983 CD GLU 189 37.564 48.571 40.965 1.00 0.00 C

ATOM 2984 OE1 GLU 189 38.425 48.727 41.835 1.00 0.00 O

ATOM 2985 OE2 GLU 189 37.875 47.813 40.042 1.00 0.00 O

ATOM 2986 C GLU 189 34.494 51.651 43.521 1.00 0.00 C

ATOM 2987 O GLU 189 34.486 51.153 44.612 1.00 0.00 O

ATOM 2988 N SER 190 34.431 53.001 43.324 1.00 0.00 N

ATOM 2989 H SER 190 34.412 53.402 42.397 1.00 0.00 H

ATOM 2990 CA SER 190 34.441 53.918 44.428 1.00 0.00 C

ATOM 2991 HA SER 190 34.160 53.364 45.324 1.00 0.00 H

ATOM 2992 CB SER 190 35.967 54.354 44.626 1.00 0.00 C

ATOM 2993 HB2 SER 190 35.994 54.527 45.702 1.00 0.00 H

ATOM 2994 HB3 SER 190 36.549 53.444 44.477 1.00 0.00 H

ATOM 2995 OG SER 190 36.315 55.468 43.823 1.00 0.00 O

ATOM 2996 HG SER 190 36.093 55.162 42.941 1.00 0.00 H

ATOM 2997 C SER 190 33.479 55.063 44.231 1.00 0.00 C

ATOM 2998 O SER 190 33.287 55.508 43.133 1.00 0.00 O

ATOM 2999 N ILE 191 32.786 55.404 45.360 1.00 0.00 N

ATOM 3000 H ILE 191 33.012 55.011 46.263 1.00 0.00 H

ATOM 3001 CA ILE 191 31.844 56.564 45.277 1.00 0.00 C

ATOM 3002 HA ILE 191 31.771 56.879 44.236 1.00 0.00 H

ATOM 3003 CB ILE 191 30.347 56.071 45.633 1.00 0.00 C

ATOM 3004 HB ILE 191 29.670 56.921 45.716 1.00 0.00 H

ATOM 3005 CG2 ILE 191 29.977 55.141 44.489 1.00 0.00 C

ATOM 3006 HG21 ILE 191 28.936 54.828 44.561 1.00 0.00 H

ATOM 3007 HG22 ILE 191 30.093 55.622 43.518 1.00 0.00 H

ATOM 3008 HG23 ILE 191 30.621 54.261 44.486 1.00 0.00 H

ATOM 3009 CG1 ILE 191 30.235 55.255 46.898 1.00 0.00 C

ATOM 3010 HG12 ILE 191 30.849 54.355 46.877 1.00 0.00 H

ATOM 3011 HG13 ILE 191 30.669 55.878 47.680 1.00 0.00 H

ATOM 3012 CD1 ILE 191 28.844 54.830 47.337 1.00 0.00 C

ATOM 3013 HD11 ILE 191 28.133 55.637 47.163 1.00 0.00 H

ATOM 3014 HD12 ILE 191 28.590 53.880 46.868 1.00 0.00 H

ATOM 3015 HD13 ILE 191 28.887 54.542 48.388 1.00 0.00 H

ATOM 3016 C ILE 191 32.175 57.748 46.159 1.00 0.00 C

ATOM 3017 O ILE 191 32.792 57.545 47.201 1.00 0.00 O

ATOM 3018 N LYS 192 31.852 58.935 45.710 1.00 0.00 N

ATOM 3019 H LYS 192 31.097 59.058 45.050 1.00 0.00 H

ATOM 3020 CA LYS 192 32.490 60.224 46.037 1.00 0.00 C

ATOM 3021 HA LYS 192 33.538 60.004 45.835 1.00 0.00 H

ATOM 3022 CB LYS 192 32.120 61.417 45.155 1.00 0.00 C

ATOM 3023 HB2 LYS 192 31.128 61.738 45.474 1.00 0.00 H

ATOM 3024 HB3 LYS 192 32.815 62.229 45.370 1.00 0.00 H

ATOM 3025 CG LYS 192 32.231 61.242 43.644 1.00 0.00 C

ATOM 3026 HG2 LYS 192 33.226 60.891 43.371 1.00 0.00 H

ATOM 3027 HG3 LYS 192 31.558 60.431 43.366 1.00 0.00 H

ATOM 3028 CD LYS 192 31.882 62.490 42.741 1.00 0.00 C

ATOM 3029 HD2 LYS 192 30.875 62.808 43.014 1.00 0.00 H

ATOM 3030 HD3 LYS 192 32.480 63.297 43.164 1.00 0.00 H

ATOM 3031 CE LYS 192 32.177 62.366 41.220 1.00 0.00 C

ATOM 3032 HE2 LYS 192 31.988 63.414 40.989 1.00 0.00 H

ATOM 3033 HE3 LYS 192 33.171 61.940 41.084 1.00 0.00 H

ATOM 3034 NZ LYS 192 31.199 61.635 40.393 1.00 0.00 N

ATOM 3035 HZ1 LYS 192 31.476 60.678 40.227 1.00 0.00 H

ATOM 3036 HZ2 LYS 192 30.241 61.720 40.700 1.00 0.00 H

ATOM 3037 HZ3 LYS 192 31.220 62.009 39.455 1.00 0.00 H

ATOM 3038 C LYS 192 32.271 60.560 47.508 1.00 0.00 C

ATOM 3039 O LYS 192 33.207 60.912 48.237 1.00 0.00 O

ATOM 3040 N LYS 193 31.026 60.416 48.039 1.00 0.00 N

ATOM 3041 H LYS 193 30.268 60.075 47.465 1.00 0.00 H

ATOM 3042 CA LYS 193 30.851 60.662 49.482 1.00 0.00 C

ATOM 3043 HA LYS 193 31.223 61.659 49.718 1.00 0.00 H

ATOM 3044 CB LYS 193 29.316 60.838 49.737 1.00 0.00 C

ATOM 3045 HB2 LYS 193 28.840 59.908 49.427 1.00 0.00 H

ATOM 3046 HB3 LYS 193 29.087 61.062 50.779 1.00 0.00 H

ATOM 3047 CG LYS 193 28.689 62.006 48.878 1.00 0.00 C

ATOM 3048 HG2 LYS 193 29.125 62.967 49.153 1.00 0.00 H

ATOM 3049 HG3 LYS 193 28.982 61.939 47.830 1.00 0.00 H

ATOM 3050 CD LYS 193 27.210 62.324 49.011 1.00 0.00 C

ATOM 3051 HD2 LYS 193 26.604 61.529 48.576 1.00 0.00 H

ATOM 3052 HD3 LYS 193 26.946 62.308 50.068 1.00 0.00 H

ATOM 3053 CE LYS 193 26.721 63.630 48.418 1.00 0.00 C

ATOM 3054 HE2 LYS 193 25.651 63.593 48.623 1.00 0.00 H

ATOM 3055 HE3 LYS 193 27.075 64.494 48.980 1.00 0.00 H

ATOM 3056 NZ LYS 193 26.742 63.747 46.881 1.00 0.00 N

ATOM 3057 HZ1 LYS 193 27.698 63.957 46.630 1.00 0.00 H

ATOM 3058 HZ2 LYS 193 26.419 62.889 46.457 1.00 0.00 H

ATOM 3059 HZ3 LYS 193 26.191 64.491 46.478 1.00 0.00 H

ATOM 3060 C LYS 193 31.665 59.682 50.398 1.00 0.00 C

ATOM 3061 O LYS 193 32.320 60.071 51.356 1.00 0.00 O

ATOM 3062 N LYS 194 31.718 58.417 49.902 1.00 0.00 N

ATOM 3063 H LYS 194 31.266 58.259 49.013 1.00 0.00 H

ATOM 3064 CA LYS 194 32.503 57.361 50.622 1.00 0.00 C

ATOM 3065 HA LYS 194 32.193 57.376 51.667 1.00 0.00 H

ATOM 3066 CB LYS 194 32.386 55.971 50.031 1.00 0.00 C

ATOM 3067 HB2 LYS 194 31.359 55.607 50.021 1.00 0.00 H

ATOM 3068 HB3 LYS 194 32.761 55.906 49.009 1.00 0.00 H

ATOM 3069 CG LYS 194 33.178 54.837 50.815 1.00 0.00 C

ATOM 3070 HG2 LYS 194 34.266 54.906 50.808 1.00 0.00 H

ATOM 3071 HG3 LYS 194 32.904 54.821 51.870 1.00 0.00 H

ATOM 3072 CD LYS 194 32.760 53.451 50.358 1.00 0.00 C

ATOM 3073 HD2 LYS 194 31.781 53.187 50.758 1.00 0.00 H

ATOM 3074 HD3 LYS 194 32.635 53.413 49.276 1.00 0.00 H

ATOM 3075 CE LYS 194 33.771 52.430 50.888 1.00 0.00 C

ATOM 3076 HE2 LYS 194 34.811 52.685 50.684 1.00 0.00 H

ATOM 3077 HE3 LYS 194 33.674 52.513 51.970 1.00 0.00 H

ATOM 3078 NZ LYS 194 33.332 51.043 50.460 1.00 0.00 N

ATOM 3079 HZ1 LYS 194 32.412 50.725 50.728 1.00 0.00 H

ATOM 3080 HZ2 LYS 194 33.393 50.966 49.455 1.00 0.00 H

ATOM 3081 HZ3 LYS 194 34.016 50.375 50.786 1.00 0.00 H

ATOM 3082 C LYS 194 34.008 57.752 50.699 1.00 0.00 C

ATOM 3083 O LYS 194 34.615 57.655 51.731 1.00 0.00 O

ATOM 3084 N VAL 195 34.600 58.295 49.605 1.00 0.00 N

ATOM 3085 H VAL 195 33.968 58.325 48.818 1.00 0.00 H

ATOM 3086 CA VAL 195 35.992 58.734 49.558 1.00 0.00 C

ATOM 3087 HA VAL 195 36.688 57.974 49.911 1.00 0.00 H

ATOM 3088 CB VAL 195 36.426 58.998 48.135 1.00 0.00 C

ATOM 3089 HB VAL 195 35.627 59.632 47.751 1.00 0.00 H

ATOM 3090 CG1 VAL 195 37.803 59.735 47.927 1.00 0.00 C

ATOM 3091 HG11 VAL 195 37.780 60.646 48.525 1.00 0.00 H

ATOM 3092 HG12 VAL 195 38.595 59.005 48.091 1.00 0.00 H

ATOM 3093 HG13 VAL 195 37.919 60.013 46.879 1.00 0.00 H

ATOM 3094 CG2 VAL 195 36.422 57.630 47.375 1.00 0.00 C

ATOM 3095 HG21 VAL 195 36.593 57.876 46.327 1.00 0.00 H

ATOM 3096 HG22 VAL 195 37.256 57.006 47.696 1.00 0.00 H

ATOM 3097 HG23 VAL 195 35.435 57.179 47.485 1.00 0.00 H

ATOM 3098 C VAL 195 36.212 59.989 50.358 1.00 0.00 C

ATOM 3099 O VAL 195 37.210 60.086 51.029 1.00 0.00 O

ATOM 3100 N LEU 196 35.263 60.945 50.317 1.00 0.00 N

ATOM 3101 H LEU 196 34.541 60.718 49.648 1.00 0.00 H

ATOM 3102 CA LEU 196 35.304 62.200 51.114 1.00 0.00 C

ATOM 3103 HA LEU 196 36.227 62.737 50.899 1.00 0.00 H

ATOM 3104 CB LEU 196 34.084 62.978 50.666 1.00 0.00 C

ATOM 3105 HB2 LEU 196 34.124 63.061 49.580 1.00 0.00 H

ATOM 3106 HB3 LEU 196 33.195 62.406 50.930 1.00 0.00 H

ATOM 3107 CG LEU 196 33.972 64.366 51.427 1.00 0.00 C

ATOM 3108 HG LEU 196 34.126 64.206 52.494 1.00 0.00 H

ATOM 3109 CD1 LEU 196 35.152 65.305 50.979 1.00 0.00 C

ATOM 3110 HD11 LEU 196 35.035 65.549 49.923 1.00 0.00 H

ATOM 3111 HD12 LEU 196 35.238 66.153 51.657 1.00 0.00 H

ATOM 3112 HD13 LEU 196 36.106 64.828 51.205 1.00 0.00 H

ATOM 3113 CD2 LEU 196 32.671 65.104 51.165 1.00 0.00 C

ATOM 3114 HD21 LEU 196 31.863 64.381 51.272 1.00 0.00 H

ATOM 3115 HD22 LEU 196 32.406 65.811 51.952 1.00 0.00 H

ATOM 3116 HD23 LEU 196 32.540 65.608 50.208 1.00 0.00 H

ATOM 3117 C LEU 196 35.179 61.939 52.625 1.00 0.00 C

ATOM 3118 O LEU 196 35.913 62.614 53.343 1.00 0.00 O

ATOM 3119 N GLU 197 34.482 60.920 53.059 1.00 0.00 N

ATOM 3120 H GLU 197 33.839 60.444 52.442 1.00 0.00 H

ATOM 3121 CA GLU 197 34.227 60.700 54.508 1.00 0.00 C

ATOM 3122 HA GLU 197 34.340 61.679 54.974 1.00 0.00 H

ATOM 3123 CB GLU 197 32.795 60.274 54.728 1.00 0.00 C

ATOM 3124 HB2 GLU 197 32.137 60.966 54.204 1.00 0.00 H

ATOM 3125 HB3 GLU 197 32.618 59.351 54.175 1.00 0.00 H

ATOM 3126 CG GLU 197 32.523 60.198 56.271 1.00 0.00 C

ATOM 3127 HG2 GLU 197 33.093 59.352 56.654 1.00 0.00 H

ATOM 3128 HG3 GLU 197 32.830 61.055 56.871 1.00 0.00 H

ATOM 3129 CD GLU 197 31.044 59.883 56.477 1.00 0.00 C

ATOM 3130 OE1 GLU 197 30.446 60.336 57.486 1.00 0.00 O

ATOM 3131 OE2 GLU 197 30.403 59.179 55.637 1.00 0.00 O

ATOM 3132 C GLU 197 35.287 59.770 55.186 1.00 0.00 C

ATOM 3133 O GLU 197 35.491 59.823 56.409 1.00 0.00 O

ATOM 3134 N PHE 198 36.098 59.158 54.355 1.00 0.00 N

ATOM 3135 H PHE 198 35.656 58.959 53.469 1.00 0.00 H

ATOM 3136 CA PHE 198 37.407 58.524 54.591 1.00 0.00 C

ATOM 3137 HA PHE 198 37.430 58.178 55.624 1.00 0.00 H

ATOM 3138 CB PHE 198 37.728 57.283 53.664 1.00 0.00 C

ATOM 3139 HB2 PHE 198 36.828 56.674 53.751 1.00 0.00 H

ATOM 3140 HB3 PHE 198 37.841 57.624 52.635 1.00 0.00 H

ATOM 3141 CG PHE 198 38.917 56.414 54.027 1.00 0.00 C

ATOM 3142 CD1 PHE 198 38.903 55.693 55.248 1.00 0.00 C

ATOM 3143 HD1 PHE 198 38.096 55.814 55.956 1.00 0.00 H

ATOM 3144 CE1 PHE 198 39.902 54.750 55.544 1.00 0.00 C

ATOM 3145 HE1 PHE 198 39.892 54.255 56.504 1.00 0.00 H

ATOM 3146 CZ PHE 198 40.920 54.516 54.633 1.00 0.00 C

ATOM 3147 HZ PHE 198 41.684 53.788 54.864 1.00 0.00 H

ATOM 3148 CE2 PHE 198 40.943 55.268 53.408 1.00 0.00 C

ATOM 3149 HE2 PHE 198 41.788 55.151 52.746 1.00 0.00 H

ATOM 3150 CD2 PHE 198 39.944 56.228 53.111 1.00 0.00 C

ATOM 3151 HD2 PHE 198 39.977 56.774 52.180 1.00 0.00 H

ATOM 3152 C PHE 198 38.534 59.575 54.673 1.00 0.00 C

ATOM 3153 O PHE 198 39.233 59.658 55.675 1.00 0.00 O

ATOM 3154 N LEU 199 38.581 60.426 53.631 1.00 0.00 N

ATOM 3155 H LEU 199 38.041 60.239 52.798 1.00 0.00 H

ATOM 3156 CA LEU 199 39.411 61.604 53.595 1.00 0.00 C

ATOM 3157 HA LEU 199 40.414 61.196 53.466 1.00 0.00 H

ATOM 3158 CB LEU 199 39.267 62.394 52.343 1.00 0.00 C

ATOM 3159 HB2 LEU 199 39.668 61.776 51.539 1.00 0.00 H

ATOM 3160 HB3 LEU 199 38.226 62.447 52.026 1.00 0.00 H

ATOM 3161 CG LEU 199 39.943 63.808 52.362 1.00 0.00 C

ATOM 3162 HG LEU 199 39.817 64.207 53.369 1.00 0.00 H

ATOM 3163 CD1 LEU 199 41.390 63.715 52.001 1.00 0.00 C

ATOM 3164 HD11 LEU 199 41.542 63.005 51.188 1.00 0.00 H

ATOM 3165 HD12 LEU 199 41.763 64.725 51.832 1.00 0.00 H

ATOM 3166 HD13 LEU 199 42.025 63.407 52.832 1.00 0.00 H

ATOM 3167 CD2 LEU 199 39.339 64.730 51.296 1.00 0.00 C

ATOM 3168 HD21 LEU 199 39.664 64.416 50.304 1.00 0.00 H

ATOM 3169 HD22 LEU 199 38.256 64.623 51.339 1.00 0.00 H

ATOM 3170 HD23 LEU 199 39.580 65.784 51.434 1.00 0.00 H

ATOM 3171 C LEU 199 39.224 62.359 54.901 1.00 0.00 C

ATOM 3172 O LEU 199 40.239 62.817 55.433 1.00 0.00 O

ATOM 3173 N ALA 200 38.058 62.550 55.517 1.00 0.00 N

ATOM 3174 H ALA 200 37.239 62.412 54.943 1.00 0.00 H

ATOM 3175 CA ALA 200 37.732 63.201 56.793 1.00 0.00 C

ATOM 3176 HA ALA 200 38.124 64.218 56.788 1.00 0.00 H

ATOM 3177 CB ALA 200 36.284 63.345 56.851 1.00 0.00 C

ATOM 3178 HB1 ALA 200 35.987 64.129 56.154 1.00 0.00 H

ATOM 3179 HB2 ALA 200 35.855 62.364 56.646 1.00 0.00 H

ATOM 3180 HB3 ALA 200 35.998 63.680 57.848 1.00 0.00 H

ATOM 3181 C ALA 200 38.220 62.563 58.140 1.00 0.00 C

ATOM 3182 O ALA 200 38.024 63.191 59.202 1.00 0.00 O

ATOM 3183 N LYS 201 38.737 61.298 58.052 1.00 0.00 N

ATOM 3184 H LYS 201 38.838 60.939 57.114 1.00 0.00 H

ATOM 3185 CA LYS 201 38.900 60.444 59.296 1.00 0.00 C

ATOM 3186 HA LYS 201 37.954 60.184 59.771 1.00 0.00 H

ATOM 3187 CB LYS 201 39.512 59.079 58.954 1.00 0.00 C

ATOM 3188 HB2 LYS 201 38.869 58.629 58.198 1.00 0.00 H

ATOM 3189 HB3 LYS 201 40.565 59.132 58.680 1.00 0.00 H

ATOM 3190 CG LYS 201 39.411 58.012 60.033 1.00 0.00 C

ATOM 3191 HG2 LYS 201 39.876 58.323 60.969 1.00 0.00 H

ATOM 3192 HG3 LYS 201 38.370 57.893 60.335 1.00 0.00 H

ATOM 3193 CD LYS 201 39.979 56.676 59.585 1.00 0.00 C

ATOM 3194 HD2 LYS 201 39.318 56.253 58.829 1.00 0.00 H

ATOM 3195 HD3 LYS 201 40.909 56.821 59.035 1.00 0.00 H

ATOM 3196 CE LYS 201 40.160 55.760 60.815 1.00 0.00 C

ATOM 3197 HE2 LYS 201 41.003 56.152 61.384 1.00 0.00 H

ATOM 3198 HE3 LYS 201 39.227 55.877 61.366 1.00 0.00 H

ATOM 3199 NZ LYS 201 40.376 54.310 60.459 1.00 0.00 N

ATOM 3200 HZ1 LYS 201 39.516 53.810 60.287 1.00 0.00 H

ATOM 3201 HZ2 LYS 201 40.952 54.128 59.650 1.00 0.00 H

ATOM 3202 HZ3 LYS 201 40.814 53.780 61.199 1.00 0.00 H

ATOM 3203 C LYS 201 39.730 61.012 60.450 1.00 0.00 C

ATOM 3204 O LYS 201 39.388 60.770 61.625 1.00 0.00 O

ATOM 3205 N LEU 202 40.773 61.728 60.110 1.00 0.00 N

ATOM 3206 H LEU 202 40.922 61.850 59.119 1.00 0.00 H

ATOM 3207 CA LEU 202 41.872 62.102 60.957 1.00 0.00 C

ATOM 3208 HA LEU 202 41.971 61.380 61.768 1.00 0.00 H

ATOM 3209 CB LEU 202 43.147 62.077 60.088 1.00 0.00 C

ATOM 3210 HB2 LEU 202 43.139 61.013 59.850 1.00 0.00 H

ATOM 3211 HB3 LEU 202 43.064 62.578 59.124 1.00 0.00 H

ATOM 3212 CG LEU 202 44.482 62.531 60.745 1.00 0.00 C

ATOM 3213 HG LEU 202 44.361 63.574 61.036 1.00 0.00 H

ATOM 3214 CD1 LEU 202 44.940 61.677 61.953 1.00 0.00 C

ATOM 3215 HD11 LEU 202 45.233 60.715 61.532 1.00 0.00 H

ATOM 3216 HD12 LEU 202 45.815 62.152 62.397 1.00 0.00 H

ATOM 3217 HD13 LEU 202 44.204 61.715 62.756 1.00 0.00 H

ATOM 3218 CD2 LEU 202 45.456 62.383 59.614 1.00 0.00 C

ATOM 3219 HD21 LEU 202 45.200 62.925 58.704 1.00 0.00 H

ATOM 3220 HD22 LEU 202 46.405 62.763 59.993 1.00 0.00 H

ATOM 3221 HD23 LEU 202 45.714 61.327 59.530 1.00 0.00 H

ATOM 3222 C LEU 202 41.557 63.397 61.572 1.00 0.00 C

ATOM 3223 O LEU 202 42.075 63.613 62.661 1.00 0.00 O

ATOM 3224 N ASN 203 40.705 64.280 60.906 1.00 0.00 N

ATOM 3225 H ASN 203 40.263 63.932 60.067 1.00 0.00 H

ATOM 3226 CA ASN 203 40.110 65.483 61.457 1.00 0.00 C

ATOM 3227 HA ASN 203 40.861 65.875 62.143 1.00 0.00 H

ATOM 3228 CB ASN 203 39.838 66.431 60.306 1.00 0.00 C

ATOM 3229 HB2 ASN 203 40.826 66.518 59.854 1.00 0.00 H

ATOM 3230 HB3 ASN 203 39.116 65.941 59.653 1.00 0.00 H

ATOM 3231 CG ASN 203 39.255 67.769 60.727 1.00 0.00 C

ATOM 3232 OD1 ASN 203 39.949 68.570 61.370 1.00 0.00 O

ATOM 3233 ND2 ASN 203 38.024 68.090 60.461 1.00 0.00 N

ATOM 3234 HD21 ASN 203 37.735 69.037 60.662 1.00 0.00 H

ATOM 3235 HD22 ASN 203 37.371 67.375 60.174 1.00 0.00 H

ATOM 3236 C ASN 203 38.858 65.325 62.370 1.00 0.00 C

ATOM 3237 O ASN 203 38.478 66.237 63.092 1.00 0.00 O

ATOM 3238 N ASN 204 38.298 64.135 62.301 1.00 0.00 N

ATOM 3239 H ASN 204 38.759 63.564 61.607 1.00 0.00 H

ATOM 3240 CA ASN 204 36.964 63.882 62.846 1.00 0.00 C

ATOM 3241 HA ASN 204 36.590 64.773 63.349 1.00 0.00 H

ATOM 3242 CB ASN 204 36.208 63.485 61.507 1.00 0.00 C

ATOM 3243 HB2 ASN 204 36.316 64.282 60.771 1.00 0.00 H

ATOM 3244 HB3 ASN 204 36.613 62.579 61.056 1.00 0.00 H

ATOM 3245 CG ASN 204 34.725 63.301 61.772 1.00 0.00 C

ATOM 3246 OD1 ASN 204 34.134 63.918 62.745 1.00 0.00 O

ATOM 3247 ND2 ASN 204 34.082 62.509 60.894 1.00 0.00 N

ATOM 3248 HD21 ASN 204 33.077 62.444 60.811 1.00 0.00 H

ATOM 3249 HD22 ASN 204 34.661 62.034 60.217 1.00 0.00 H

ATOM 3250 C ASN 204 36.936 62.666 63.894 1.00 0.00 C

ATOM 3251 O ASN 204 35.916 62.641 64.620 1.00 0.00 O

ATOM 3252 N THR 205 37.851 61.671 63.888 1.00 0.00 N

ATOM 3253 H THR 205 38.578 61.676 63.187 1.00 0.00 H

ATOM 3254 CA THR 205 37.943 60.619 64.953 1.00 0.00 C

ATOM 3255 HA THR 205 37.096 60.799 65.615 1.00 0.00 H

ATOM 3256 CB THR 205 37.760 59.220 64.398 1.00 0.00 C

ATOM 3257 HB THR 205 37.777 58.569 65.272 1.00 0.00 H

ATOM 3258 CG2 THR 205 36.419 59.166 63.545 1.00 0.00 C

ATOM 3259 HG21 THR 205 35.937 58.188 63.532 1.00 0.00 H

ATOM 3260 HG22 THR 205 35.692 59.896 63.903 1.00 0.00 H

ATOM 3261 HG23 THR 205 36.532 59.377 62.482 1.00 0.00 H

ATOM 3262 OG1 THR 205 38.870 58.810 63.637 1.00 0.00 O

ATOM 3263 HG1 THR 205 38.902 59.480 62.951 1.00 0.00 H

ATOM 3264 C THR 205 39.261 60.847 65.729 1.00 0.00 C

ATOM 3265 O THR 205 40.308 61.123 65.158 1.00 0.00 O

ATOM 3266 N VAL 206 39.300 60.778 67.070 1.00 0.00 N

ATOM 3267 H VAL 206 38.443 60.566 67.562 1.00 0.00 H

ATOM 3268 CA VAL 206 40.491 60.888 67.949 1.00 0.00 C

ATOM 3269 HA VAL 206 41.185 61.522 67.398 1.00 0.00 H

ATOM 3270 CB VAL 206 40.100 61.632 69.200 1.00 0.00 C

ATOM 3271 HB VAL 206 39.313 61.068 69.702 1.00 0.00 H

ATOM 3272 CG1 VAL 206 41.259 61.693 70.170 1.00 0.00 C

ATOM 3273 HG11 VAL 206 40.789 62.034 71.093 1.00 0.00 H

ATOM 3274 HG12 VAL 206 41.682 60.719 70.418 1.00 0.00 H

ATOM 3275 HG13 VAL 206 42.058 62.298 69.742 1.00 0.00 H

ATOM 3276 CG2 VAL 206 39.610 63.052 68.977 1.00 0.00 C

ATOM 3277 HG21 VAL 206 40.319 63.671 68.427 1.00 0.00 H

ATOM 3278 HG22 VAL 206 38.664 63.014 68.437 1.00 0.00 H

ATOM 3279 HG23 VAL 206 39.545 63.653 69.884 1.00 0.00 H

ATOM 3280 C VAL 206 41.203 59.486 68.212 1.00 0.00 C

ATOM 3281 O VAL 206 40.630 58.634 68.876 1.00 0.00 O

ATOM 3282 N PRO 207 42.474 59.300 67.755 1.00 0.00 N

ATOM 3283 CD PRO 207 43.202 60.328 67.077 1.00 0.00 C

ATOM 3284 HD2 PRO 207 43.241 61.144 67.799 1.00 0.00 H

ATOM 3285 HD3 PRO 207 42.700 60.604 66.150 1.00 0.00 H

ATOM 3286 CG PRO 207 44.597 59.702 66.775 1.00 0.00 C

ATOM 3287 HG2 PRO 207 45.354 60.046 67.479 1.00 0.00 H

ATOM 3288 HG3 PRO 207 44.961 60.069 65.815 1.00 0.00 H

ATOM 3289 CB PRO 207 44.415 58.243 66.944 1.00 0.00 C

ATOM 3290 HB2 PRO 207 45.325 57.662 67.093 1.00 0.00 H

ATOM 3291 HB3 PRO 207 44.093 57.944 65.947 1.00 0.00 H

ATOM 3292 CA PRO 207 43.314 58.169 67.996 1.00 0.00 C

ATOM 3293 HA PRO 207 42.784 57.234 67.811 1.00 0.00 H

ATOM 3294 C PRO 207 43.913 57.999 69.396 1.00 0.00 C

ATOM 3295 O PRO 207 44.058 58.927 70.203 1.00 0.00 O

ATOM 3296 N SER 208 44.383 56.814 69.723 1.00 0.00 N

ATOM 3297 H SER 208 44.423 56.118 68.993 1.00 0.00 H

ATOM 3298 CA SER 208 44.783 56.315 71.092 1.00 0.00 C

ATOM 3299 HA SER 208 44.197 56.917 71.787 1.00 0.00 H

ATOM 3300 CB SER 208 44.443 54.861 71.352 1.00 0.00 C

ATOM 3301 HB2 SER 208 44.650 54.613 72.393 1.00 0.00 H

ATOM 3302 HB3 SER 208 43.407 54.632 71.103 1.00 0.00 H

ATOM 3303 OG SER 208 45.202 53.909 70.638 1.00 0.00 O

ATOM 3304 HG SER 208 45.127 54.056 69.693 1.00 0.00 H

ATOM 3305 C SER 208 46.275 56.625 71.394 1.00 0.00 C

ATOM 3306 O SER 208 47.064 56.845 70.488 1.00 0.00 O

ATOM 3307 N SER 209 46.610 56.812 72.650 1.00 0.00 N

ATOM 3308 H SER 209 46.091 56.315 73.360 1.00 0.00 H

ATOM 3309 CA SER 209 47.948 57.248 73.022 1.00 0.00 C

ATOM 3310 HA SER 209 48.708 57.092 72.257 1.00 0.00 H

ATOM 3311 CB SER 209 48.055 58.789 73.450 1.00 0.00 C

ATOM 3312 HB2 SER 209 49.032 59.012 73.879 1.00 0.00 H

ATOM 3313 HB3 SER 209 48.043 59.423 72.563 1.00 0.00 H

ATOM 3314 OG SER 209 47.102 59.149 74.457 1.00 0.00 O

ATOM 3315 HG SER 209 46.213 58.827 74.293 1.00 0.00 H

ATOM 3316 C SER 209 48.496 56.484 74.239 1.00 0.00 C

ATOM 3317 O SER 209 47.695 55.901 75.041 1.00 0.00 O

ATOM 3318 N PHE 210 49.843 56.440 74.355 1.00 0.00 N

ATOM 3319 H PHE 210 50.373 57.110 73.817 1.00 0.00 H

ATOM 3320 CA PHE 210 50.585 55.887 75.508 1.00 0.00 C

ATOM 3321 HA PHE 210 49.985 54.997 75.701 1.00 0.00 H

ATOM 3322 CB PHE 210 52.001 55.252 75.291 1.00 0.00 C

ATOM 3323 HB2 PHE 210 52.710 56.077 75.213 1.00 0.00 H

ATOM 3324 HB3 PHE 210 52.254 54.543 76.079 1.00 0.00 H

ATOM 3325 CG PHE 210 52.137 54.493 73.923 1.00 0.00 C

ATOM 3326 CD1 PHE 210 51.491 53.249 73.772 1.00 0.00 C

ATOM 3327 HD1 PHE 210 50.981 52.812 74.618 1.00 0.00 H

ATOM 3328 CE1 PHE 210 51.670 52.520 72.597 1.00 0.00 C

ATOM 3329 HE1 PHE 210 51.293 51.525 72.412 1.00 0.00 H

ATOM 3330 CZ PHE 210 52.490 52.996 71.526 1.00 0.00 C

ATOM 3331 HZ PHE 210 52.577 52.482 70.580 1.00 0.00 H

ATOM 3332 CE2 PHE 210 53.103 54.269 71.702 1.00 0.00 C

ATOM 3333 HE2 PHE 210 53.754 54.643 70.926 1.00 0.00 H

ATOM 3334 CD2 PHE 210 52.999 54.953 72.903 1.00 0.00 C

ATOM 3335 HD2 PHE 210 53.435 55.933 73.028 1.00 0.00 H

ATOM 3336 C PHE 210 50.524 56.770 76.836 1.00 0.00 C

ATOM 3337 O PHE 210 50.408 57.992 76.667 1.00 0.00 O

ATOM 3338 N PRO 211 50.533 56.150 78.018 1.00 0.00 N

ATOM 3339 CD PRO 211 50.372 54.698 78.194 1.00 0.00 C

ATOM 3340 HD2 PRO 211 51.100 54.076 77.674 1.00 0.00 H

ATOM 3341 HD3 PRO 211 49.431 54.461 77.699 1.00 0.00 H

ATOM 3342 CG PRO 211 50.098 54.386 79.669 1.00 0.00 C

ATOM 3343 HG2 PRO 211 50.992 53.930 80.095 1.00 0.00 H

ATOM 3344 HG3 PRO 211 49.206 53.762 79.717 1.00 0.00 H

ATOM 3345 CB PRO 211 49.975 55.793 80.264 1.00 0.00 C

ATOM 3346 HB2 PRO 211 50.406 55.849 81.264 1.00 0.00 H

ATOM 3347 HB3 PRO 211 48.908 56.001 80.341 1.00 0.00 H

ATOM 3348 CA PRO 211 50.574 56.777 79.335 1.00 0.00 C

ATOM 3349 HA PRO 211 50.054 57.734 79.382 1.00 0.00 H

ATOM 3350 C PRO 211 51.979 57.069 79.856 1.00 0.00 C

ATOM 3351 O PRO 211 53.032 56.576 79.478 1.00 0.00 O

ATOM 3352 N SER 212 52.058 57.979 80.885 1.00 0.00 N

ATOM 3353 H SER 212 51.199 58.449 81.135 1.00 0.00 H

ATOM 3354 CA SER 212 53.322 58.684 81.214 1.00 0.00 C

ATOM 3355 HA SER 212 53.788 58.970 80.271 1.00 0.00 H

ATOM 3356 CB SER 212 53.000 60.084 81.722 1.00 0.00 C

ATOM 3357 HB2 SER 212 52.736 59.939 82.770 1.00 0.00 H

ATOM 3358 HB3 SER 212 53.861 60.732 81.557 1.00 0.00 H

ATOM 3359 OG SER 212 51.914 60.633 81.005 1.00 0.00 O

ATOM 3360 HG SER 212 51.952 60.419 80.070 1.00 0.00 H

ATOM 3361 C SER 212 54.289 57.957 82.050 1.00 0.00 C

ATOM 3362 O SER 212 53.985 57.172 82.958 1.00 0.00 O

ATOM 3363 N TRP 213 55.622 58.174 81.845 1.00 0.00 N

ATOM 3364 H TRP 213 55.787 58.726 81.016 1.00 0.00 H

ATOM 3365 CA TRP 213 56.613 57.191 82.286 1.00 0.00 C

ATOM 3366 HA TRP 213 56.334 56.163 82.054 1.00 0.00 H

ATOM 3367 CB TRP 213 57.942 57.345 81.544 1.00 0.00 C

ATOM 3368 HB2 TRP 213 57.838 57.061 80.497 1.00 0.00 H

ATOM 3369 HB3 TRP 213 58.278 58.379 81.619 1.00 0.00 H

ATOM 3370 CG TRP 213 59.051 56.422 81.927 1.00 0.00 C

ATOM 3371 CD1 TRP 213 59.077 55.170 81.500 1.00 0.00 C

ATOM 3372 HD1 TRP 213 58.386 54.602 80.895 1.00 0.00 H

ATOM 3373 NE1 TRP 213 60.298 54.556 81.926 1.00 0.00 N

ATOM 3374 HE1 TRP 213 60.651 53.632 81.721 1.00 0.00 H

ATOM 3375 CE2 TRP 213 60.978 55.431 82.779 1.00 0.00 C

ATOM 3376 CZ2 TRP 213 62.195 55.346 83.416 1.00 0.00 C

ATOM 3377 HZ2 TRP 213 62.765 54.431 83.487 1.00 0.00 H

ATOM 3378 CH2 TRP 213 62.672 56.498 84.115 1.00 0.00 C

ATOM 3379 HH2 TRP 213 63.578 56.425 84.698 1.00 0.00 H

ATOM 3380 CZ3 TRP 213 62.024 57.758 83.947 1.00 0.00 C

ATOM 3381 HZ3 TRP 213 62.305 58.611 84.548 1.00 0.00 H

ATOM 3382 CE3 TRP 213 60.817 57.813 83.268 1.00 0.00 C

ATOM 3383 HE3 TRP 213 60.261 58.732 83.156 1.00 0.00 H

ATOM 3384 CD2 TRP 213 60.289 56.671 82.665 1.00 0.00 C

ATOM 3385 C TRP 213 56.827 57.189 83.811 1.00 0.00 C

ATOM 3386 O TRP 213 57.146 56.152 84.465 1.00 0.00 O

ATOM 3387 N TYR 214 56.614 58.421 84.329 1.00 0.00 N

ATOM 3388 H TYR 214 56.618 59.150 83.629 1.00 0.00 H

ATOM 3389 CA TYR 214 56.490 58.628 85.774 1.00 0.00 C

ATOM 3390 HA TYR 214 57.236 57.927 86.146 1.00 0.00 H

ATOM 3391 CB TYR 214 56.991 60.086 86.069 1.00 0.00 C

ATOM 3392 HB2 TYR 214 56.544 60.805 85.381 1.00 0.00 H

ATOM 3393 HB3 TYR 214 56.564 60.433 87.010 1.00 0.00 H

ATOM 3394 CG TYR 214 58.496 60.415 86.163 1.00 0.00 C

ATOM 3395 CD1 TYR 214 59.137 60.954 85.005 1.00 0.00 C

ATOM 3396 HD1 TYR 214 58.699 61.087 84.027 1.00 0.00 H

ATOM 3397 CE1 TYR 214 60.509 61.224 85.087 1.00 0.00 C

ATOM 3398 HE1 TYR 214 61.094 61.541 84.236 1.00 0.00 H

ATOM 3399 CZ TYR 214 61.224 60.966 86.289 1.00 0.00 C

ATOM 3400 OH TYR 214 62.507 61.362 86.265 1.00 0.00 O

ATOM 3401 HH TYR 214 62.850 61.636 85.411 1.00 0.00 H

ATOM 3402 CE2 TYR 214 60.592 60.423 87.415 1.00 0.00 C

ATOM 3403 HE2 TYR 214 61.222 60.362 88.290 1.00 0.00 H

ATOM 3404 CD2 TYR 214 59.184 60.231 87.372 1.00 0.00 C

ATOM 3405 HD2 TYR 214 58.607 59.989 88.252 1.00 0.00 H

ATOM 3406 C TYR 214 55.222 58.279 86.512 1.00 0.00 C

ATOM 3407 O TYR 214 55.281 58.222 87.743 1.00 0.00 O

ATOM 3408 N LYS 215 54.114 57.988 85.847 1.00 0.00 N

ATOM 3409 H LYS 215 54.156 58.125 84.847 1.00 0.00 H

ATOM 3410 CA LYS 215 52.789 57.639 86.448 1.00 0.00 C

ATOM 3411 HA LYS 215 52.919 57.802 87.518 1.00 0.00 H

ATOM 3412 CB LYS 215 51.669 58.544 85.901 1.00 0.00 C

ATOM 3413 HB2 LYS 215 52.053 59.543 86.111 1.00 0.00 H

ATOM 3414 HB3 LYS 215 51.671 58.429 84.817 1.00 0.00 H

ATOM 3415 CG LYS 215 50.344 58.194 86.576 1.00 0.00 C

ATOM 3416 HG2 LYS 215 49.902 57.251 86.256 1.00 0.00 H

ATOM 3417 HG3 LYS 215 50.529 57.986 87.630 1.00 0.00 H

ATOM 3418 CD LYS 215 49.354 59.381 86.448 1.00 0.00 C

ATOM 3419 HD2 LYS 215 48.426 59.063 86.923 1.00 0.00 H

ATOM 3420 HD3 LYS 215 49.578 60.316 86.960 1.00 0.00 H

ATOM 3421 CE LYS 215 49.035 59.772 84.985 1.00 0.00 C

ATOM 3422 HE2 LYS 215 49.942 59.869 84.388 1.00 0.00 H

ATOM 3423 HE3 LYS 215 48.379 59.016 84.554 1.00 0.00 H

ATOM 3424 NZ LYS 215 48.413 61.036 84.897 1.00 0.00 N

ATOM 3425 HZ1 LYS 215 47.688 61.174 85.586 1.00 0.00 H

ATOM 3426 HZ2 LYS 215 49.077 61.797 84.911 1.00 0.00 H

ATOM 3427 HZ3 LYS 215 47.872 61.188 84.059 1.00 0.00 H

ATOM 3428 C LYS 215 52.504 56.144 86.343 1.00 0.00 C

ATOM 3429 O LYS 215 52.103 55.584 87.336 1.00 0.00 O

ATOM 3430 N ASP 216 52.692 55.520 85.207 1.00 0.00 N

ATOM 3431 H ASP 216 53.179 55.969 84.445 1.00 0.00 H

ATOM 3432 CA ASP 216 52.171 54.181 84.841 1.00 0.00 C

ATOM 3433 HA ASP 216 51.404 53.870 85.551 1.00 0.00 H

ATOM 3434 CB ASP 216 51.513 54.299 83.396 1.00 0.00 C

ATOM 3435 HB2 ASP 216 50.991 55.256 83.360 1.00 0.00 H

ATOM 3436 HB3 ASP 216 52.251 54.311 82.594 1.00 0.00 H

ATOM 3437 CG ASP 216 50.459 53.225 83.189 1.00 0.00 C

ATOM 3438 OD1 ASP 216 49.351 53.351 83.654 1.00 0.00 O

ATOM 3439 OD2 ASP 216 50.793 52.234 82.483 1.00 0.00 O

ATOM 3440 C ASP 216 53.165 53.105 84.766 1.00 0.00 C

ATOM 3441 O ASP 216 52.731 51.993 84.923 1.00 0.00 O

ATOM 3442 N ALA 217 54.402 53.337 84.301 1.00 0.00 N

ATOM 3443 H ALA 217 54.649 54.317 84.310 1.00 0.00 H

ATOM 3444 CA ALA 217 55.384 52.458 83.680 1.00 0.00 C

ATOM 3445 HA ALA 217 54.823 51.783 83.033 1.00 0.00 H

ATOM 3446 CB ALA 217 56.166 53.318 82.734 1.00 0.00 C

ATOM 3447 HB1 ALA 217 56.898 52.843 82.081 1.00 0.00 H

ATOM 3448 HB2 ALA 217 55.446 53.849 82.111 1.00 0.00 H

ATOM 3449 HB3 ALA 217 56.576 54.136 83.327 1.00 0.00 H

ATOM 3450 C ALA 217 56.198 51.698 84.803 1.00 0.00 C

ATOM 3451 O ALA 217 57.121 50.993 84.411 1.00 0.00 O

TER

ATOM 3506 N GLN 221 63.527 58.824 48.275 1.00 0.00 N

ATOM 3507 H1 GLN 221 62.595 58.435 48.260 1.00 0.00 H

ATOM 3508 H2 GLN 221 64.014 58.185 47.664 1.00 0.00 H

ATOM 3509 H3 GLN 221 63.575 59.696 47.768 1.00 0.00 H

ATOM 3510 CA GLN 221 64.136 58.796 49.598 1.00 0.00 C

ATOM 3511 HA GLN 221 65.188 59.014 49.414 1.00 0.00 H

ATOM 3512 CB GLN 221 63.541 59.880 50.491 1.00 0.00 C

ATOM 3513 HB2 GLN 221 63.610 60.890 50.087 1.00 0.00 H

ATOM 3514 HB3 GLN 221 62.487 59.641 50.632 1.00 0.00 H

ATOM 3515 CG GLN 221 64.185 59.991 51.907 1.00 0.00 C

ATOM 3516 HG2 GLN 221 63.356 60.306 52.541 1.00 0.00 H

ATOM 3517 HG3 GLN 221 64.444 58.975 52.203 1.00 0.00 H

ATOM 3518 CD GLN 221 65.402 60.950 51.967 1.00 0.00 C

ATOM 3519 OE1 GLN 221 65.883 61.445 50.934 1.00 0.00 O

ATOM 3520 NE2 GLN 221 65.957 61.053 53.187 1.00 0.00 N

ATOM 3521 HE21 GLN 221 66.690 61.733 53.326 1.00 0.00 H

ATOM 3522 HE22 GLN 221 65.553 60.568 53.976 1.00 0.00 H

ATOM 3523 C GLN 221 63.985 57.466 50.219 1.00 0.00 C

ATOM 3524 O GLN 221 62.870 57.004 50.410 1.00 0.00 O

ATOM 3525 N ARG 222 65.094 56.808 50.508 1.00 0.00 N

ATOM 3526 H ARG 222 66.009 57.191 50.315 1.00 0.00 H

ATOM 3527 CA ARG 222 65.096 55.552 51.172 1.00 0.00 C

ATOM 3528 HA ARG 222 64.299 54.896 50.824 1.00 0.00 H

ATOM 3529 CB ARG 222 66.412 54.761 50.916 1.00 0.00 C

ATOM 3530 HB2 ARG 222 67.259 55.426 51.087 1.00 0.00 H

ATOM 3531 HB3 ARG 222 66.452 53.983 51.679 1.00 0.00 H

ATOM 3532 CG ARG 222 66.551 54.113 49.520 1.00 0.00 C

ATOM 3533 HG2 ARG 222 65.732 53.423 49.316 1.00 0.00 H

ATOM 3534 HG3 ARG 222 66.560 54.941 48.811 1.00 0.00 H

ATOM 3535 CD ARG 222 67.892 53.342 49.512 1.00 0.00 C

ATOM 3536 HD2 ARG 222 68.688 54.055 49.727 1.00 0.00 H

ATOM 3537 HD3 ARG 222 67.820 52.624 50.329 1.00 0.00 H

ATOM 3538 NE ARG 222 68.163 52.636 48.271 1.00 0.00 N

ATOM 3539 HE ARG 222 67.652 52.873 47.432 1.00 0.00 H

ATOM 3540 CZ ARG 222 68.975 51.639 48.082 1.00 0.00 C

ATOM 3541 NH1 ARG 222 69.199 51.135 46.901 1.00 0.00 N

ATOM 3542 HH11 ARG 222 68.563 51.564 46.244 1.00 0.00 H

ATOM 3543 HH12 ARG 222 69.823 50.359 46.732 1.00 0.00 H

ATOM 3544 NH2 ARG 222 69.757 51.136 49.006 1.00 0.00 N

ATOM 3545 HH21 ARG 222 69.686 51.447 49.964 1.00 0.00 H

ATOM 3546 HH22 ARG 222 70.478 50.500 48.695 1.00 0.00 H

ATOM 3547 C ARG 222 64.949 55.683 52.718 1.00 0.00 C

ATOM 3548 O ARG 222 65.276 56.721 53.305 1.00 0.00 O

ATOM 3549 N ASN 223 64.287 54.766 53.427 1.00 0.00 N

ATOM 3550 H ASN 223 64.077 53.946 52.876 1.00 0.00 H

ATOM 3551 CA ASN 223 63.798 54.837 54.858 1.00 0.00 C

ATOM 3552 HA ASN 223 62.934 54.202 55.058 1.00 0.00 H

ATOM 3553 CB ASN 223 64.905 54.195 55.723 1.00 0.00 C

ATOM 3554 HB2 ASN 223 65.108 53.178 55.389 1.00 0.00 H

ATOM 3555 HB3 ASN 223 65.800 54.807 55.609 1.00 0.00 H

ATOM 3556 CG ASN 223 64.426 54.120 57.170 1.00 0.00 C

ATOM 3557 OD1 ASN 223 63.288 53.721 57.518 1.00 0.00 O

ATOM 3558 ND2 ASN 223 65.254 54.616 58.024 1.00 0.00 N

ATOM 3559 HD21 ASN 223 64.810 54.854 58.899 1.00 0.00 H

ATOM 3560 HD22 ASN 223 66.119 55.081 57.786 1.00 0.00 H

ATOM 3561 C ASN 223 63.293 56.283 55.240 1.00 0.00 C

ATOM 3562 O ASN 223 64.044 57.075 55.839 1.00 0.00 O

ATOM 3563 N PRO 224 62.062 56.579 54.785 1.00 0.00 N

ATOM 3564 CD PRO 224 61.186 55.733 53.995 1.00 0.00 C

ATOM 3565 HD2 PRO 224 60.744 54.957 54.619 1.00 0.00 H

ATOM 3566 HD3 PRO 224 61.824 55.291 53.230 1.00 0.00 H

ATOM 3567 CG PRO 224 60.105 56.594 53.315 1.00 0.00 C

ATOM 3568 HG2 PRO 224 59.162 56.047 53.321 1.00 0.00 H

ATOM 3569 HG3 PRO 224 60.408 56.715 52.275 1.00 0.00 H

ATOM 3570 CB PRO 224 60.166 57.918 54.143 1.00 0.00 C

ATOM 3571 HB2 PRO 224 59.334 58.160 54.805 1.00 0.00 H

ATOM 3572 HB3 PRO 224 60.354 58.737 53.449 1.00 0.00 H

ATOM 3573 CA PRO 224 61.360 57.843 55.137 1.00 0.00 C

ATOM 3574 HA PRO 224 62.012 58.708 55.015 1.00 0.00 H

ATOM 3575 C PRO 224 60.747 57.739 56.623 1.00 0.00 C

ATOM 3576 O PRO 224 60.343 56.669 57.093 1.00 0.00 O

ATOM 3577 N HIE 225 60.559 58.892 57.310 1.00 0.00 N

ATOM 3578 H HIE 225 61.028 59.712 56.953 1.00 0.00 H

ATOM 3579 CA HIE 225 60.020 59.013 58.613 1.00 0.00 C

ATOM 3580 HA HIE 225 60.394 58.211 59.250 1.00 0.00 H

ATOM 3581 CB HIE 225 60.531 60.263 59.391 1.00 0.00 C

ATOM 3582 HB2 HIE 225 61.544 60.455 59.039 1.00 0.00 H

ATOM 3583 HB3 HIE 225 59.990 61.130 59.012 1.00 0.00 H

ATOM 3584 CG HIE 225 60.354 60.118 60.902 1.00 0.00 C

ATOM 3585 ND1 HIE 225 61.266 59.552 61.724 1.00 0.00 N

ATOM 3586 CE1 HIE 225 60.718 59.741 62.920 1.00 0.00 C

ATOM 3587 HE1 HIE 225 61.095 59.369 63.861 1.00 0.00 H

ATOM 3588 NE2 HIE 225 59.659 60.504 62.883 1.00 0.00 N

ATOM 3589 HE2 HIE 225 59.151 60.894 63.664 1.00 0.00 H

ATOM 3590 CD2 HIE 225 59.322 60.620 61.584 1.00 0.00 C

ATOM 3591 HD2 HIE 225 58.473 61.203 61.260 1.00 0.00 H

ATOM 3592 C HIE 225 58.514 58.915 58.579 1.00 0.00 C

ATOM 3593 O HIE 225 57.844 59.890 58.838 1.00 0.00 O

ATOM 3594 N ARG 226 57.952 57.742 58.191 1.00 0.00 N

ATOM 3595 H ARG 226 58.544 56.970 57.921 1.00 0.00 H

ATOM 3596 CA ARG 226 56.565 57.731 57.827 1.00 0.00 C

ATOM 3597 HA ARG 226 56.283 58.445 57.054 1.00 0.00 H

ATOM 3598 CB ARG 226 56.291 56.366 57.166 1.00 0.00 C

ATOM 3599 HB2 ARG 226 56.553 55.570 57.862 1.00 0.00 H

ATOM 3600 HB3 ARG 226 55.248 56.321 56.852 1.00 0.00 H

ATOM 3601 CG ARG 226 57.174 56.064 55.967 1.00 0.00 C

ATOM 3602 HG2 ARG 226 57.356 57.015 55.467 1.00 0.00 H

ATOM 3603 HG3 ARG 226 58.119 55.627 56.290 1.00 0.00 H

ATOM 3604 CD ARG 226 56.496 55.050 55.026 1.00 0.00 C

ATOM 3605 HD2 ARG 226 57.142 54.666 54.237 1.00 0.00 H

ATOM 3606 HD3 ARG 226 56.100 54.257 55.660 1.00 0.00 H

ATOM 3607 NE ARG 226 55.321 55.502 54.358 1.00 0.00 N

ATOM 3608 HE ARG 226 55.239 56.508 54.314 1.00 0.00 H

ATOM 3609 CZ ARG 226 54.544 54.730 53.636 1.00 0.00 C

ATOM 3610 NH1 ARG 226 53.511 55.248 53.069 1.00 0.00 N

ATOM 3611 HH11 ARG 226 53.137 56.167 53.259 1.00 0.00 H

ATOM 3612 HH12 ARG 226 52.902 54.656 52.521 1.00 0.00 H

ATOM 3613 NH2 ARG 226 54.706 53.459 53.452 1.00 0.00 N

ATOM 3614 HH21 ARG 226 55.510 52.978 53.829 1.00 0.00 H

ATOM 3615 HH22 ARG 226 53.983 52.911 53.008 1.00 0.00 H

ATOM 3616 C ARG 226 55.589 57.928 59.019 1.00 0.00 C

ATOM 3617 O ARG 226 54.406 58.282 58.845 1.00 0.00 O

ATOM 3618 N VAL 227 56.039 57.732 60.271 1.00 0.00 N

ATOM 3619 H VAL 227 56.936 57.275 60.346 1.00 0.00 H

ATOM 3620 CA VAL 227 55.359 57.943 61.557 1.00 0.00 C

ATOM 3621 HA VAL 227 54.362 57.517 61.439 1.00 0.00 H

ATOM 3622 CB VAL 227 56.052 57.276 62.733 1.00 0.00 C

ATOM 3623 HB VAL 227 55.417 57.330 63.617 1.00 0.00 H

ATOM 3624 CG1 VAL 227 56.214 55.814 62.573 1.00 0.00 C

ATOM 3625 HG11 VAL 227 55.214 55.384 62.512 1.00 0.00 H

ATOM 3626 HG12 VAL 227 56.739 55.421 61.703 1.00 0.00 H

ATOM 3627 HG13 VAL 227 56.650 55.318 63.440 1.00 0.00 H

ATOM 3628 CG2 VAL 227 57.451 57.862 63.120 1.00 0.00 C

ATOM 3629 HG21 VAL 227 57.774 57.421 64.063 1.00 0.00 H

ATOM 3630 HG22 VAL 227 58.209 57.597 62.383 1.00 0.00 H

ATOM 3631 HG23 VAL 227 57.207 58.907 63.311 1.00 0.00 H

ATOM 3632 C VAL 227 55.076 59.410 61.830 1.00 0.00 C

ATOM 3633 O VAL 227 54.401 59.759 62.754 1.00 0.00 O

ATOM 3634 N ASP 228 55.643 60.349 61.023 1.00 0.00 N

ATOM 3635 H ASP 228 56.088 59.869 60.254 1.00 0.00 H

ATOM 3636 CA ASP 228 55.134 61.716 60.953 1.00 0.00 C

ATOM 3637 HA ASP 228 54.140 61.675 61.400 1.00 0.00 H

ATOM 3638 CB ASP 228 55.965 62.752 61.730 1.00 0.00 C

ATOM 3639 HB2 ASP 228 56.271 62.415 62.720 1.00 0.00 H

ATOM 3640 HB3 ASP 228 56.874 63.000 61.182 1.00 0.00 H

ATOM 3641 CG ASP 228 55.144 64.111 61.852 1.00 0.00 C

ATOM 3642 OD1 ASP 228 54.148 64.134 62.589 1.00 0.00 O

ATOM 3643 OD2 ASP 228 55.513 65.132 61.251 1.00 0.00 O

ATOM 3644 C ASP 228 54.863 62.215 59.530 1.00 0.00 C

ATOM 3645 O ASP 228 54.000 63.104 59.353 1.00 0.00 O

ATOM 3646 N LEU 229 55.538 61.767 58.519 1.00 0.00 N

ATOM 3647 H LEU 229 56.252 61.054 58.551 1.00 0.00 H

ATOM 3648 CA LEU 229 55.458 62.502 57.185 1.00 0.00 C

ATOM 3649 HA LEU 229 55.246 63.561 57.327 1.00 0.00 H

ATOM 3650 CB LEU 229 56.759 62.438 56.401 1.00 0.00 C

ATOM 3651 HB2 LEU 229 57.076 61.395 56.436 1.00 0.00 H

ATOM 3652 HB3 LEU 229 56.628 62.606 55.332 1.00 0.00 H

ATOM 3653 CG LEU 229 57.883 63.371 56.901 1.00 0.00 C

ATOM 3654 HG LEU 229 57.918 63.460 57.987 1.00 0.00 H

ATOM 3655 CD1 LEU 229 59.300 62.942 56.544 1.00 0.00 C

ATOM 3656 HD11 LEU 229 59.443 61.901 56.832 1.00 0.00 H

ATOM 3657 HD12 LEU 229 59.566 63.108 55.499 1.00 0.00 H

ATOM 3658 HD13 LEU 229 60.045 63.484 57.126 1.00 0.00 H

ATOM 3659 CD2 LEU 229 57.665 64.796 56.228 1.00 0.00 C

ATOM 3660 HD21 LEU 229 56.783 65.326 56.589 1.00 0.00 H

ATOM 3661 HD22 LEU 229 58.548 65.418 56.374 1.00 0.00 H

ATOM 3662 HD23 LEU 229 57.563 64.654 55.152 1.00 0.00 H

ATOM 3663 C LEU 229 54.309 61.983 56.335 1.00 0.00 C

ATOM 3664 O LEU 229 53.747 62.784 55.540 1.00 0.00 O

ATOM 3665 N ASP 230 53.837 60.777 56.552 1.00 0.00 N

ATOM 3666 H ASP 230 54.307 60.144 57.183 1.00 0.00 H

ATOM 3667 CA ASP 230 52.628 60.364 55.828 1.00 0.00 C

ATOM 3668 HA ASP 230 52.710 60.702 54.796 1.00 0.00 H

ATOM 3669 CB ASP 230 52.535 58.813 55.887 1.00 0.00 C

ATOM 3670 HB2 ASP 230 53.059 58.453 56.773 1.00 0.00 H

ATOM 3671 HB3 ASP 230 51.521 58.412 55.900 1.00 0.00 H

ATOM 3672 CG ASP 230 53.244 58.137 54.688 1.00 0.00 C

ATOM 3673 OD1 ASP 230 54.474 58.154 54.738 1.00 0.00 O

ATOM 3674 OD2 ASP 230 52.567 57.726 53.727 1.00 0.00 O

ATOM 3675 C ASP 230 51.382 61.035 56.536 1.00 0.00 C

ATOM 3676 O ASP 230 50.411 61.395 55.914 1.00 0.00 O

ATOM 3677 N ILE 231 51.445 61.247 57.804 1.00 0.00 N

ATOM 3678 H ILE 231 52.176 60.806 58.344 1.00 0.00 H

ATOM 3679 CA ILE 231 50.557 62.106 58.606 1.00 0.00 C

ATOM 3680 HA ILE 231 49.573 61.645 58.519 1.00 0.00 H

ATOM 3681 CB ILE 231 50.954 62.219 60.093 1.00 0.00 C

ATOM 3682 HB ILE 231 51.831 62.844 60.262 1.00 0.00 H

ATOM 3683 CG2 ILE 231 49.831 62.947 60.922 1.00 0.00 C

ATOM 3684 HG21 ILE 231 49.962 63.046 61.999 1.00 0.00 H

ATOM 3685 HG22 ILE 231 49.775 63.979 60.577 1.00 0.00 H

ATOM 3686 HG23 ILE 231 48.894 62.457 60.654 1.00 0.00 H

ATOM 3687 CG1 ILE 231 51.345 60.878 60.702 1.00 0.00 C

ATOM 3688 HG12 ILE 231 50.620 60.114 60.421 1.00 0.00 H

ATOM 3689 HG13 ILE 231 52.291 60.650 60.211 1.00 0.00 H

ATOM 3690 CD1 ILE 231 51.539 60.808 62.240 1.00 0.00 C

ATOM 3691 HD11 ILE 231 52.274 61.583 62.459 1.00 0.00 H

ATOM 3692 HD12 ILE 231 50.577 61.011 62.711 1.00 0.00 H

ATOM 3693 HD13 ILE 231 51.951 59.841 62.526 1.00 0.00 H

ATOM 3694 C ILE 231 50.494 63.474 58.014 1.00 0.00 C

ATOM 3695 O ILE 231 49.321 63.852 57.779 1.00 0.00 O

ATOM 3696 N LEU 232 51.644 64.173 57.734 1.00 0.00 N

ATOM 3697 H LEU 232 52.461 63.766 58.166 1.00 0.00 H

ATOM 3698 CA LEU 232 51.603 65.392 56.855 1.00 0.00 C

ATOM 3699 HA LEU 232 50.990 66.138 57.361 1.00 0.00 H

ATOM 3700 CB LEU 232 53.021 65.961 56.770 1.00 0.00 C

ATOM 3701 HB2 LEU 232 53.419 66.216 57.752 1.00 0.00 H

ATOM 3702 HB3 LEU 232 53.712 65.239 56.335 1.00 0.00 H

ATOM 3703 CG LEU 232 53.098 67.316 56.013 1.00 0.00 C

ATOM 3704 HG LEU 232 52.810 67.094 54.985 1.00 0.00 H

ATOM 3705 CD1 LEU 232 52.247 68.395 56.606 1.00 0.00 C

ATOM 3706 HD11 LEU 232 52.385 69.340 56.081 1.00 0.00 H

ATOM 3707 HD12 LEU 232 51.187 68.157 56.515 1.00 0.00 H

ATOM 3708 HD13 LEU 232 52.511 68.520 57.656 1.00 0.00 H

ATOM 3709 CD2 LEU 232 54.578 67.722 55.875 1.00 0.00 C

ATOM 3710 HD21 LEU 232 55.147 66.949 55.359 1.00 0.00 H

ATOM 3711 HD22 LEU 232 54.763 68.738 55.524 1.00 0.00 H

ATOM 3712 HD23 LEU 232 54.934 67.799 56.903 1.00 0.00 H

ATOM 3713 C LEU 232 50.951 65.118 55.481 1.00 0.00 C

ATOM 3714 O LEU 232 50.121 65.817 54.955 1.00 0.00 O

ATOM 3715 N THR 233 51.214 63.929 54.846 1.00 0.00 N

ATOM 3716 H THR 233 51.817 63.303 55.361 1.00 0.00 H

ATOM 3717 CA THR 233 50.690 63.625 53.526 1.00 0.00 C

ATOM 3718 HA THR 233 50.956 64.510 52.948 1.00 0.00 H

ATOM 3719 CB THR 233 51.350 62.387 52.984 1.00 0.00 C

ATOM 3720 HB THR 233 51.217 61.551 53.670 1.00 0.00 H

ATOM 3721 CG2 THR 233 50.980 61.883 51.546 1.00 0.00 C

ATOM 3722 HG21 THR 233 51.352 60.864 51.433 1.00 0.00 H

ATOM 3723 HG22 THR 233 49.933 61.809 51.251 1.00 0.00 H

ATOM 3724 HG23 THR 233 51.507 62.518 50.836 1.00 0.00 H

ATOM 3725 OG1 THR 233 52.759 62.561 52.959 1.00 0.00 O

ATOM 3726 HG1 THR 233 53.038 62.536 53.877 1.00 0.00 H

ATOM 3727 C THR 233 49.220 63.352 53.415 1.00 0.00 C

ATOM 3728 O THR 233 48.558 63.747 52.454 1.00 0.00 O

ATOM 3729 N PHE 234 48.587 62.706 54.379 1.00 0.00 N

ATOM 3730 H PHE 234 49.143 62.218 55.067 1.00 0.00 H

ATOM 3731 CA PHE 234 47.199 62.454 54.430 1.00 0.00 C

ATOM 3732 HA PHE 234 46.843 61.879 53.576 1.00 0.00 H

ATOM 3733 CB PHE 234 46.858 61.602 55.664 1.00 0.00 C

ATOM 3734 HB2 PHE 234 47.538 60.763 55.814 1.00 0.00 H

ATOM 3735 HB3 PHE 234 46.992 62.166 56.587 1.00 0.00 H

ATOM 3736 CG PHE 234 45.424 61.008 55.616 1.00 0.00 C

ATOM 3737 CD1 PHE 234 44.322 61.655 56.186 1.00 0.00 C

ATOM 3738 HD1 PHE 234 44.465 62.645 56.594 1.00 0.00 H

ATOM 3739 CE1 PHE 234 43.037 61.110 56.076 1.00 0.00 C

ATOM 3740 HE1 PHE 234 42.248 61.625 56.606 1.00 0.00 H

ATOM 3741 CZ PHE 234 42.886 59.880 55.443 1.00 0.00 C

ATOM 3742 HZ PHE 234 41.867 59.527 55.388 1.00 0.00 H

ATOM 3743 CE2 PHE 234 43.969 59.202 54.884 1.00 0.00 C

ATOM 3744 HE2 PHE 234 43.793 58.245 54.415 1.00 0.00 H

ATOM 3745 CD2 PHE 234 45.225 59.783 54.949 1.00 0.00 C

ATOM 3746 HD2 PHE 234 46.007 59.254 54.424 1.00 0.00 H

ATOM 3747 C PHE 234 46.429 63.856 54.554 1.00 0.00 C

ATOM 3748 O PHE 234 45.181 63.904 54.350 1.00 0.00 O

ATOM 3749 N THR 235 47.128 64.974 54.910 1.00 0.00 N

ATOM 3750 H THR 235 48.135 64.965 54.835 1.00 0.00 H

ATOM 3751 CA THR 235 46.588 66.267 55.317 1.00 0.00 C

ATOM 3752 HA THR 235 45.556 66.314 54.969 1.00 0.00 H

ATOM 3753 CB THR 235 46.594 66.447 56.830 1.00 0.00 C

ATOM 3754 HB THR 235 46.126 67.362 57.193 1.00 0.00 H

ATOM 3755 CG2 THR 235 45.942 65.400 57.657 1.00 0.00 C

ATOM 3756 HG21 THR 235 46.095 65.712 58.690 1.00 0.00 H

ATOM 3757 HG22 THR 235 44.870 65.442 57.463 1.00 0.00 H

ATOM 3758 HG23 THR 235 46.440 64.443 57.503 1.00 0.00 H

ATOM 3759 OG1 THR 235 47.985 66.422 57.187 1.00 0.00 O

ATOM 3760 HG1 THR 235 48.239 65.506 57.051 1.00 0.00 H

ATOM 3761 C THR 235 47.049 67.516 54.579 1.00 0.00 C

ATOM 3762 O THR 235 46.536 68.572 54.851 1.00 0.00 O

ATOM 3763 N ILE 236 47.895 67.418 53.561 1.00 0.00 N

ATOM 3764 H ILE 236 48.210 66.486 53.329 1.00 0.00 H

ATOM 3765 CA ILE 236 48.423 68.563 52.760 1.00 0.00 C

ATOM 3766 HA ILE 236 48.315 69.362 53.494 1.00 0.00 H

ATOM 3767 CB ILE 236 49.905 68.360 52.491 1.00 0.00 C

ATOM 3768 HB ILE 236 50.307 68.144 53.481 1.00 0.00 H

ATOM 3769 CG2 ILE 236 50.114 67.311 51.519 1.00 0.00 C

ATOM 3770 HG21 ILE 236 51.113 66.879 51.564 1.00 0.00 H

ATOM 3771 HG22 ILE 236 49.449 66.455 51.638 1.00 0.00 H

ATOM 3772 HG23 ILE 236 49.831 67.717 50.548 1.00 0.00 H

ATOM 3773 CG1 ILE 236 50.620 69.638 52.102 1.00 0.00 C

ATOM 3774 HG12 ILE 236 50.214 69.998 51.156 1.00 0.00 H

ATOM 3775 HG13 ILE 236 50.407 70.482 52.758 1.00 0.00 H

ATOM 3776 CD1 ILE 236 52.160 69.451 52.129 1.00 0.00 C

ATOM 3777 HD11 ILE 236 52.417 69.376 53.186 1.00 0.00 H

ATOM 3778 HD12 ILE 236 52.489 68.524 51.659 1.00 0.00 H

ATOM 3779 HD13 ILE 236 52.628 70.341 51.708 1.00 0.00 H

ATOM 3780 C ILE 236 47.568 69.054 51.601 1.00 0.00 C

ATOM 3781 O ILE 236 47.965 69.983 50.865 1.00 0.00 O

ATOM 3782 N ALA 237 46.475 68.346 51.313 1.00 0.00 N

ATOM 3783 H ALA 237 46.447 67.470 51.815 1.00 0.00 H

ATOM 3784 CA ALA 237 45.740 68.413 50.059 1.00 0.00 C

ATOM 3785 HA ALA 237 46.422 68.193 49.237 1.00 0.00 H

ATOM 3786 CB ALA 237 44.606 67.395 50.057 1.00 0.00 C

ATOM 3787 HB1 ALA 237 43.991 67.484 49.162 1.00 0.00 H

ATOM 3788 HB2 ALA 237 44.989 66.374 50.074 1.00 0.00 H

ATOM 3789 HB3 ALA 237 43.911 67.681 50.847 1.00 0.00 H

ATOM 3790 C ALA 237 45.204 69.773 49.733 1.00 0.00 C

ATOM 3791 O ALA 237 45.348 70.265 48.619 1.00 0.00 O

ATOM 3792 N LEU 238 44.681 70.554 50.749 1.00 0.00 N

ATOM 3793 H LEU 238 44.661 70.092 51.647 1.00 0.00 H

ATOM 3794 CA LEU 238 44.115 71.917 50.618 1.00 0.00 C

ATOM 3795 HA LEU 238 43.628 72.001 49.646 1.00 0.00 H

ATOM 3796 CB LEU 238 43.090 72.156 51.722 1.00 0.00 C

ATOM 3797 HB2 LEU 238 43.682 72.388 52.607 1.00 0.00 H

ATOM 3798 HB3 LEU 238 42.584 73.104 51.536 1.00 0.00 H

ATOM 3799 CG LEU 238 42.021 71.119 52.058 1.00 0.00 C

ATOM 3800 HG LEU 238 42.381 70.109 52.252 1.00 0.00 H

ATOM 3801 CD1 LEU 238 41.246 71.540 53.265 1.00 0.00 C

ATOM 3802 HD11 LEU 238 41.773 71.891 54.152 1.00 0.00 H

ATOM 3803 HD12 LEU 238 40.573 72.367 53.039 1.00 0.00 H

ATOM 3804 HD13 LEU 238 40.521 70.761 53.501 1.00 0.00 H

ATOM 3805 CD2 LEU 238 41.023 71.041 50.907 1.00 0.00 C

ATOM 3806 HD21 LEU 238 41.554 70.857 49.973 1.00 0.00 H

ATOM 3807 HD22 LEU 238 40.398 70.156 51.022 1.00 0.00 H

ATOM 3808 HD23 LEU 238 40.376 71.917 50.852 1.00 0.00 H

ATOM 3809 C LEU 238 45.160 73.028 50.536 1.00 0.00 C

ATOM 3810 O LEU 238 44.808 74.138 50.041 1.00 0.00 O

ATOM 3811 N THR 239 46.404 72.854 50.966 1.00 0.00 N

ATOM 3812 H THR 239 46.538 71.900 51.270 1.00 0.00 H

ATOM 3813 CA THR 239 47.573 73.766 51.091 1.00 0.00 C

ATOM 3814 HA THR 239 47.165 74.775 51.032 1.00 0.00 H

ATOM 3815 CB THR 239 48.242 73.730 52.520 1.00 0.00 C

ATOM 3816 HB THR 239 49.185 74.272 52.447 1.00 0.00 H

ATOM 3817 CG2 THR 239 47.309 74.406 53.642 1.00 0.00 C

ATOM 3818 HG21 THR 239 47.925 74.174 54.511 1.00 0.00 H

ATOM 3819 HG22 THR 239 47.291 75.493 53.560 1.00 0.00 H

ATOM 3820 HG23 THR 239 46.374 73.849 53.691 1.00 0.00 H

ATOM 3821 OG1 THR 239 48.567 72.456 53.031 1.00 0.00 O

ATOM 3822 HG1 THR 239 47.819 71.871 52.890 1.00 0.00 H

ATOM 3823 C THR 239 48.694 73.595 50.019 1.00 0.00 C

ATOM 3824 O THR 239 49.624 74.423 49.905 1.00 0.00 O

ATOM 3825 N ALA 240 48.471 72.557 49.177 1.00 0.00 N

ATOM 3826 H ALA 240 47.708 71.918 49.352 1.00 0.00 H

ATOM 3827 CA ALA 240 49.414 72.187 48.124 1.00 0.00 C

ATOM 3828 HA ALA 240 50.416 72.315 48.533 1.00 0.00 H

ATOM 3829 CB ALA 240 49.073 70.782 47.801 1.00 0.00 C

ATOM 3830 HB1 ALA 240 49.730 70.416 47.011 1.00 0.00 H

ATOM 3831 HB2 ALA 240 49.219 70.177 48.695 1.00 0.00 H

ATOM 3832 HB3 ALA 240 48.054 70.697 47.422 1.00 0.00 H

ATOM 3833 C ALA 240 49.499 73.073 46.823 1.00 0.00 C

ATOM 3834 O ALA 240 50.614 73.137 46.264 1.00 0.00 O

ATOM 3835 N SER 241 48.438 73.772 46.432 1.00 0.00 N

ATOM 3836 H SER 241 47.553 73.727 46.916 1.00 0.00 H

ATOM 3837 CA SER 241 48.389 74.488 45.126 1.00 0.00 C

ATOM 3838 HA SER 241 48.680 73.806 44.327 1.00 0.00 H

ATOM 3839 CB SER 241 46.960 74.897 44.825 1.00 0.00 C

ATOM 3840 HB2 SER 241 46.481 75.326 45.704 1.00 0.00 H

ATOM 3841 HB3 SER 241 47.137 75.593 44.005 1.00 0.00 H

ATOM 3842 OG SER 241 46.257 73.714 44.498 1.00 0.00 O

ATOM 3843 HG SER 241 46.111 73.248 45.324 1.00 0.00 H

ATOM 3844 C SER 241 49.320 75.726 45.188 1.00 0.00 C

ATOM 3845 O SER 241 49.942 76.079 44.228 1.00 0.00 O

ATOM 3846 N GLU 242 49.394 76.373 46.401 1.00 0.00 N

ATOM 3847 H GLU 242 48.799 76.051 47.151 1.00 0.00 H

ATOM 3848 CA GLU 242 50.019 77.686 46.608 1.00 0.00 C

ATOM 3849 HA GLU 242 49.908 78.316 45.725 1.00 0.00 H

ATOM 3850 CB GLU 242 49.254 78.404 47.708 1.00 0.00 C

ATOM 3851 HB2 GLU 242 49.280 77.743 48.574 1.00 0.00 H

ATOM 3852 HB3 GLU 242 49.772 79.343 47.901 1.00 0.00 H

ATOM 3853 CG GLU 242 47.811 78.666 47.356 1.00 0.00 C

ATOM 3854 HG2 GLU 242 47.764 79.521 46.682 1.00 0.00 H

ATOM 3855 HG3 GLU 242 47.484 77.782 46.809 1.00 0.00 H

ATOM 3856 CD GLU 242 46.989 78.896 48.632 1.00 0.00 C

ATOM 3857 OE1 GLU 242 46.572 77.856 49.137 1.00 0.00 O

ATOM 3858 OE2 GLU 242 46.859 80.120 49.039 1.00 0.00 O

ATOM 3859 C GLU 242 51.485 77.604 46.937 1.00 0.00 C

ATOM 3860 O GLU 242 52.208 78.604 47.018 1.00 0.00 O

ATOM 3861 N VAL 243 52.056 76.414 47.101 1.00 0.00 N

ATOM 3862 H VAL 243 51.374 75.689 47.270 1.00 0.00 H

ATOM 3863 CA VAL 243 53.562 76.231 47.383 1.00 0.00 C

ATOM 3864 HA VAL 243 53.809 76.919 48.191 1.00 0.00 H

ATOM 3865 CB VAL 243 53.811 74.815 47.915 1.00 0.00 C

ATOM 3866 HB VAL 243 53.785 74.072 47.117 1.00 0.00 H

ATOM 3867 CG1 VAL 243 55.178 74.666 48.593 1.00 0.00 C

ATOM 3868 HG11 VAL 243 55.339 73.626 48.878 1.00 0.00 H

ATOM 3869 HG12 VAL 243 55.993 74.973 47.937 1.00 0.00 H

ATOM 3870 HG13 VAL 243 55.161 75.231 49.526 1.00 0.00 H

ATOM 3871 CG2 VAL 243 52.763 74.220 48.843 1.00 0.00 C

ATOM 3872 HG21 VAL 243 52.801 74.798 49.766 1.00 0.00 H

ATOM 3873 HG22 VAL 243 51.790 74.196 48.352 1.00 0.00 H

ATOM 3874 HG23 VAL 243 53.000 73.221 49.210 1.00 0.00 H

ATOM 3875 C VAL 243 54.484 76.563 46.196 1.00 0.00 C

ATOM 3876 O VAL 243 55.553 77.140 46.362 1.00 0.00 O

ATOM 3877 N ILE 244 54.100 76.237 44.950 1.00 0.00 N

ATOM 3878 H ILE 244 53.338 75.575 44.934 1.00 0.00 H

ATOM 3879 CA ILE 244 54.954 76.509 43.754 1.00 0.00 C

ATOM 3880 HA ILE 244 55.980 76.245 44.008 1.00 0.00 H

ATOM 3881 CB ILE 244 54.555 75.690 42.524 1.00 0.00 C

ATOM 3882 HB ILE 244 54.643 74.669 42.894 1.00 0.00 H

ATOM 3883 CG2 ILE 244 53.121 75.895 42.069 1.00 0.00 C

ATOM 3884 HG21 ILE 244 53.032 76.971 41.918 1.00 0.00 H

ATOM 3885 HG22 ILE 244 52.929 75.269 41.198 1.00 0.00 H

ATOM 3886 HG23 ILE 244 52.453 75.650 42.894 1.00 0.00 H

ATOM 3887 CG1 ILE 244 55.554 75.859 41.303 1.00 0.00 C

ATOM 3888 HG12 ILE 244 55.249 76.725 40.715 1.00 0.00 H

ATOM 3889 HG13 ILE 244 56.588 76.044 41.597 1.00 0.00 H

ATOM 3890 CD1 ILE 244 55.571 74.725 40.274 1.00 0.00 C

ATOM 3891 HD11 ILE 244 56.490 74.144 40.354 1.00 0.00 H

ATOM 3892 HD12 ILE 244 54.683 74.094 40.229 1.00 0.00 H

ATOM 3893 HD13 ILE 244 55.576 75.130 39.262 1.00 0.00 H

ATOM 3894 C ILE 244 54.823 78.046 43.441 1.00 0.00 C

ATOM 3895 O ILE 244 55.776 78.582 42.823 1.00 0.00 O

ATOM 3896 N ASN 245 53.709 78.664 43.766 1.00 0.00 N

ATOM 3897 H ASN 245 52.948 78.075 44.074 1.00 0.00 H

ATOM 3898 CA ASN 245 53.436 80.016 43.339 1.00 0.00 C

ATOM 3899 HA ASN 245 53.307 79.909 42.262 1.00 0.00 H

ATOM 3900 CB ASN 245 52.264 80.523 44.079 1.00 0.00 C

ATOM 3901 HB2 ASN 245 51.550 79.706 43.977 1.00 0.00 H

ATOM 3902 HB3 ASN 245 52.479 80.748 45.124 1.00 0.00 H

ATOM 3903 CG ASN 245 51.593 81.654 43.315 1.00 0.00 C

ATOM 3904 OD1 ASN 245 50.830 81.525 42.371 1.00 0.00 O

ATOM 3905 ND2 ASN 245 51.783 82.847 43.833 1.00 0.00 N

ATOM 3906 HD21 ASN 245 51.198 83.624 43.561 1.00 0.00 H

ATOM 3907 HD22 ASN 245 52.347 82.881 44.670 1.00 0.00 H

ATOM 3908 C ASN 245 54.675 81.028 43.428 1.00 0.00 C

ATOM 3909 O ASN 245 55.099 81.545 42.380 1.00 0.00 O

ATOM 3910 N PRO 246 55.255 81.240 44.582 1.00 0.00 N

ATOM 3911 CD PRO 246 55.130 80.502 45.868 1.00 0.00 C

ATOM 3912 HD2 PRO 246 55.170 79.417 45.775 1.00 0.00 H

ATOM 3913 HD3 PRO 246 54.246 80.858 46.396 1.00 0.00 H

ATOM 3914 CG PRO 246 56.425 80.878 46.627 1.00 0.00 C

ATOM 3915 HG2 PRO 246 57.230 80.222 46.298 1.00 0.00 H

ATOM 3916 HG3 PRO 246 56.226 80.877 47.699 1.00 0.00 H

ATOM 3917 CB PRO 246 56.680 82.294 46.149 1.00 0.00 C

ATOM 3918 HB2 PRO 246 57.695 82.611 46.389 1.00 0.00 H

ATOM 3919 HB3 PRO 246 55.964 82.959 46.632 1.00 0.00 H

ATOM 3920 CA PRO 246 56.458 82.095 44.711 1.00 0.00 C

ATOM 3921 HA PRO 246 56.242 83.098 44.343 1.00 0.00 H

ATOM 3922 C PRO 246 57.701 81.631 43.879 1.00 0.00 C

ATOM 3923 O PRO 246 58.575 82.436 43.686 1.00 0.00 O

ATOM 3924 N LEU 247 57.732 80.398 43.332 1.00 0.00 N

ATOM 3925 H LEU 247 57.005 79.712 43.476 1.00 0.00 H

ATOM 3926 CA LEU 247 58.742 79.888 42.323 1.00 0.00 C

ATOM 3927 HA LEU 247 59.720 80.321 42.537 1.00 0.00 H

ATOM 3928 CB LEU 247 58.948 78.391 42.393 1.00 0.00 C

ATOM 3929 HB2 LEU 247 58.084 77.888 41.960 1.00 0.00 H

ATOM 3930 HB3 LEU 247 59.865 78.213 41.831 1.00 0.00 H

ATOM 3931 CG LEU 247 59.112 77.779 43.788 1.00 0.00 C

ATOM 3932 HG LEU 247 58.189 78.029 44.309 1.00 0.00 H

ATOM 3933 CD1 LEU 247 59.235 76.226 43.710 1.00 0.00 C

ATOM 3934 HD11 LEU 247 60.120 75.979 43.123 1.00 0.00 H

ATOM 3935 HD12 LEU 247 59.365 75.814 44.711 1.00 0.00 H

ATOM 3936 HD13 LEU 247 58.406 75.755 43.183 1.00 0.00 H

ATOM 3937 CD2 LEU 247 60.260 78.385 44.502 1.00 0.00 C

ATOM 3938 HD21 LEU 247 60.371 78.082 45.543 1.00 0.00 H

ATOM 3939 HD22 LEU 247 61.157 78.001 44.016 1.00 0.00 H

ATOM 3940 HD23 LEU 247 60.261 79.475 44.510 1.00 0.00 H

ATOM 3941 C LEU 247 58.417 80.242 40.800 1.00 0.00 C

ATOM 3942 O LEU 247 59.191 80.126 39.925 1.00 0.00 O

ATOM 3943 N ILE 248 57.174 80.538 40.432 1.00 0.00 N

ATOM 3944 H ILE 248 56.508 80.285 41.147 1.00 0.00 H

ATOM 3945 CA ILE 248 56.613 80.833 39.119 1.00 0.00 C

ATOM 3946 HA ILE 248 57.458 80.932 38.437 1.00 0.00 H

ATOM 3947 CB ILE 248 55.626 79.723 38.713 1.00 0.00 C

ATOM 3948 HB ILE 248 55.239 79.931 37.715 1.00 0.00 H

ATOM 3949 CG2 ILE 248 56.281 78.315 38.503 1.00 0.00 C

ATOM 3950 HG21 ILE 248 56.929 78.346 37.626 1.00 0.00 H

ATOM 3951 HG22 ILE 248 56.838 77.949 39.365 1.00 0.00 H

ATOM 3952 HG23 ILE 248 55.469 77.589 38.538 1.00 0.00 H

ATOM 3953 CG1 ILE 248 54.332 79.711 39.569 1.00 0.00 C

ATOM 3954 HG12 ILE 248 54.602 79.402 40.579 1.00 0.00 H

ATOM 3955 HG13 ILE 248 53.974 80.733 39.451 1.00 0.00 H

ATOM 3956 CD1 ILE 248 53.092 78.903 39.085 1.00 0.00 C

ATOM 3957 HD11 ILE 248 52.769 79.333 38.137 1.00 0.00 H

ATOM 3958 HD12 ILE 248 53.231 77.842 38.882 1.00 0.00 H

ATOM 3959 HD13 ILE 248 52.352 79.015 39.878 1.00 0.00 H

ATOM 3960 C ILE 248 55.821 82.213 38.914 1.00 0.00 C

ATOM 3961 O ILE 248 55.565 82.569 37.747 1.00 0.00 O

ATOM 3962 N GLU 249 55.580 82.972 39.964 1.00 0.00 N

ATOM 3963 H GLU 249 55.785 82.575 40.869 1.00 0.00 H

ATOM 3964 CA GLU 249 54.986 84.274 39.960 1.00 0.00 C

ATOM 3965 HA GLU 249 54.815 84.639 38.948 1.00 0.00 H

ATOM 3966 CB GLU 249 53.619 84.174 40.708 1.00 0.00 C

ATOM 3967 HB2 GLU 249 53.707 83.454 41.522 1.00 0.00 H

ATOM 3968 HB3 GLU 249 53.332 85.126 41.154 1.00 0.00 H

ATOM 3969 CG GLU 249 52.587 83.733 39.721 1.00 0.00 C

ATOM 3970 HG2 GLU 249 52.983 82.935 39.093 1.00 0.00 H

ATOM 3971 HG3 GLU 249 51.674 83.363 40.187 1.00 0.00 H

ATOM 3972 CD GLU 249 52.279 84.776 38.716 1.00 0.00 C

ATOM 3973 OE1 GLU 249 51.824 85.944 39.061 1.00 0.00 O

ATOM 3974 OE2 GLU 249 52.391 84.584 37.489 1.00 0.00 O

ATOM 3975 C GLU 249 55.787 85.487 40.518 1.00 0.00 C

ATOM 3976 O GLU 249 55.298 86.643 40.484 1.00 0.00 O

ATOM 3977 N GLU 250 57.067 85.338 40.923 1.00 0.00 N

ATOM 3978 H GLU 250 57.493 84.426 40.838 1.00 0.00 H

ATOM 3979 CA GLU 250 57.969 86.355 41.518 1.00 0.00 C

ATOM 3980 HA GLU 250 57.531 87.351 41.452 1.00 0.00 H

ATOM 3981 CB GLU 250 58.212 86.138 43.037 1.00 0.00 C

ATOM 3982 HB2 GLU 250 58.684 85.156 43.065 1.00 0.00 H

ATOM 3983 HB3 GLU 250 58.911 86.869 43.445 1.00 0.00 H

ATOM 3984 CG GLU 250 56.978 86.226 43.886 1.00 0.00 C

ATOM 3985 HG2 GLU 250 56.488 87.169 43.643 1.00 0.00 H

ATOM 3986 HG3 GLU 250 56.258 85.498 43.512 1.00 0.00 H

ATOM 3987 CD GLU 250 57.102 86.015 45.363 1.00 0.00 C

ATOM 3988 OE1 GLU 250 56.067 86.135 46.091 1.00 0.00 O

ATOM 3989 OE2 GLU 250 58.202 85.780 45.884 1.00 0.00 O

ATOM 3990 C GLU 250 59.314 86.464 40.857 1.00 0.00 C

ATOM 3991 O GLU 250 59.670 87.595 40.423 1.00 0.00 O

ATOM 3992 OXT GLU 250 59.964 85.419 40.681 1.00 0.00 O

TER

END
